# Analysis of Pools of Targeted *Salmonella* Deletion Mutants Identifies Novel Genes Affecting Fitness during Competitive Infection in Mice

**DOI:** 10.1371/journal.ppat.1000477

**Published:** 2009-07-03

**Authors:** Carlos A. Santiviago, M. Megan Reynolds, Steffen Porwollik, Sang-Ho Choi, Fred Long, Helene L. Andrews-Polymenis, Michael McClelland

**Affiliations:** 1 Sidney Kimmel Cancer Center, San Diego, California, United States of America; 2 Department of Microbial and Molecular Pathogenesis, College of Medicine, Texas A&M University System Health Science Center, College Station, Texas, United States of America; University of Washington, United States of America

## Abstract

Pools of mutants of minimal complexity but maximal coverage of genes of interest facilitate screening for genes under selection in a particular environment. We constructed individual deletion mutants in 1,023 *Salmonella enterica* serovar Typhimurium genes, including almost all genes found in *Salmonella* but not in related genera. All mutations were confirmed simultaneously using a novel amplification strategy to produce labeled RNA from a T7 RNA polymerase promoter, introduced during the construction of each mutant, followed by hybridization of this labeled RNA to a Typhimurium genome tiling array. To demonstrate the ability to identify fitness phenotypes using our pool of mutants, the pool was subjected to selection by intraperitoneal injection into BALB/c mice and subsequent recovery from spleens. Changes in the representation of each mutant were monitored using T7 transcripts hybridized to a novel inexpensive minimal microarray. Among the top 120 statistically significant spleen colonization phenotypes, more than 40 were mutations in genes with no previously known role in this model. Fifteen phenotypes were tested using individual mutants in competitive assays of intraperitoneal infection in mice and eleven were confirmed, including the first two examples of attenuation for sRNA mutants in *Salmonella*. We refer to the method as Array-based analysis of cistrons under selection (ABACUS).

## Author Summary

One strategy to define bacterial genes with a role during infection involves the screening of a pool of random transposon insertion mutants, where each mutant is identifiable by a specific motif or unique transcript. Changes in the survival of each mutant indicate a role of the mutated region during infection. To ensure coverage of most genes of interest, a large number of random transposon mutants would be needed. However, when a pool of bacterial mutants spreads into different sites in an animal host, some mutants are lost at random if the founder population (i.e. the bacteria initially reaching this site, before expansion) is smaller than the number of mutants introduced into the animal. This random loss severely obscures mutants that are truly at a disadvantage. In order to minimize the number of mutants to be screened and thus minimize random loss of mutants from an infecting pool, we generated targeted specific deletions in each gene of interest. Furthermore, we inserted a promoter in each mutant that allows simultaneous monitoring of a pool of these mutants on a novel inexpensive microarray. To demonstrate the utility of our technique, a pool of over 1,000 gene-targeted *Salmonella* mutants was injected into mice and recovered from spleens. We identified mutants that were less fit than wild-type *Salmonella* in this model. Eleven mutants in genes that were not previously known to affect *Salmonella* fitness in intraperitoneal infection were confirmed using the individual mutants in competition with wild-type bacteria. These new phenotypes include the first two examples of attenuation and one of hypervirulence in *Salmonella* due to mutations in small stable RNAs, a class of regulators that bind to other RNAs and proteins.

## Introduction

Genetic screening remains one of the most efficient methods to identify genes associated with a phenotype of interest in bacteria. Array-based methods for these screens originated with the transposon-based “signature tagged mutagenesis” (STM) strategy that used unique signature sequences in each transposon to evaluate the relative abundance of individual mutants in pools after selection [1]. STM was later improved by modifying the mutagenizing transposon to include a T7 RNA polymerase promoter (P_T7_) that is used to generate a unique transcript for each mutant from the genomic sequence adjacent to the mutation. This modification makes exogenous unique sequence tags unnecessary. Relative abundance of the input and output P_T7_ transcripts is monitored using an ORF microarray [2],[3],[4],[5].

Transposon mutagenesis suffers from several drawbacks. First, tens of thousands of random transposon insertion mutants are necessary to ensure that mutations occur in most small genes. There are over 1,100 annotated open reading frames in *Salmonella* that are less than 500 bases in length. In mathematical simulations of pools of 40,000 random transposon integrations, over 200 of these short genes fail to be disrupted by any transposon, on average (data not shown). Second, this need for a high complexity is a critical limitation of random mutants for genetic screens in environments, including live animals, where the bacterial population may fall to low levels during infection. These ‘bottlenecks’ may occur at various points during infection – survival of the acidic environment in the stomach, invasion of Peyer's patches and survival in the bloodstream represent some of the processes where the founder population may be very small. Such ‘bottlenecks’ cause undesirable random loss of mutants and complicate forward genetic screening in such environments. In addition, the polar nature of transposon insertions makes mapping of a phenotype more difficult.

We used the lambda-red recombination method that includes features to minimize polarity [6] to construct targeted deletion mutants in *Salmonella*. We added a P_T7_ to the cassette inserted during mutagenesis, positioned to produce a gene-specific transcript from the genomic sequence adjacent to the insertion. Thus, our targeted deletion mutants can be pooled for genetic screens. Far fewer specific mutants are needed to ensure representation of every gene of interest than is needed when using random transposon mutagenesis. We introduce a novel and inexpensive array designed for monitoring changes in specific deletion mutant representation in the pool after selection. Finally, we demonstrate the utility of these techniques by identifying novel candidate genes encoding proteins and sRNAs deletions of which affect fitness in competitive infection with wild-type *Salmonella*, and we validate eleven of these fitness phenotypes.

## Results

### Genome sequencing

The virulent isolate *Salmonella enterica* serovar Typhimurium ATCC14028 is extensively studied both *in vitro* and *in vivo*. To design primers for generation of our deletion collection, we produced a near complete draft sequence of this genome using the 454 shotgun approach (GenBank accession in process). The ATCC14028 sequence was compared to the completed genome of the 1,000-fold less pathogenic laboratory strain Typhimurium LT2 [7]. As expected, over 95% of the two genomes were orthologous, and the orthologous regions had less than 1% divergence. The ATCC14028 and LT2 genomes differ only by a few hundred single base mutations (including a mutation in *rpoS* that is partly responsible for the attenuation of LT2 [8]), the absence of the two Fels phage in ATCC14028, and other insertions and deletions encompassing less than 40 kb (Sandy Clifton et al., unpublished data).

### Generation of specific gene deletions in ATCC14028

We targeted 1,052 genes for deletion (**Table S1**), primarily genes in *Salmonella* that are not found in *E. coli* [7]. Such genes are usually in very A+T rich regions [9], and include nearly all of the ∼200 genes previously associated with *Salmonella* virulence, including the Type III secretion systems (TTSS) and their known effectors. Targeted deletions were also generated in nearly all of the 100 genes in fimbrial and surface antigen regulons. Finally, we deleted a subset of genes shared by *Salmonella* and *E. coli*, including 44 known and candidate sRNAs, and genes that have known motility, regulatory and pathogenesis functions.

**Figure 1:**
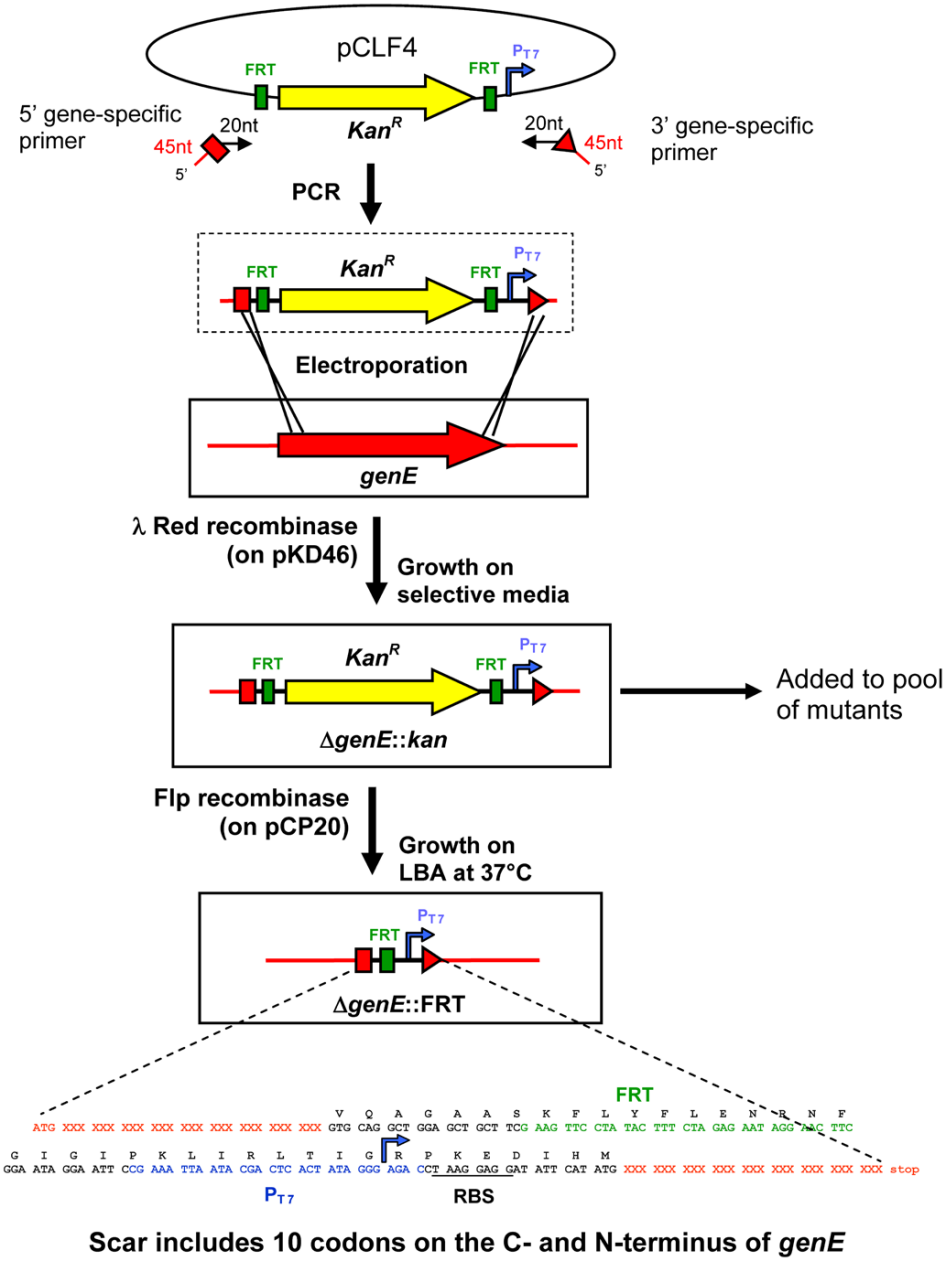
Generation of specific deletions in *S. enterica* serovar Typhimurium ATCC14028. Our procedure is identical to the Red-swap described in reference [6] with the exception that we re-engineered the original insert to include an in-frame T7 RNA polymerase promoter (P_T7_), and the sequences used for recombination are longer. A gene, identified in the schematic as *genE*, is targeted for deletion. Two 65mer primers (red) are used to amplify the region containing the antibiotic resistance cassette, the P_T7_, and the FRT sites (among other elements) from the plasmid pCLF4. The resulting PCR product has 45 base sequences at each end that are homologous to sequences near the 5′ and 3′ ends of the *targeted ORF*. Transformation of these PCR products into ATCC14028 expressing lambda Red recombinase *in trans* leads to a recombination event resulting in the swap-in of the PCR product, and swap-out of the targeted gene. A ribosomal binding site (RBS) and a downstream ATG start codon near the 3′ end of the inserted sequence ensures that a 12 amino acid peptide is made from any RNA that is transcribed in this strand to reduce polar effects. Targeted mutants in many genes were pooled and used for Array-based analysis of cistrons under selection (ABACUS). Finally, antibiotic resistance markers in targeted genes can be removed using the FLP recombinase resulting in a gene encoding a mini-protein of the first ten amino acids of GenE, 39 amino acids from the inserted DNA (called a “scar”), and the last nine amino acids of GenE, and retaining the P_T7_.

The original vectors for the lambda-red swap strategy, pKD3 (Cm^R^) and pKD4 (Kan^R^) [6], were redesigned to include a T7 RNA polymerase promoter positioned to generate a unique transcript from the *Salmonella* genome directly downstream of each mutant. The construct includes an ATG and ribosome binding site (RBS) to preserve any translation coupling. An outline of our variation on the lambda-red swap strategy is shown in **Figure 1**. The sequences of the redesigned vectors, pCLF3 and pCLF4, are available in GenBank (Accession numbers EU629213 and EU629214, respectively).

The oligos used for mutagenesis were 65mers that contained the same 3′ sequence of 20 bases for vector PCR that were used previously [6]. However, they included a 45-base rather than the conventional 35-base gene-specific portion, to allow a secondary function for the oligonucleotides as probes in a microarray (described later). Homology of the oligonucleotides with the genome was positioned so that each swap eliminated the entire gene except for the coding regions of 30 base pairs at the 5′ and 3′ ends of each gene. These two ends of each gene were preserved to minimize unintended effects on adjacent genes caused by removal of the gene sequence and insertion of the mutagenic cassette. If FLP recombination is used to remove the antibiotic cassette after mutant construction then an open reading frame is generated including ten codons from the 5′ end of the original gene, 39 amino acids from the lambda-red mutagenesis referred to as a “scar”, and nine amino acids and the stop codon from the original gene. In our constructs, the T7 promoter remains in place in the scar after FLP recombination.

We also increased the level of throughput in the generation of our mutant collections. Lambda-red swap recombination in ATCC14028 was performed with a mixture of two PCR products, one originating from pCLF4 containing a kanamycin resistance cassette (Kan^R^, sense orientation) and one originating from pCLF3 containing a chloramphenicol resistance cassette (Cm^R^, antisense orientation). Each transformation was plated on LB-Kan and LB-Cm, and two transformants from each plate were colony purified and stored (a total of four transformants). These two collections marked with different antibiotic resistance cassettes facilitate the construction of double mutants by transduction.

The *Salmonella* genes targeted for deletion were spot-checked by PCR. Of 1,052 mutants attempted, 1,040 produced Kan^R^ or Cm^R^ clones, or both of which 1023 were confirmed, below (**Table S1**). To confirm that mutations occurred at the targeted location, two clones obtained for the first 304 Kan^R^ mutants and for 231 Cm^R^ mutants were checked by PCR using primers to the flanking genomic regions. Only one mutation was incorrect.

The accuracy of FLP recombination to remove the antibiotic resistance marker was verified for twelve transformants (as detailed in the Methods and Materials section). The mutant including flanking regions was amplified by PCR, and both strands of the amplification product were sequenced. All twelve swaps examined were precise, and each recombination event resulted in the intended truncated open reading frame.

### Array-based verification of P_T7_ location and activity from a pool of mutants

**Figure 2:**
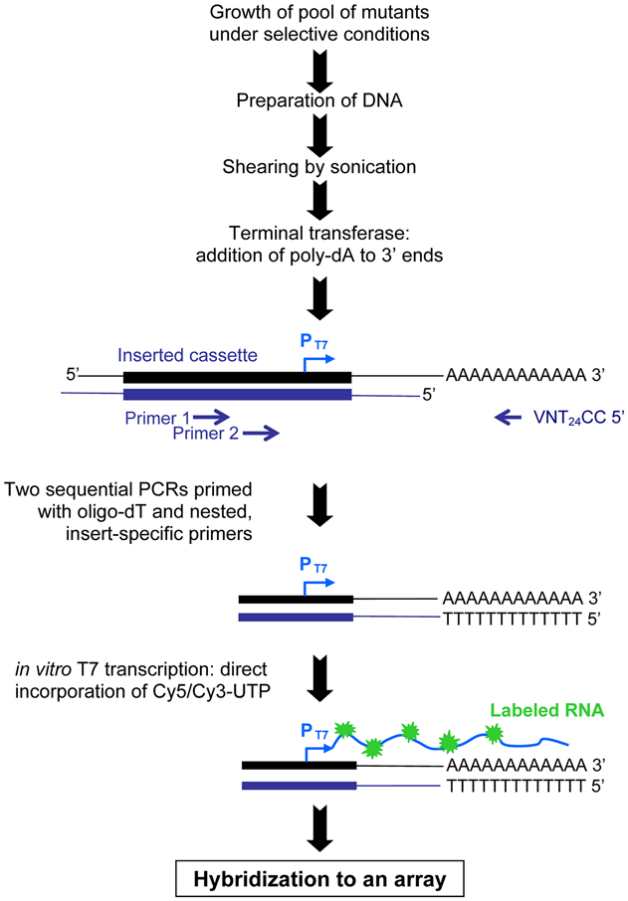
Labeling technique for detection of mutants in a pool by arrays.

We used a NimbleGen tiling array to verify the correct insertion and activity of the P_T7_ in all of our mutants simultaneously. 1,031 Kan^R^ mutants were pooled by growing each mutant separately to stationary phase in LB, mixing in equal volume, and storing as glycerol stocks at −80°C. The region 3′ to each mutation in the pool of 1,031 Kan^R^ mutants was labeled by a novel method designed to generate uniform signals from every P_T7_ (Materials and Methods and **Figure 2**). For the necessary DNA fragmentation prior to amplification and labeling, we replaced the restriction digestion step used in previous protocols with genome shearing and polyA tailing. To identify active P_T7_ in the mutants, labeled RNA obtained by T7 *in vitro* transcription from the pool was hybridized to a custom NimbleGen tiling array of 50mer oligonucleotides covering the ATCC14028 genome in overlapping 24 base steps on both strands. Oligos directly adjacent to each functional insert and in the opposite strand from the transcript hybridized intensely, with a rapid decrease in signal over a 200 base region (**Figure S1**). Using this method we simultaneously identified 933 inserts from Kan^R^ mutants with functional P_T7_ sites in the correct location. 48 additional Kan^R^ mutants are likely correct but could not be formally confirmed, due to an overlapping transcript from a nearby mutation in another mutant in the pool. 50 Kan^R^ mutants (4.8% of all pooled mutants) did not display active transcription at the correct location perhaps due to a mutation in the P_T7_. Four inserts were at an incorrect location.

We repeated these experiments with a pool of 972 Cm^R^ mutants and at least 892 of these have a functional P_T7_. 78 Cm^R^ mutants were in genes where a Kan^R^ clone was not confirmed. Overall, correct Kan^R^ or Cm^R^ mutants, or both, with functional P_T7_ were confirmed in 1,023 genes out of 1052 targeted for deletion. We attempted transduction to construct double mutants of Cm^R^ clones in combination with different Kan^R^ clones. All ten double mutants attempted were successful (data not shown). In addition, the Cam^R^ mutants will also be useful in the future as a pool.

Failure to generate the correct targeted mutation could occur if the mutation was lethal. We did not attempt to delete any genes that are orthologous to genes previously reported to be essential in *E. coli* [10]. However, we did target 38 genes that were previously reported as essential in *Salmonella* [11] and succeeded in obtaining mutants in all but two; *STM1008*, a phage gene that has a close paralogue in the genome, and seems unlikely to be essential in LB, and *STM2087*, encoding an enzyme required for the synthesis of LPS side chains. The reason for the 29 failures to construct a mutant is as yet undetermined.

### Generation and testing of an ‘in house’ microarray for screening

Pools of our mutants can be studied using competitive assays and the representation of each mutant can be determined using microarrays. For such detection, we manufactured an economical “in house” array based on 1,241 65mers derived from the 3′ ends of genes, including all those previously utilized for mutant construction. As noted earlier, these 65mers contained a 45-base gene-specific portion, and oligonucleotides of 45 bases in length produce a more robust RNA hybridization signal [12] than the 35mers that are normally used for lambda-red recombination [6]. As negative controls we included in the array 308 65mer oligos with homology to the 3′ end of mutants that had not yet been generated and were therefore absent from the pool, and 955 65mers designed on the 5′ end of genes (previously used for mutant construction).

One aliquot of the pool of 1,031 Kan^R^ mutants was used to test the “in house” array. Labeled RNA probe was prepared from the pool, and hybridized to the array as described (Materials and Methods). The 5′ ends of these RNA molecules consist of conserved 27 bases common to every mutant. A specific 27 base oligonucleotide complementary to this region was included in the hybridization to block cross-hybridization of this portion of the T7 transcripts to a complementary 20 base sequence present in each of the 3′ gene probes on the array (**Figure S2**). Specific signal at least three-fold over the mean signal of the negative controls was detected for 97% (905) of the 933 mutants known to be in the pool. **Table S2** illustrates the specificity and sensitivity of our approach. Only seven (0.7%) of the 5′ oligos included as negative controls in the array hybridized, possibly due to a transcript from a nearby mutant in the complementary strand.

### Genes that affect fitness of *Salmonella* during passage through BALB/c mice

**Figure 3:**
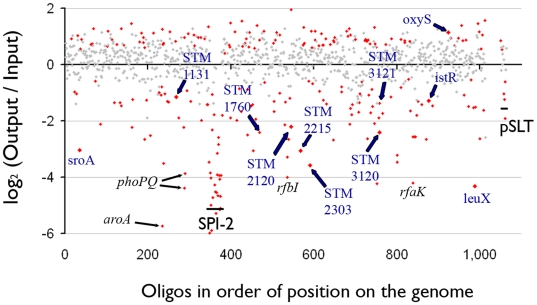
Loss of defined mutants from the input pool after intraperitoneal delivery to six BALB/c mice and recovery from spleen at 48 h post-infection. Twelve array hybridizations were performed using two each for six mice. WebArray analysis results using quantile normalization for all oligos representing candidate deletions in the pool are shown. Data with P<0.0005 are depicted in red, those with P>0.0005 in grey. The X-axis plots the data for each of the mutants in the order in which those mutants occur in the *Salmonella* genome. Mutants confirmed individually in a competitive assay with wild-type are labeled in blue. Examples of important genetic elements are marked in black.

BALB/c mice develop fatal systemic infection from *S. enterica* serotype Typhimurium, as can also occur in humans infected with *S. enterica* serotype Typhi. We used intraperitoneal infection in BALB/c mice, a well-studied model [1],[13],[14],[15],[16], to demonstrate that our mutant screening method could correctly identify both previously observed and novel gene requirements during infection. A group of six BALB/c mice were infected intraperitoneally (IP) and bacteria were recovered from the spleen after euthanasia at 48 hours post-infection. Labeled RNA probe was prepared using aliquots of DNA from the input pool and output pools from each animal as outlined in **Figure 2**, and the labeling protocol was repeated with dyes reversed. Hybridizations were performed using twelve slides, each slide containing the array printed in triplicate. The data were processed using WebArray [17], as described in Materials & Methods, and are presented in **Table S1**, and visualized by genome position in **Figure 3**.

**Figure 4:**
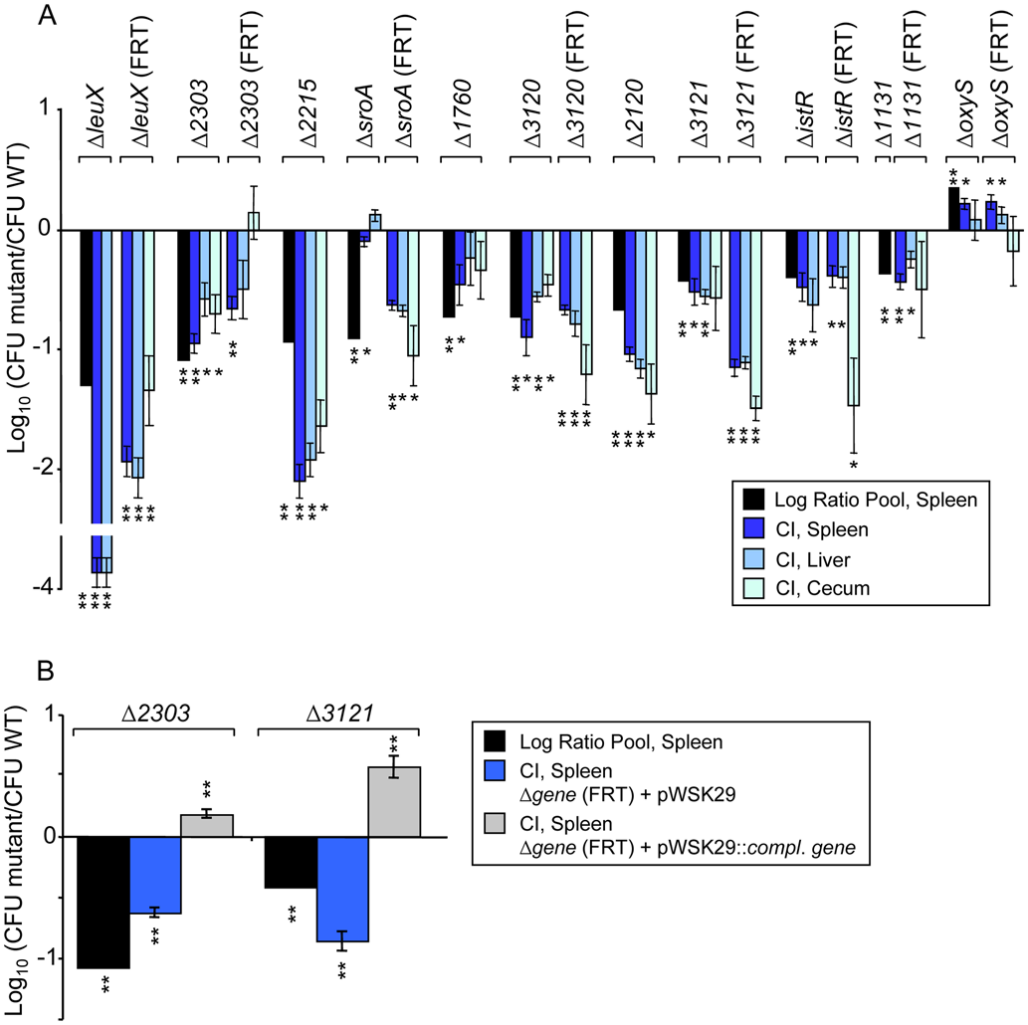
Competitive Index experiments with individual mutants versus wild-type after intraperitoneal delivery and recovery from the spleen, liver, and cecum. 10^6^ cfu of 933 mutants (pool) or a 1∶1 mixture of individual mutants versus wild-type ATCC14028 Δ*phoN*::*kan* were injected into 5–6 mice and recovered from spleen one or two days post infection. Statistical significance was determined using a Student's 2-tailed *t* test, and asterisks indicate that normalized output ratios were significantly statistically different from the equivalent ratio in the inoculum. ** P value of <0.001, * P value of <0.05. Strains marked with (FRT) have undergone FLP-mediated recombination to reduce or eliminate polar effects. A. Confirmation of mutants, identified by ABACUS, in competitive infections in BALB/c mice. ABACUS prediction for selected mutants (black bars) and confirmation data from competitive infections in BALB/c mice (dark blue bars are from spleen, lighter blue are from liver and cecum) of infected animals. B. Complementation of *ΔSTM2303*::FRT and *ΔSTM3121*::FRT mutants *in trans*. The predictions from ABACUS are indicated as black bars. For complementation experiments animals were infected with either a 1∶1 mixture of the mutant bearing pWSK29 vs. ATCC14028 Δ*phoN*::*kan* (negative control, blue bars) or the mutant bearing the corresponding complementing gene on pWSK29 vs. the wild-type ATCC14028Δ *phoN*::*kan* (complemented, grey bars).

At a threshold of a two-fold change and P<0.0005, 120 mutants showed a change in fitness in IP infection. Among the most unfit were mutants in the TTSS encoded by SPI-2, and associated effector genes, and genes for cell wall biosynthetic enzymes, all known to be important during systemic infection [1],[13]. None of these mutants have a change in fitness during growth of the pool in LB for four serial passages to stationary phase with 100-fold dilutions (data not presented). Fifteen mutants without previously known fitness phenotypes during systemic infection in BALB/c mice were selected from the 120 statistically top-ranked candidate mutants. These included mutants in all five candidate sRNA, a supernumerary tRNA (LeuX), and nine randomly picked protein coding genes. These 15 mutants were studied one at a time in competitive assays with wild-type ATCC14028, after P22 transduction of each mutation to a clean genetic background. The phenotypes observed in the pool of mutants were confirmed for eleven mutants, as listed in **Figure 4** and **Table S3**. Four mutants failed to reiterate the phenotype observed in the mutant pool (*sraA*, *rybB*, *STM4529*, and *STM0857*) but this result does not rule out confirmation of a phenotype in a study involving more animals.

All eleven mutants with confirmed competitive index phenotypes had their Kan^R^ cassette removed by FLP recombination to reduce the chance of polar effects on nearby genes. Eight of these (*STM1131*, *STM2303*, *STM3120*, *STM3121*, *sroA*, *istR*, *leuX*, and *oxyS*) were retested in competitive infections in BALB/c mice (**Figure 4A**). The phenotype was confirmed in all eight of these unmarked mutants that should have minimal or no polar effects.

In order to definitively link the deleted genes to the reduced fitness phenotype, two of our unmarked (“FLP-ed”) mutants (Δ*STM2303*::FRT and Δ*STM3121*::FRT) were re-tested after complementation *in trans*. Intact copies of these genes were cloned into a stable low copy plasmid vector, pWSK29 [18], and the complementing plasmids were transformed into the corresponding unmarked deletion strains. The competitive fitness defect of both mutants was reversed when these strains were complemented with an intact copy of the corresponding gene *in trans* (**Figure 4B**). The presence of the vector alone did not improve the fitness of these two mutants during infection.

## Discussion

Targeted deletion by lambda-red recombination was previously used to construct a library of specific gene deletion mutants for *E. coli* [10]. Here, we use this approach for *Salmonella*, and modify it by adding a T7 RNA polymerase promoter to the insert for the generation of unique transcripts from each mutant. These transcripts are used to identify and measure the relative abundance of each mutant from a pool containing all of our mutants. Furthermore, we introduce several technical innovations to improve the throughput of library construction, and the labeling and detection of transcripts from each mutant on an inexpensive, customized microarray. We use a well-studied model, systemic infection of BALB/c mice, to validate our assay and identify novel candidate genes that affect the fitness of *Salmonella* in that infection model.

Our targeted deletion strategy has several important advantages over screening of pools of mutants made by random transposon mutagenesis. Screening targeted deletion pools reduces the complexity of the bacterial pool necessary to cover all genes of interest by at least 10-fold over random transposon mutant pools; mathematically, 40,000 random transposon mutants cover about 90% of all non-essential *Salmonella* genes, whereas 4,000 specific mutants in each non-essential gene provides 100% coverage of the targeted genes. This less complex pool is highly advantageous for forward genetic strategies in circumstances where population sizes drop during the selection process, such as in animal models [4],[19],[20],[21],[22]. For *Salmonella*, this “bottleneck” is particularly severe in the transition from the intestinal tract to the systemic circulation [23],[24] and perhaps in different parts of the gastrointestinal tract where niche conditions can vary considerably. These bottlenecks cause random loss of mutants from the pool if the population falls to numbers close to the complexity of the interrogated pool. For example, if a population falls transiently to 40,000, then a pool of 4,000 should not lose any mutants (P<0.01 for loss of one mutant). However, 40,000 mutants will lose about 37% of its members at random, because the probability of not picking a mutant that has a frequency of 1/40,000 in 40,000 attempts is (1-1/40,000)^40,000^ = 0.37. Furthermore, a pool of lower complexity may be used at a lower infectious dose, allowing a more physiologically relevant infection. The smallest possible pool that includes each mutant of interest is therefore highly desirable.

In any particular experimental system the size of the inoculum needed to avoid bottlenecks will have to be determined empirically. It is possible to determine the optimum size of the inoculum and ascertain whether or not there is a bottleneck by including a “neutral” mutant carrying a different antibiotic marker at the same CFU as the average mutant CFU in the pool in each experiment. If the representation of this marked strain varies dramatically (either highly over- or under-represented) in the recovered samples in this “fluctuation test”, then the inoculum is of insufficient size to prevent random loss from a pool of that complexity (McClelland et al., unpublished data).

Random transposons can generate pronounced downstream effects that contribute to selection. Our targeted in-frame deletions are engineered to minimize or eliminate these effects. Desirable existing features of the cassette inserted by lambda-red recombination [6], such as an internal ATG start codon at the end of the inserts to allow downstream translational coupling, and the inclusion of FRT sites for the removal of the antibiotic cassette by FLP recombination, remain intact. In our construct, FLP recombination results in the production of an in-frame mini-protein that uses the original start and stop codons of the deleted protein, while retaining the P_T7_. The first and last 30 bases of each targeted gene were maintained to reduce the chance that targeted deletions disrupt adjacent genes, overlapping genes, and *cis*-acting sequences thus further minimizing the risk of polar effects. Subsequent analyses should consider that in rare cases the remaining mini-protein may retain some function. Finally, confirmation of mutants identified by genetic screening requires the generation of targeted mutations. A targeted deletion library allows independently generated mutants in the gene of interest to simply be picked from the ordered collection of mutants for further analysis.

Earlier labeling strategies for T7 transcripts from transposon libraries use restriction digestion to fragment the genomic DNA, which has the effect of producing shorter labeled transcripts that are easily mapped near the P_T7_ [2],[3],[4],[5]. This approach results in non-uniform labeling of transcripts, because those transposons that are not in the ideal proximity to a restriction site are poorly sampled. Our labeling strategy uses randomly sheared ends, resulting in more uniform and consistent labeling efficiency for every mutated gene.

The presence of a P_T7_ promoter in each targeted mutant allows the presence and the level of each individual mutant to be tracked when mutants are pooled, using the mutant-specific transcript produced from this promoter. This innovation eliminates the need to introduce a different ‘signature’ sequence in each of the mutants in the collection. The P_T7_ facilitates efficient RNA synthesis only from those DNA fragments that contain P_T7_. Any artifacts that might be generated during the tailing and PCR steps are not transcribed. Our P_T7_ procedure generates primarily single stranded labeled RNA, minimizing competition for hybridization to the oligonucleotide array, which might be one reason that the performance of this protocol was consistently superior to direct labeling of PCR products in detection sensitivity and in the standard deviation of measurements, as measured by microrray (data not shown).

The presence of the T7 RNA polymerase promoter in each mutant construct allowed us to check all candidate mutants simultaneously for the location of a functional P_T7_. Using a NimbleGen tiling array of 387,000 oligonucleotides, we confirmed 933 of 1,031 mutants in the Kan ^R^ pool as present in exactly the correct place, and functional, simultaneously. Similarly, 892 of 972 Cm^R^ mutants were confirmed using this technique. Thus, we bypassed much more expensive and laborious traditional PCR confirmation. We also confirm the activity of the P_T7_, which is not possible using PCR.

For routine hybridizations of the pool, we constructed an inexpensive oligonucleotide microarray using the same oligonucleotides that we used to construct the mutants, containing 45 bases of homology to the *Salmonella* genome. This array could also be used to confirm the presence of nearby active P_T7_ in a pool. However, unlike the NimbleGen array, this minimal array does not prove the location of the inserted cassettes, or find extraneous insertions that occur elsewhere in the genome. Fortunately, the more expensive NimbleGen arrays need only be used once to determine which mutations are in the correct location and have an active P_T7_. After the library is characterized, the less expensive in-house arrays can be used for further experiments without ambiguity. We expect that multiplexed high-throughput sequencing of experimental samples will replace arrays as the method for assessing changes in the T7 transcripts as soon as prices become competitive.

In order to test our targeted deletion collection and novel protocols, we used systemic infection of BALB/c mice, a model system that has been studied extensively using transposon libraries [1],[13],[14],[15],[16]. All the mutants in our collection that were reported to have attenuated growth in these previous transposon assays were confirmed in our assay, including those in SPI-2 and lipopolysaccharide biosynthesis genes. In addition, among the 120 mutants showing the strongest fitness phenotype we identified more than 40 candidates with no previously known phenotype in this model, although many of these new candidate phenotypes are generally more subtle than those seen for SPI-2 (**Table S1**, **Figure 3**).

We retested fifteen of the novel mutants with at least a 2-fold change in representation between the input and output pool (P<0.0001) in individual competitive infections, and eleven were confirmed in mutants containing the antibiotic cassette. We retested eight of these mutants (*STM1131*, *STM2303*, *STM3120*, *STM3121*, *sroA*, *istR*, *leuX* and *oxyS*) after removal of the antibiotic resistance cassette. The reduced competitive fitness seen in the mutants containing an antibiotic resistance cassette was also seen in all eight of these mutants after removal of this cassette (**Figure 4A** and **Table S3**). We complemented two of these novel mutants, *STM2303* (*pmrM*) and *STM3121* (a LysR-type transcriptional regulator), with the corresponding intact open reading frames *in trans*. The complemented mutants were found in the spleen in numbers similar or greater than the wild-type (**Figure 4B**). These findings directly and unambiguously link the genes we complemented here to the observed defect in fitness of the corresponding mutant during competitive infection with the wild-type. Thus, most of the remaining 25 mutants of the original top 40 novel candidates that we identified may have a phenotype in this BALB/c acute systemic infection model. There are likely to be further phenotypes yet to be discovered among the many genes that were not mutated for the experiments performed here.

Using our method, the overall false negative rate (failure to observe phenotypes for mutants already known to be under strong selection in this model and measured on the array) was zero. Among 120 mutants showing a phenotype with P<0.0001 and a greater than two-fold change, more than half are already known to have a phenotype and among the new candidates, eleven out of fifteen were confirmed. Thus, the false discovery rate is very low. However, the false discovery rate for new mutants in a system that is already well studied is invariably high. When most genes with a role in a process are known, only a few phenotypes remain to be discovered among the thousands of genes that have no phenotype in that process. The future rate of discovery in the intraperitoneal model in BALB/c mice is unlikely to be better than the rate of 11 out of 15 that we experienced. The rate of confirmation of mutants that show a weaker statistical confidence and lower fold-difference in fitness is likely to be worse. In addition, those mutants with subtle phenotypes in an animal will require a larger number of biological replicates in order to establish the phenotype with confidence. Other phenotypes that will be missed by using a pool of mutants are any of those rescued, *in trans*, by the presence of wild-type bacteria.

Of the mutants we confirm in individual competitive infections with wild-type, four were in small RNA molecules. Mutations in *leuX*, encoding a minor tRNA-Leu, were previously known to reduce the expression of Type I fimbriae, and reduce bladder epithelial invasion and intracellular proliferation of Uropathogenic *E. coli* [25],[26]. The attenuation of a *leuX* deletion during *Salmonella* infection was previously unknown. Mutants in the regulatory sRNAs IstR, OxyS and SroA showed small, but reproducible, phenotypes in our *in vivo* experiment, and all have orthologs in *E. coli*. In *E. coli*, IstR inhibits the translation of *tisAB*, an SOS-induced toxic peptide, and may arrest growth allowing DNA repair [27],[28],[29],[30]. SroA is a known regulator of the *thiBPQ* operon in *E. coli*, that encodes an ABC transport system for the import of thiamine and thiamine pyrophosphate (TPP) into the cell [31]. TPP is an essential cofactor for key enzymes in carbon metabolism.

The *oxyS* mutant was one of the few examples of a mutant that displayed an increase in fitness *in vivo* in our screen. OxyS is a member of the OxyR regulon expressed during oxidative stress. This sRNA is a pleiotropic regulator of about 40 genes in *E. coli* including *rpoS*, and an anti-mutator that may inhibit alternate stress adaptation pathways when OxyR is activated [30],[32]. Our observations for OxyS, SroA, and IstR, are the first examples of phenotypes for small non-coding RNA mutants during *Salmonella* infection, other than the tmRNA system that targets proteins for degradation [33]. The targets of these sRNAs in *Salmonella* are not fully known, and given, for example, the recently reported variable composition of the PhoP regulon across related organisms [34], may differ significantly from their targets in related organisms.

Of the seven remaining confirmed mutant phenotypes, four are in genes that encode putative membrane proteins (*STM1131*, *STM2120*, *STM2215 and STM2303*), and a fifth gene (*STM1760*) encodes a putative secreted protein. *STM1131* encodes a member of the KdgM superfamily (oligogalacturonate-specific porin). A paralog of this gene, *STM4016* (*yshA*, 26% identity, 40% similarity) is involved in O-Antigen capsule production and environmental persistence [35]. Mutants in a second membrane protein, *STM2120* (*asmA*), are highly sensitive to gastric contents of swine, but otherwise little is known of the function of this gene in *Salmonellae* [36]. Mutants in the *E. coli* orthlog of *asmA* are extragenic suppressors of outer membrane protein assembly and have less LPS on the bacterial surface [37]. A third gene encoding a predicted membrane protein, *STM2215*, is one of at least eight proteins encoded by Typhimurium that contain cyclic-di-GMP phosphodiesterase, or EAL domains (named after the amino acids in the conserved domain). Unlike several other EAL-containing proteins, mutations in *STM2215* do not influence a multicellular behavior known as the rdar morphotype [38]. Our work reports the first phenotype of any kind for *STM2215*.

Finally, *STM2303* (*pmrM*; *pbgE3*) is the terminal gene in the *pmr* operon (polymyxin resistance) and also encodes a predicted membrane protein. This operon is involved in 4-aminoarabinose addition to Lipid A, a modification that confers resistance to polymixin [39]. Paradoxically, PmrM does not appear to be necessary for this function [39],[40], but may be important for resistance of *Salmonella* to high iron concentrations *in vitro* [41],[42]. Recent studies show that mutants in *STM2303* have reduced expression of *hilA*, an important regulator of virulence genes in *Salmonella*, and reduced invasion in T84 human colonic epithelial carcinoma cells [43].

Mutants in *STM1760*, encoding a protein containing a classical N-terminal secretion signal sequence, were also confirmed as selected against in our competitive infection assays. The protein encoded by this gene contains multiple Tricopeptide Repeats (TPR) of the Sel-1 superfamily, a motif that mediates protein-protein interactions [44]. Nothing further is reported in the literature about this protein and its potential role during systemic infection by *Salmonella*.

The final genes we confirmed to be important during systemic infection in BALB/c mice, *STM3120* and *STM3121*, are neighboring in the genome. *STM3121* is a LysR family transcriptional regulator neighboring *STM3120* but is transcribed in the opposite direction. Interestingly, expression of *STM3121 in trans* appeared to increase fitness during infection (**Figure 4B**). *STM3121* has not previously been reported to be important during Typhimurium infection, and determination of its regulatory targets may reveal clues as to its role during infection.

*STM3120* and several adjacent genes (cluster *STM3120*-*STM3117*) are present in serotypes Typhimurium, Enteritidis, and Gallinarum [45] and in some *Yersinia* [46], but are not present in the typhoidal *Salmonellae* or other enterobacterial species studied to date. A second gene in this group, *STM3119*, was also selected against during our ABACUS screen (**Table S1**). Proteomic and transcriptional data indicates that the proteins encoded in this region are highly up-regulated in macrophages [47],[48],[49]. In serotype Enteritidis, these genes are essential for growth in chicken macrophages and mutants in some of these genes in Gallinarum are defective for systemic colonization after oral infection in chickens [45],[50].

Although *STM3120* is a predicted *citE* (citrate lyase) homolog, the other two subunits (*citD* encoding the α-subunit, and *citF* encoding the γ-subunit) that normally make up bacterial ATP-dependent citrate lyase are not encoded in the region containing this gene. This suggests that *STM3120* may have a different function than a traditional citrate lyase. In *Yersinia pestis*, homologs of *STM3120-3117* are necessary for survival in macrophages that are activated with γ-interferon after infection. These genes appear to function in *Y. pestis* by lowering the level of nitric oxide in macrophages without affecting iNOS levels in γ-interferon post-treated macrophages [46]. It is possible that these genes play a similar role in *Salmonella*, but this hypothesis remains to be investigated.

To summarize, we have designed a collection of novel technologies that permit easy generation and confirmation of specific deletion mutants for genetic screening, both individually and in pools. Use of our collection, designed to minimize the effects of population bottlenecks on screening, will allow relevant animal models and more relevant doses to be efficiently used to identify *Salmonella* mutants with altered fitness *in vivo*. During our testing of this approach, eleven of such mutants were confirmed in individual competition assays, including three sRNAs and *leuX*. The tools used here are a first step to a more complete description of *Salmonella* genes involved in systemic infection in mice, particularly those genes with milder phenotypes that were difficult to identify with confidence in previous studies. These tools are also of wide applicability to identify genes involved in other aspects of *Salmonella* biology. Finally, similar strategies can be applied in other genetically malleable bacteria.

## Materials and Methods

### Ethics statement regarding the use of animals, choice of species, and numbers of animals

The search for new mechanisms in infection has no *in vitro* surrogate. Mice are natural hosts for *Salmonella* and are also a potential source of human infection. The library and pooling strategies with microarray output (ABACUS) reduces the number of animals required to obtain mutant phenotypes by at least three orders of magnitude compared to screening individual mutants.

### Veterinary care

All animals were housed in AAALAC-accredited animal research facilities at Texas A&M University and were observed daily for assessment of animal health. The facilities are in compliance with the standards set forth in the PHS “Guide for the Care and Use of Laboratory Animals”.

### Methods for pain and stress relief

If during the course of these experiments any animal showed anorexia for greater that 24 hr., disinclination to move for 12 hours, loss of normal neurological function as evidenced by locomotory or balance deficits then euthanasia was performed using CO_2_ (>70%, inhaled) in accordance with the 2000 AVMA Guide for Euthanasia.

### Strains and standard culture conditions

All strains used in this study are derivatives of *Salmonella enterica* serovar Typhimurium ATCC14028 (Manassas, VA). Strains were routinely cultured in Luria-Bertani (LB) broth and plates, supplemented with 50 mg/l Kanamycin (Kan), 20 mg/l Chloramphenicol (Cm) or 100 mg/l Ampicillin (Amp) where appropriate.

### Construction of plasmids with T7 RNA polymerase promoters

We used a PCR-based strategy to include a T7 promoter in the original pKD3 (Cm^R^) and pKD4 (Kan^R^) vectors for the lambda-red recombination method. Briefly, pKD3 and pKD4 were used as template for independent PCR reactions using primers PT72_EcoRI (ACTCGAA**TTC** **CGAAATTAATACGACTCACTATAGGGAGAC**CTAAGGAGGATATTCATATG) and FRT3-EcoRI (CATCGAATTCCTATTCCGAAGTTCCTATTCTCTAGAAAGTATAGGAACTTCGGCGCGCCT) or primers PT72-EcoRI and FRT4-EcoRI (CATCGAATTCCTATTCCGAAGTTCCTATTCTCTAGAAAGTATAGGAACTTCAGAGCGCT), respectively. The *Eco*RI sites are underlined and the P_T7_ is in bold. Each PCR product was digested with *Eco*RI, gel purified and self-ligated. Electrocompetent *E. coli* EC100D *pir*-116 (Epicentre), was transformed with aliquots of the ligation, and transformants were selected at 37°C on LB Cm or LB Kan plates. The sequence (both strands) of the resulting plasmids pCLF3 and pCLF4 was determined by the conventional dye-terminating Sanger method, and deposited under GenBank accession numbers EU629213 and EU629214, respectively.

### Generation of specific deletion mutants

Deletion strains were generated using the lambda-red recombinase method [6], with the following modifications, as illustrated in **Figure 1**. Plasmids pCLF3 (Cm^R^) and pCLF4 (Kan^R^) were used as templates to generate unique PCR products for deletion of each gene of interest. The primers used are shown in **Table S1**. PCR amplifications were carried out in 96 well plates using ExTaq polymerase (Takara) in a total reaction volume of 60 µl, and a PCR reaction of 30 cycles at an annealing temperature of 55°C. For each gene to be deleted, 30 µl of both PCR products bearing different antibiotic markers were combined and purified using the Qiaquick PCR purification kit in 96 well format (Qiagen). Mixed, purified PCR products (10 µl) were dialyzed against sterile water for 10 minutes using filters with 0.025 µM pore size (Millipore). 2–5 µl of mixed, purified PCR products were used for transformation by electroporation (Bio-Rad Gene Pulser) of electrocompetent ATCC14028 expressing lambda-red recombinase, prepared as previously described [6]. Transformations were allowed to recover for 3 hours in LB at 37°C and 150 µl of each transformation was plated on LB Kan and LB Cm plates. The remaining transformation was saved and replated when very few colonies were obtained using the shorter grow out period. Two transformants for each gene and each antibiotic resistance marker were twice colony purified, and stored in 30% glycerol at −80°C. The Kan^R^ mutants characterized here are available to the community.

### Labeling technique, part I: DNA isolation, sonication and polyadenylation

Our labeling protocol is outlined in **Figure 2**. Genomic DNA was prepared for the input and output pools of ∼1000 mutants using the GenElute Bacterial Genomic DNA kit (Sigma). 4 µg of genomic DNA was fragmented by sonication using twenty pulses of two seconds in a Branson Sonifier 150 (Branson Ultrasonics Corp., Danbury, CT). PolyA tails were added to fragmented genomic DNA using terminal transferase (TdT) as follows: 1.5 µg of DNA fragments were incubated for 30 min at 37°C in a total reaction volume of 50 µl containing 40 U TdT (New England BioLabs.), CoCl_2_ 0.25 mM, and dATP 0.4 mM. Terminal transferase was subsequently inactivated at 70°C for 10 min and the tailed product was purified using the QIAquick PCR purification kit (Qiagen).

### Labeling technique, part II: PCR amplification and labeling of fragments adjacent the deletion location

Nested PCR was used to amplify the polyA-tailed DNA fragments containing the insert P_T7_ and the flanking inserted region. In the first PCR reaction, 50 ng of purified polyA-tailed DNA was used as template for a PCR reaction using primer FRT Out 3-1 (TTCCTATACTTTCTAGAGAA), and a primer designed to anneal to the polyA-tail (CCT_24_VN). The reaction mixture consisted of 1× PCR buffer, 0.2 mM of dNTP, 1.5 mM MgCl_2_, 0.05 U Taq polymerase (Promega, WI), and 0.2 µM of each forward and reverse primer in a total reaction volume of 25 µl. The PCR reaction was performed under the following conditions: initial denaturation at 94°C for 1 min followed by 30 cycles with denaturation at 94°C for 10 s, annealing at 50°C for 10 s, and extension at 72°C for 5 s. The last cycle was followed by a final extension for 3 min at 72°C. In the second amplification step, a nested PCR was performed using 1 µl amplified product from the initial PCR in a total volume of 50 µl. Internal primer FRT Out 3-2 (TAGGAACTTCGGAATAGGAA) and primer CCT_24_VN were used under identical cycling conditions as during the initial PCR reaction. PCR products were analyzed on 1% agarose gels.

An aliquot of 8 µl of the nested PCR reaction was used directly as template for a 20 µl *in vitro* transcription reaction using the AmpliScribe T7 transcription kit (Epicentre), following the manufacturer's protocol with some modifications. The RNA was labeled during the synthesis by including 2 µl of 5 mM Cy5- or Cy3-UTP (GE Healthcare) in the *in vitro* transcription reaction. Labeled RNA was treated with RNase-free DNase (Epicentre) and purified with the RNeasy Mini Kit (Qiagen).

### NimbleGen microarray hybridization

Hybridizations were performed according to the manufacturer's protocols (NimbleGen Systems, Madison, WI, http://www.nimblegen.com/products/lit/lit.html, cgh_userguide_2008_05_271.pdf) with some modifications. For each hybridization, 4 µg of labeled RNA was mixed with alignment oligo, NimbleGen hybridization components and hybridization buffer. The arrays were hybridized at 42°C for 16 hours. Arrays were washed according to the manufacturer's protocol, and scanned using a GenePix 4000B laser scanner (Molecular Devices, Sunnyvale, California) at 5 µm resolution. The signal intensities were quantified using NimbleScan software v2.4 (NimbleGen Systems). The data was normalized and analyzed using Webarray [17] and WebarrayDB (www.webarraydb.org), with quantile normalization. The moving median intensity of five adjacent probes on the same strand in the genome was calculated and plotted against the genome annotation. Peaks were identified visually (**Figure S1**).

### In-house array hybridization

The 933 65mer 3′ oligos and over 1000 control oligos were printed at 10 µg/ml in 50% DMSO on Corning UltraGap II slides in triplicate arrays. Oligo sequences are in **Table S1**, and the arrays deposited in GEO as platform GPL5687 and GPL5688. Array hybridization was performed in hybridization chambers (Corning Inc., Corning, NY), following protocols suggested by the manufacturer for hybridizations in formamide buffer for pre-hybridization, hybridization, and post-hybridization washes (http://www.corning.com/Lifesciences/technical_information/techDocs/gaps_ii_manual_protocol_5_02_cls_gaps_ 005.pdf). Immediately before hybridization, 2 µg of the labeled probes for input and 2 µg of each experimental sample were unified in 36.5 µl, mixed with 3.5 µl of 100 µM 27mer competitor oligo (CATATGAATATCCTCCTTAGGTCTCCC) complementary to the 27 bases of RNA produced by every mutant (**Figure S2**), and 40 µl of 2× hybridization buffer (50% formamide, 10× SSC [1× SSC is 0.15 M NaCl plus 0.015 M sodium citrate], 0.2% sodium dodecyl sulfate) and then denatured at >96°C for 5 min. The probes were hybridized to the *Salmonella* microarray overnight at 42°C in a water bath.

After overnight incubation, slides were washed and scanned using the ScanArray 5000 laser scanner (Packard BioChip Technologies, Billerica, Mass.) with ScanArray 2.1 software. Fluorescence signal intensities were quantified using the QuantArray 2.0 software package (Packard BioChip Technologies). The data were subsequently analyzed using Webarray [17] (www.webarraydb.org), with quantile normalization.

### Recombination between FRT sites using FLP recombinase

Antibiotic resistance cassettes were removed from targeted deletion mutants as previously described [6]. Briefly, each mutant was transformed with the temperature-sensitive plasmid pCP20, that encodes FLP recombinase [51]. Transformants bearing the plasmid were selected at 30°C on LB Ampicillin plates. Several colonies from each transformation were streaked twice and grown at 37°C on LB plates (without antibiotics). Loss of the antibiotic resistance cassettes was determined by patching candidate strains on LB containing the appropriate antibiotics (either Kan or Cm). The concomitant loss of plasmid pCP20 was confirmed by patching the candidate mutants on LB Amp plates.

In our mutant collection, the recombination event leaves ten codons from the 5′ end of the original gene, a 39 codon region containing the P_T7_ (referred to as a “scar”), and the final ten codons of the original gene (**Figure 1**). Scarred derivatives of mutants Δ*STM0854*::*kan*, Δ*STM1402*::*kan*, Δ*STM2840*::*kan*, Δ*STM2884*::*kan*, Δ*STM2581*::*kan* and Δ*STM2581*::*cam* were generated to ensure the accuracy of FLP recombination. Two independent examples of each mutant were picked and the presence of each scarred mutant allele was confirmed by PCR amplification using primers flanking mutation, and the subsequent sequence of both strands of the PCR product was obtained by the conventional dye-terminating Sanger method. Non-resistant scarred derivatives of mutants Δ*leuX*::*kan*, Δ*sroA*::*kan*, Δ*istR*::*kan*, Δ*oxyS*::*kan*, Δ*STM1131*::*kan*, Δ*STM2303*::*kan*, Δ*STM3120*::*kan*, and Δ*STM3121*::*kan* were generated for further testing in mice.

### Construction of complementing plasmids

*STM2303* was amplified for complementation using the following primers: *STM2303* forward primer 5′ GGCGTCTGGTACCAATTCAGTAT 3′, and *STM2303* reverse primer 5′ TGTTCTGGATCCGTGCGATAGC 3′. The resulting 947 base pair PCR product, in which the only full length ORF is *STM2303*, was purified using agarose gel electrophoresis and extracted from the gel using the Qiaquick gel extraction kit (Qiagen). *STM3121* was amplified for complementation using the following primers: *STM3121* forward primer 5′ GCTATTTTCAGGGTACCGTTTGGTCG 3′, and *STM3121* reverse primer 5′ GCTCCGTTAGCGGATCCTTTAGACAC 3′. The resulting 1,266 base pair PCR product was purified by agarose gel electrophoresis, and extracted from the gel using the Qiaquick gel extraction kit (Qiagen). The PCR product contains the full-length *STM3121* open reading frame (879 bp) with approximately 296 bp of upstream and 90 bp of downstream sequence.

Purified PCR products were digested with *Kpn*I and *Bam*HI and ligated into pWSK29 previously cut with the same enzymes and gel purified. *E. coli* DH5α was transformed with each ligation, and positive clones were selected on LB plates containing Ampicillin. Plasmids bearing the correct insert from each cloning were found by identification of fragments of the proper size after digestion with *Pvu*I. pWSK29, pWSK29::*STM2303*, and pWSK29::*STM3121* were transformed into *S.* Typhimurium strain LB5000 (restriction−, modification+) [52]. Plasmid DNA was prepared from LB5000 plasmid-bearing transformants using the Qiaprep spin Miniprep kit (Qiagen), and purified plasmid DNA was used to transform *ΔSTM2303*::FRT and *ΔSTM3121*::FRT mutants using heat shock. Plasmid-bearing deletion mutants were selected on LB Amp plates, and were twice colony purified prior to use for infections.

### Animal studies in BALB/c mice

The pools of ∼1,000 mutants used as inocula were grown overnight at 37°C in LB with aeration and serially diluted in PBS to the proper concentration for inoculation, and the titer of the inoculum was determined by plating on LB containing Kanamycin. A group of 6 BALB/c mice (8–10 week old female) was infected intraperitoneally with 1×10^6^ CFU of the pool in 100 µl PBS. Mice were monitored twice daily for signs of infection, and were humanely euthanized at 48 hours post infection. Immediately after euthanasia, the spleen was removed and homogenized in 5 ml sterile ice cold PBS. 100 µl of the spleen homogenate was used for serial dilution and titer on LB Kan plates. The bacteria in the remaining homogenate were grown in LB to stationary phase and total DNA was extracted.

Individual mutants with an apparent phenotype in the pools were chosen for confirmation in mixed infections with wild-type strain HA431 (ATCC14028 *phoN* Nal^R^), to determine the competitive index. Inactivation of *phoN*, encoding alkaline phosphatase, abolishes the ability to cleave 5-bromo-4-chloro-3-indolyl phosphate (XP), but does not reduce the ability of serotype Typhimurium to colonize organs or reduce fecal shedding of this organism in mice during competitive infections [53]. Growth on LB agar plates supplemented with XP thus provided an easy means to distinguish between individual mutants (PhoN^+^ blue colonies) and HA431 (PhoN^−^ white colonies).

Prior to further analysis, the mutant allele present in each individual mutant to be confirmed was transferred to HA420 (ATCC14028 Nal^R^) by P22 transduction. Mutant strains and our wild-type strain HA431 were grown overnight at 37°C with aeration, mixed in a 1∶1 ratio, and serially diluted in PBS to the proper concentration for inoculation and titer. Titer was used to determine the exact ratio of strains administered. Groups of 4–6 BALB/c mice (8–10 week old female) were infected intraperitoneally with 1×10^6^ CFU of mutant and wild-type virulent HA431 in 100 µl PBS. Mice were euthanized and spleens, livers, and ceca were recovered as above. Mutant and wild-type organisms were enumerated by serial dilution and plating on LB plates containing 20 mg/L of XP.

## Supporting Information

Figure S1 NimbleGen tiling array data for T7 RNA polymerase transcripts from a pool of 1031 Kanamycin resistant specific deletion mutants.
(3.18 MB PDF)

Figure S2 Addition of the 27mer competitor oligo results in high specificity of array detection.
(0.10 MB PDF)

Table S1 Mutants constructed and results of selection *in vivo*.
(0.57 MB XLS)

Table S2 Specificity and sensitivity of array-based detection of mutants in a pool.
(0.01 MB PDF)

Table S3 Details of the Competitive Index data.
(0.03 MB PDF)
