# Supplementary material for: Analysis of Pools of Targeted Salmonella Deletion Mutants Identifies Novel Genes Affecting Fitness during Competitive Infection in Mice
Source: PLoS Pathog. 2009 Jul 3;5(7):e1000477. doi: 10.1371/journal.ppat.1000477 (PMC2698986; doi:10.1371/journal.ppat.1000477)
Supplement: Figure S1 — NimbleGen tiling array data for T7 RNA polymerase transcripts from a pool of 1031 Kanamycin resistant specific deletion mutants. (3.18 MB PDF) [file ppat.1000477.s001.pdf]

Santiviago et al., Supplementary Figure 1: NimbleGen tiling array data for T7 RNA polymerase transcripts from a pool of 1031 Kanamycin resistant specific deletion mutants.

The panels show median signal intensities of three oligos representing immediate adjacent regions on the same strand (running median of 3 probes). Peaks indicate the presence of transcript, and therefore the location of a successful deletion mutation slightly upstream in the opposite strand.

Each panel spans data for 10,000 bases of the Typhimurium LT2 genome with approximately 400 oligonucleotide probes in each strand. Red plots the top (positive) strand and blue plots the bottom (negative) strand. Corresponding Typhimurium 14028 gene numbers are given at the top of each panel with red indicating genes with a sense strand orientation, and blue indicating genes with an anti-sense strand orientation. The beginning and end of genes are marked with a triangle. Note that the genome locations are for LT2 as the very similar 14028 genome sequence has not been fully annotated as yet.

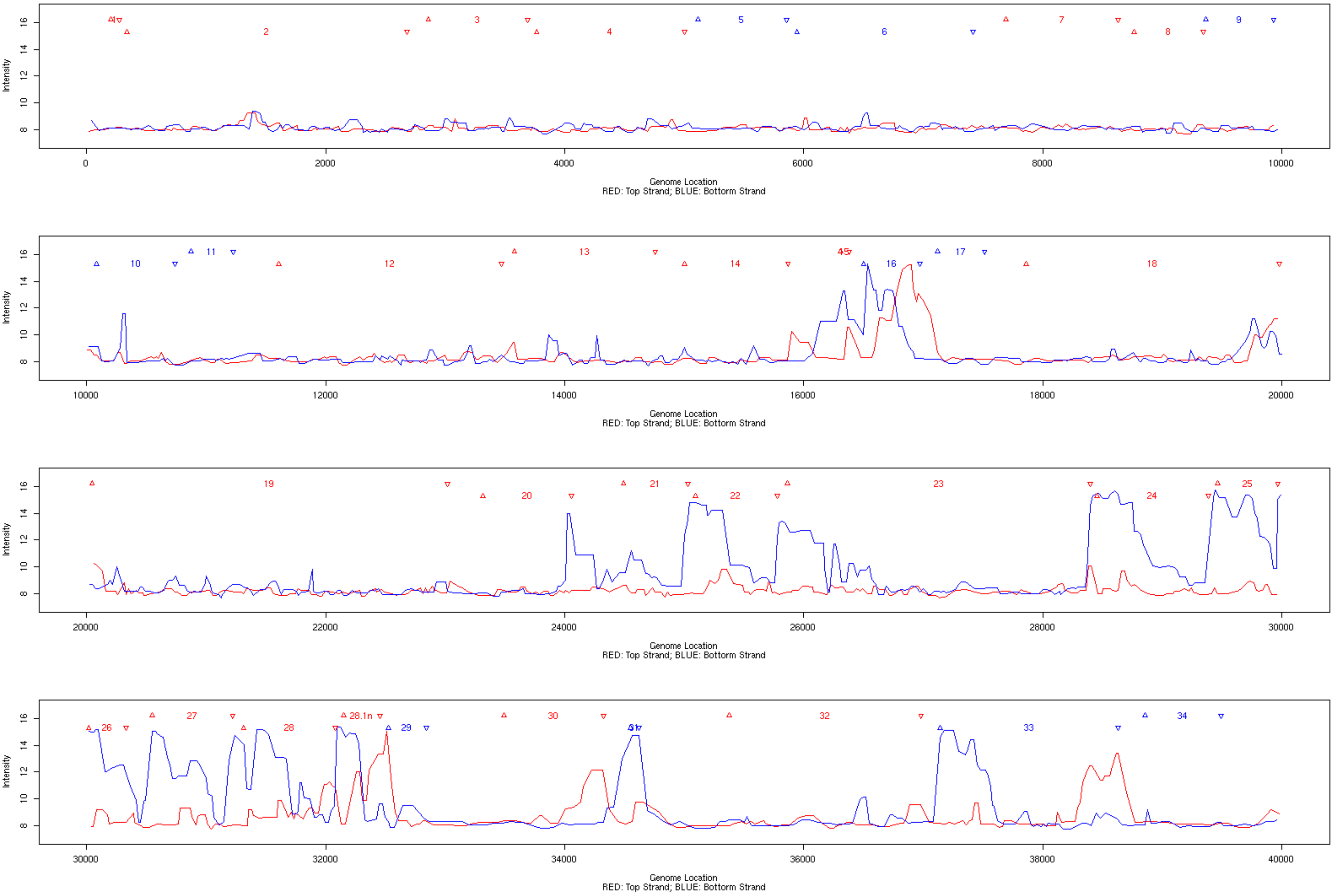

Intensity
Genome Location
RED: Top Strand; BLUE: Bottorm Strand

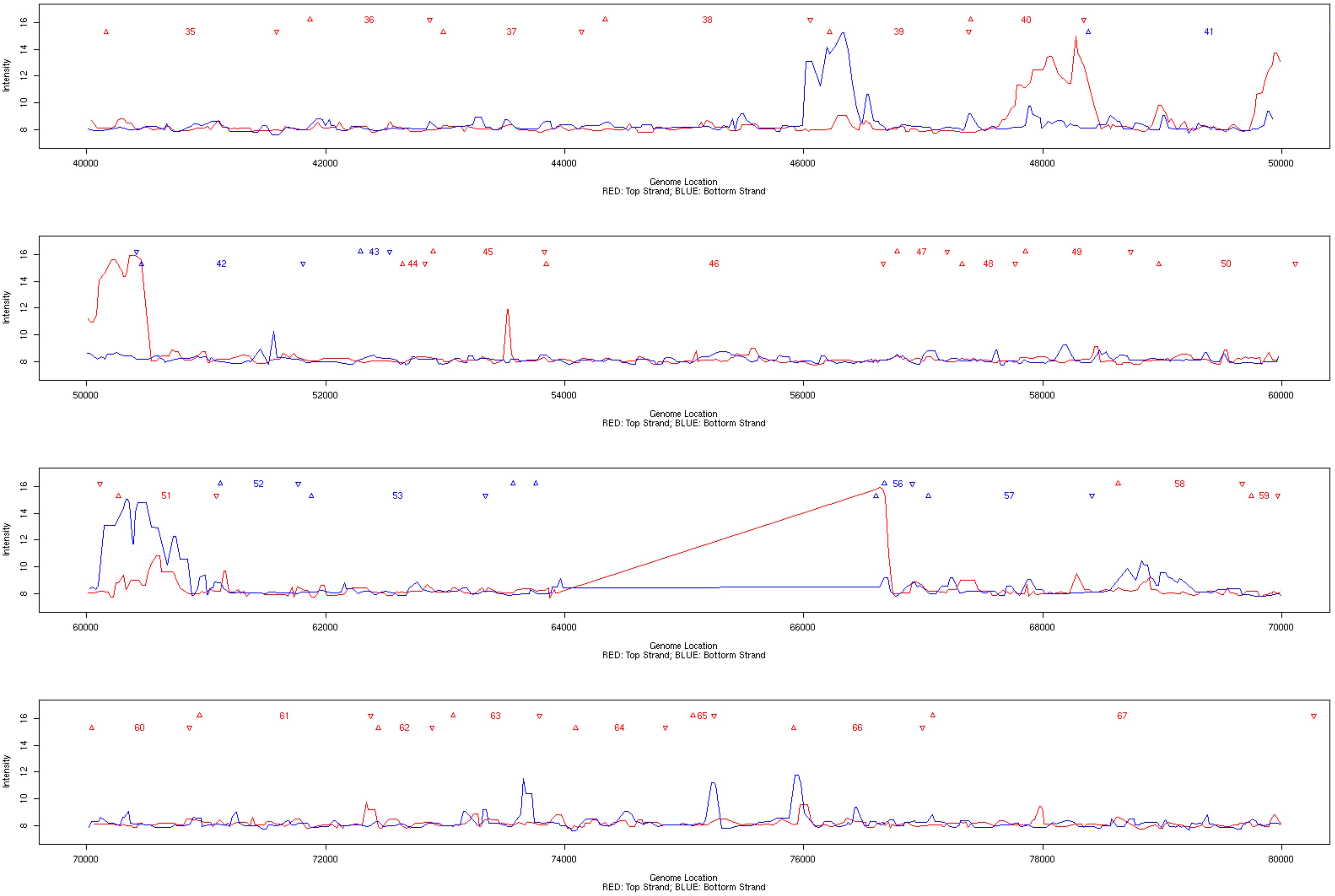

Intensity
Genome Location
RED: Top Strand; BLUE: Bottom Strand
35
36
37
38
39
40
41
42
43
44
45
46
47
48
49
50
51
52
53
56
57
58
59
60
61
62
63
64
65
66
67

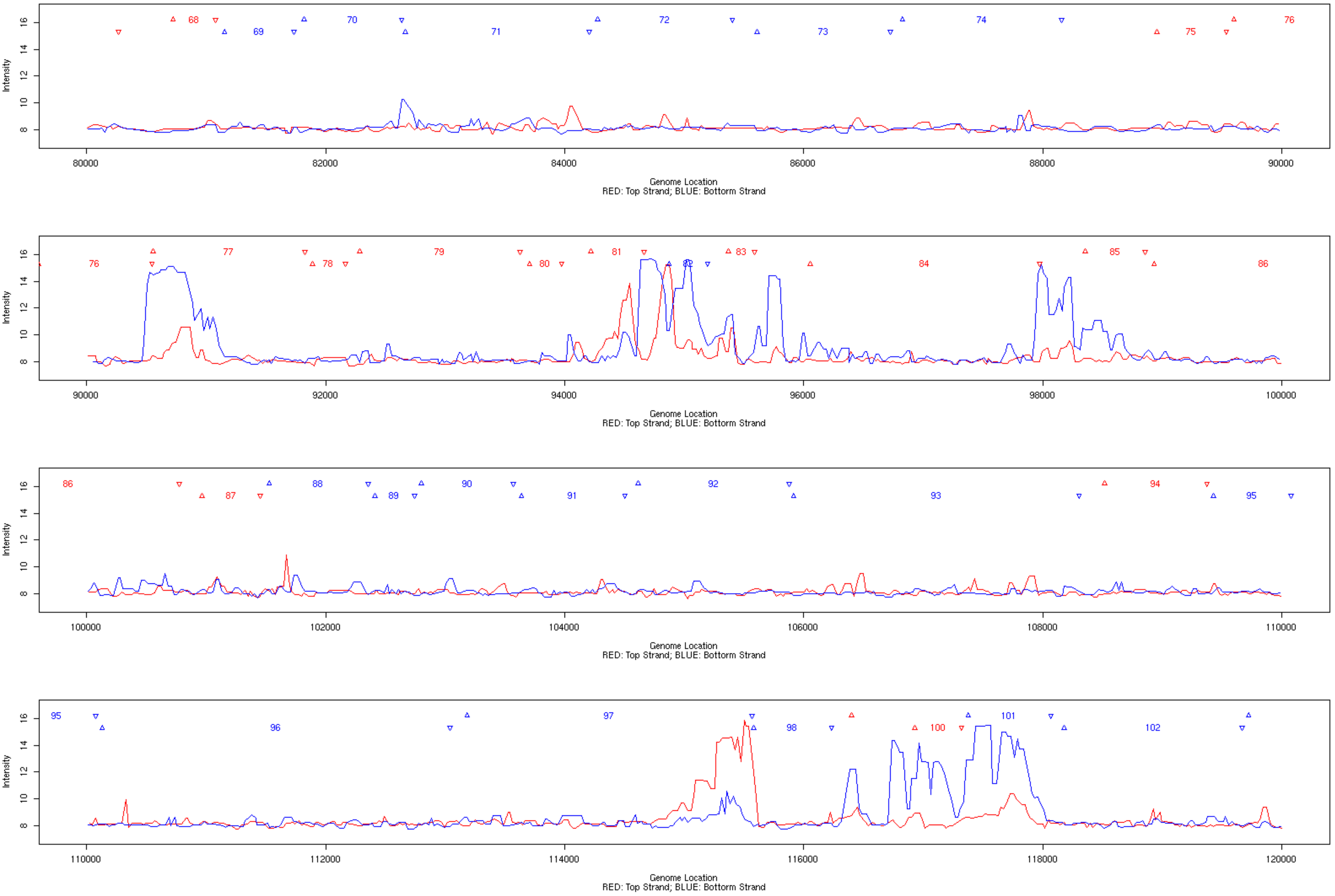

Intensity
Genome Location
RED: Top Strand; BLUE: Bottorm Strand

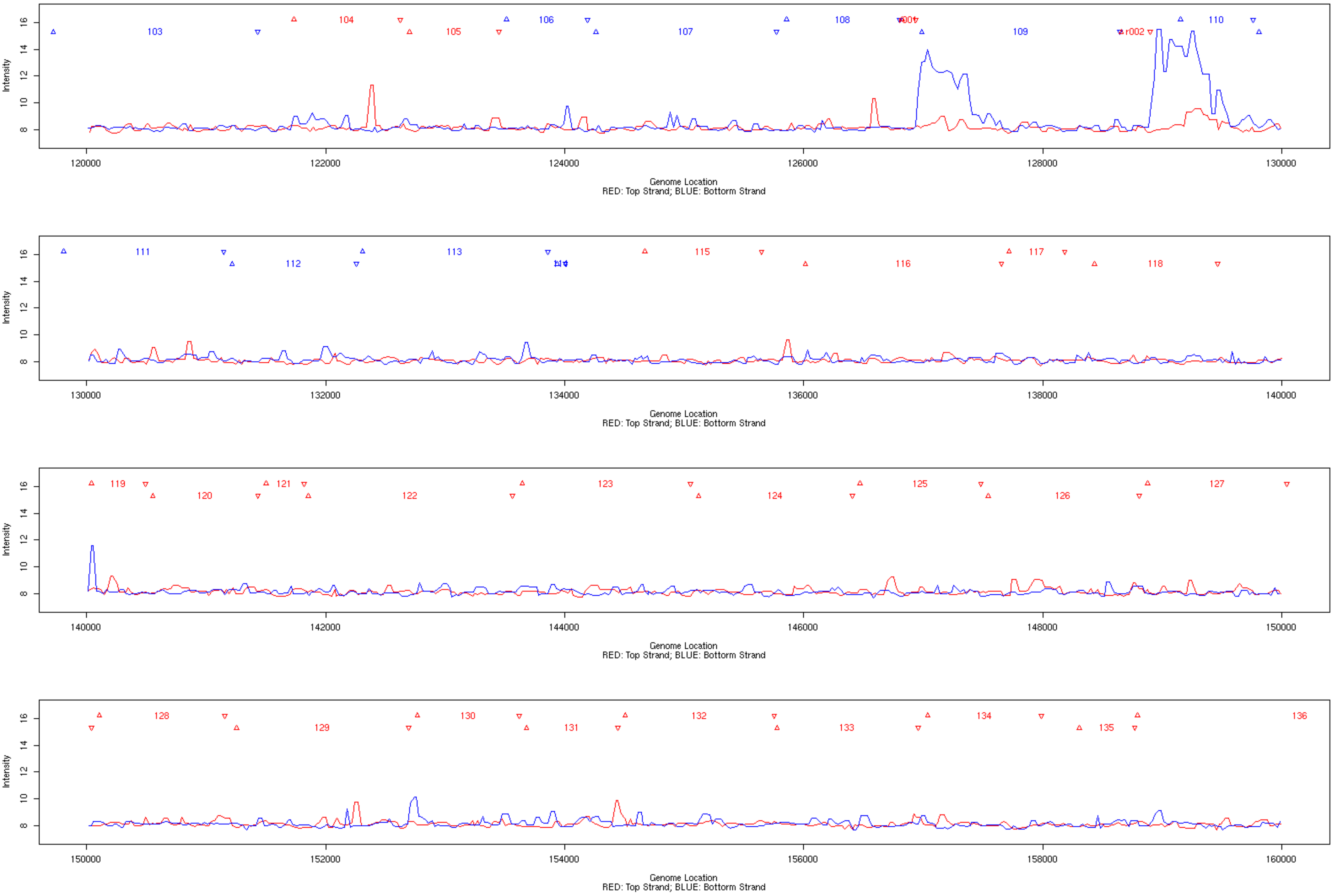

Intensity
Genome Location
RED: Top Strand; BLUE: Bottorm Strand
103
104
105
106
107
108
109
110
r002
111
112
113
115
116
117
118
119
120
121
122
123
124
125
126
127
128
129
130
131
132
133
134
135
136

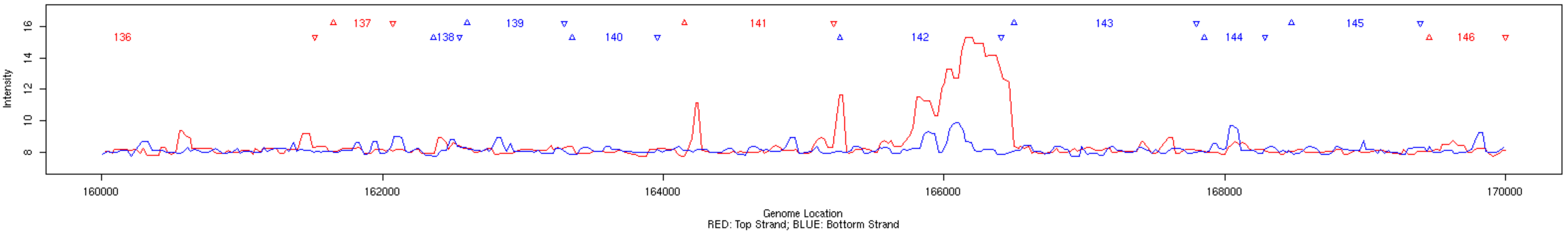
Intensity
136
137
138
139
140
141
142
143
144
145
146
160000
162000
164000
166000
168000
170000
Genome Location
RED: Top Strand; BLUE: Bottorm Strand

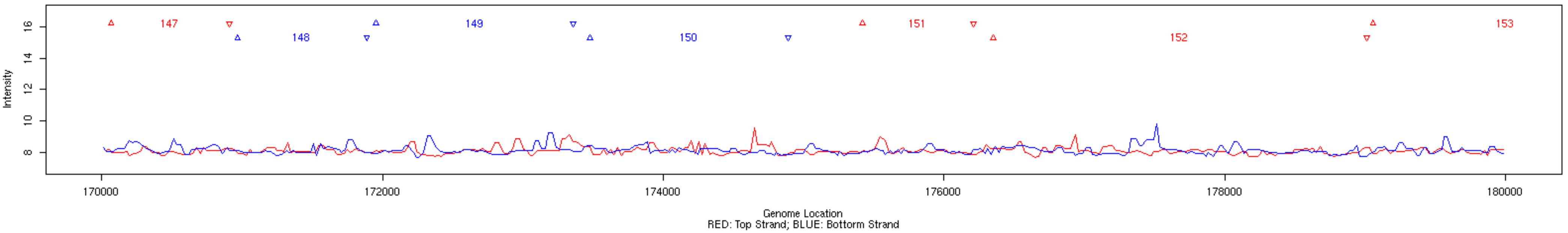
Intensity
147
148
149
150
151
152
153
170000
172000
174000
176000
178000
180000
Genome Location
RED: Top Strand; BLUE: Bottorm Strand

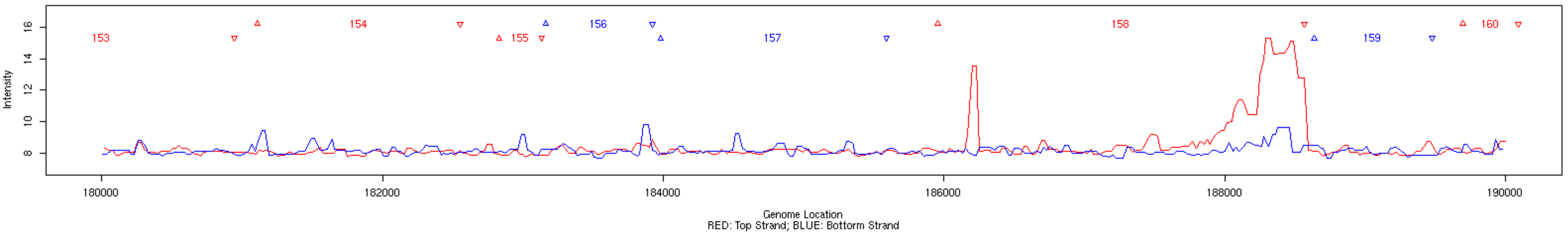
Intensity
153
154
155
156
157
158
159
160
180000
182000
184000
186000
188000
190000
Genome Location
RED: Top Strand; BLUE: Bottorm Strand

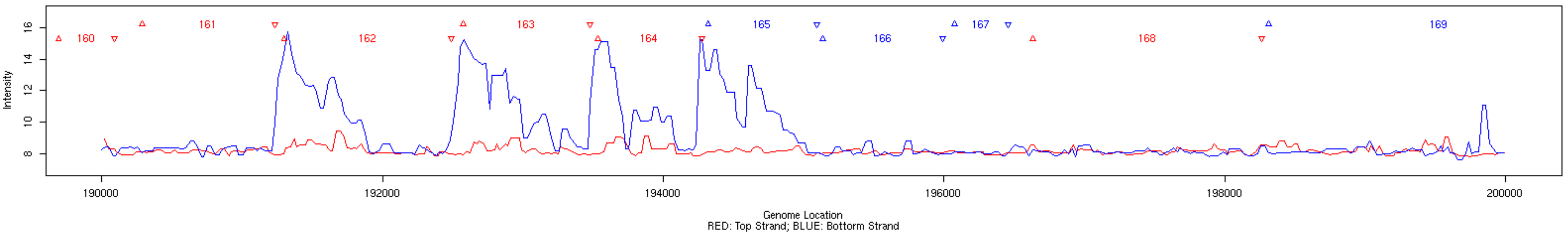
Intensity
160
161
162
163
164
165
166
167
168
169
190000
192000
194000
196000
198000
200000
Genome Location
RED: Top Strand; BLUE: Bottorm Strand

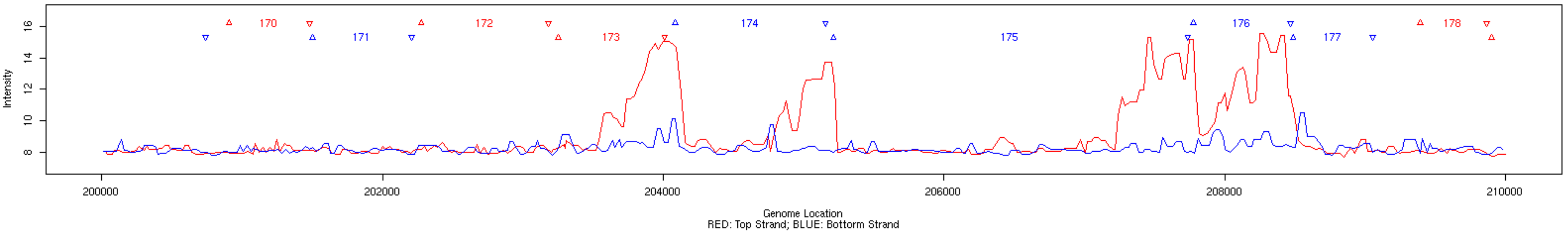
Intensity
170
171
172
173
174
175
176
177
178
Genome Location
RED: Top Strand; BLUE: Bottorm Strand

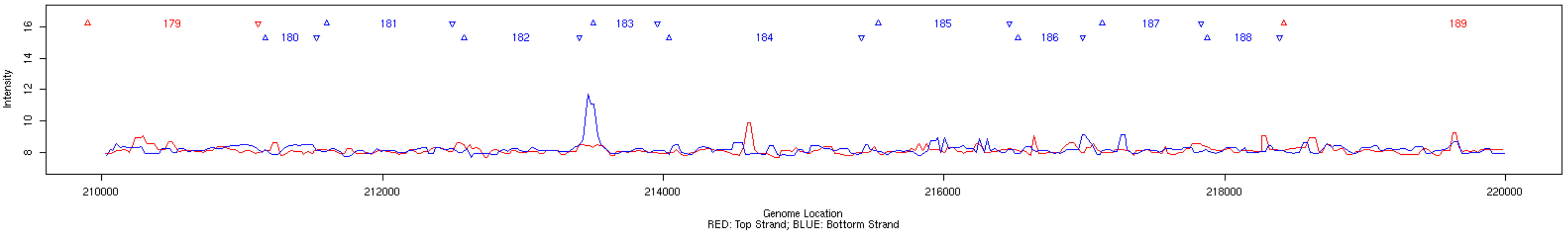
Intensity
179
180
181
182
183
184
185
186
187
188
189
Genome Location
RED: Top Strand; BLUE: Bottorm Strand

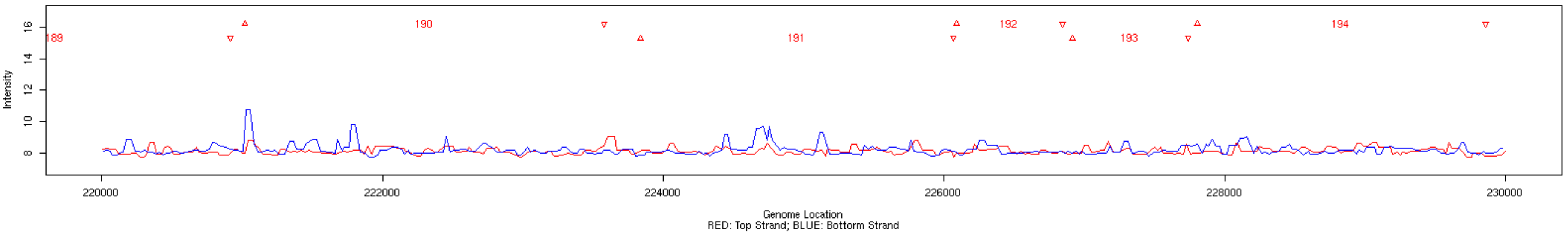
Intensity
189
190
191
192
193
194
Genome Location
RED: Top Strand; BLUE: Bottorm Strand

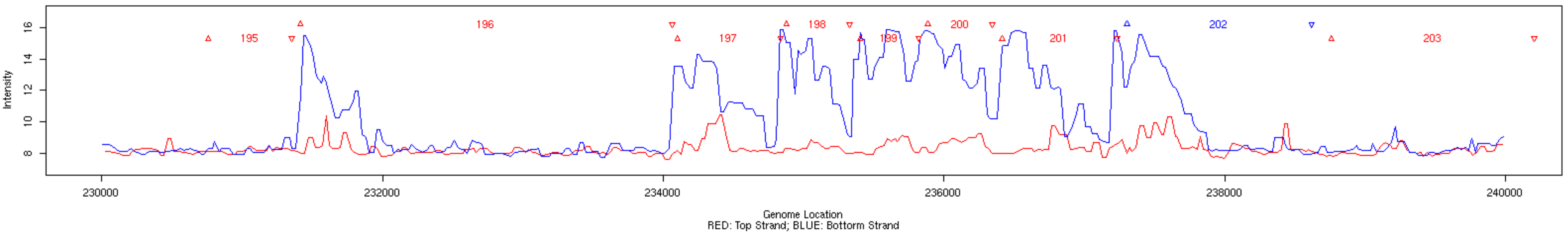
Intensity
195
196
197
198
199
200
201
202
203
Genome Location
RED: Top Strand; BLUE: Bottorm Strand

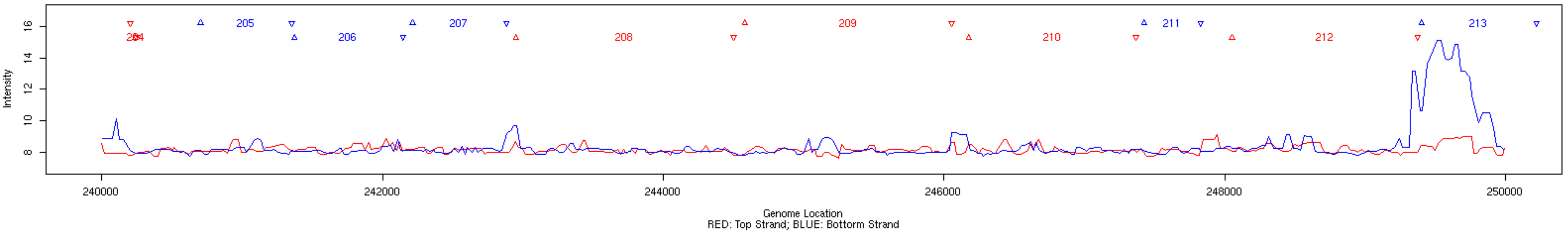
Intensity
Genome Location
RED: Top Strand; BLUE: Bottorm Strand

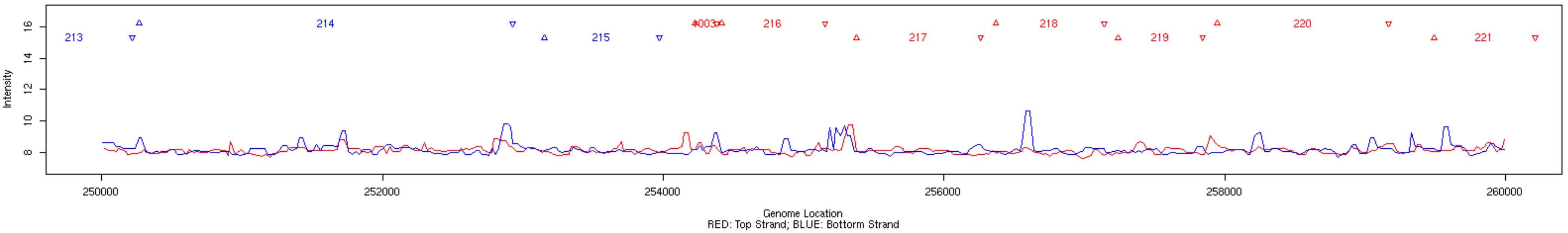
Intensity
Genome Location
RED: Top Strand; BLUE: Bottorm Strand

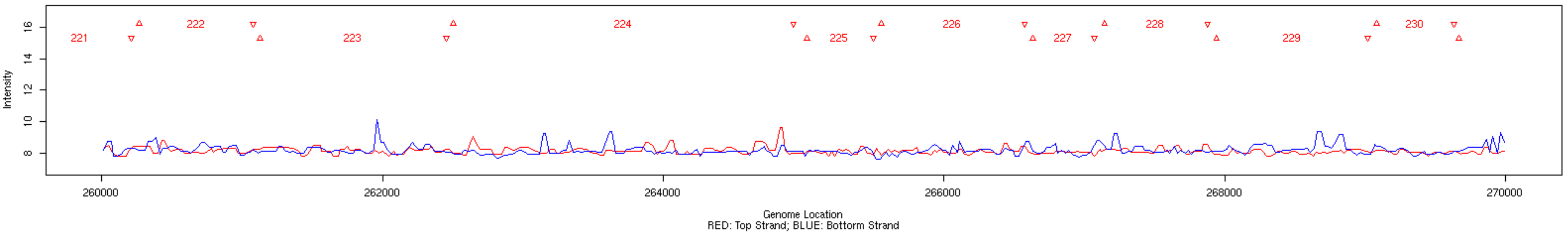
Intensity
Genome Location
RED: Top Strand; BLUE: Bottorm Strand

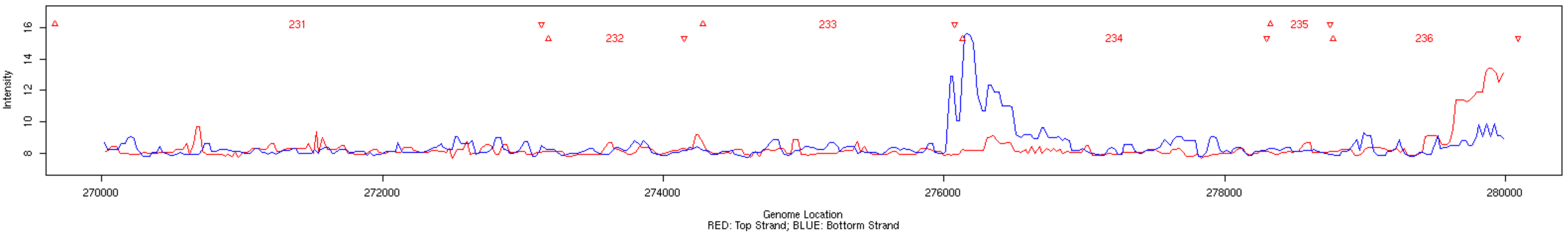
Intensity
Genome Location
RED: Top Strand; BLUE: Bottorm Strand

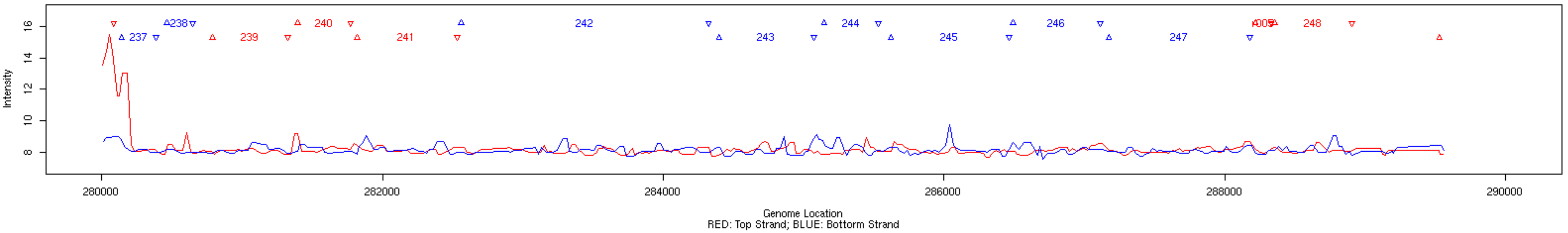
Intensity
237
238
239
240
241
242
243
244
245
246
247
248
280000
282000
284000
286000
288000
290000
Genome Location
RED: Top Strand; BLUE: Bottorm Strand

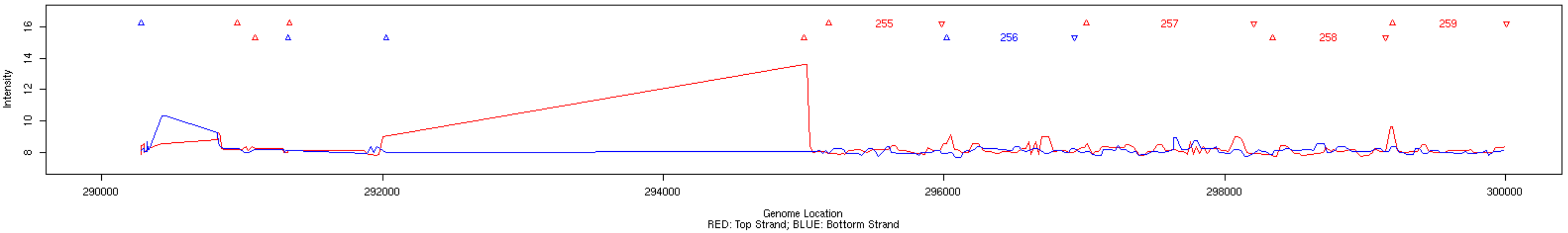
Intensity
255
256
257
258
259
290000
292000
294000
296000
298000
300000
Genome Location
RED: Top Strand; BLUE: Bottorm Strand

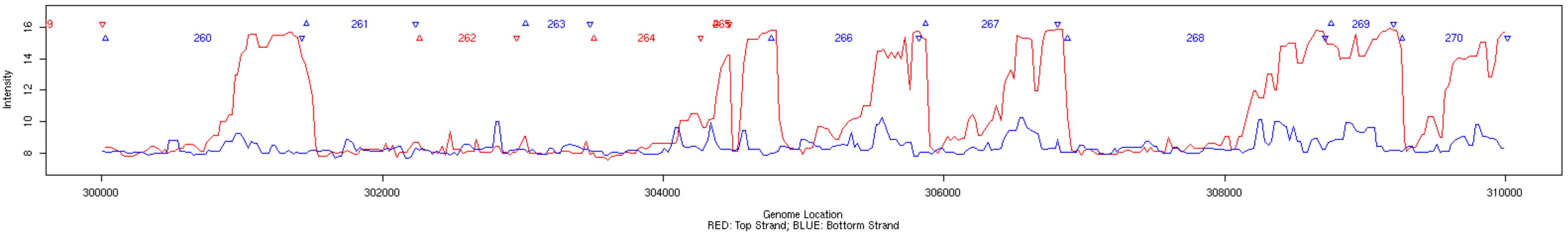
Intensity
260
261
262
263
264
266
267
268
269
270
300000
302000
304000
306000
308000
310000
Genome Location
RED: Top Strand; BLUE: Bottorm Strand

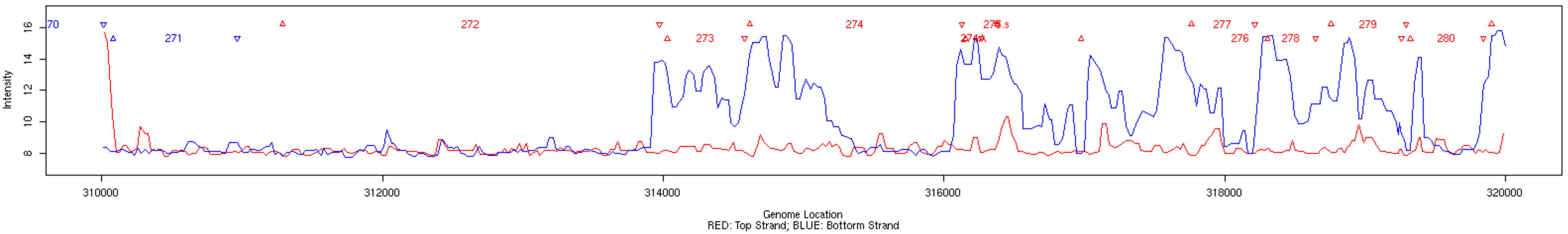
Intensity
271
272
273
274
276
277
278
279
280
310000
312000
314000
316000
318000
320000
Genome Location
RED: Top Strand; BLUE: Bottorm Strand

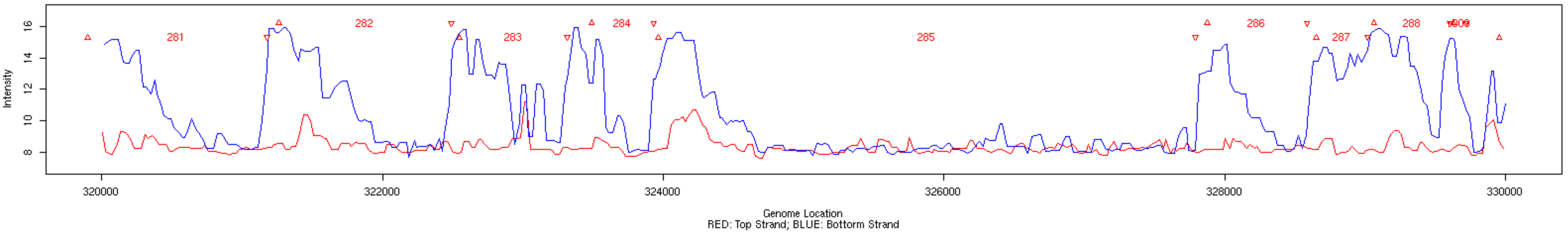
Intensity
281
282
283
284
285
286
287
288
320000
322000
324000
326000
328000
330000
Genome Location
RED: Top Strand; BLUE: Bottom Strand

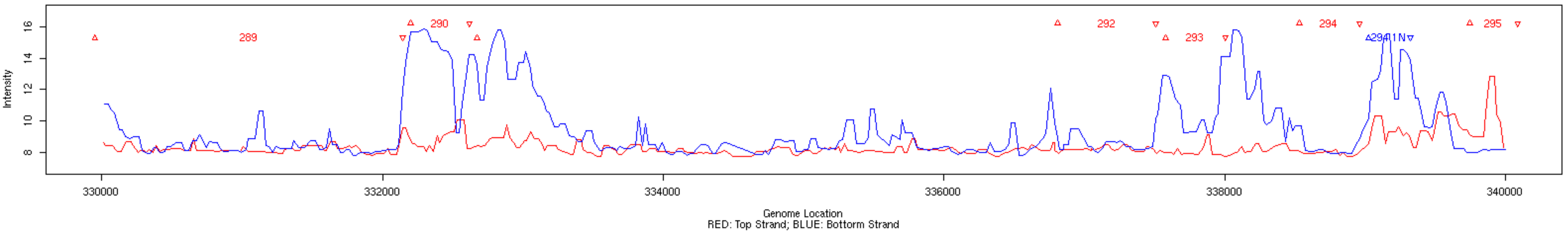
Intensity
289
290
292
293
294
295
330000
332000
334000
336000
338000
340000
Genome Location
RED: Top Strand; BLUE: Bottom Strand

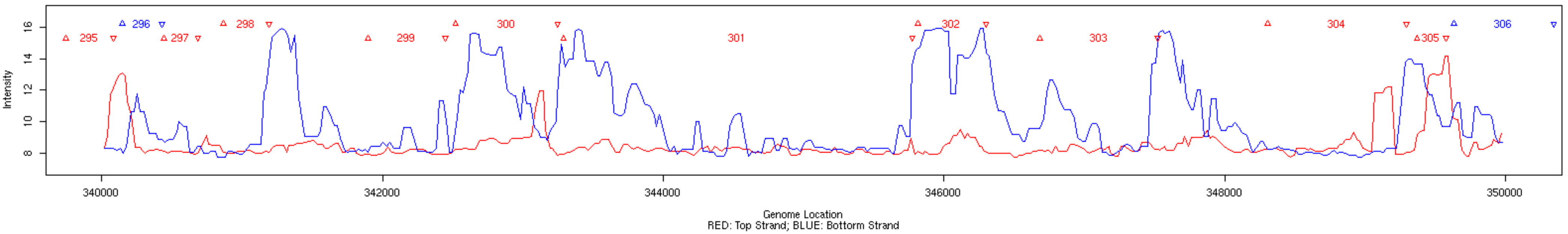
Intensity
295
296
297
298
299
300
301
302
303
304
305
306
340000
342000
344000
346000
348000
350000
Genome Location
RED: Top Strand; BLUE: Bottom Strand

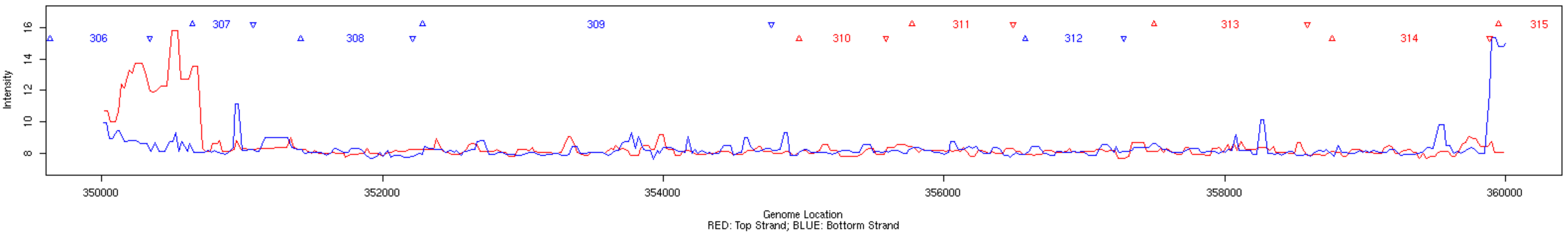
Intensity
306
307
308
309
310
311
312
313
314
315
350000
352000
354000
356000
358000
360000
Genome Location
RED: Top Strand; BLUE: Bottom Strand

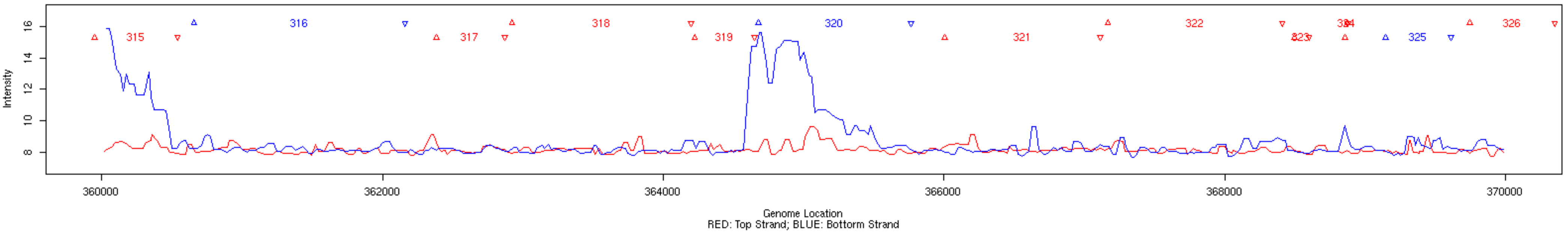
Intensity
Genome Location
RED: Top Strand; BLUE: Bottorm Strand

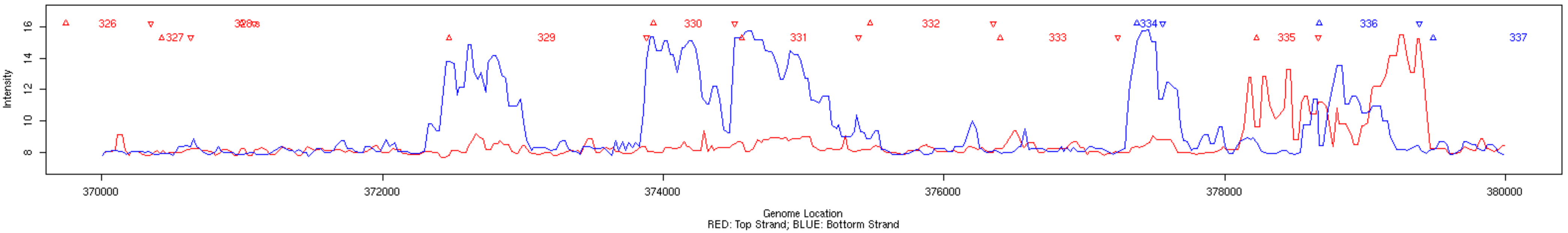
Intensity
Genome Location
RED: Top Strand; BLUE: Bottorm Strand

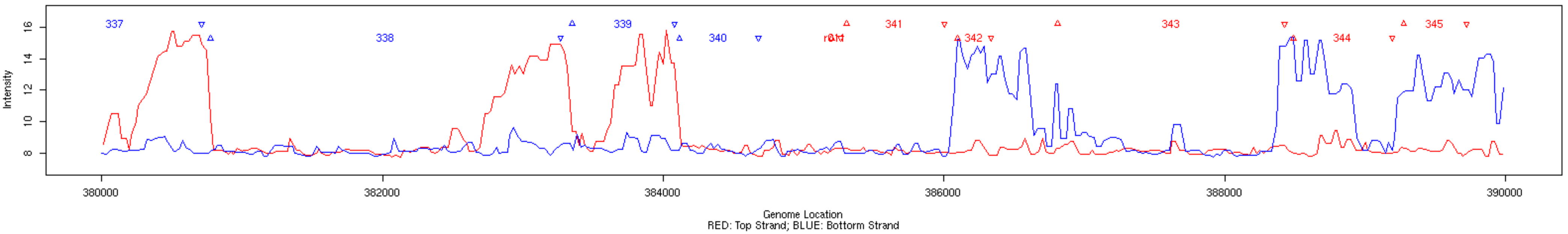
Intensity
Genome Location
RED: Top Strand; BLUE: Bottorm Strand

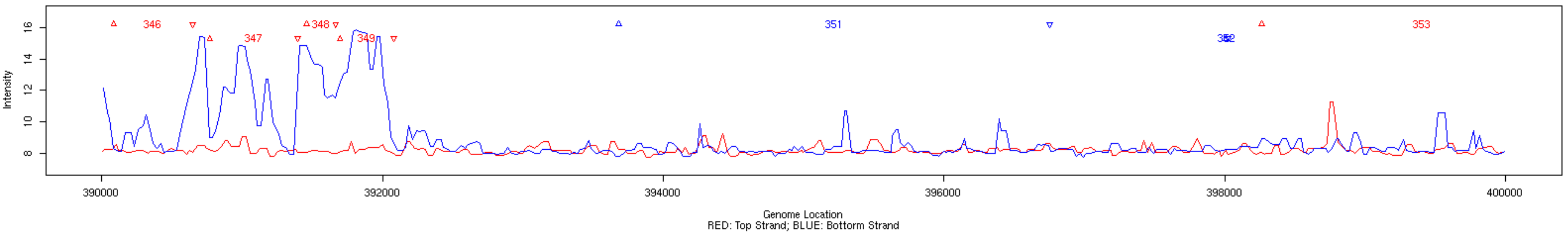
Intensity
Genome Location
RED: Top Strand; BLUE: Bottorm Strand

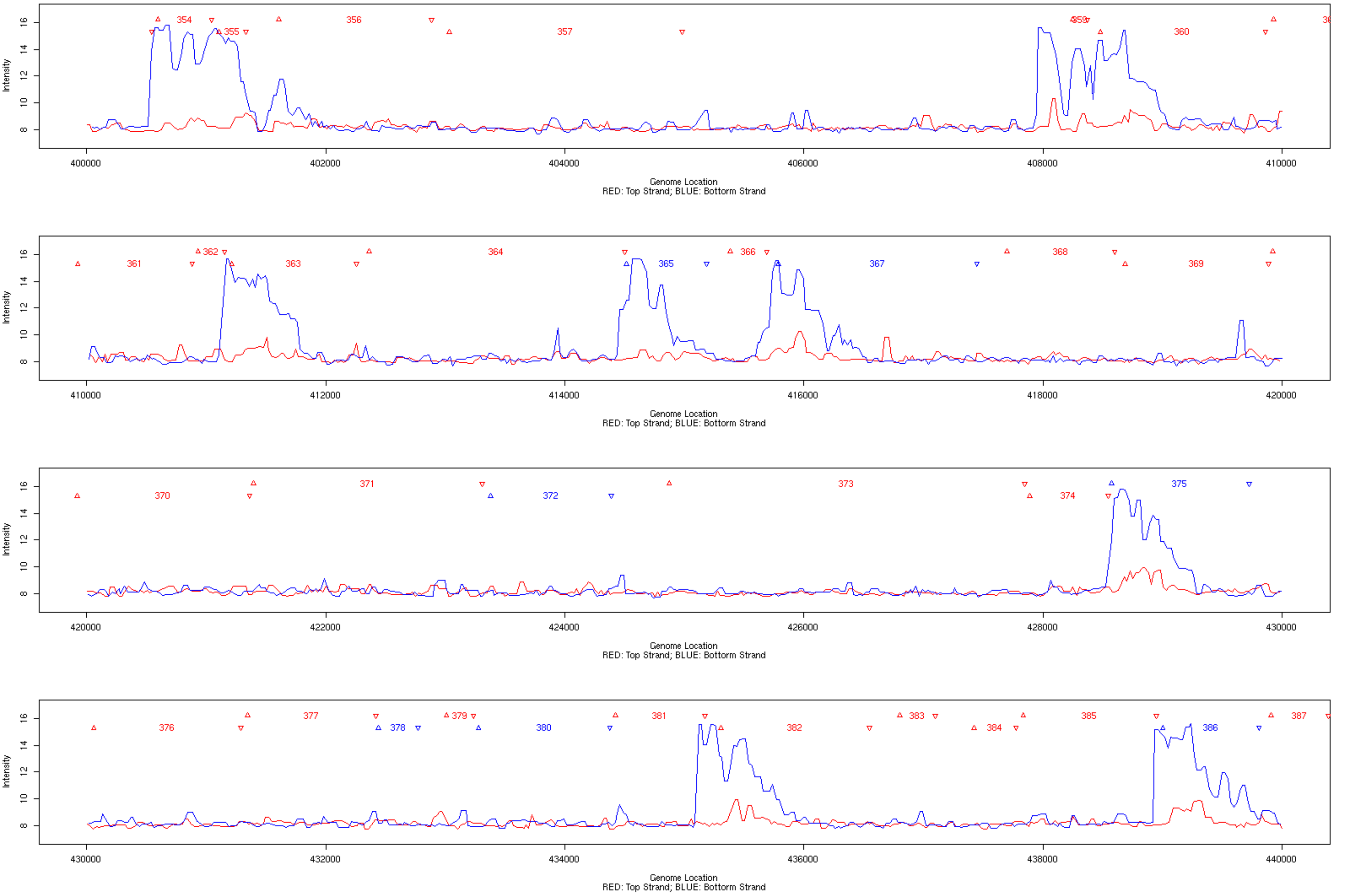
Intensity
Genome Location
RED: Top Strand; BLUE: Bottorm Strand
354
355
356
357
359
360
361
362
363
364
365
366
367
368
369
370
371
372
373
374
375
376
377
378
379
380
381
382
383
384
385
386
387

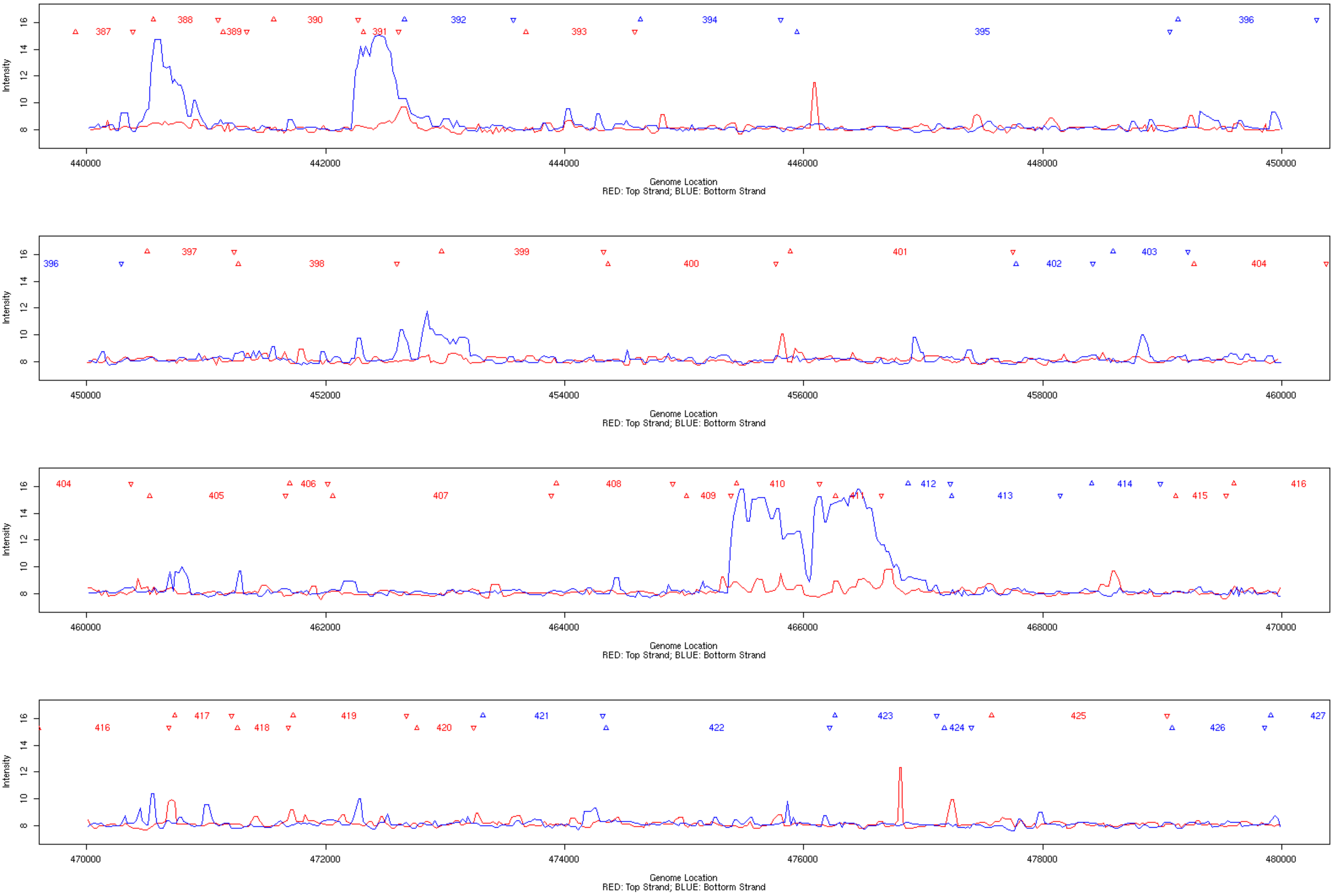
Intensity
Genome Location
RED: Top Strand; BLUE: Bottorm Strand
387 388 389 390 391 392 393 394 395 396
396 397 398 399 400 401 402 403 404
404 405 406 407 408 409 410 411 412 413 414 415 416
416 417 418 419 420 421 422 423 424 425 426 427

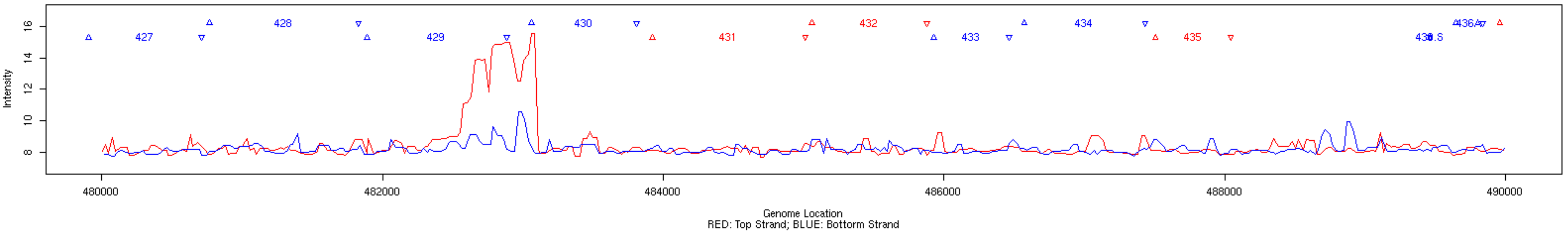

Intensity
427
428
429
430
431
432
433
434
435
480000
482000
484000
486000
488000
490000
Genome Location
RED: Top Strand; BLUE: Bottorm Strand

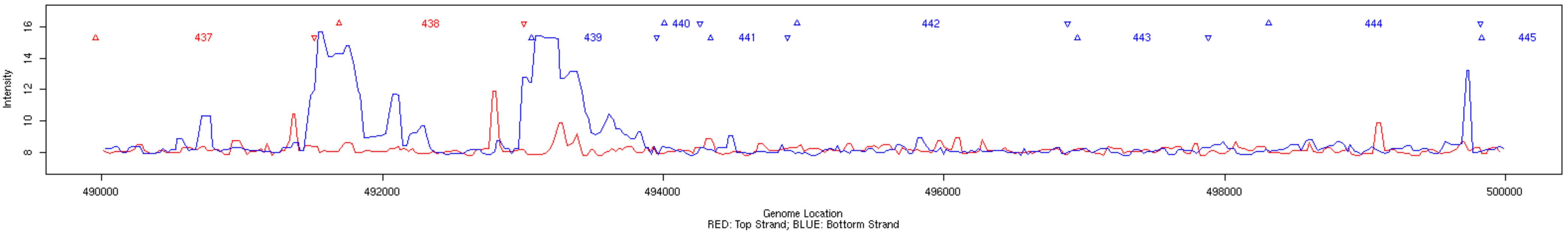

Intensity
437
438
439
440
441
442
443
444
445
490000
492000
494000
496000
498000
500000
Genome Location
RED: Top Strand; BLUE: Bottorm Strand

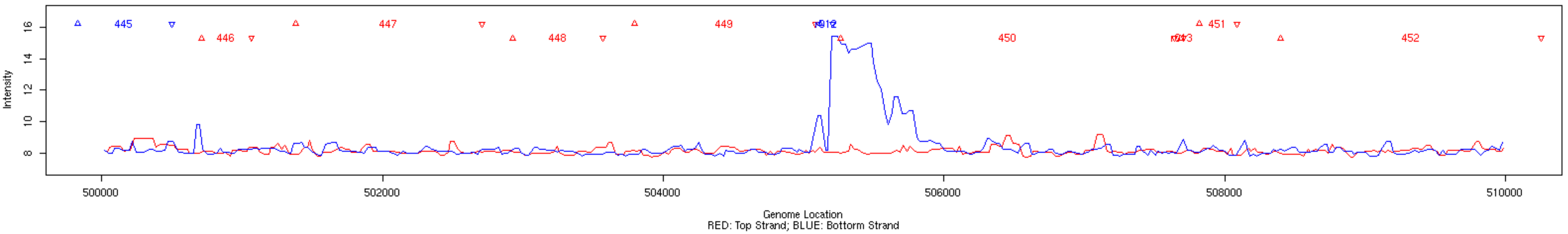

Intensity
445
446
447
448
449
450
451
452
500000
502000
504000
506000
508000
510000
Genome Location
RED: Top Strand; BLUE: Bottorm Strand

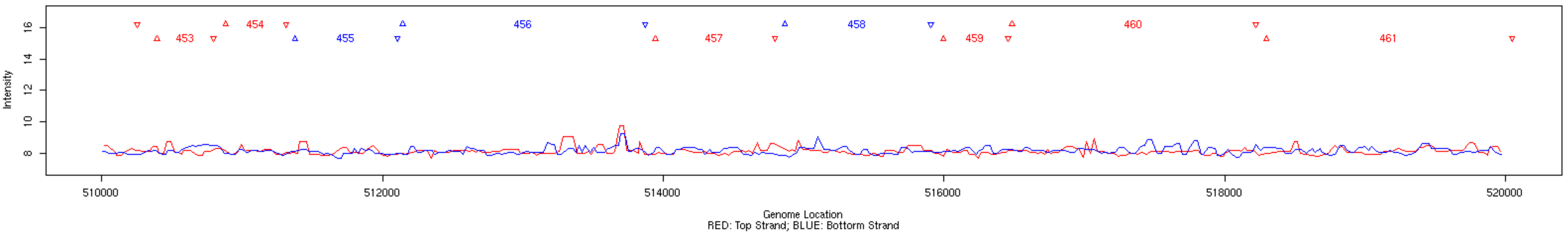

Intensity
453
454
455
456
457
458
459
460
461
510000
512000
514000
516000
518000
520000
Genome Location
RED: Top Strand; BLUE: Bottorm Strand

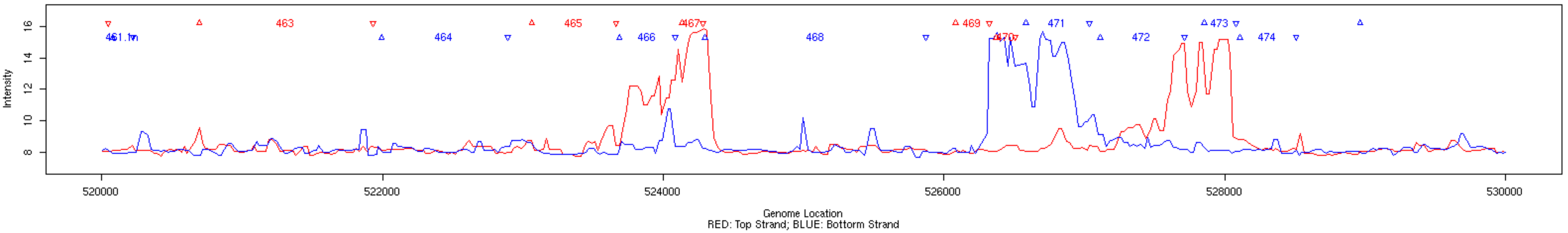
Intensity
Genome Location
RED: Top Strand; BLUE: Bottorm Strand
463
465
467
469
471
472
473
474
464
466
468
520000
522000
524000
526000
528000
530000

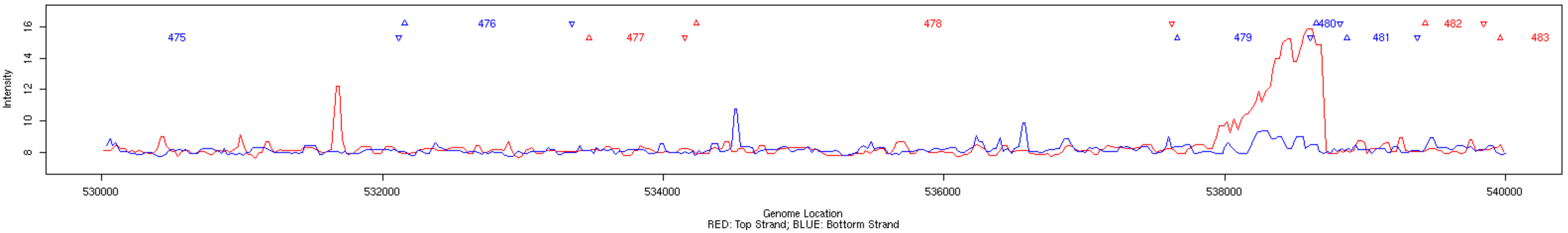
Intensity
Genome Location
RED: Top Strand; BLUE: Bottorm Strand
475
476
477
478
479
480
481
482
483
530000
532000
534000
536000
538000
540000

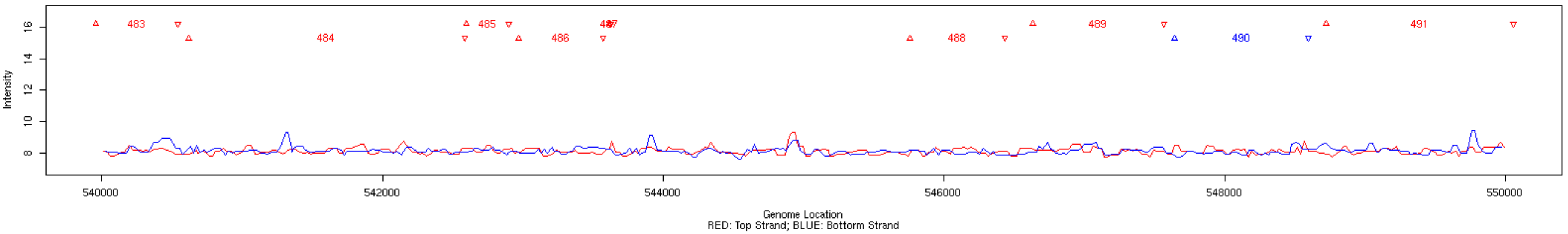
Intensity
Genome Location
RED: Top Strand; BLUE: Bottorm Strand
483
484
485
486
488
489
490
491
540000
542000
544000
546000
548000
550000

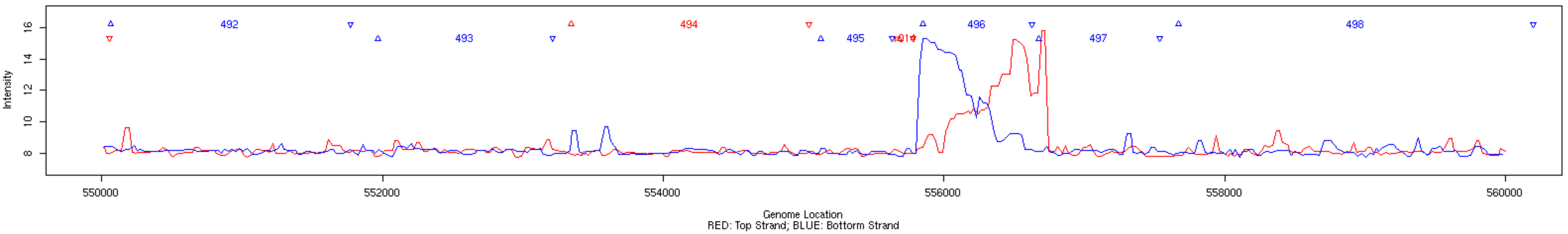
Intensity
Genome Location
RED: Top Strand; BLUE: Bottorm Strand
492
493
494
495
496
497
498
550000
552000
554000
556000
558000
560000

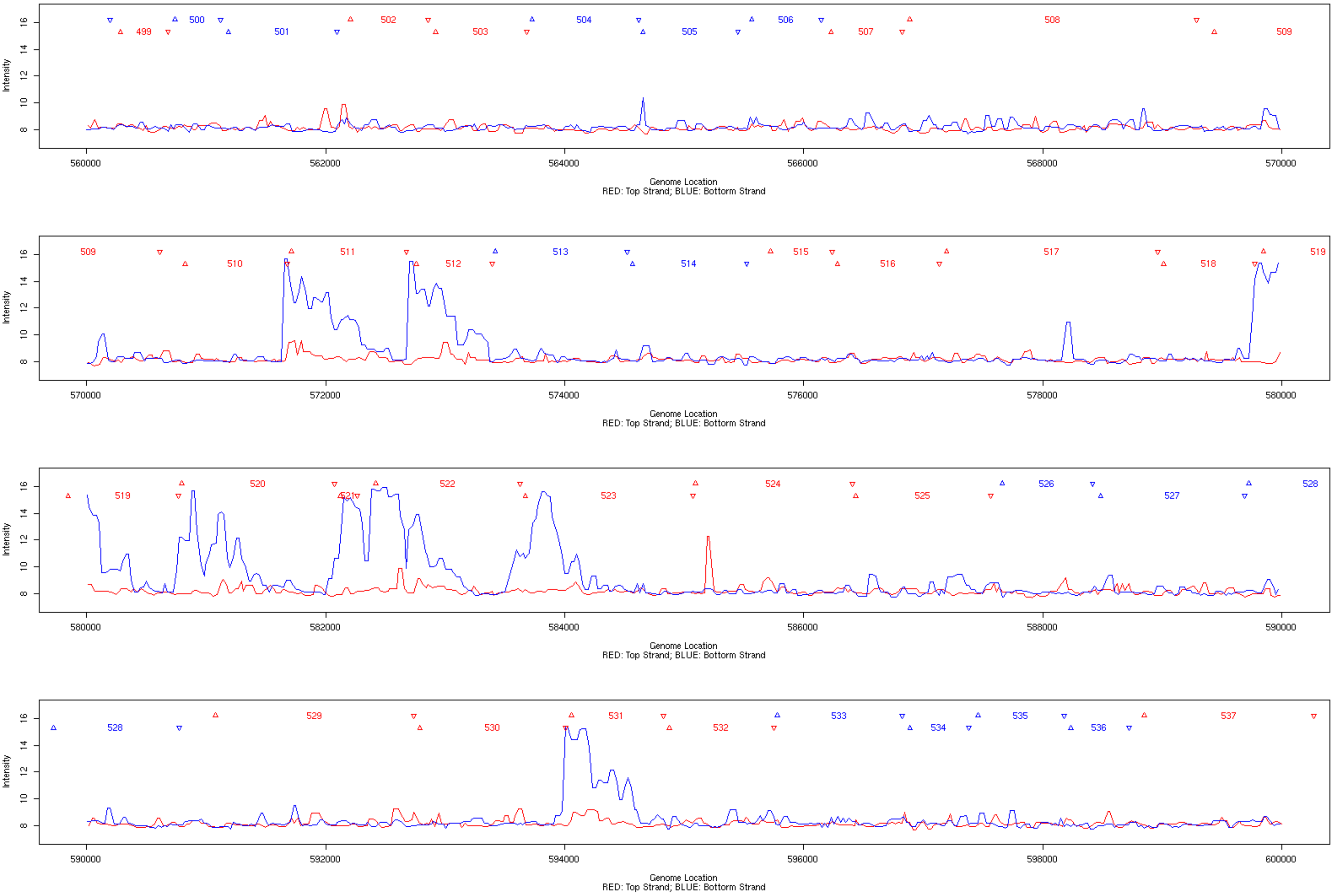
Intensity
Genome Location
RED: Top Strand; BLUE: Bottorm Strand
560000
562000
564000
566000
568000
570000
499
500
501
502
503
504
505
506
507
508
509
572000
574000
576000
578000
580000
510
511
512
513
514
515
516
517
518
519
582000
584000
586000
588000
590000
520
521
522
523
524
525
526
527
528
592000
594000
596000
598000
600000
529
530
531
532
533
534
535
536
537

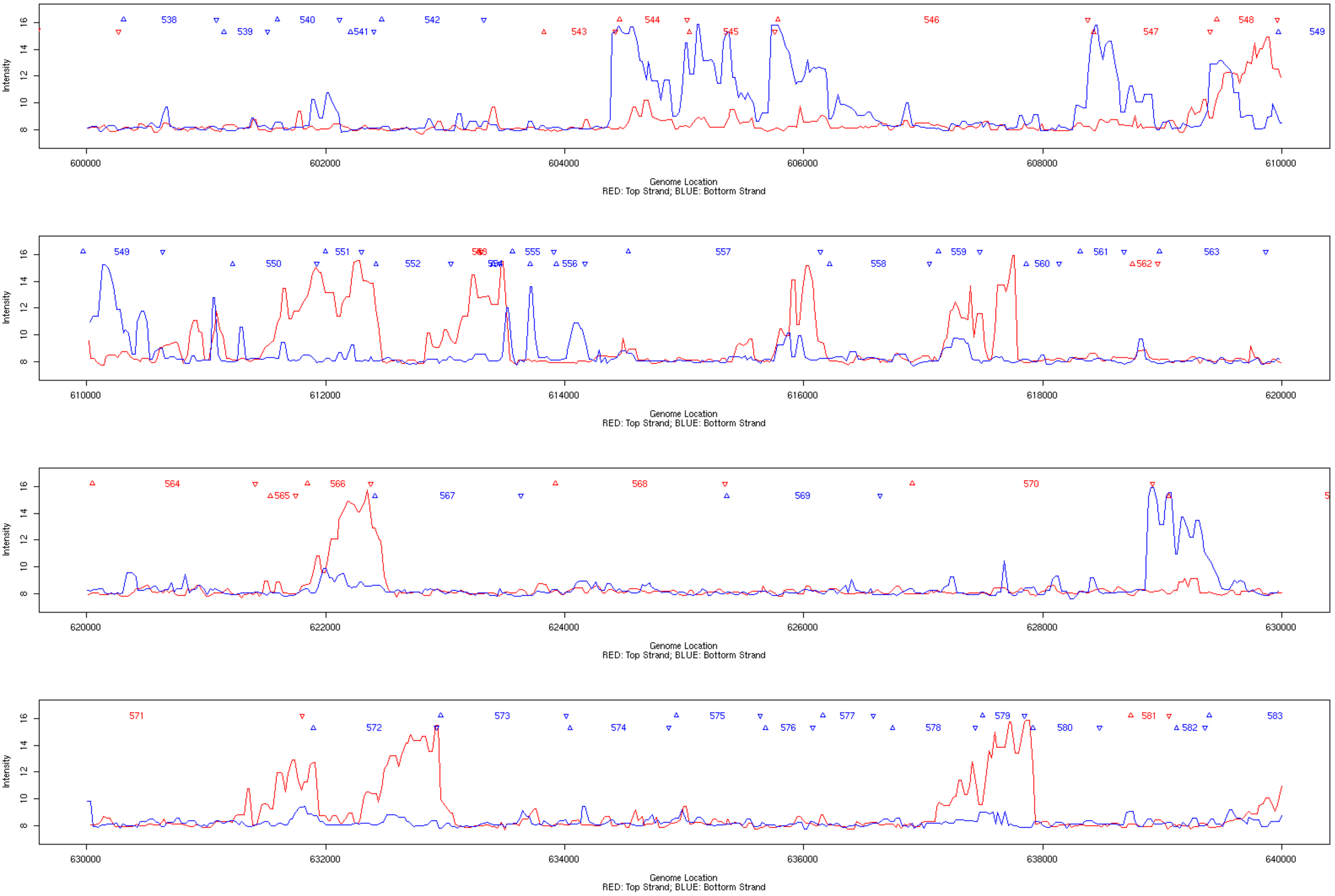
Intensity
Genome Location
RED: Top Strand; BLUE: Bottom Strand
538
539
540
541
542
543
544
545
546
547
548
549
600000
602000
604000
606000
608000
610000
550
551
552
555
556
557
558
559
560
561
562
563
612000
614000
616000
618000
620000
564
565
566
567
568
569
570
622000
624000
626000
628000
630000
571
572
573
574
575
576
577
578
579
580
581
582
583
632000
634000
636000
638000
640000
8
10
12
14
16

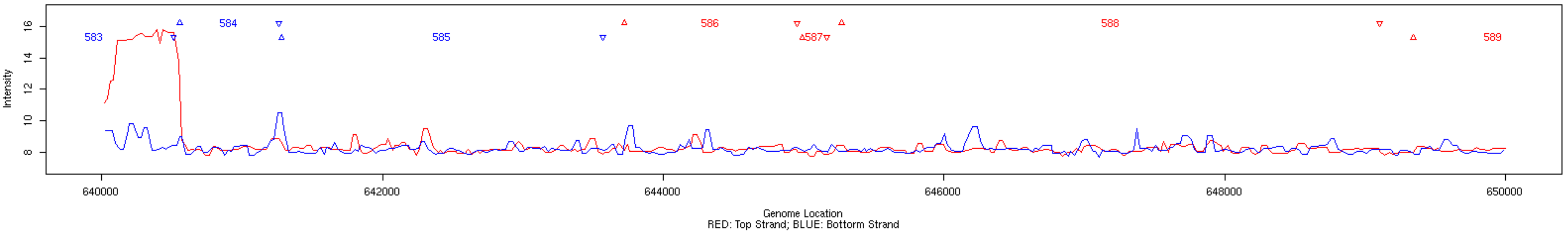
583
584
585
586
587
588
589
Intensity
Genome Location
RED: Top Strand; BLUE: Bottom Strand

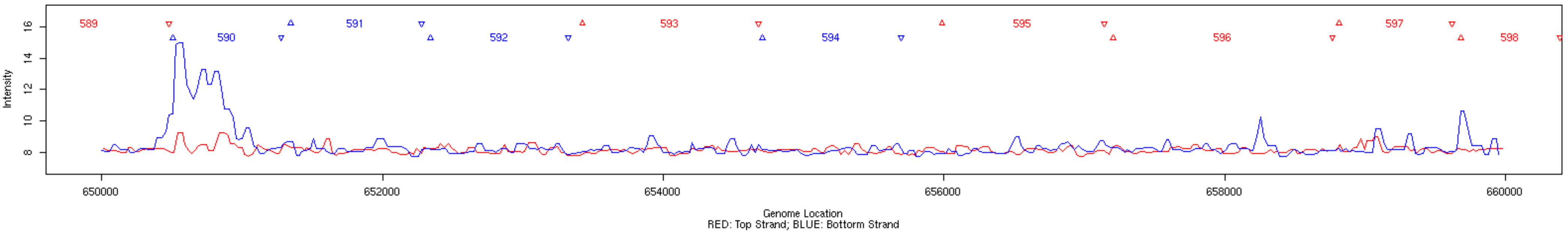
589
590
591
592
593
594
595
596
597
598
Intensity
Genome Location
RED: Top Strand; BLUE: Bottom Strand

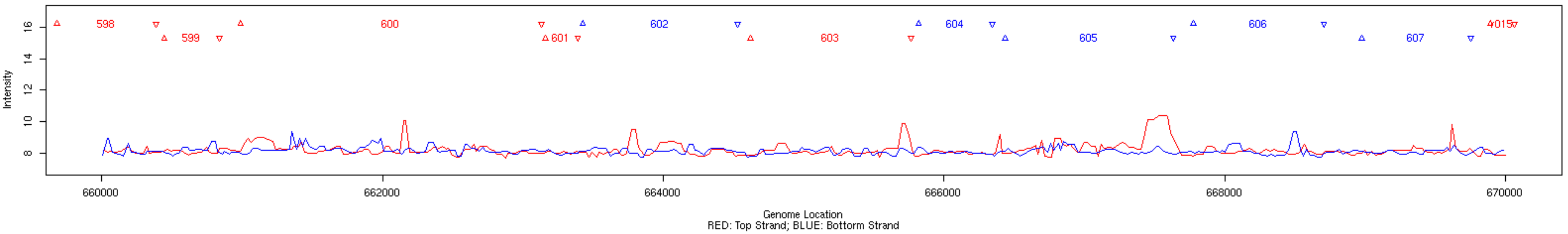
598
599
600
601
602
603
604
605
606
607
4015
Intensity
Genome Location
RED: Top Strand; BLUE: Bottom Strand

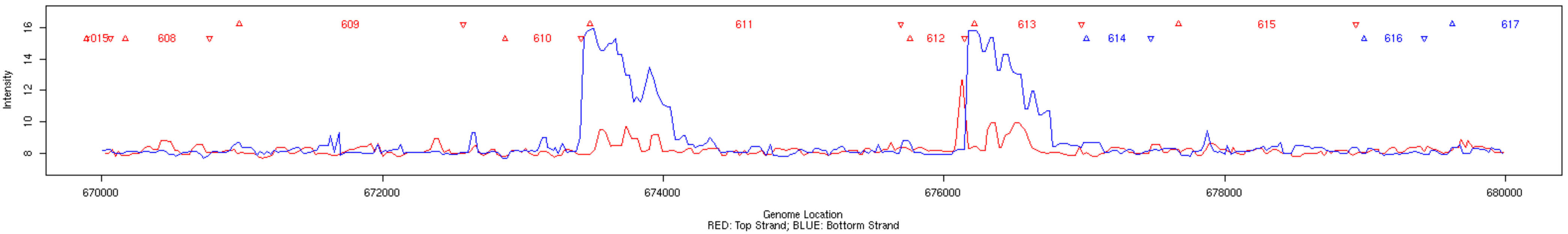
4015
608
609
610
611
612
613
614
615
616
617
Intensity
Genome Location
RED: Top Strand; BLUE: Bottom Strand

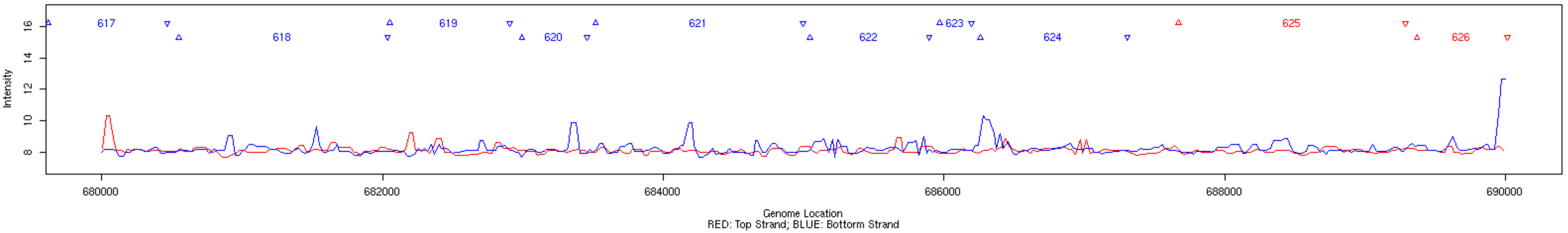
617
618
619
620
621
622
623
624
625
626
Intensity
680000
682000
684000
686000
688000
690000
Genome Location
RED: Top Strand; BLUE: Bottorm Strand

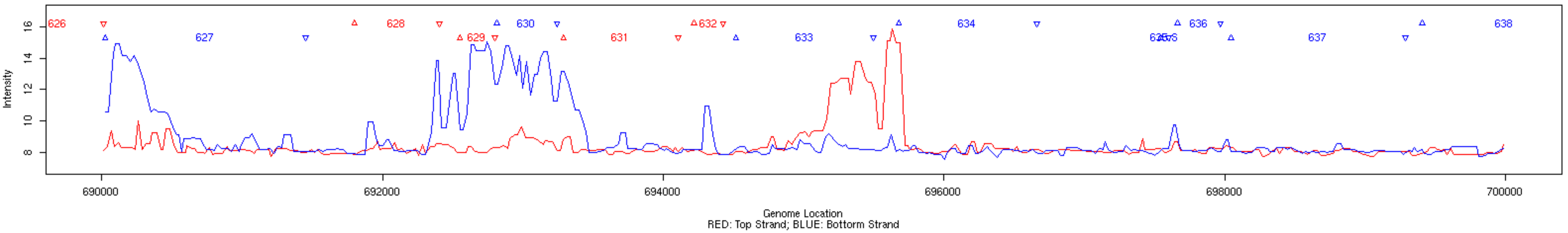
626
627
628
629
630
631
632
633
634
636
637
638
Intensity
690000
692000
694000
696000
698000
700000
Genome Location
RED: Top Strand; BLUE: Bottorm Strand

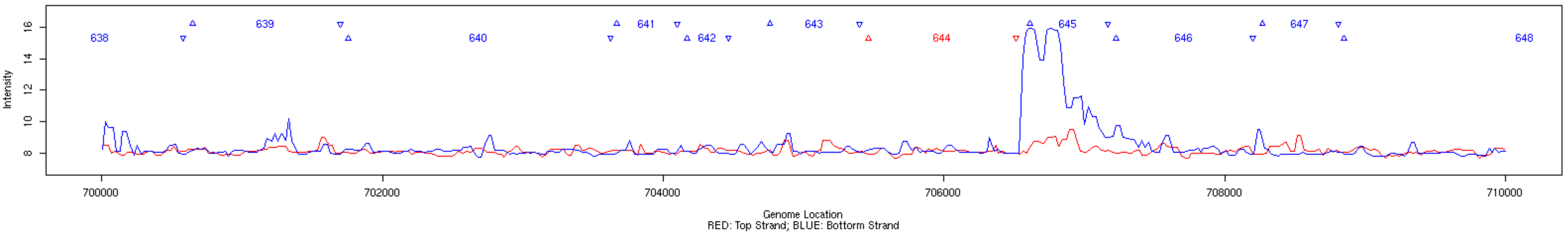
638
639
640
641
642
643
644
645
646
647
648
Intensity
700000
702000
704000
706000
708000
710000
Genome Location
RED: Top Strand; BLUE: Bottorm Strand

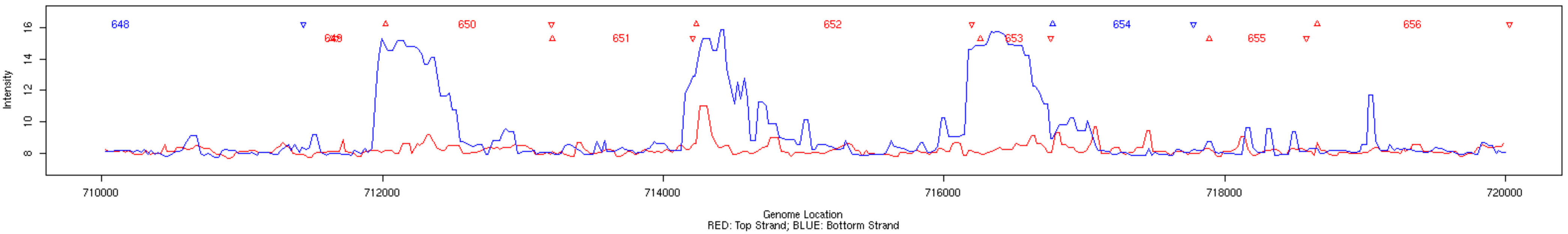
648
650
651
652
653
654
655
656
Intensity
710000
712000
714000
716000
718000
720000
Genome Location
RED: Top Strand; BLUE: Bottorm Strand

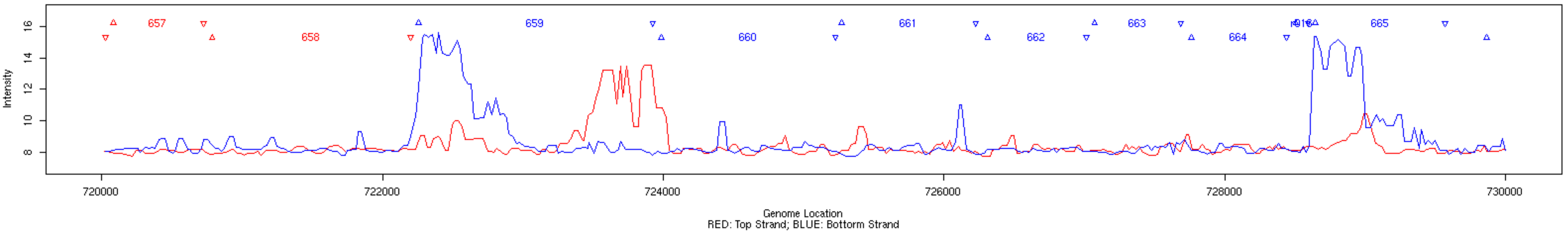
Intensity
657
658
659
660
661
662
663
664
665
720000
722000
724000
726000
728000
730000
Genome Location
RED: Top Strand; BLUE: Bottorm Strand

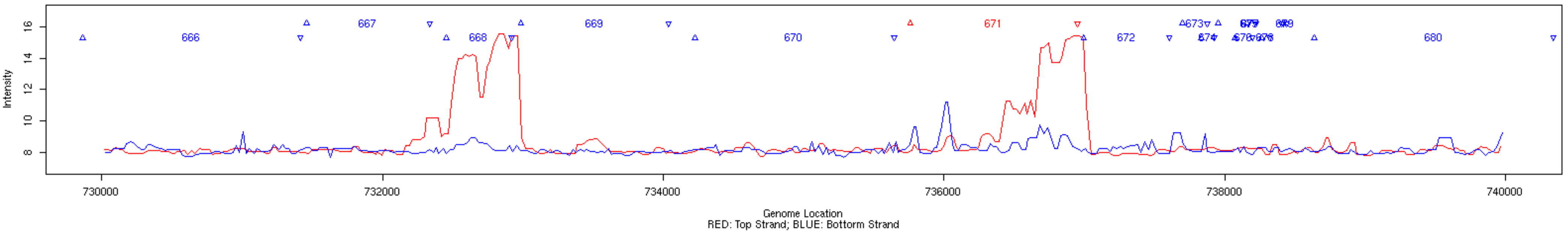
Intensity
666
667
668
669
670
671
672
680
730000
732000
734000
736000
738000
740000
Genome Location
RED: Top Strand; BLUE: Bottorm Strand

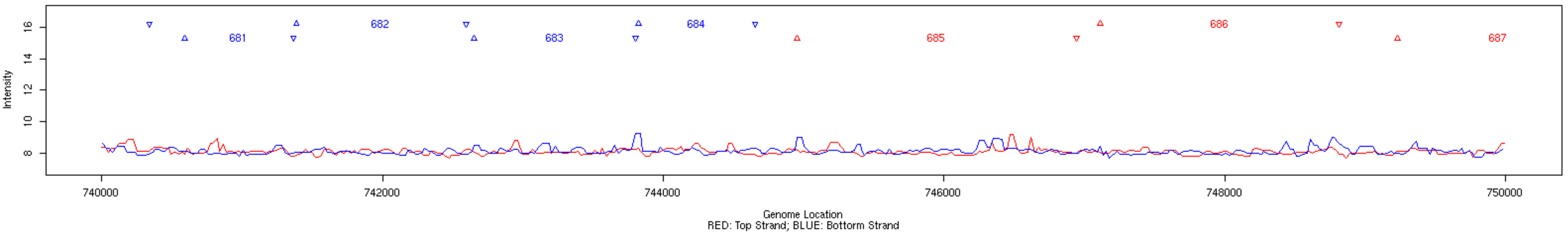
Intensity
681
682
683
684
685
686
687
740000
742000
744000
746000
748000
750000
Genome Location
RED: Top Strand; BLUE: Bottorm Strand

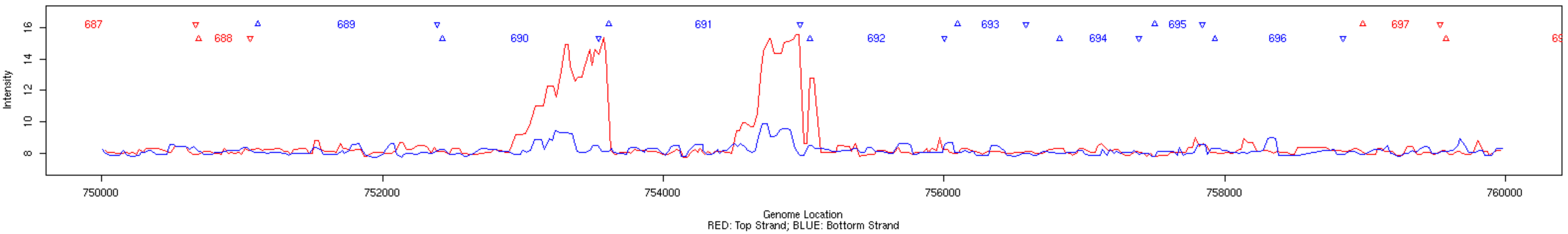
Intensity
687
688
689
690
691
692
693
694
695
696
697
750000
752000
754000
756000
758000
760000
Genome Location
RED: Top Strand; BLUE: Bottorm Strand

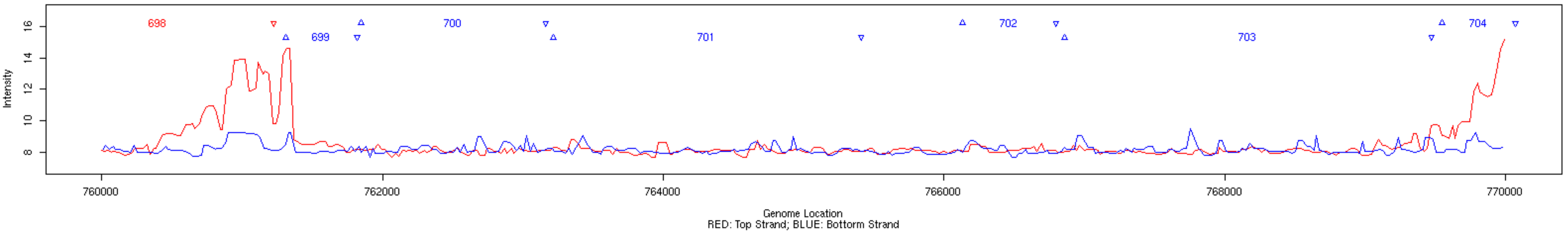
Intensity
Genome Location
RED: Top Strand; BLUE: Bottorm Strand

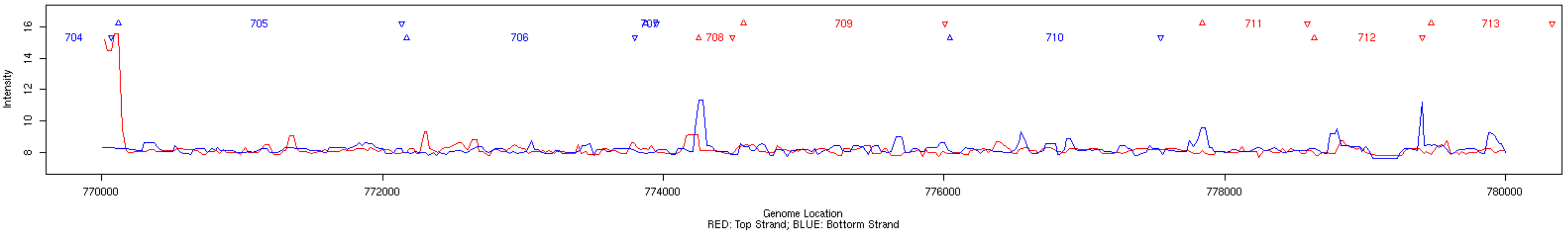
Intensity
Genome Location
RED: Top Strand; BLUE: Bottorm Strand

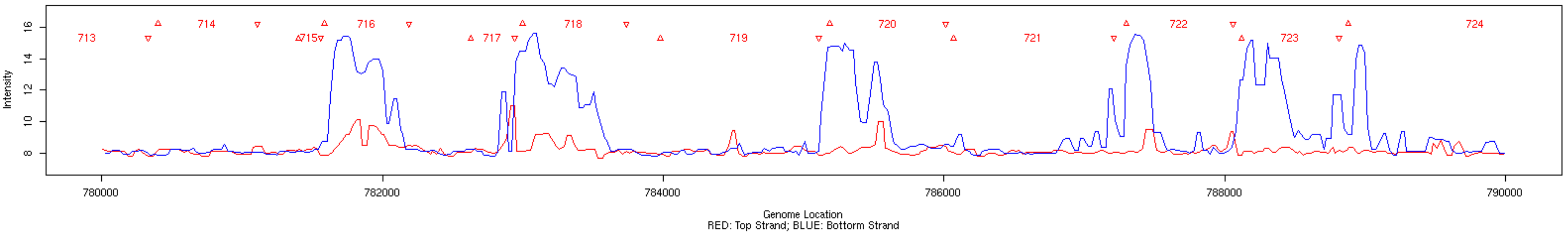
Intensity
Genome Location
RED: Top Strand; BLUE: Bottorm Strand

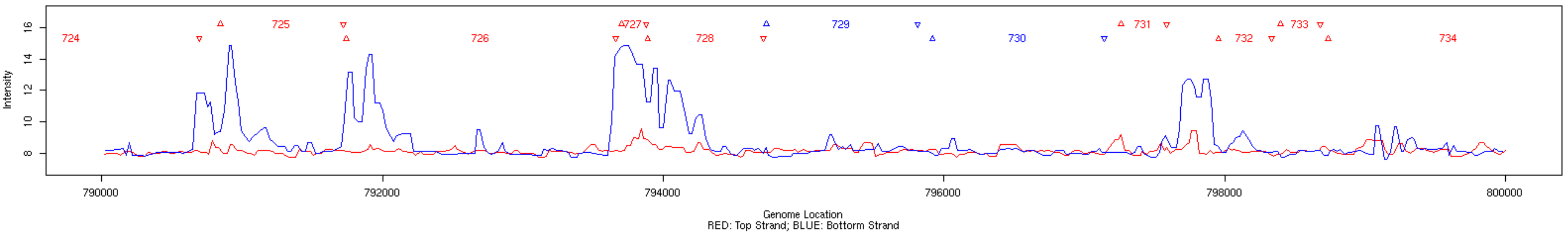
Intensity
Genome Location
RED: Top Strand; BLUE: Bottorm Strand

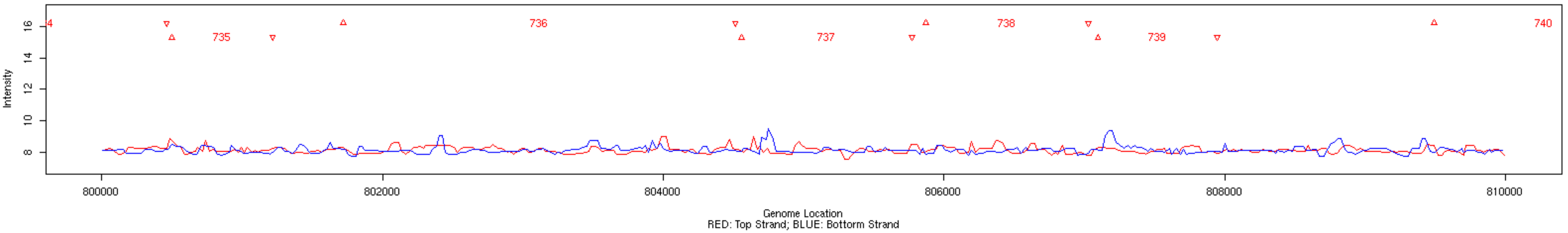
Intensity
Genome Location
RED: Top Strand; BLUE: Bottorm Strand
735
736
737
738
739
740

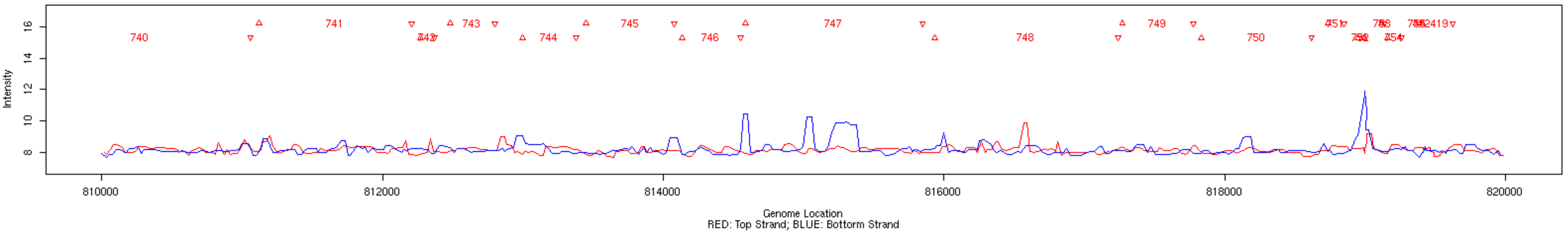
Intensity
Genome Location
RED: Top Strand; BLUE: Bottorm Strand
740
741
743
744
745
746
747
748
749
750

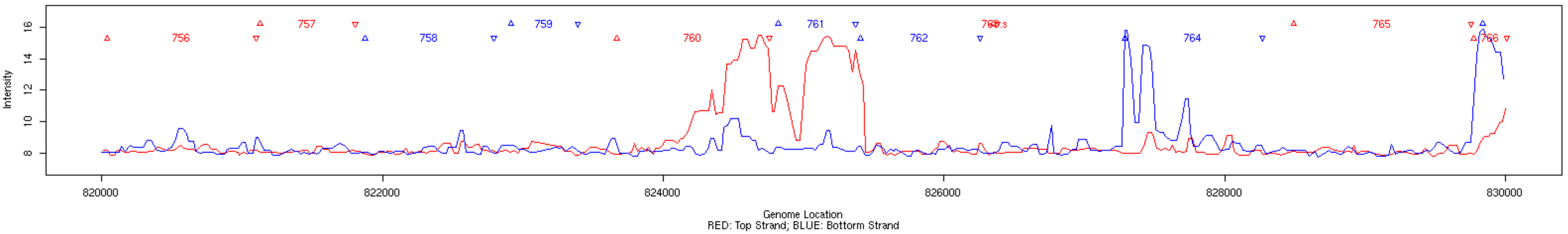
Intensity
Genome Location
RED: Top Strand; BLUE: Bottorm Strand
756
757
758
759
760
761
762
764
765

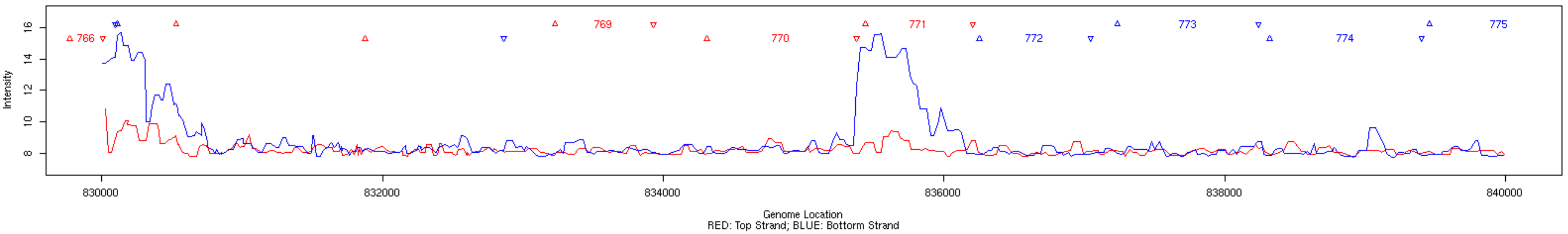
Intensity
Genome Location
RED: Top Strand; BLUE: Bottorm Strand
766
769
770
771
772
773
774
775

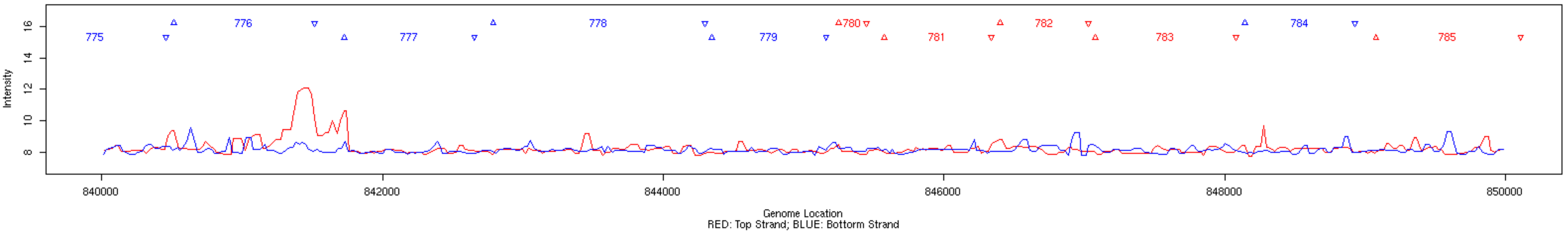
775
776
777
778
779
780
781
782
783
784
785
Intensity
840000
842000
844000
846000
848000
850000
Genome Location
RED: Top Strand; BLUE: Bottom Strand

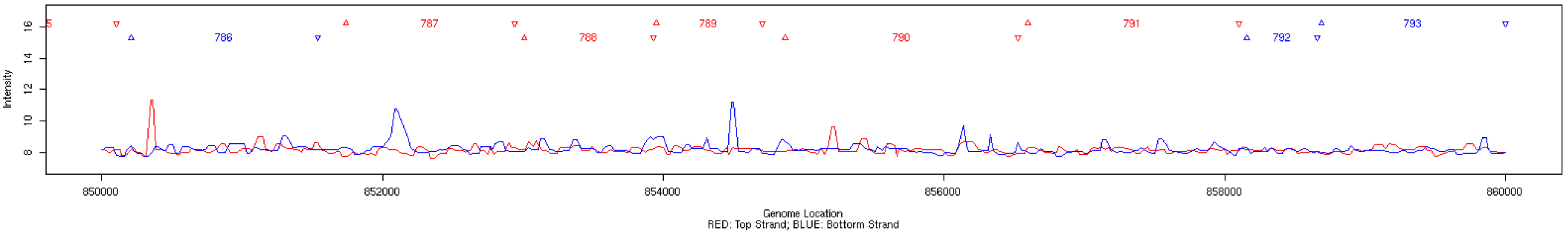
786
787
788
789
790
791
792
793
Intensity
850000
852000
854000
856000
858000
860000
Genome Location
RED: Top Strand; BLUE: Bottom Strand

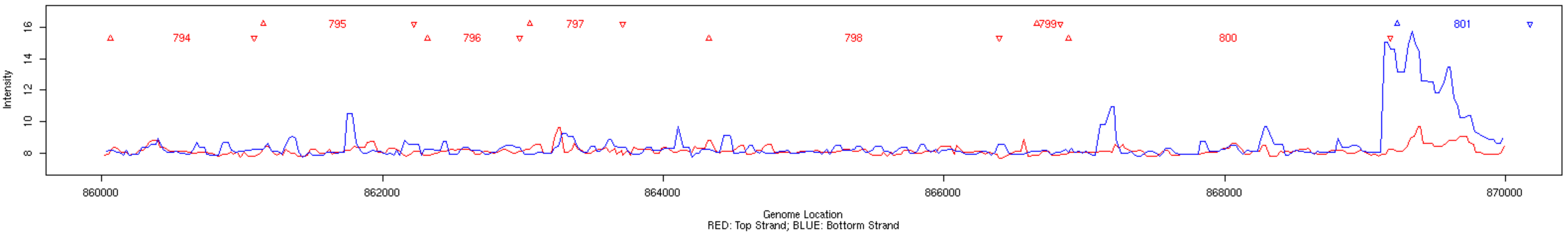
794
795
796
797
798
799
800
801
Intensity
860000
862000
864000
866000
868000
870000
Genome Location
RED: Top Strand; BLUE: Bottom Strand

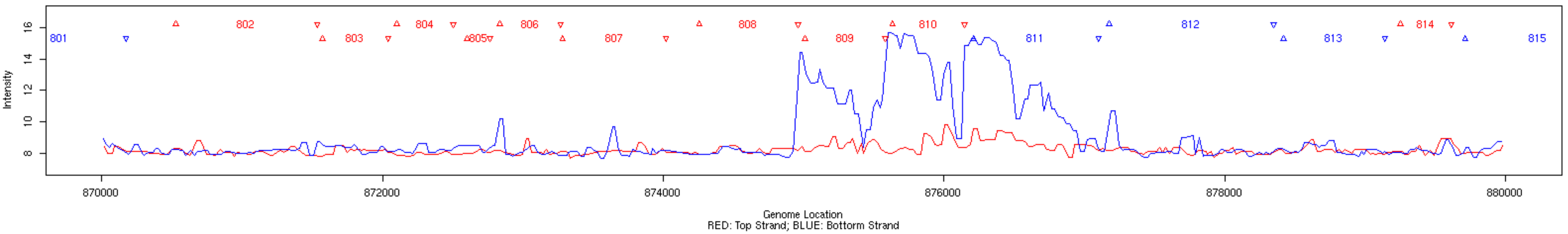
801
802
803
804
805
806
807
808
809
810
811
812
813
814
815
Intensity
870000
872000
874000
876000
878000
880000
Genome Location
RED: Top Strand; BLUE: Bottom Strand

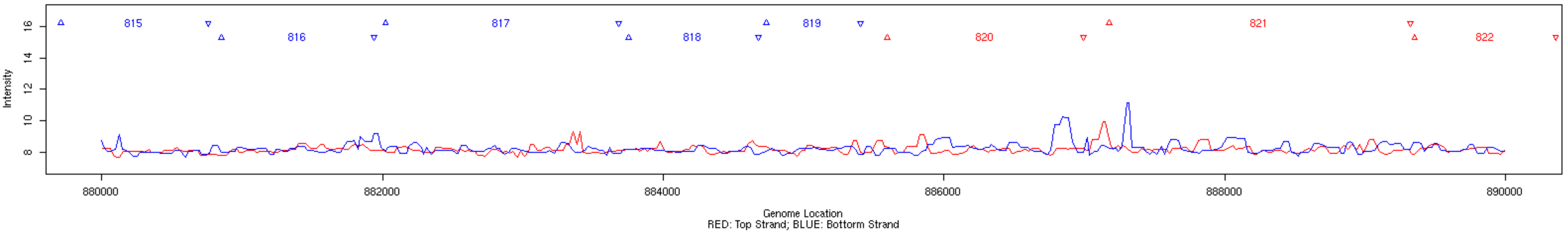
Intensity
815
816
817
818
819
820
821
822
880000
882000
884000
886000
888000
890000
Genome Location
RED: Top Strand; BLUE: Bottorm Strand

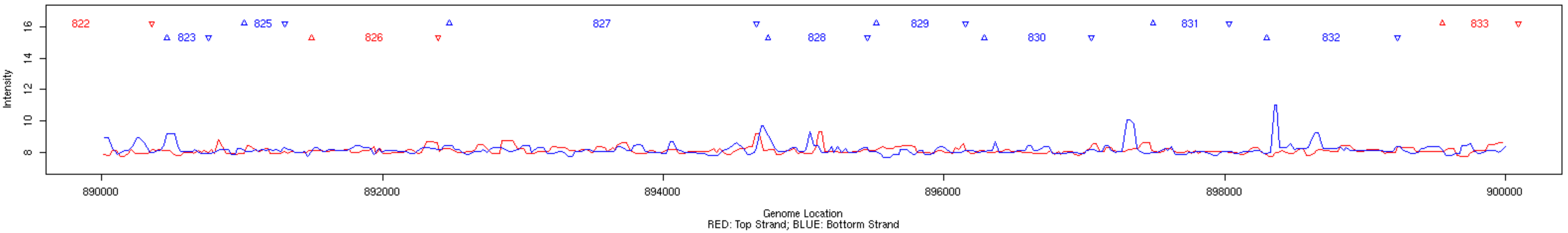
Intensity
822
823
825
826
827
828
829
830
831
832
833
890000
892000
894000
896000
898000
900000
Genome Location
RED: Top Strand; BLUE: Bottorm Strand

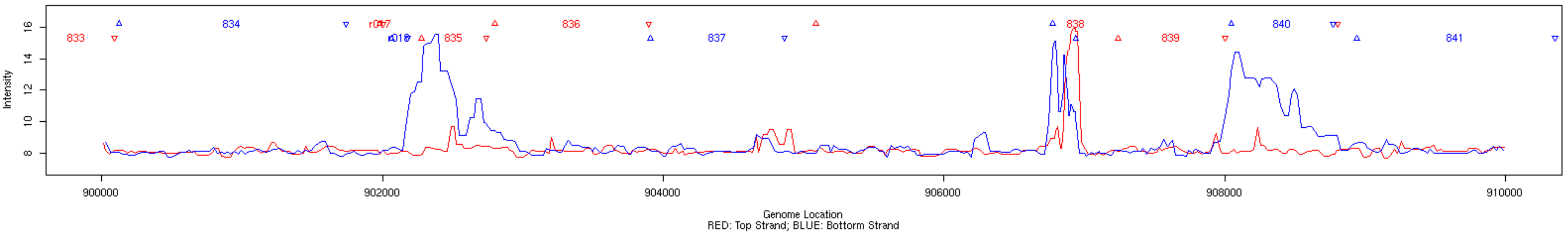
Intensity
833
834
835
836
837
838
839
840
841
900000
902000
904000
906000
908000
910000
Genome Location
RED: Top Strand; BLUE: Bottorm Strand

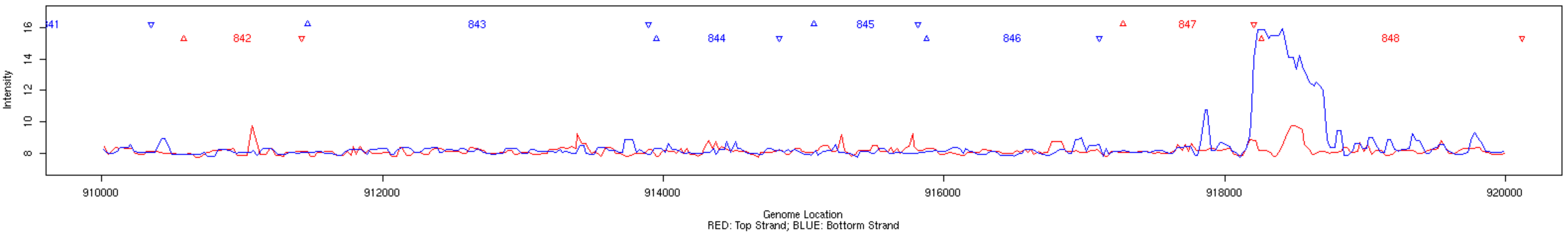
Intensity
842
843
844
845
846
847
848
910000
912000
914000
916000
918000
920000
Genome Location
RED: Top Strand; BLUE: Bottorm Strand

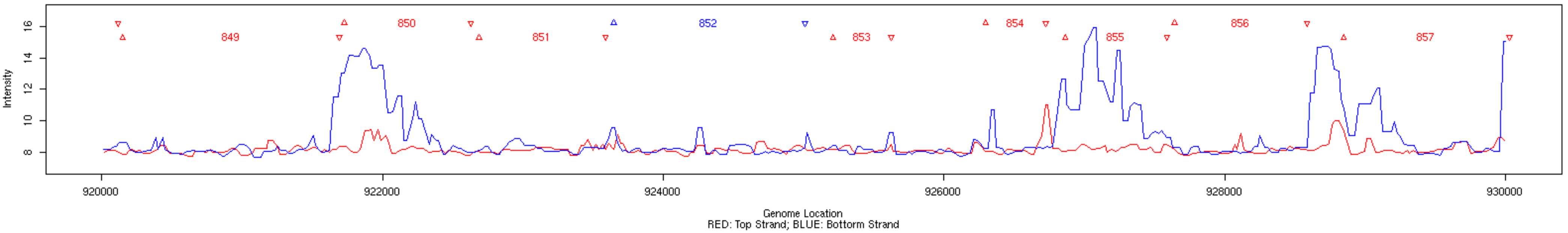
Intensity
16 14 12 10 8
849 850 851 852 853 854 855 856 857
920000 922000 924000 926000 928000 930000
Genome Location
RED: Top Strand; BLUE: Bottom Strand

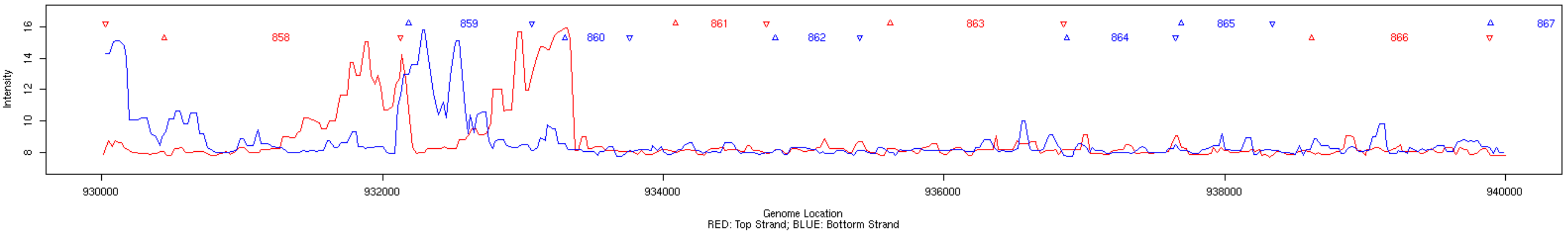
Intensity
16 14 12 10 8
858 859 860 861 862 863 864 865 866 867
930000 932000 934000 936000 938000 940000
Genome Location
RED: Top Strand; BLUE: Bottom Strand

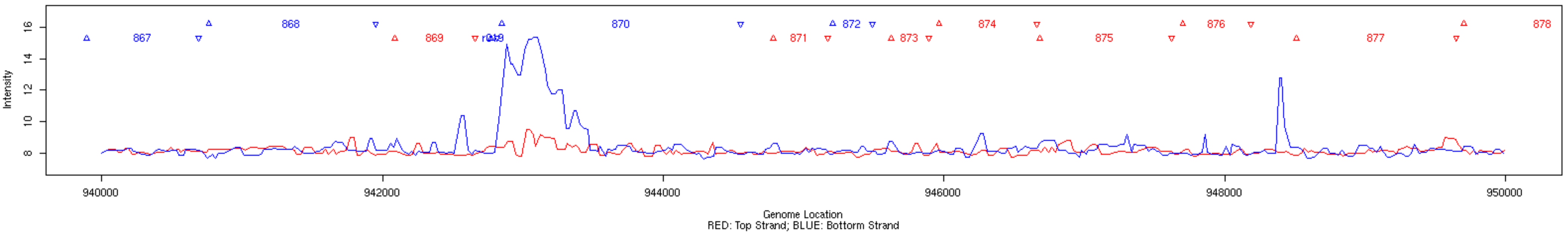
Intensity
16 14 12 10 8
867 868 869 870 871 872 873 874 875 876 877 878
940000 942000 944000 946000 948000 950000
Genome Location
RED: Top Strand; BLUE: Bottom Strand

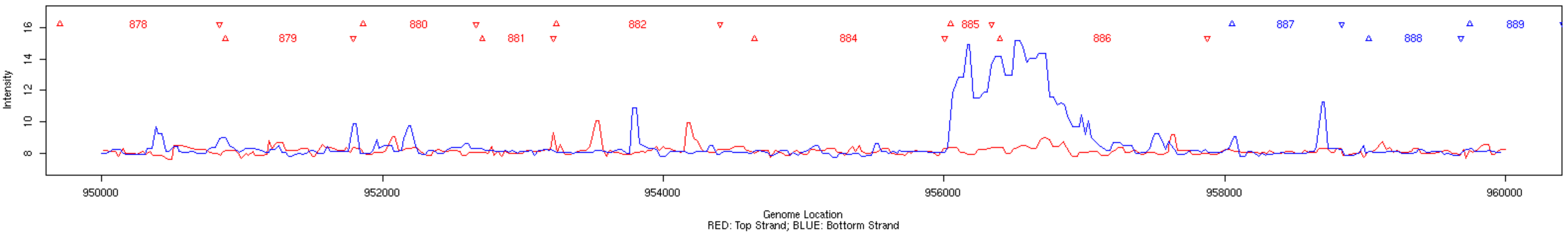
Intensity
16 14 12 10 8
878 879 880 881 882 884 885 886 887 888 889
950000 952000 954000 956000 958000 960000
Genome Location
RED: Top Strand; BLUE: Bottom Strand

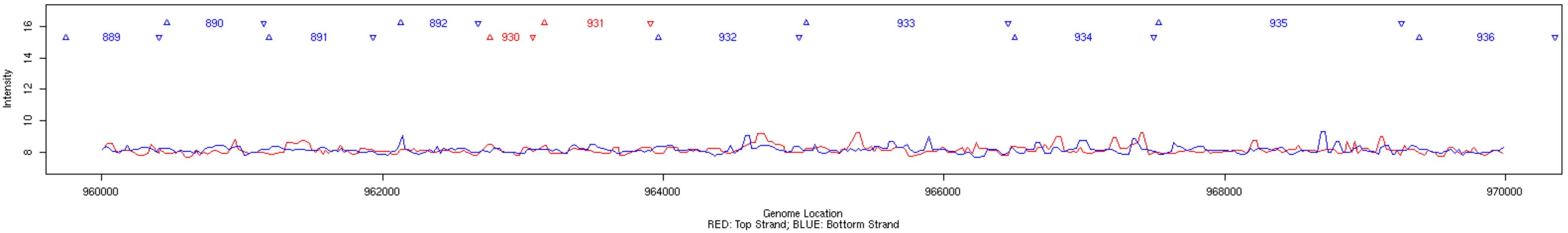
889
890
891
892
930
931
932
933
934
935
936
Intensity
960000
962000
964000
966000
968000
970000
Genome Location
RED: Top Strand; BLUE: Bottorm Strand

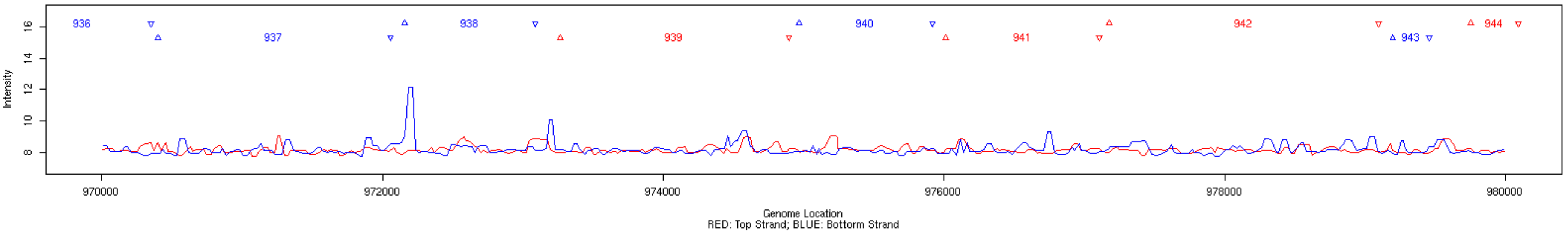
936
937
938
939
940
941
942
943
944
Intensity
970000
972000
974000
976000
978000
980000
Genome Location
RED: Top Strand; BLUE: Bottorm Strand

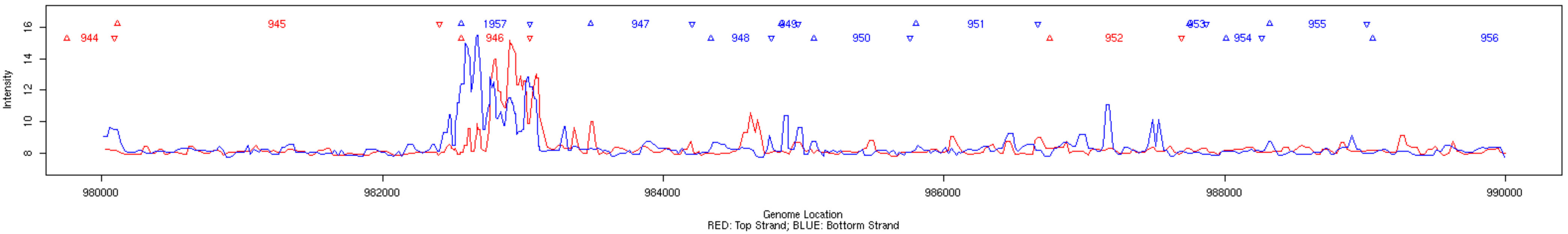
944
945
1957
946
947
948
950
951
952
954
955
956
Intensity
980000
982000
984000
986000
988000
990000
Genome Location
RED: Top Strand; BLUE: Bottorm Strand

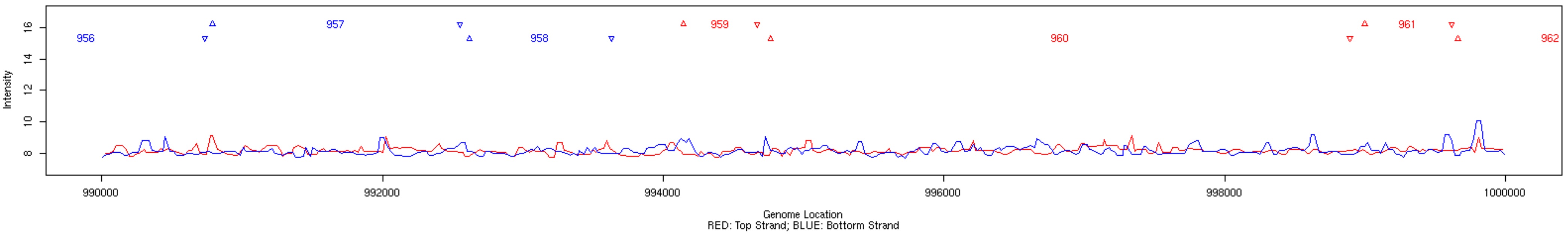
956
957
958
959
960
961
962
Intensity
990000
992000
994000
996000
998000
1000000
Genome Location
RED: Top Strand; BLUE: Bottorm Strand

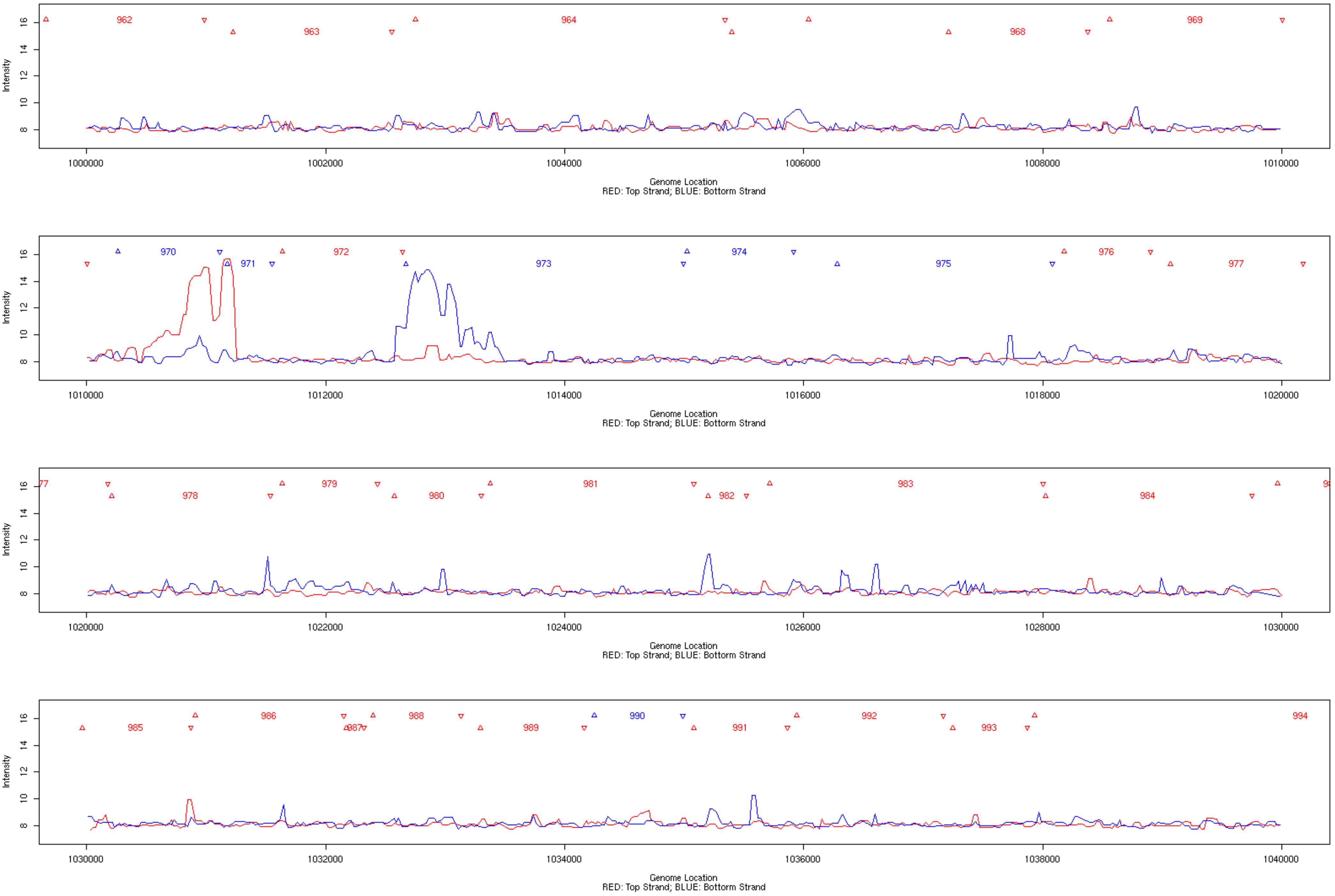

Intensity
Genome Location
RED: Top Strand; BLUE: Botto[rm] Strand
962 963 964 968 969
970 971 972 973 974 975 976 977
978 979 980 981 982 983 984
985 986 987 988 989 990 991 992 993 994

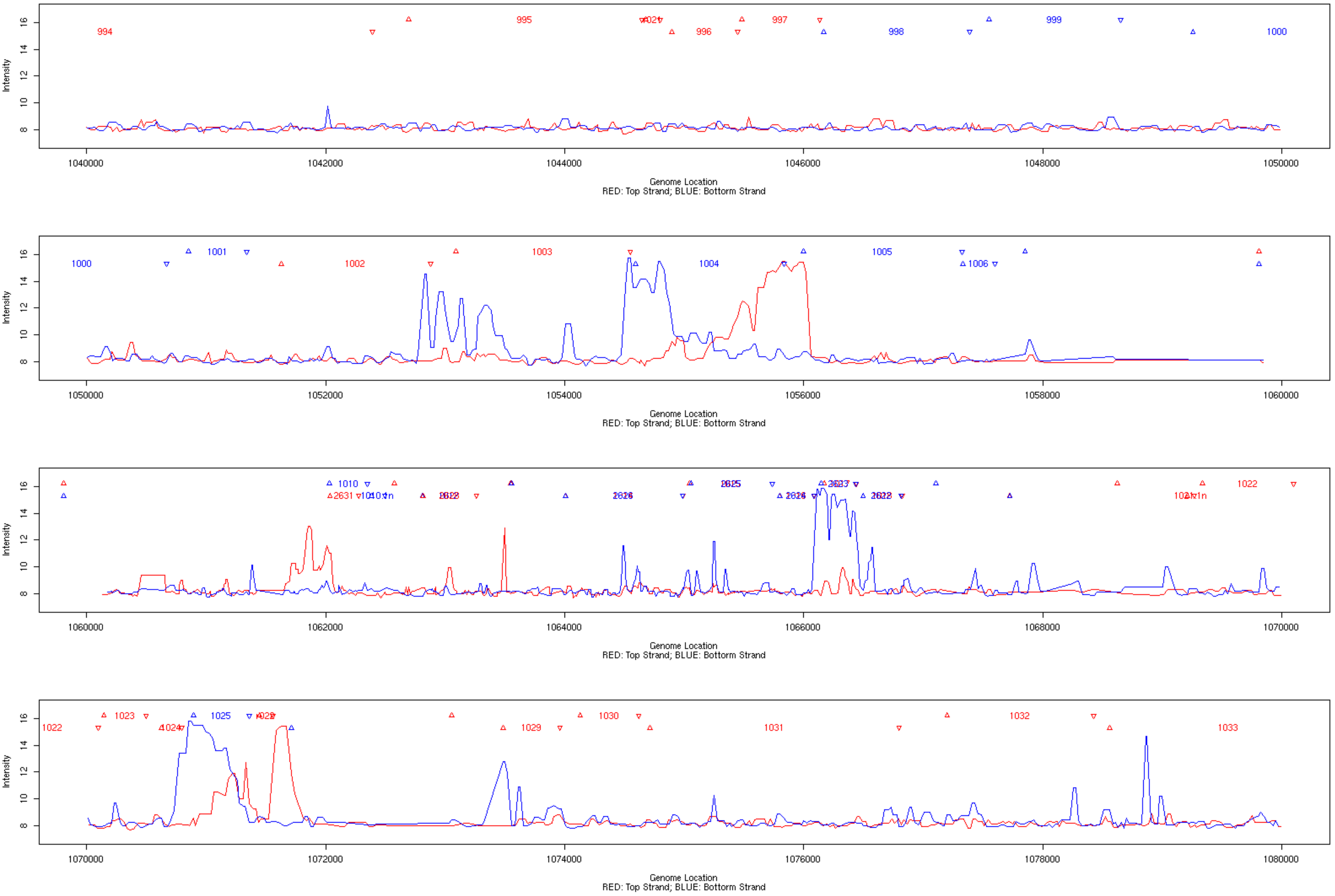
Intensity
Genome Location
RED: Top Strand; BLUE: Bottom Strand
994
995
996
997
998
999
1000
1001
1002
1003
1004
1005
1006
1010
2631
1022
1023
1025
1029
1030
1031
1032
1033
1040000
1042000
1044000
1046000
1048000
1050000
1052000
1054000
1056000
1058000
1060000
1062000
1064000
1066000
1068000
1070000
1072000
1074000
1076000
1078000
1080000

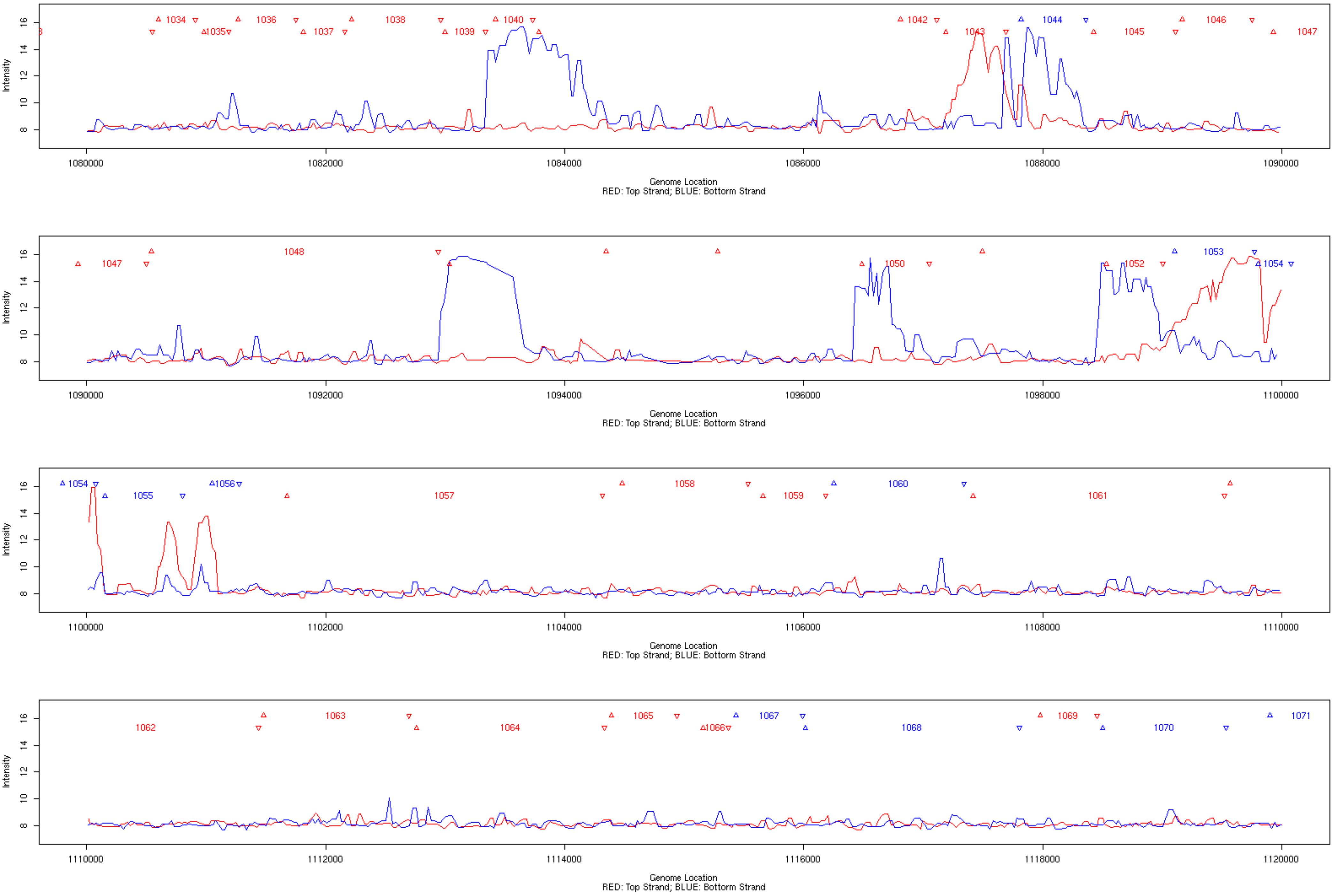
Intensity
Genome Location
RED: Top Strand; BLUE: Bottom Strand
1034 1035 1036 1037 1038 1039 1040 1042 1043 1044 1045 1046 1047
1047 1048 1050 1052 1053 1054
1054 1055 1056 1057 1058 1059 1060 1061
1062 1063 1064 1065 1066 1067 1068 1069 1070 1071

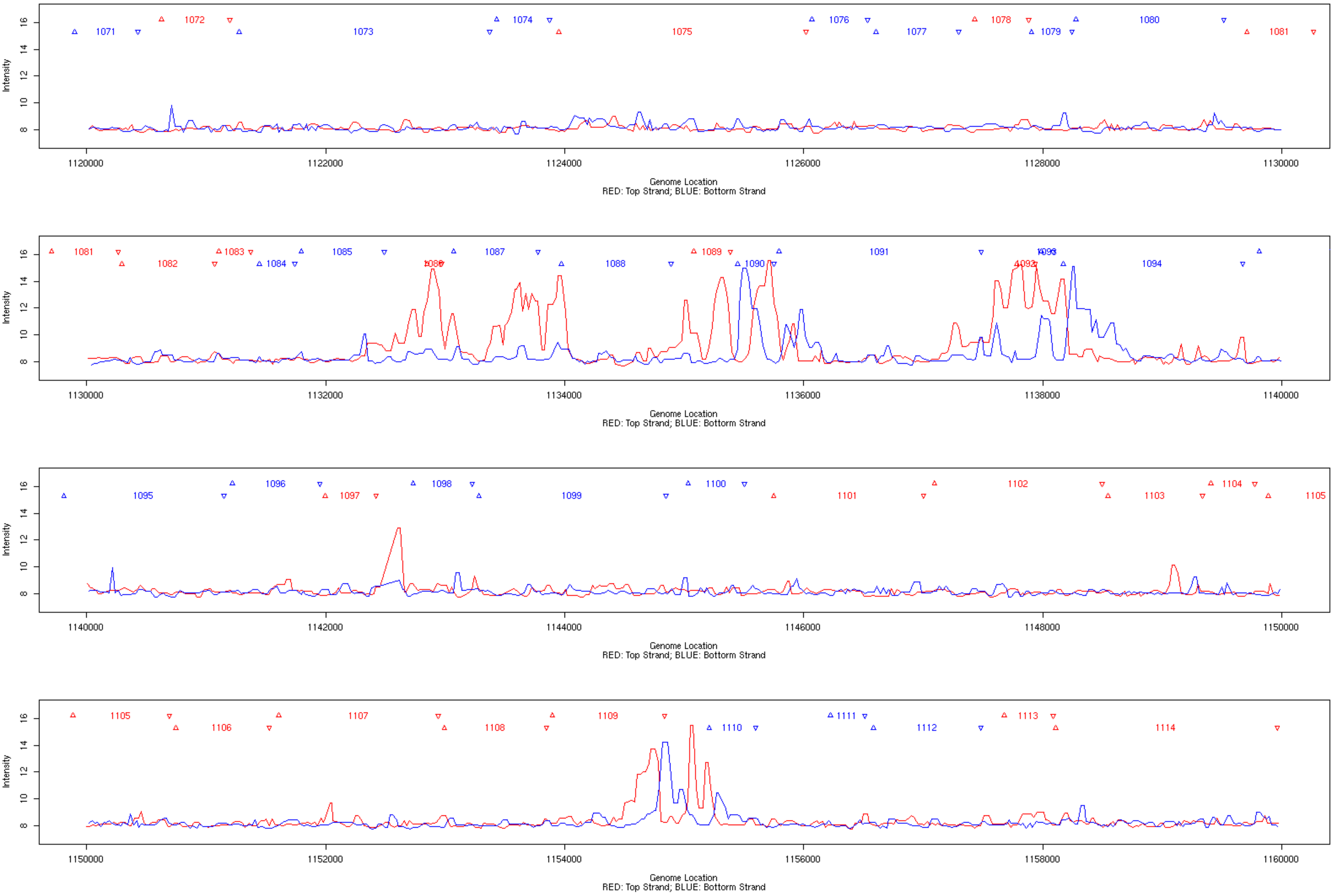
Intensity
Genome Location
RED: Top Strand; BLUE: Bottorm Strand
1071
1072
1073
1074
1075
1076
1077
1078
1079
1080
1081
1082
1083
1084
1085
1087
1088
1089
1090
1091
1094
1095
1096
1097
1098
1099
1100
1101
1102
1103
1104
1105
1106
1107
1108
1109
1110
1111
1112
1113
1114

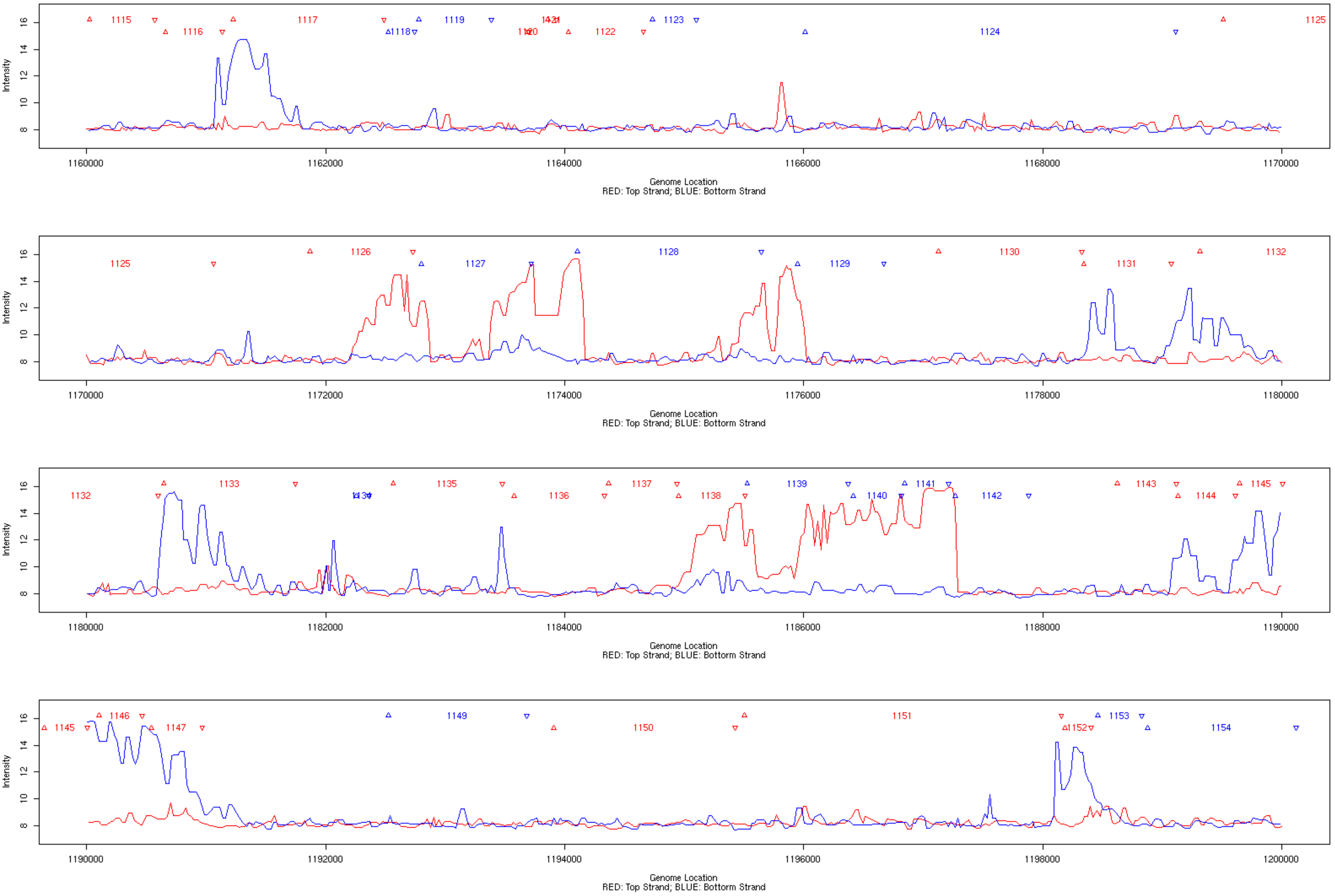
Intensity
1115
1116
1117
1118
1119
1122
1123
1124
1125
1160000
1162000
1164000
1166000
1168000
1170000
Genome Location
RED: Top Strand; BLUE: Bottorm Strand
Intensity
1125
1126
1127
1128
1129
1130
1131
1132
1170000
1172000
1174000
1176000
1178000
1180000
Genome Location
RED: Top Strand; BLUE: Bottorm Strand
Intensity
1132
1133
1135
1136
1137
1138
1139
1140
1141
1142
1143
1144
1145
1180000
1182000
1184000
1186000
1188000
1190000
Genome Location
RED: Top Strand; BLUE: Bottorm Strand
Intensity
1145
1146
1147
1149
1150
1151
1152
1153
1154
1190000
1192000
1194000
1196000
1198000
1200000
Genome Location
RED: Top Strand; BLUE: Bottorm Strand

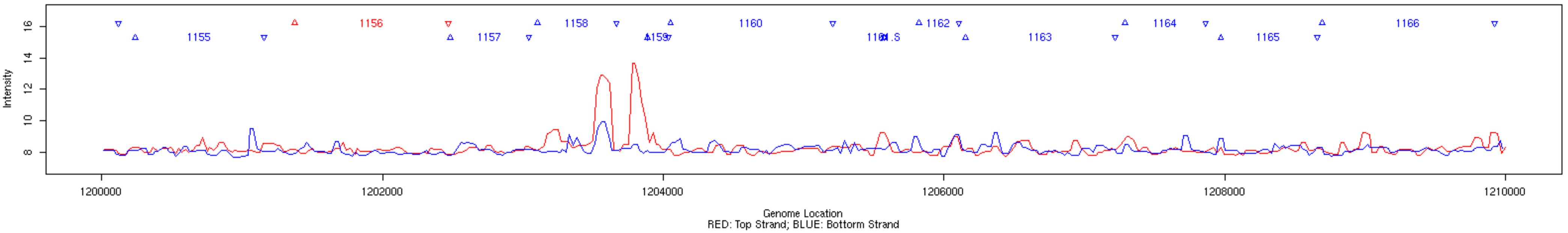
Intensity
Genome Location
RED: Top Strand; BLUE: Bottorm Strand
1155
1156
1157
1158
1160
1162
1163
1164
1165
1166
1200000
1202000
1204000
1206000
1208000
1210000

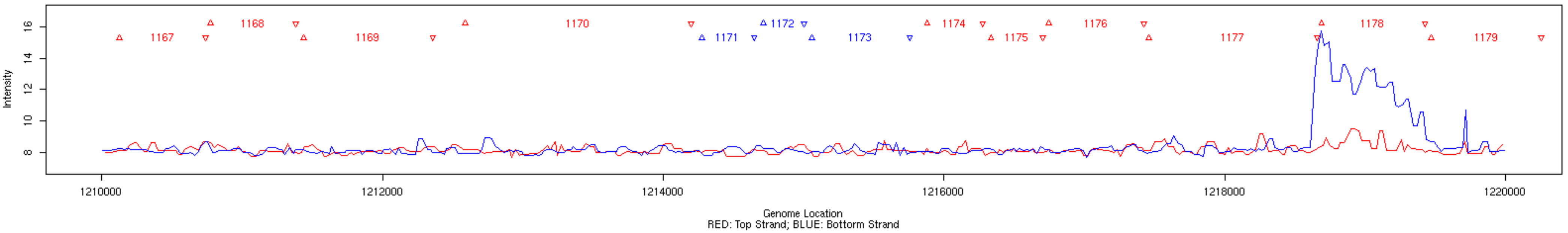
Intensity
Genome Location
RED: Top Strand; BLUE: Bottorm Strand
1167
1168
1169
1170
1171
1172
1173
1174
1175
1176
1177
1178
1179
1210000
1212000
1214000
1216000
1218000
1220000

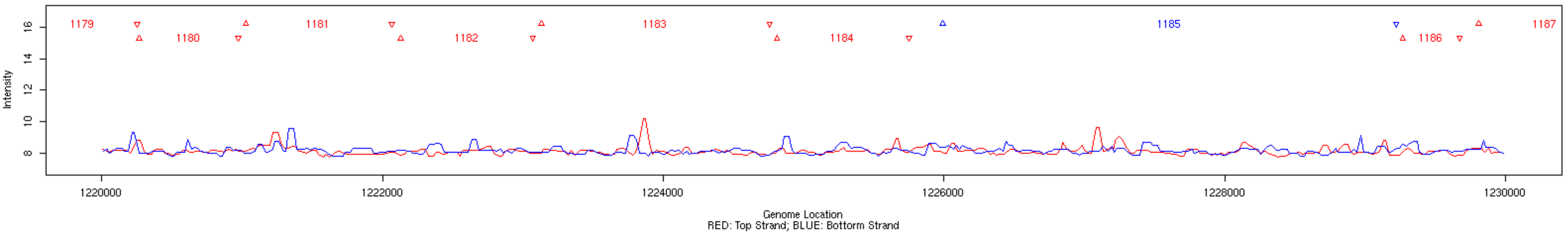
Intensity
Genome Location
RED: Top Strand; BLUE: Bottorm Strand
1179
1180
1181
1182
1183
1184
1185
1186
1187
1220000
1222000
1224000
1226000
1228000
1230000

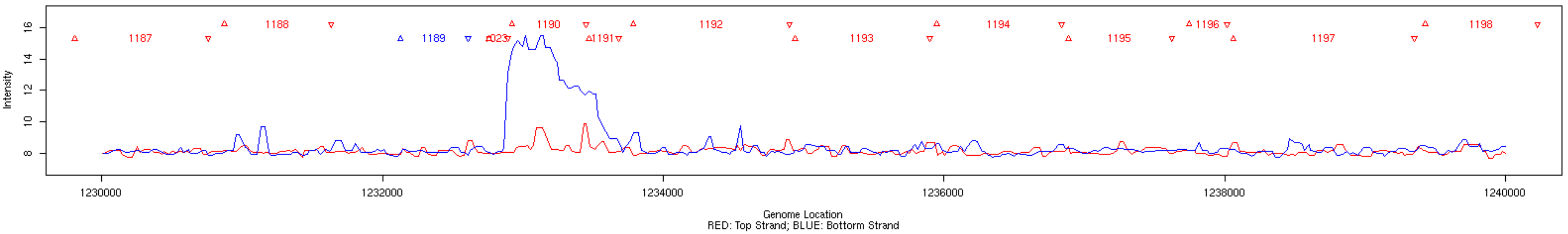
Intensity
Genome Location
RED: Top Strand; BLUE: Bottorm Strand
1187
1188
1189
1190
1191
1192
1193
1194
1195
1196
1197
1198
1230000
1232000
1234000
1236000
1238000
1240000

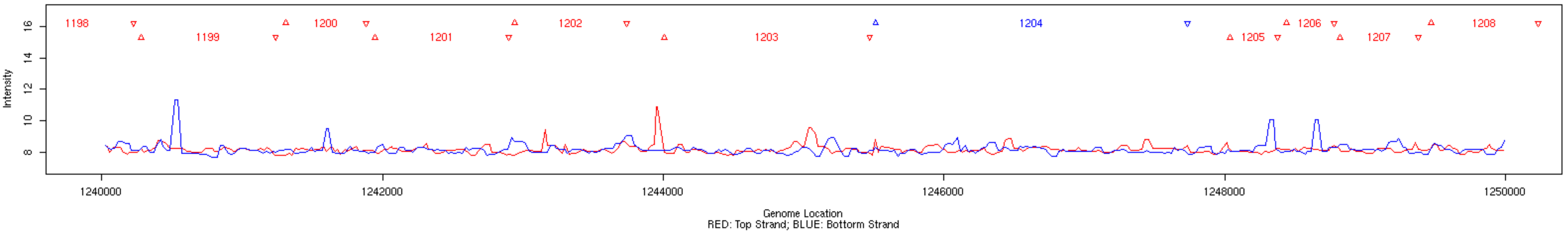
1198
1199
1200
1201
1202
1203
1204
1205
1206
1207
1208
Intensity
16
14
12
10
8
1240000
1242000
1244000
1246000
1248000
1250000
Genome Location
RED: Top Strand; BLUE: Bottorm Strand

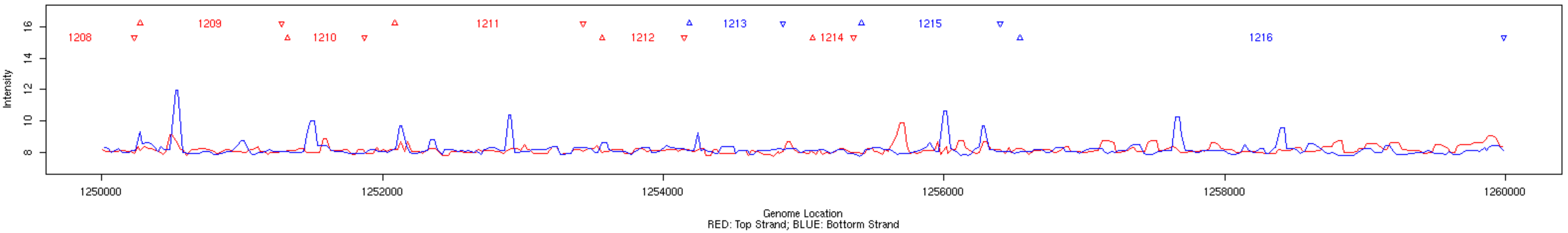
1208
1209
1210
1211
1212
1213
1214
1215
1216
Intensity
16
14
12
10
8
1250000
1252000
1254000
1256000
1258000
1260000
Genome Location
RED: Top Strand; BLUE: Bottorm Strand

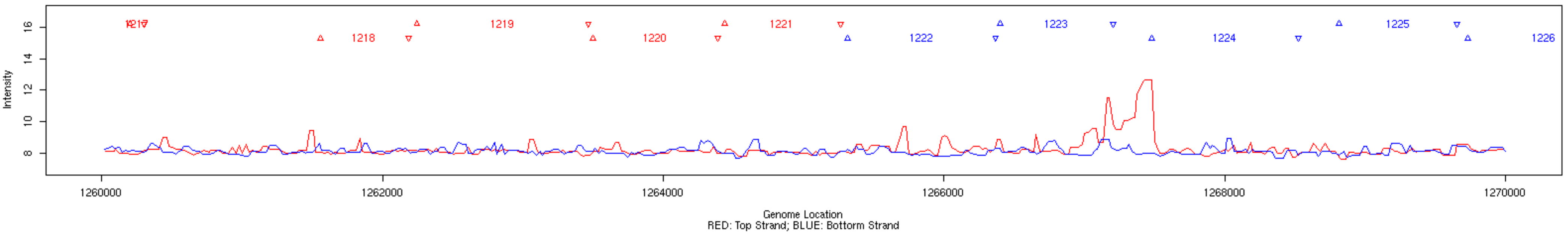
1218
1219
1220
1221
1222
1223
1224
1225
1226
Intensity
16
14
12
10
8
1260000
1262000
1264000
1266000
1268000
1270000
Genome Location
RED: Top Strand; BLUE: Bottorm Strand

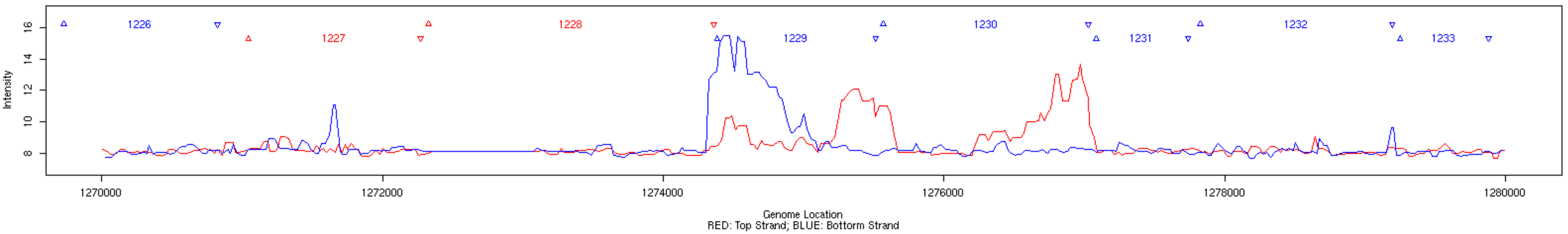
1226
1227
1228
1229
1230
1231
1232
1233
Intensity
16
14
12
10
8
1270000
1272000
1274000
1276000
1278000
1280000
Genome Location
RED: Top Strand; BLUE: Bottorm Strand

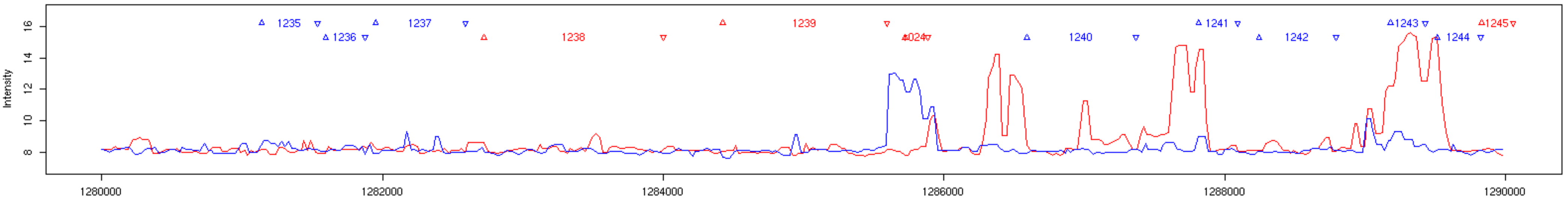
Genome Location
RED: Top Strand; BLUE: Bottom Strand

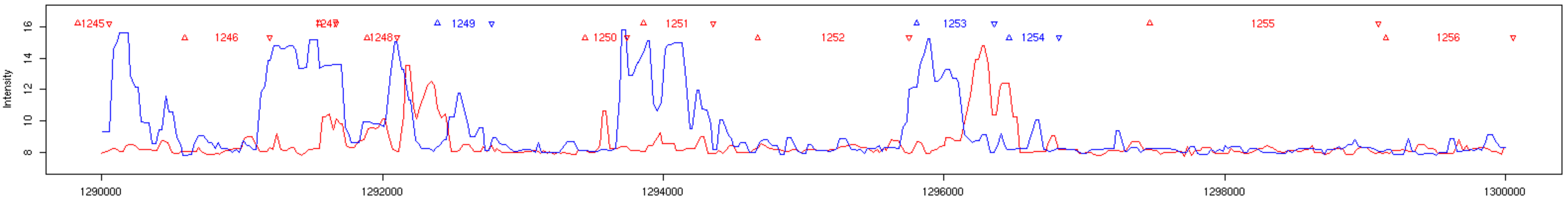
Genome Location
RED: Top Strand; BLUE: Bottom Strand

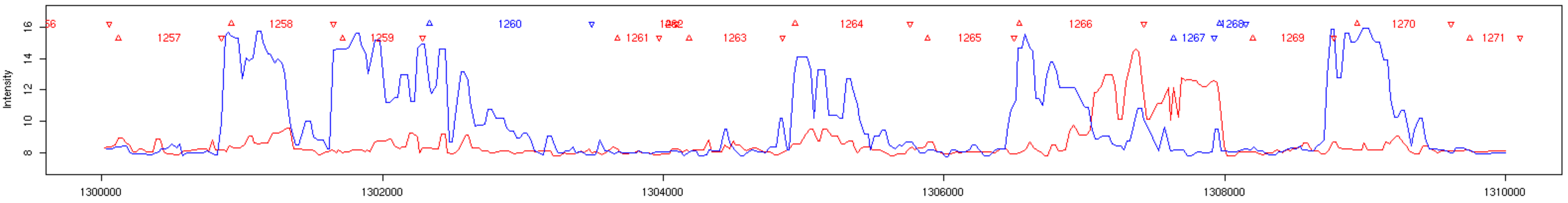
Genome Location
RED: Top Strand; BLUE: Bottom Strand

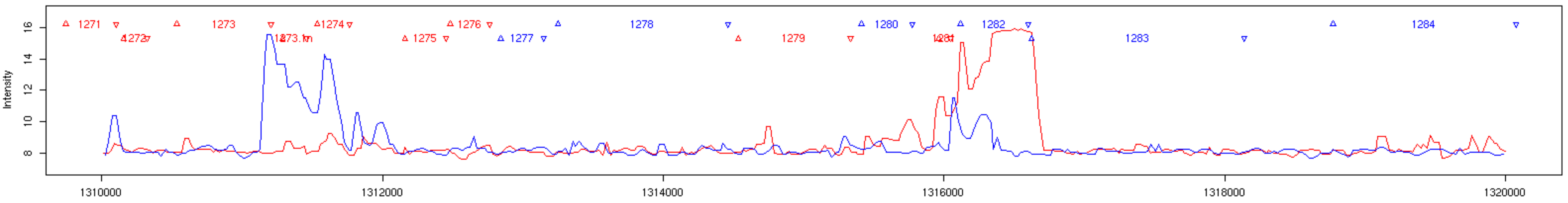
Genome Location
RED: Top Strand; BLUE: Bottom Strand

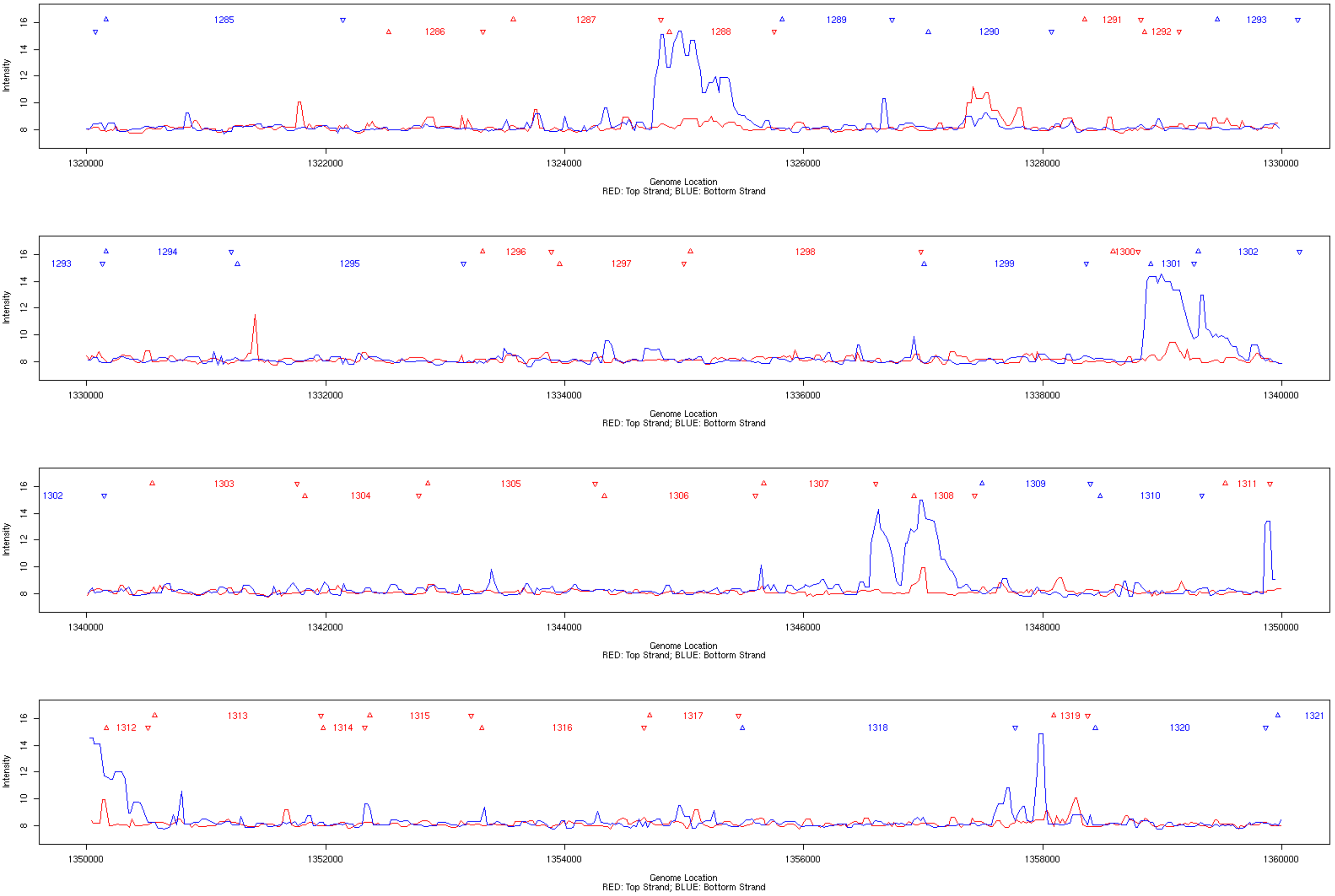
Intensity
1285
1286
1287
1288
1289
1290
1291
1292
1293
1320000
1322000
1324000
1326000
1328000
1330000
Genome Location
RED: Top Strand; BLUE: Bottom Strand
Intensity
1293
1294
1295
1296
1297
1298
1299
1300
1301
1302
1330000
1332000
1334000
1336000
1338000
1340000
Genome Location
RED: Top Strand; BLUE: Bottom Strand
Intensity
1302
1303
1304
1305
1306
1307
1308
1309
1310
1311
1340000
1342000
1344000
1346000
1348000
1350000
Genome Location
RED: Top Strand; BLUE: Bottom Strand
Intensity
1312
1313
1314
1315
1316
1317
1318
1319
1320
1321
1350000
1352000
1354000
1356000
1358000
1360000
Genome Location
RED: Top Strand; BLUE: Bottom Strand

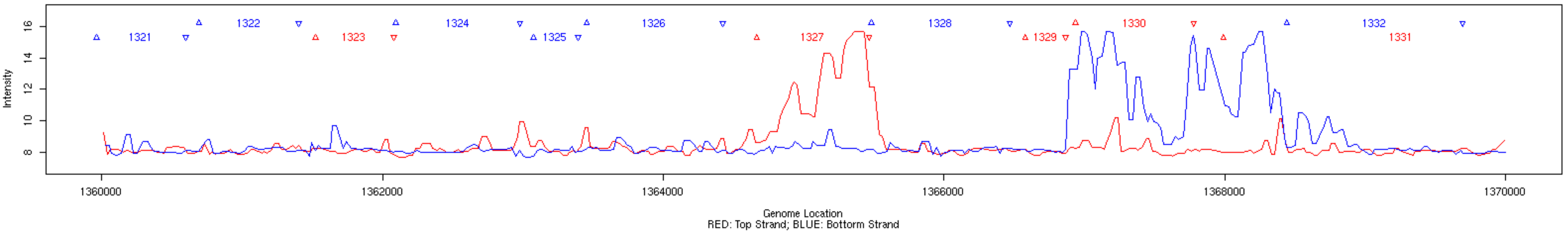
Intensity
16
14
12
10
8
1321
1322
1323
1324
1325
1326
1327
1328
1329
1330
1331
1332
1360000
1362000
1364000
1366000
1368000
1370000
Genome Location
RED: Top Strand; BLUE: Bottorm Strand

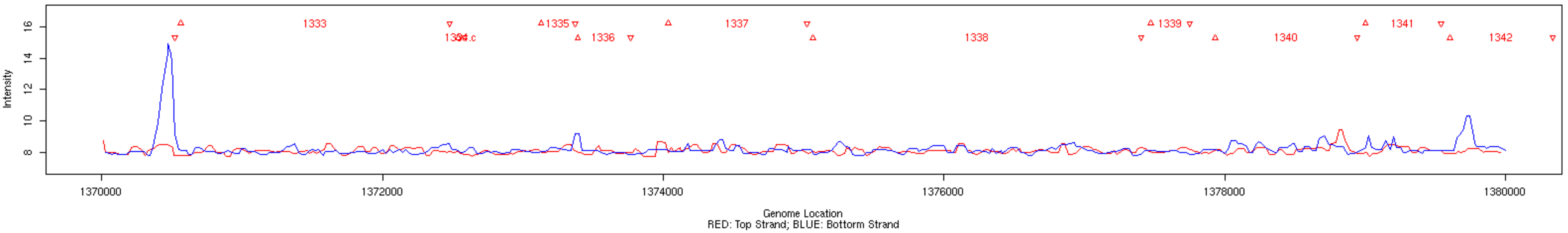
Intensity
16
14
12
10
8
1333
1335
1336
1337
1338
1339
1340
1341
1342
1370000
1372000
1374000
1376000
1378000
1380000
Genome Location
RED: Top Strand; BLUE: Bottorm Strand

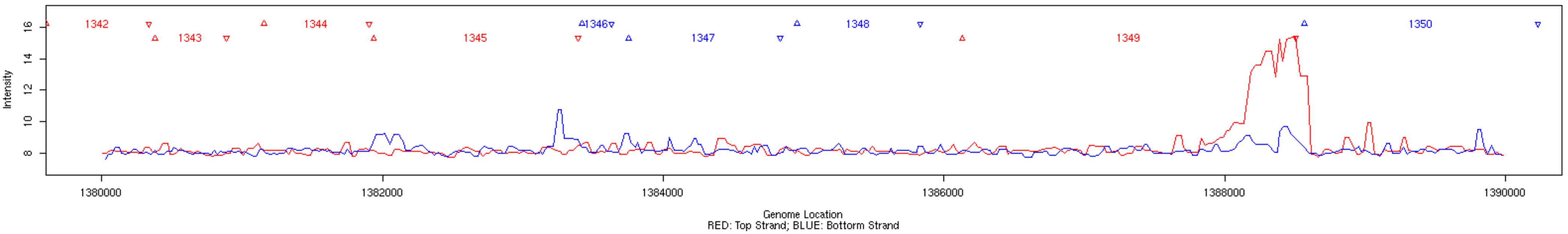
Intensity
16
14
12
10
8
1342
1343
1344
1345
1346
1347
1348
1349
1350
1380000
1382000
1384000
1386000
1388000
1390000
Genome Location
RED: Top Strand; BLUE: Bottorm Strand

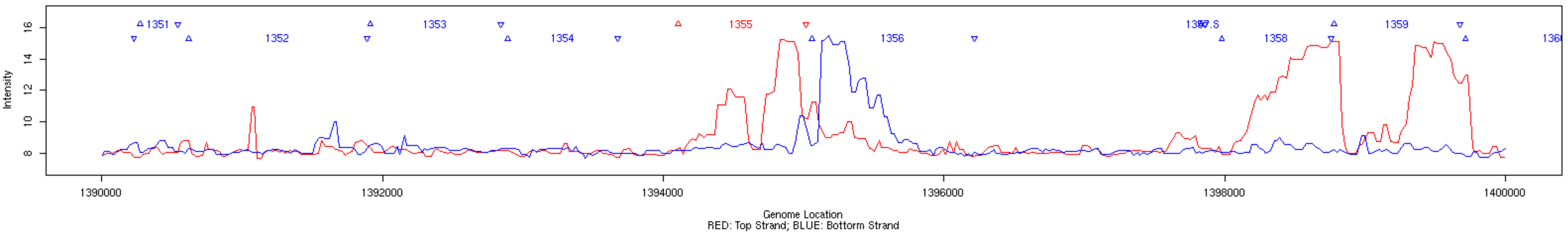
Intensity
16
14
12
10
8
1351
1352
1353
1354
1355
1356
1358
1359
1390000
1392000
1394000
1396000
1398000
1400000
Genome Location
RED: Top Strand; BLUE: Bottorm Strand

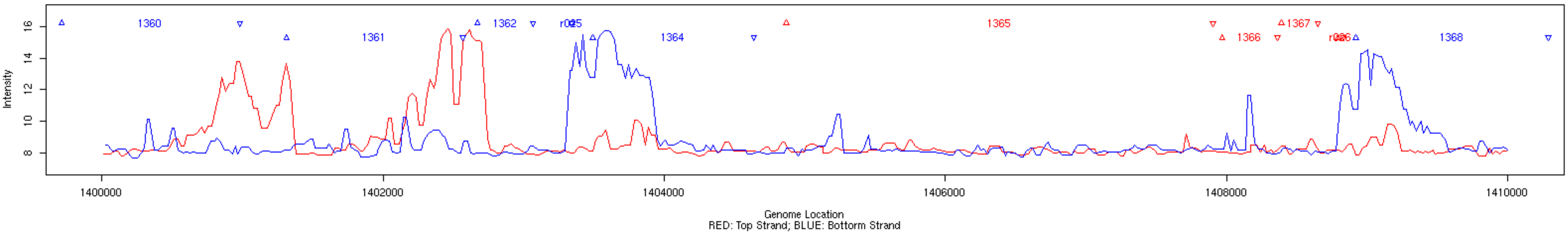
Intensity
Genome Location
RED: Top Strand; BLUE: Bottorm Strand

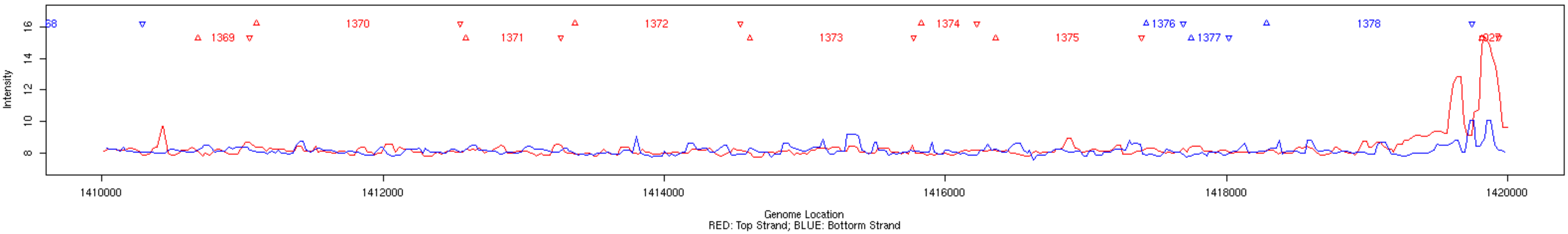
Intensity
Genome Location
RED: Top Strand; BLUE: Bottorm Strand

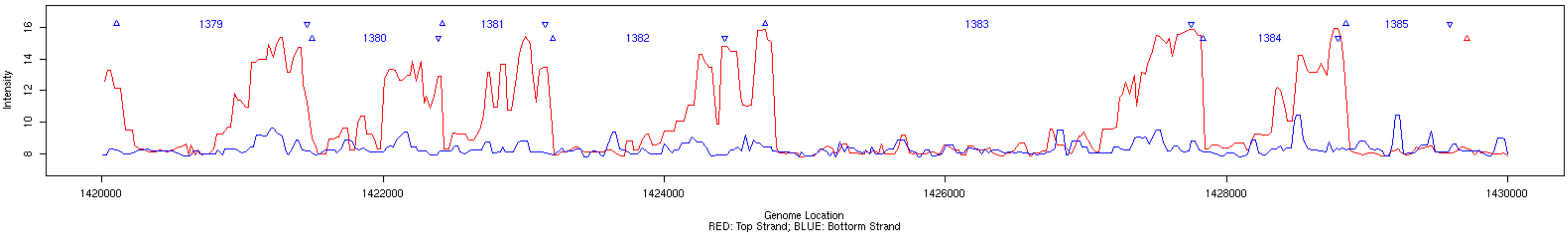
Intensity
Genome Location
RED: Top Strand; BLUE: Bottorm Strand

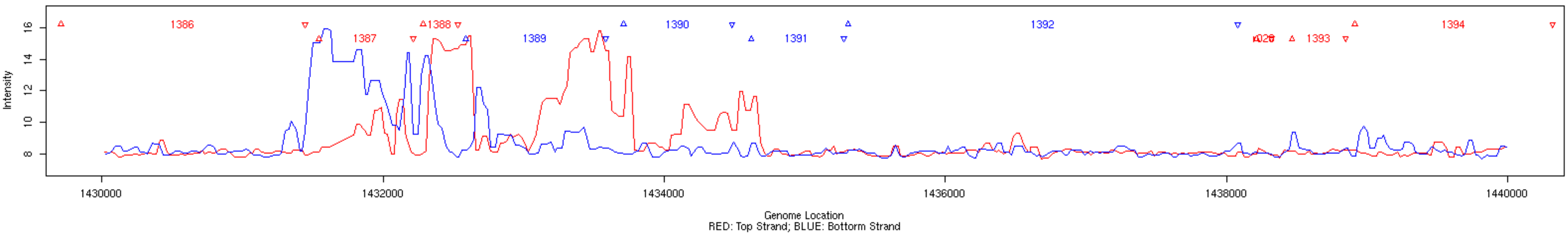
Intensity
Genome Location
RED: Top Strand; BLUE: Bottorm Strand

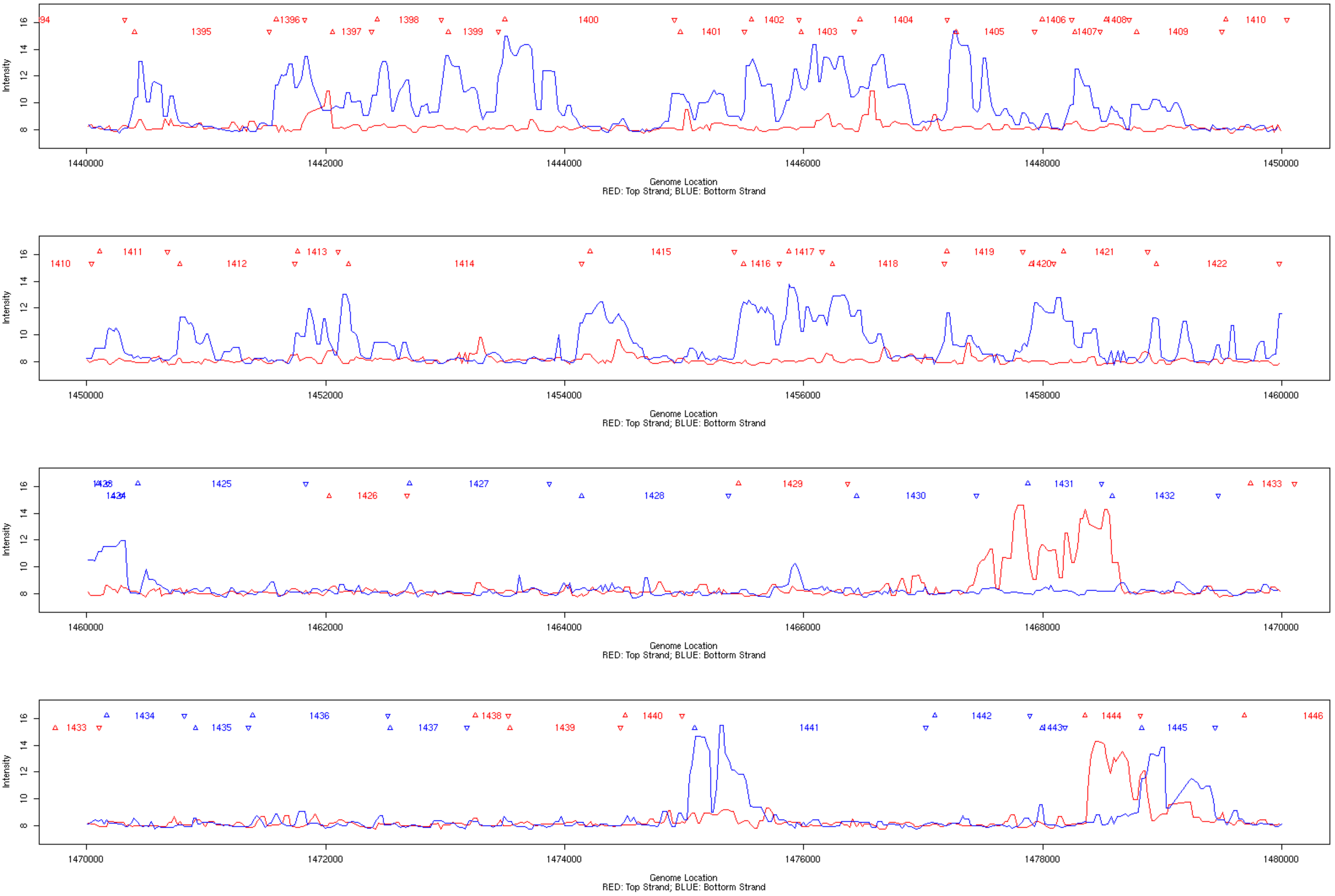
Intensity
Genome Location
RED: Top Strand; BLUE: Bottorm Strand
1395
1396
1397
1398
1399
1400
1401
1402
1403
1404
1405
1406
1407
1408
1409
1410
1411
1412
1413
1414
1415
1416
1417
1418
1419
1420
1421
1422
1425
1426
1427
1428
1429
1430
1431
1432
1433
1434
1435
1436
1437
1438
1439
1440
1441
1442
1443
1444
1445
1446

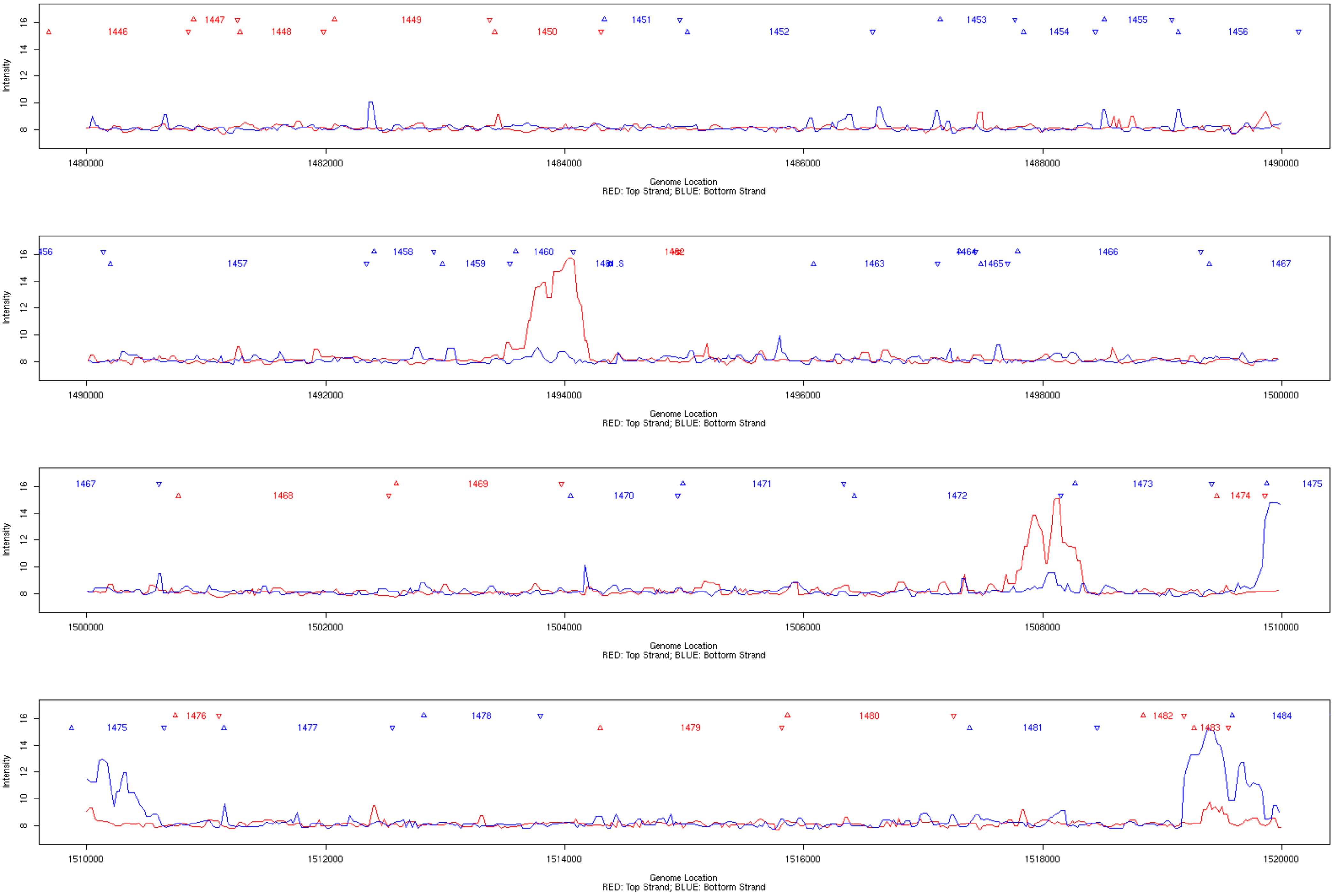
1446
1447
1448
1449
1450
1451
1452
1453
1454
1455
1456
Intensity
1480000
1482000
1484000
1486000
1488000
1490000
Genome Location
RED: Top Strand; BLUE: Bottorm Strand
1457
1458
1459
1460
1463
1466
1467
Intensity
1490000
1492000
1494000
1496000
1498000
1500000
Genome Location
RED: Top Strand; BLUE: Bottorm Strand
1467
1468
1469
1470
1471
1472
1473
1474
1475
Intensity
1500000
1502000
1504000
1506000
1508000
1510000
Genome Location
RED: Top Strand; BLUE: Bottorm Strand
1475
1476
1477
1478
1479
1480
1481
1482
1484
Intensity
1510000
1512000
1514000
1516000
1518000
1520000
Genome Location
RED: Top Strand; BLUE: Bottorm Strand

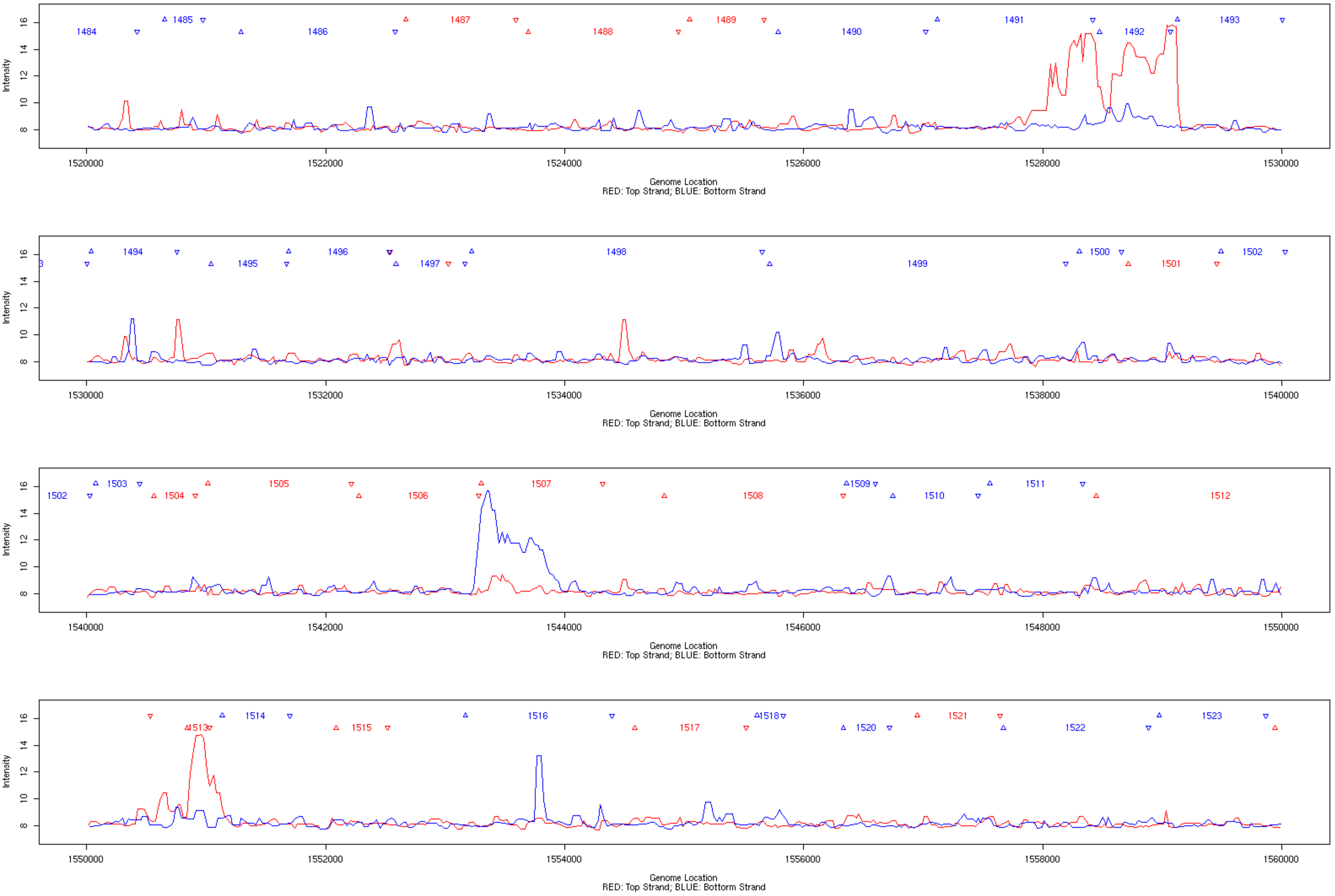
Intensity
1484
1485
1486
1487
1488
1489
1490
1491
1492
1493
1520000
1522000
1524000
1526000
1528000
1530000
Genome Location
RED: Top Strand; BLUE: Bottorm Strand
Intensity
1494
1495
1496
1497
1498
1499
1500
1501
1502
1530000
1532000
1534000
1536000
1538000
1540000
Genome Location
RED: Top Strand; BLUE: Bottorm Strand
Intensity
1502
1503
1504
1505
1506
1507
1508
1509
1510
1511
1512
1540000
1542000
1544000
1546000
1548000
1550000
Genome Location
RED: Top Strand; BLUE: Bottorm Strand
Intensity
1513
1514
1515
1516
1517
1518
1520
1521
1522
1523
1550000
1552000
1554000
1556000
1558000
1560000
Genome Location
RED: Top Strand; BLUE: Bottorm Strand

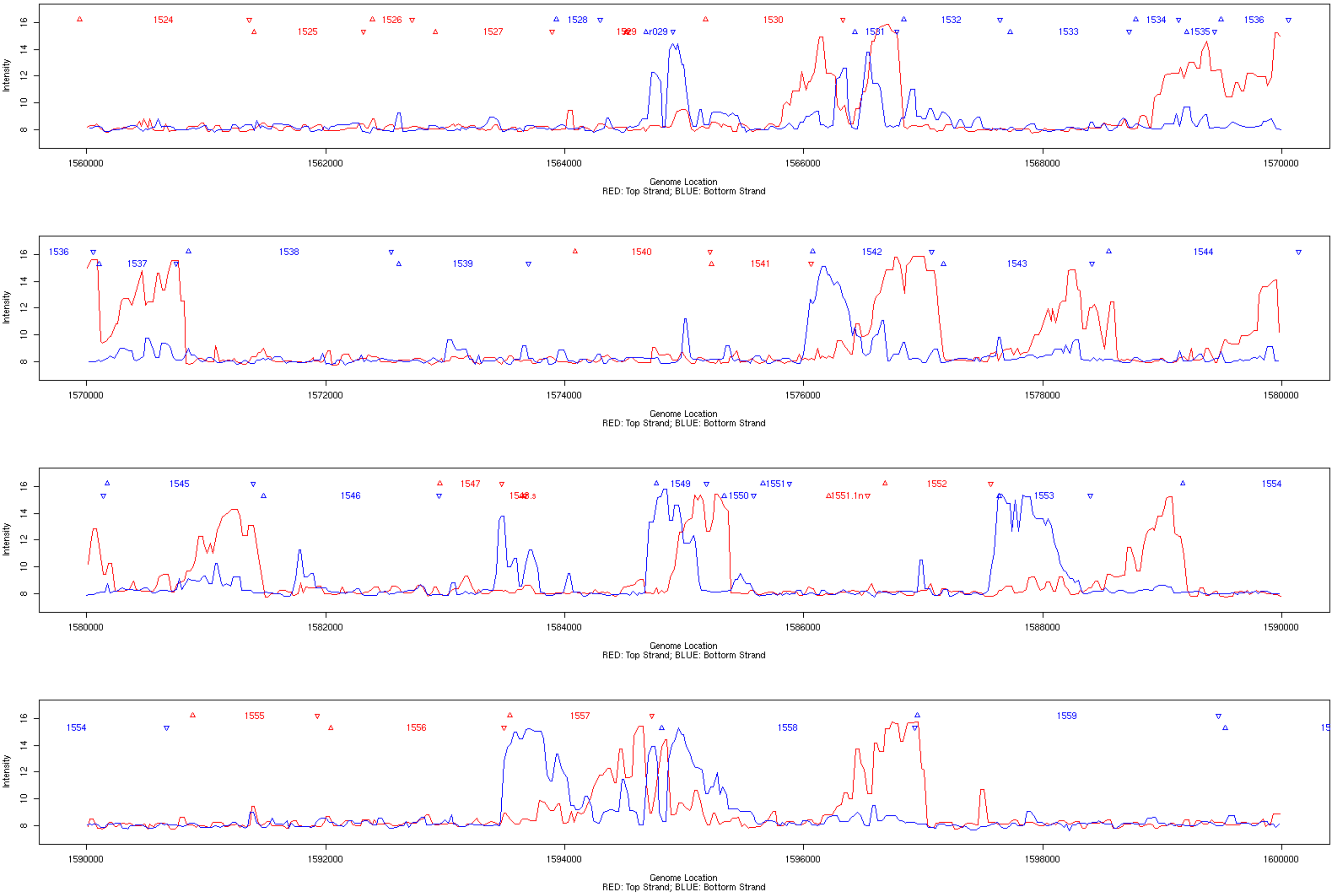
Intensity
Genome Location
RED: Top Strand; BLUE: Bottorm Strand

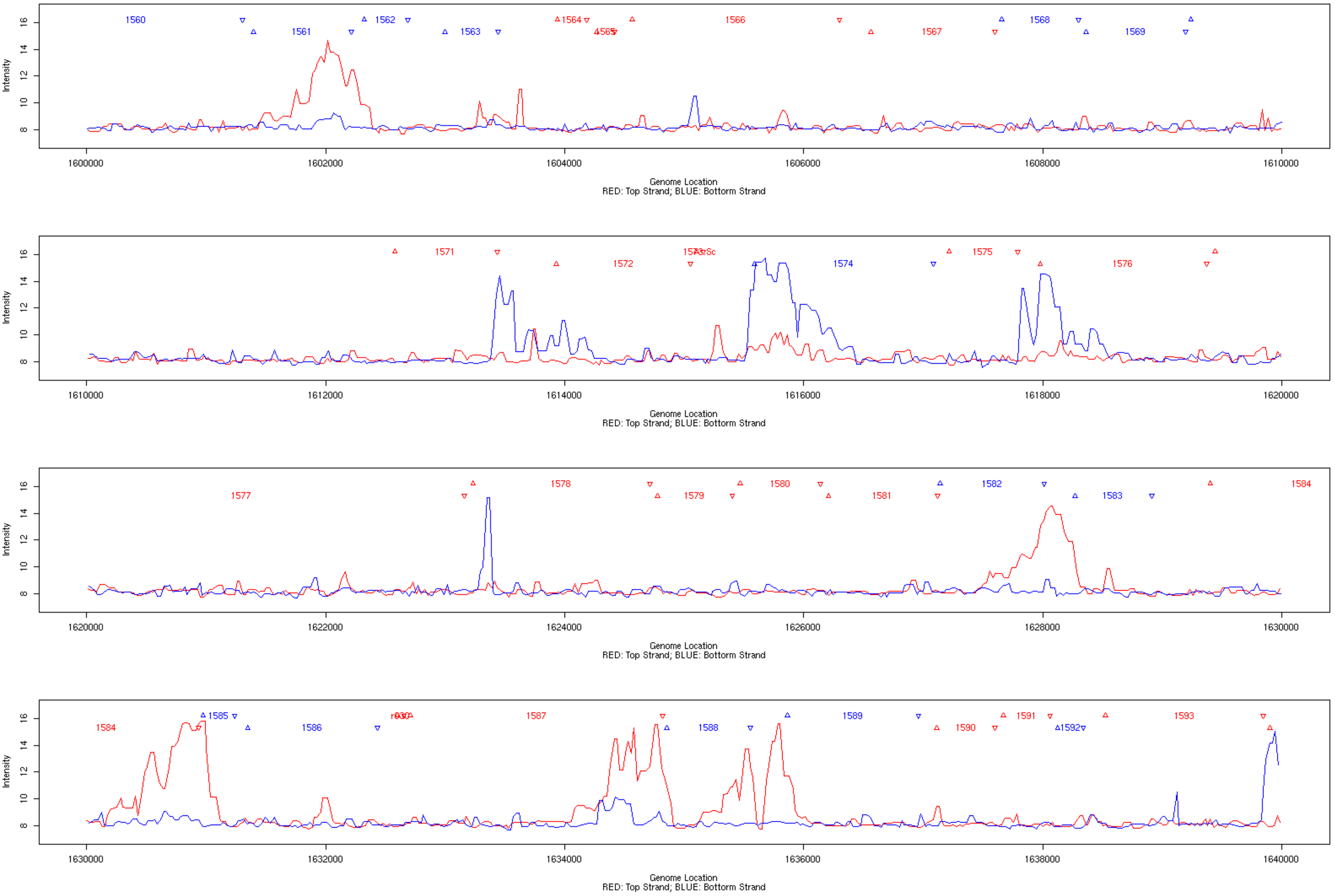
Intensity
Genome Location
RED: Top Strand; BLUE: Bottorm Strand
1560
1561
1562
1563
1564
1566
1567
1568
1569
1571
1572
1574
1575
1576
1577
1578
1579
1580
1581
1582
1583
1584
1585
1586
1587
1588
1589
1590
1591
1592
1593

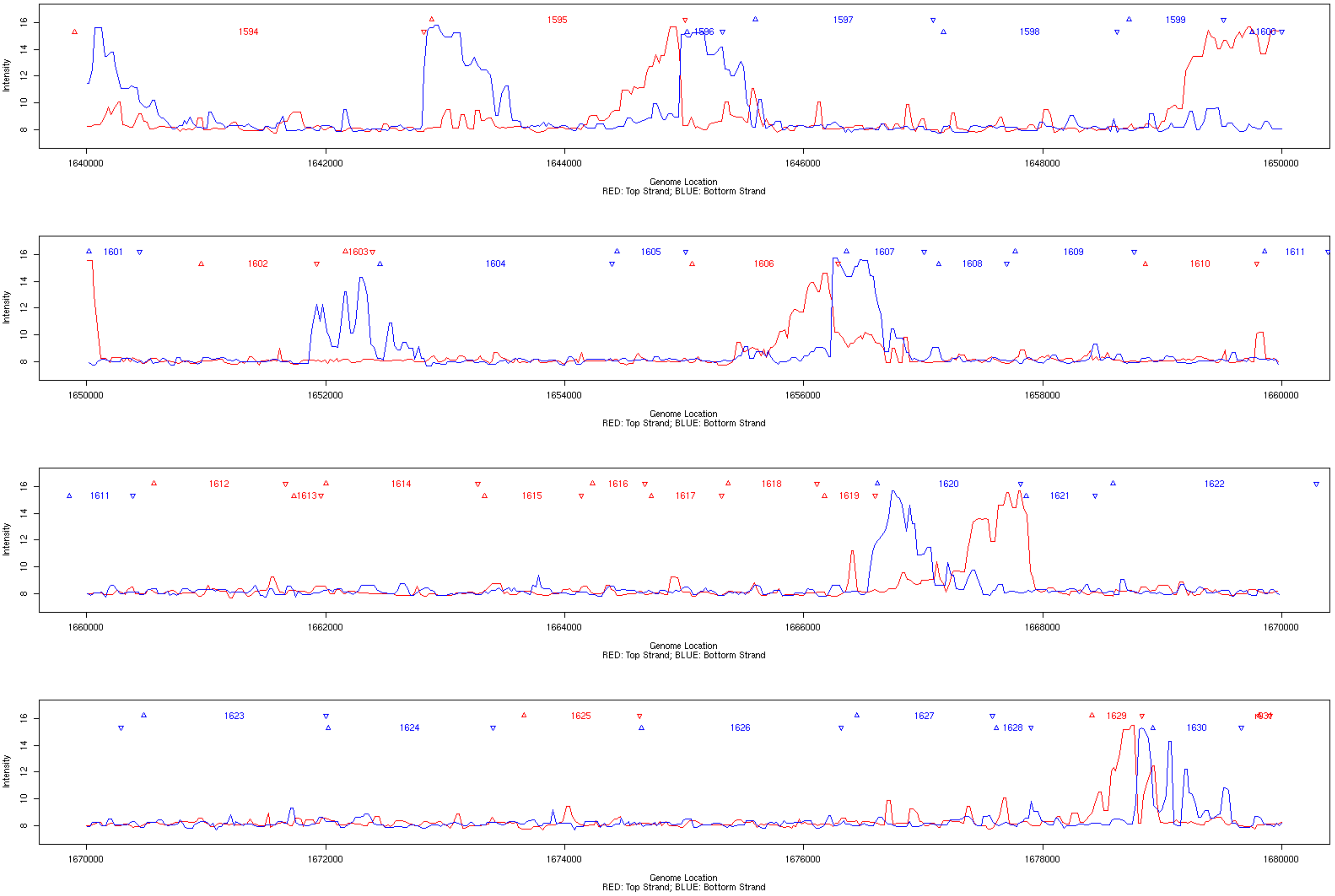
Intensity
Genome Location
RED: Top Strand; BLUE: Bottom Strand
1594
1595
1596
1597
1598
1599
1600
1601
1602
1603
1604
1605
1606
1607
1608
1609
1610
1611
1612
1613
1614
1615
1616
1617
1618
1619
1620
1621
1622
1623
1624
1625
1626
1627
1628
1629
1630

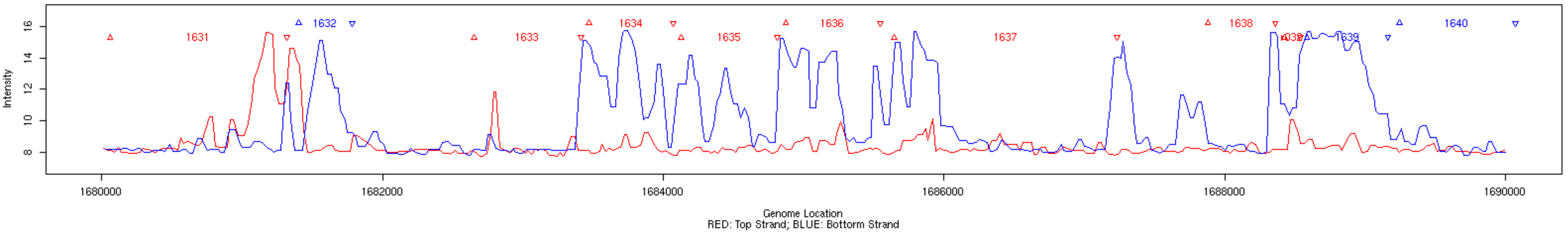
Intensity
Genome Location
RED: Top Strand; BLUE: Bottorm Strand

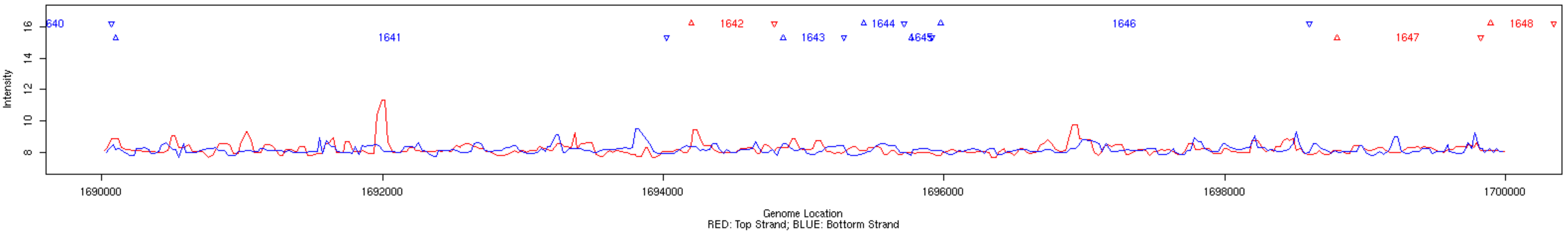
Intensity
Genome Location
RED: Top Strand; BLUE: Bottorm Strand

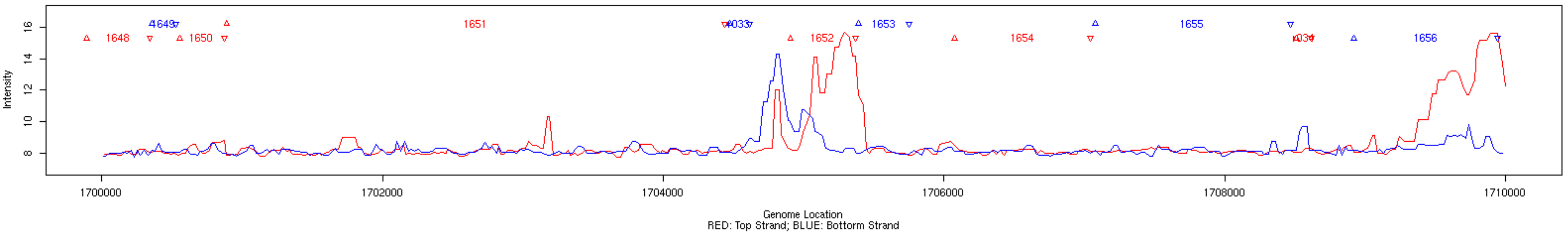
Intensity
Genome Location
RED: Top Strand; BLUE: Bottorm Strand

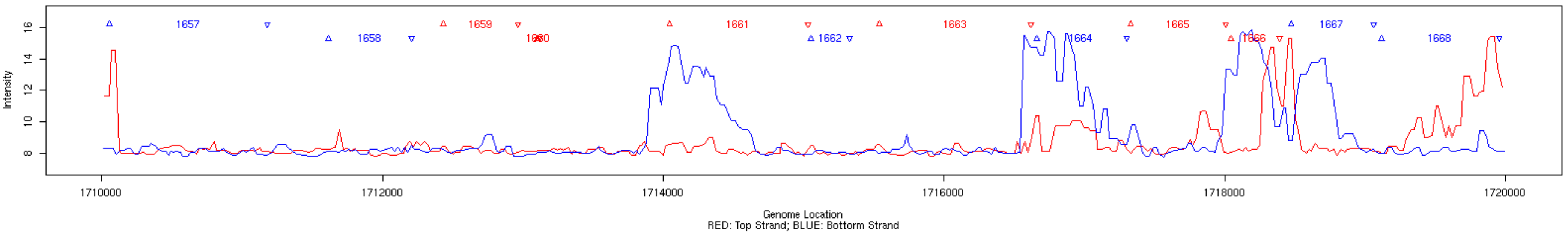
Intensity
Genome Location
RED: Top Strand; BLUE: Bottorm Strand

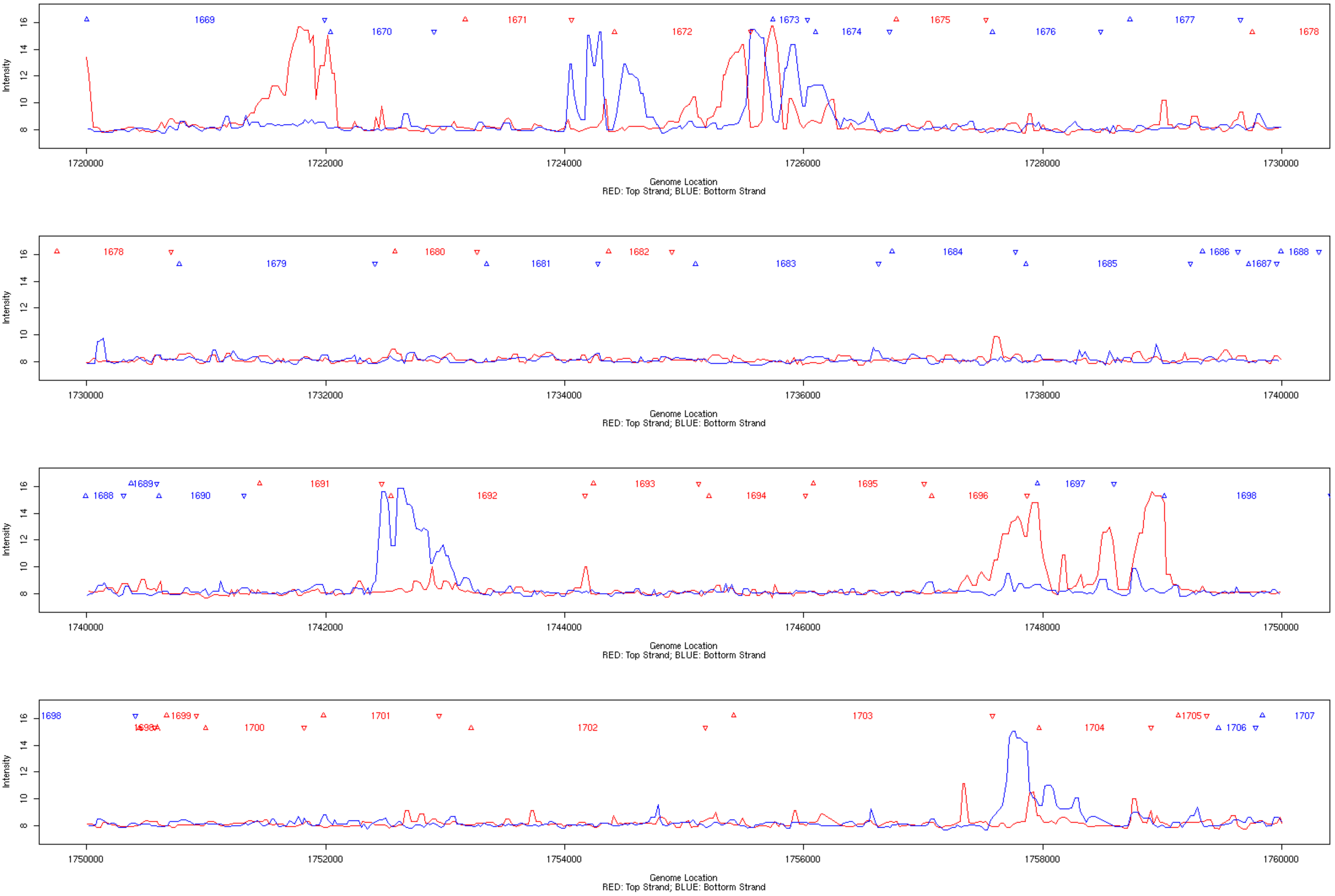
Intensity
Genome Location
RED: Top Strand; BLUE: Bottom Strand
1669
1670
1671
1672
1673
1674
1675
1676
1677
1678
1679
1680
1681
1682
1683
1684
1685
1686
1687
1688
1689
1690
1691
1692
1693
1694
1695
1696
1697
1698
1699
1700
1701
1702
1703
1704
1705
1706
1707

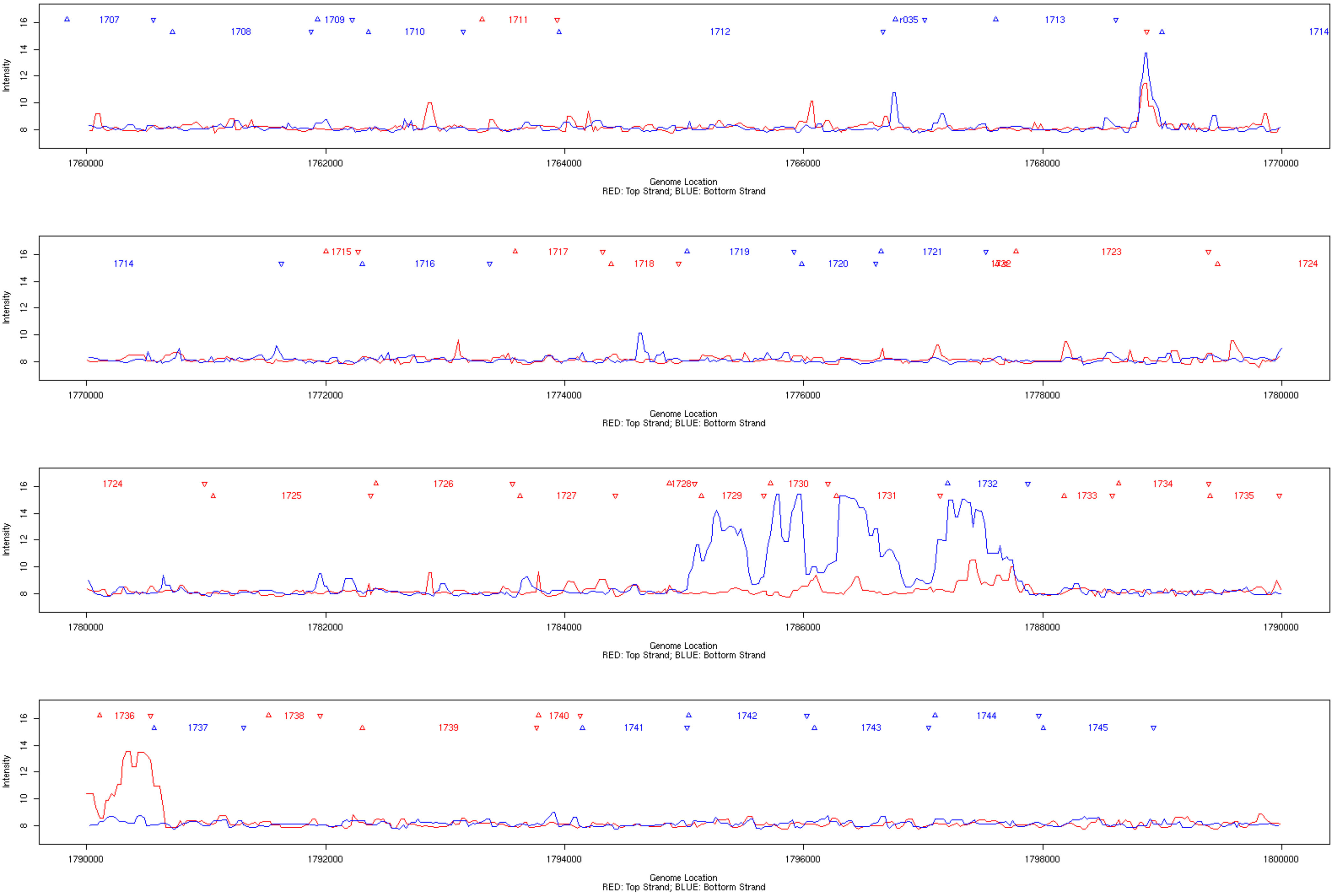
Intensity
1707
1708
1709
1710
1711
1712
r035
1713
1714
1760000
1762000
1764000
1766000
1768000
1770000
Genome Location
RED: Top Strand; BLUE: Bottom Strand
Intensity
1714
1715
1716
1717
1718
1719
1720
1721
1723
1724
1770000
1772000
1774000
1776000
1778000
1780000
Genome Location
RED: Top Strand; BLUE: Bottom Strand
Intensity
1724
1725
1726
1727
1728
1729
1730
1731
1732
1733
1734
1735
1780000
1782000
1784000
1786000
1788000
1790000
Genome Location
RED: Top Strand; BLUE: Bottom Strand
Intensity
1736
1737
1738
1739
1740
1741
1742
1743
1744
1745
1790000
1792000
1794000
1796000
1798000
1800000
Genome Location
RED: Top Strand; BLUE: Bottom Strand

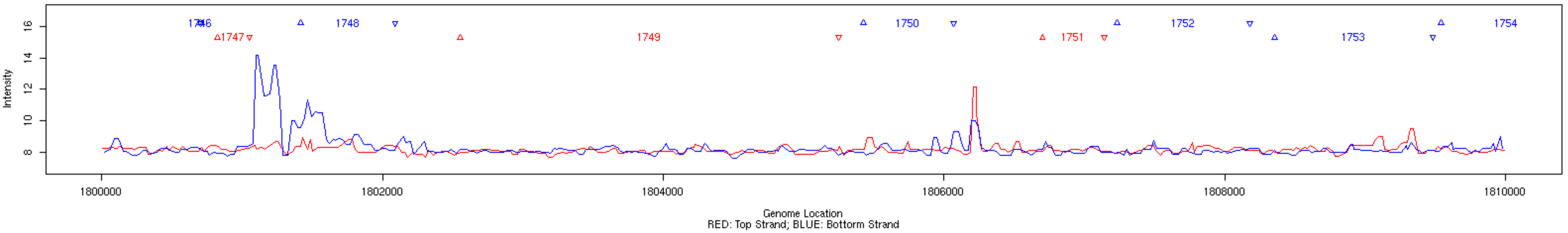
1747
1748
1749
1750
1751
1752
1753
1754
Intensity
Genome Location
RED: Top Strand; BLUE: Bottorm Strand

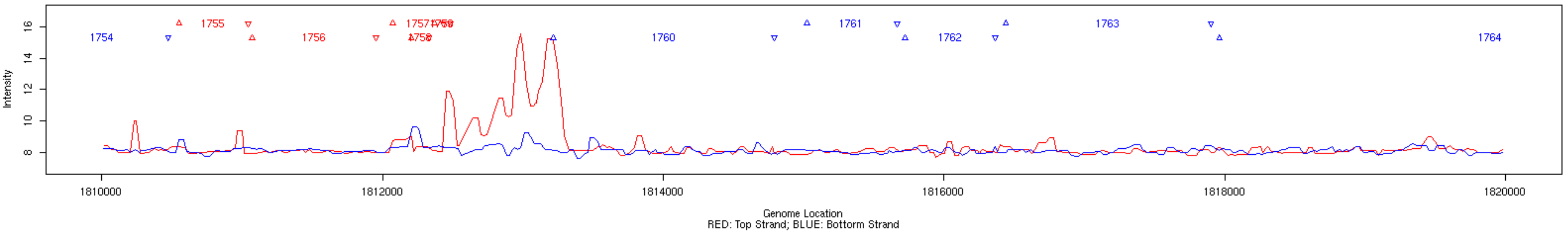
1754
1755
1756
1760
1761
1762
1763
1764
Intensity
Genome Location
RED: Top Strand; BLUE: Bottorm Strand

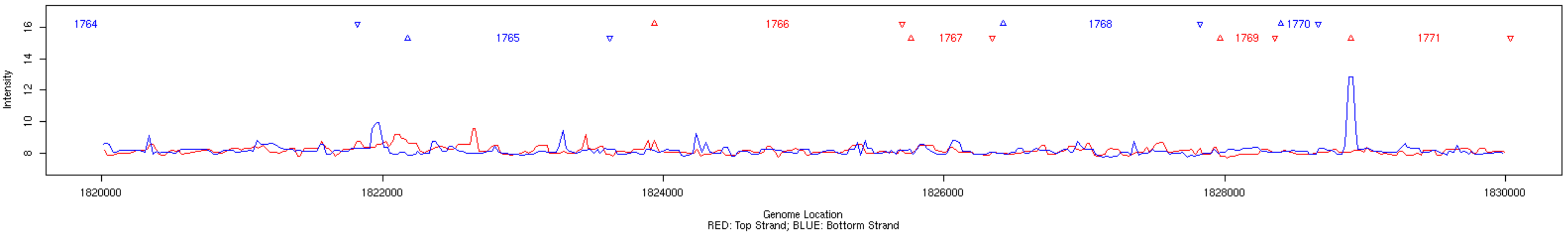
1764
1765
1766
1767
1768
1769
1770
1771
Intensity
Genome Location
RED: Top Strand; BLUE: Bottorm Strand

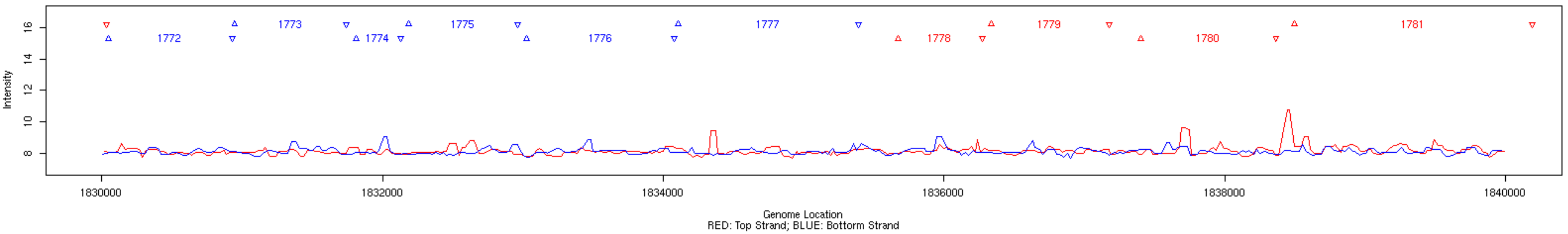
1772
1773
1774
1775
1776
1777
1778
1779
1780
1781
Intensity
Genome Location
RED: Top Strand; BLUE: Bottorm Strand

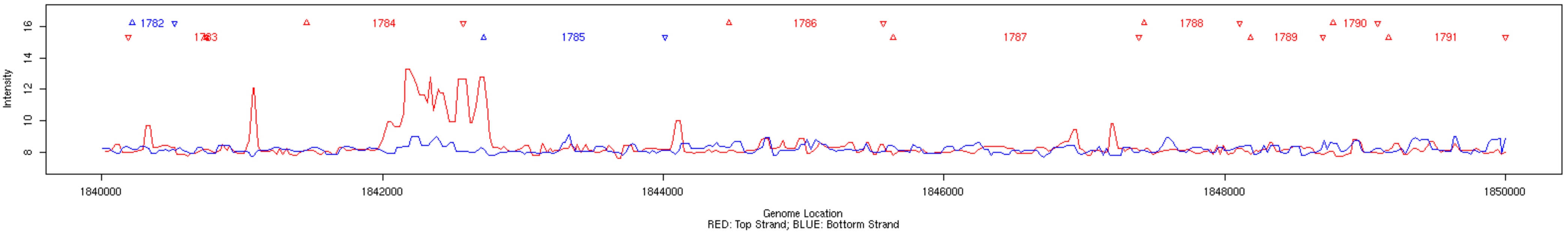
Intensity
1782
1784
1785
1786
1787
1788
1789
1790
1791
1840000
1842000
1844000
1846000
1848000
1850000
Genome Location
RED: Top Strand; BLUE: Bottorm Strand

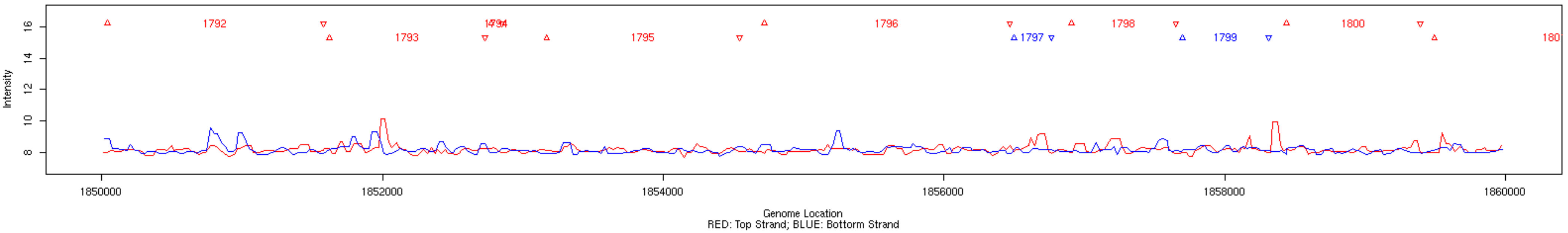
Intensity
1792
1793
1795
1796
1797
1798
1799
1800
1850000
1852000
1854000
1856000
1858000
1860000
Genome Location
RED: Top Strand; BLUE: Bottorm Strand

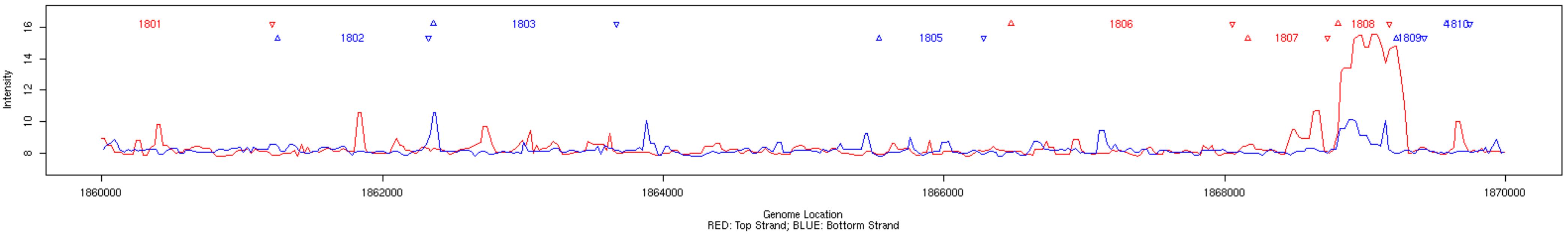
Intensity
1801
1802
1803
1805
1806
1807
1808
1809
1860000
1862000
1864000
1866000
1868000
1870000
Genome Location
RED: Top Strand; BLUE: Bottorm Strand

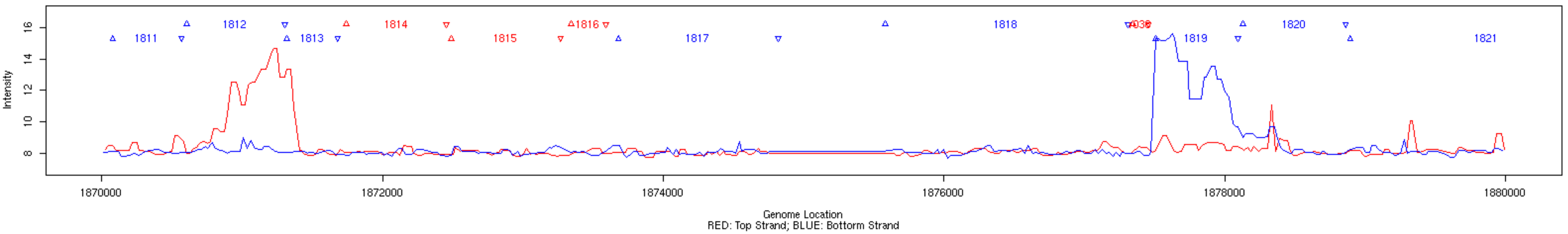
Intensity
1811
1812
1813
1814
1815
1816
1817
1818
1819
1820
1821
1870000
1872000
1874000
1876000
1878000
1880000
Genome Location
RED: Top Strand; BLUE: Bottorm Strand

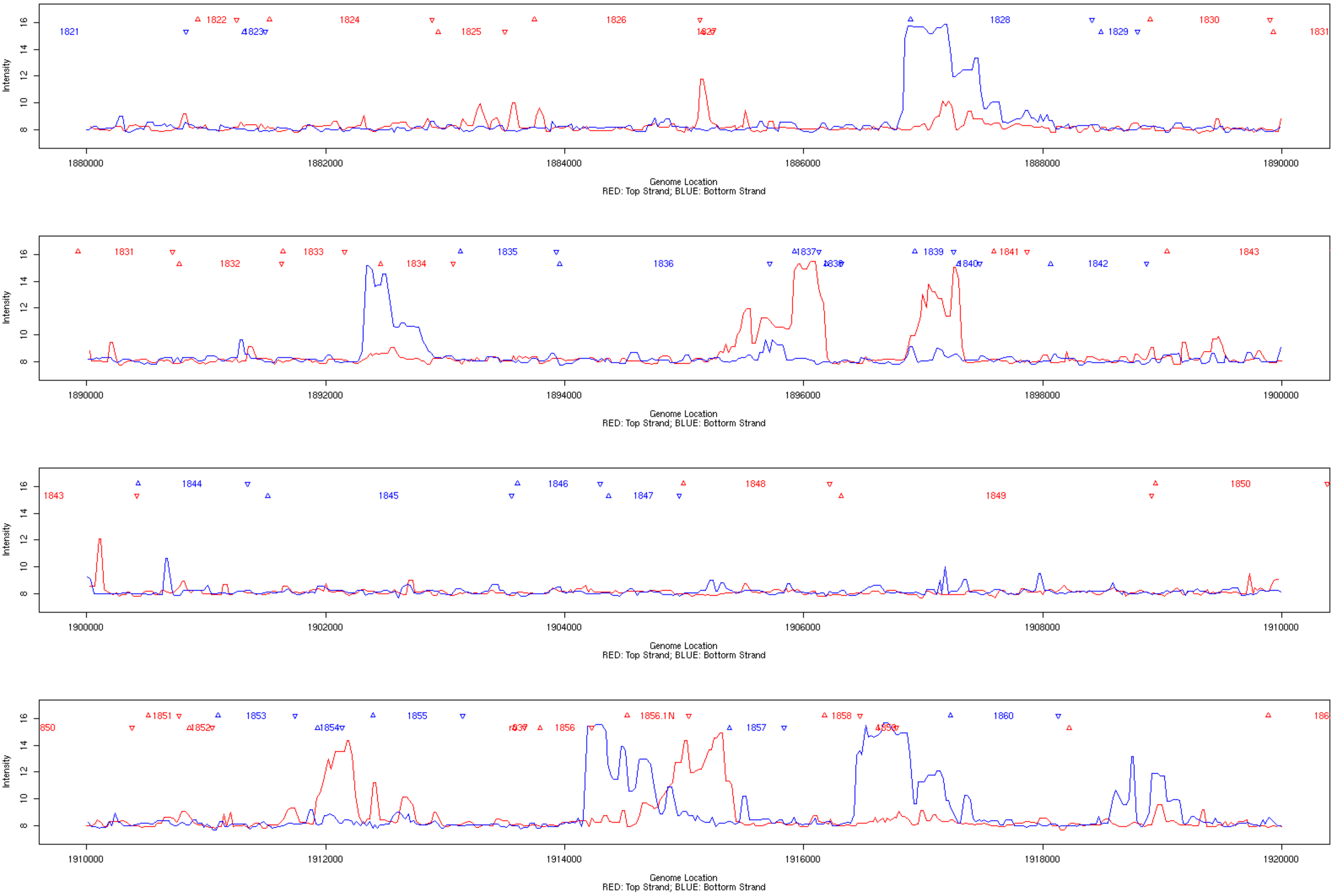
Intensity
1821
1822
1823
1824
1825
1826
1827
1828
1829
1830
1831
1880000
1882000
1884000
1886000
1888000
1890000
Genome Location
RED: Top Strand; BLUE: Bottorm Strand
Intensity
1831
1832
1833
1834
1835
1836
1837
1838
1839
1840
1841
1842
1843
1890000
1892000
1894000
1896000
1898000
1900000
Genome Location
RED: Top Strand; BLUE: Bottorm Strand
Intensity
1843
1844
1845
1846
1847
1848
1849
1850
1900000
1902000
1904000
1906000
1908000
1910000
Genome Location
RED: Top Strand; BLUE: Bottorm Strand
Intensity
1851
1852
1853
1854
1855
1856
1856.1N
1857
1858
1860
1910000
1912000
1914000
1916000
1918000
1920000
Genome Location
RED: Top Strand; BLUE: Bottorm Strand

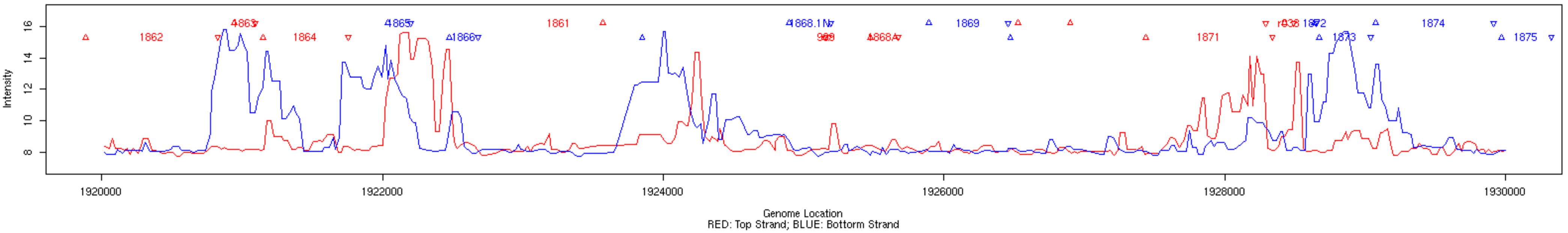
Intensity
1862
1864
1861
1871
1869
1874
1875
Genome Location
RED: Top Strand; BLUE: Bottorm Strand

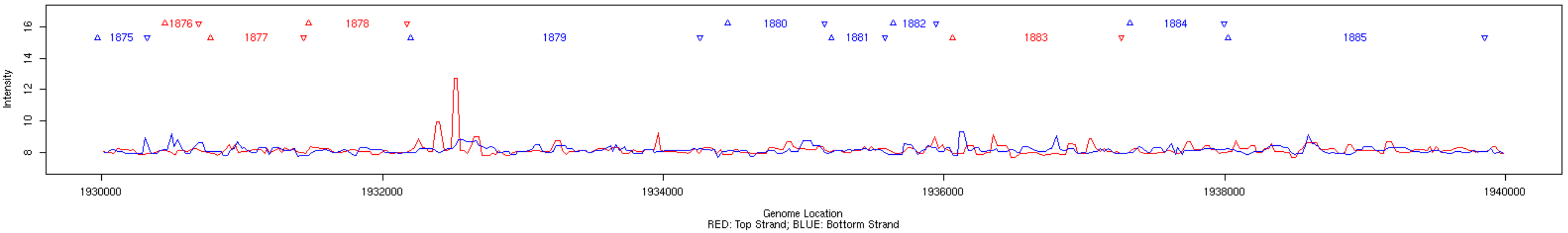
Intensity
1875
1876
1877
1878
1879
1880
1881
1882
1883
1884
1885
Genome Location
RED: Top Strand; BLUE: Bottorm Strand

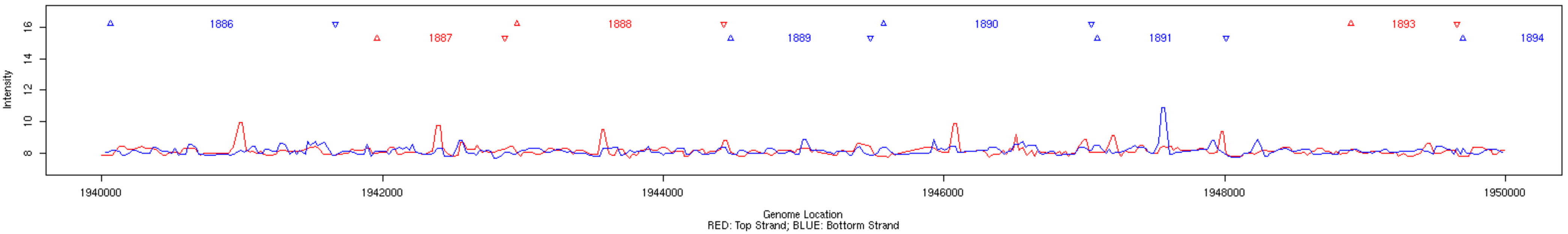
Intensity
1886
1887
1888
1889
1890
1891
1893
1894
Genome Location
RED: Top Strand; BLUE: Bottorm Strand

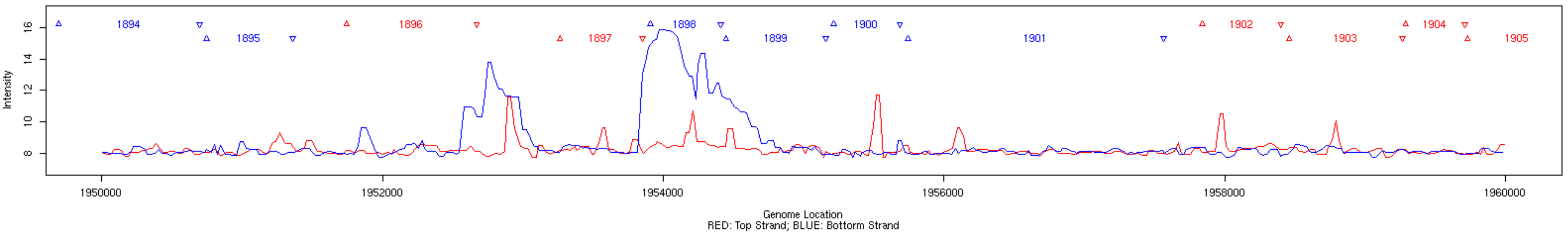
Intensity
1894
1895
1896
1897
1898
1899
1900
1901
1902
1903
1904
1905
Genome Location
RED: Top Strand; BLUE: Bottorm Strand

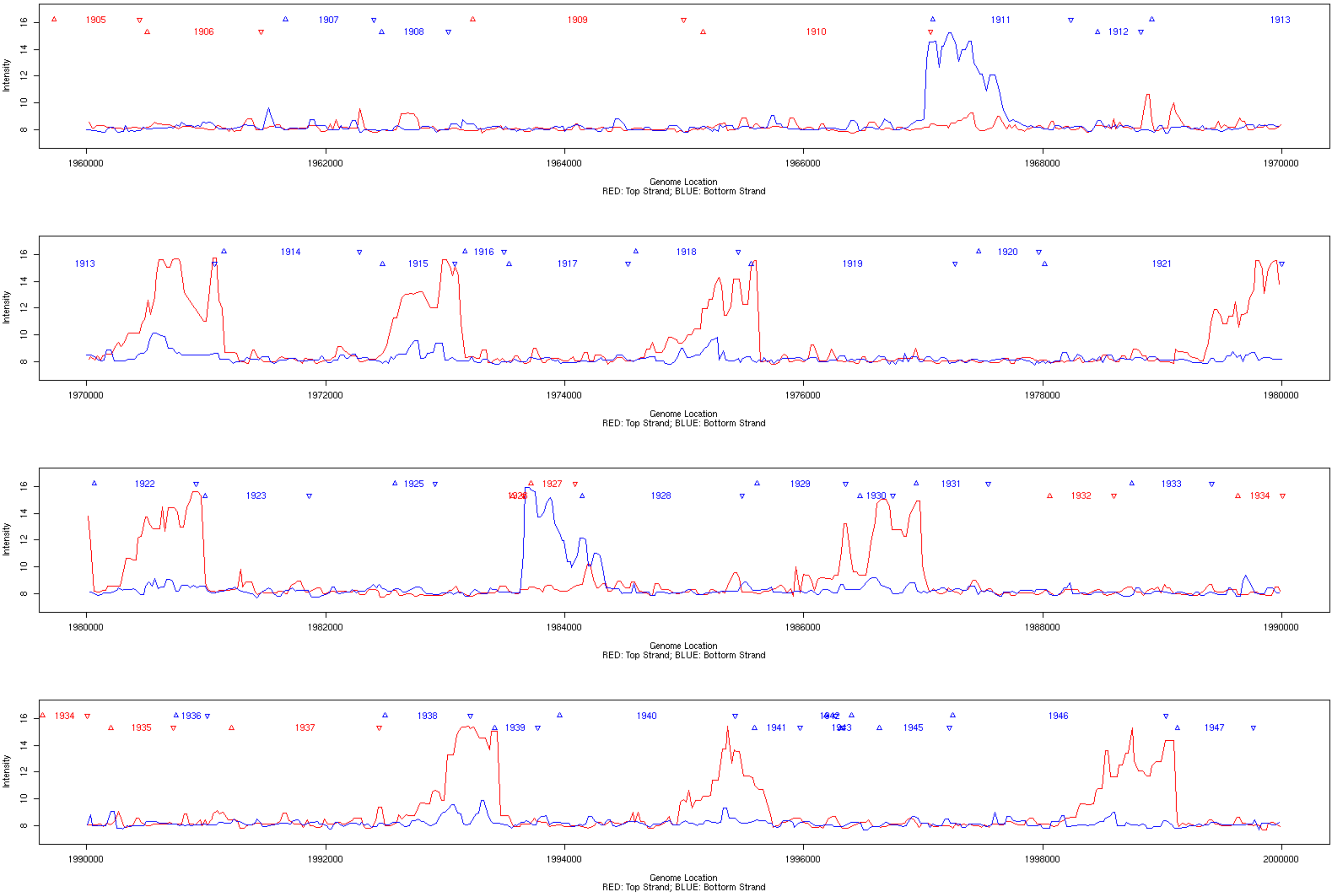

Intensity
1905
1906
1907
1908
1909
1910
1911
1912
1913
1960000
1962000
1964000
1966000
1968000
1970000
Genome Location
RED: Top Strand; BLUE: Bottom Strand
1913
1914
1915
1916
1917
1918
1919
1920
1921
1970000
1972000
1974000
1976000
1978000
1980000
Genome Location
RED: Top Strand; BLUE: Bottom Strand
1922
1923
1925
1927
1928
1929
1930
1931
1932
1933
1934
1980000
1982000
1984000
1986000
1988000
1990000
Genome Location
RED: Top Strand; BLUE: Bottom Strand
1934
1935
1936
1937
1938
1939
1940
1941
1945
1946
1947
1990000
1992000
1994000
1996000
1998000
2000000
Genome Location
RED: Top Strand; BLUE: Bottom Strand

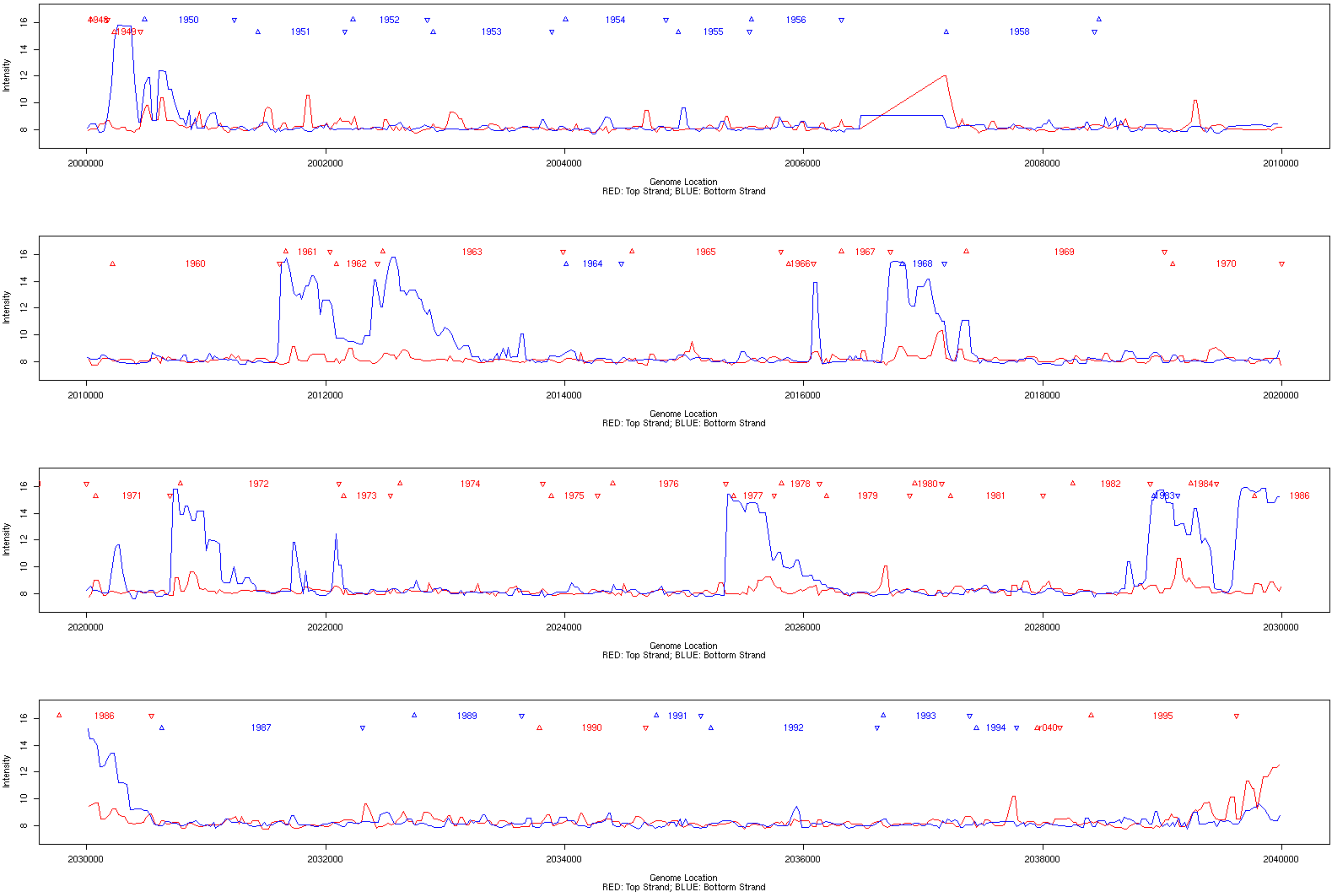
Intensity
Genome Location
RED: Top Strand; BLUE: Bottom Strand

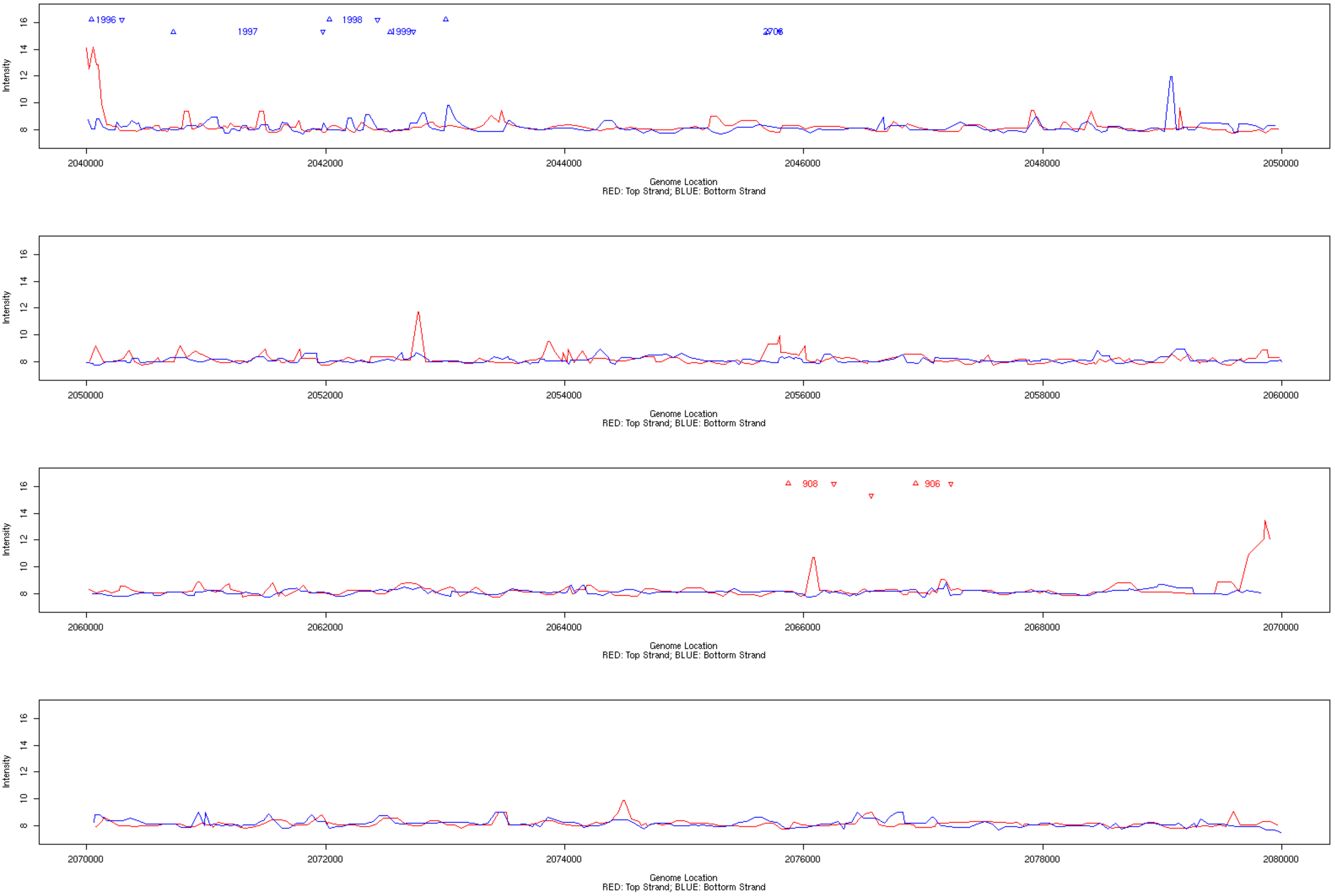
1996
1997
1998
1999
2706
908
906
Intensity
Genome Location
RED: Top Strand; BLUE: Bottom Strand

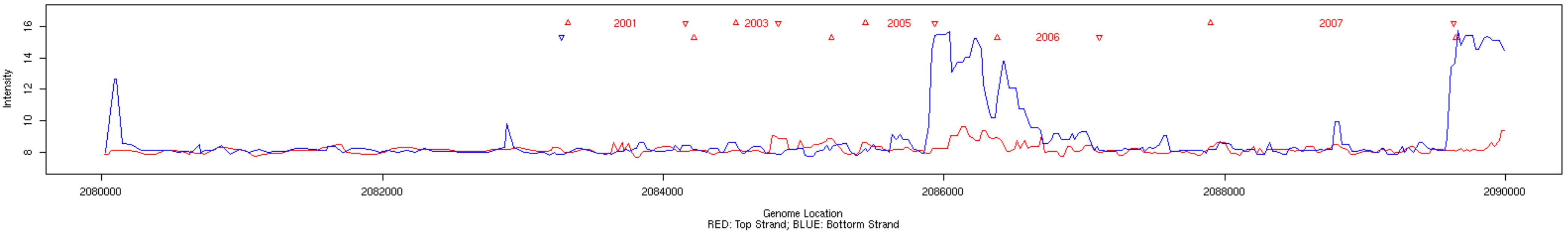

Intensity
16
14
12
10
8
2001
2003
2005
2006
2007
2080000
2082000
2084000
2086000
2088000
2090000
Genome Location
RED: Top Strand; BLUE: Bottorm Strand

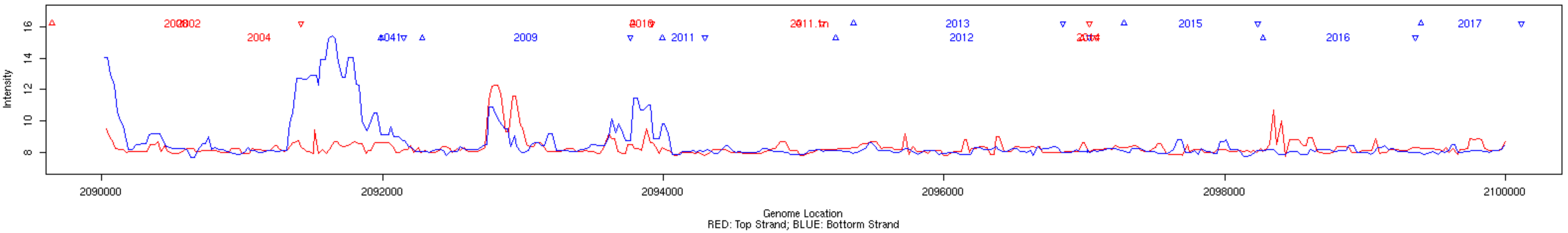

Intensity
16
14
12
10
8
2004
2009
2011
2013
2012
2015
2016
2017
2090000
2092000
2094000
2096000
2098000
2100000
Genome Location
RED: Top Strand; BLUE: Bottorm Strand

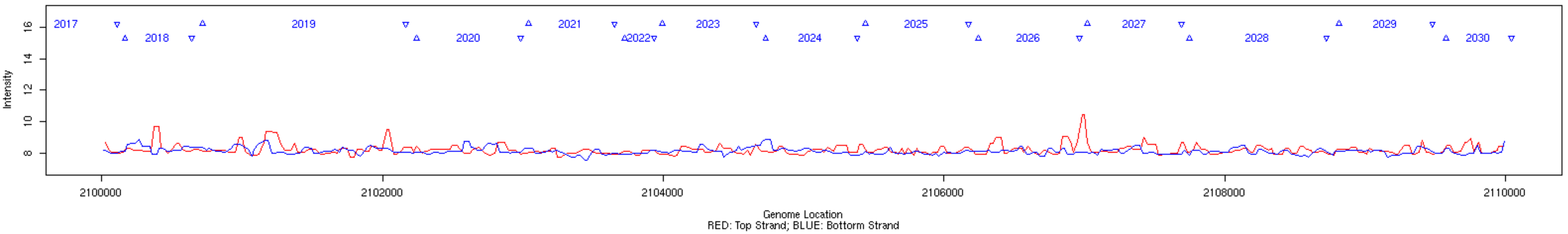

Intensity
16
14
12
10
8
2017
2018
2019
2020
2021
2023
2024
2025
2026
2027
2028
2029
2030
2100000
2102000
2104000
2106000
2108000
2110000
Genome Location
RED: Top Strand; BLUE: Bottorm Strand

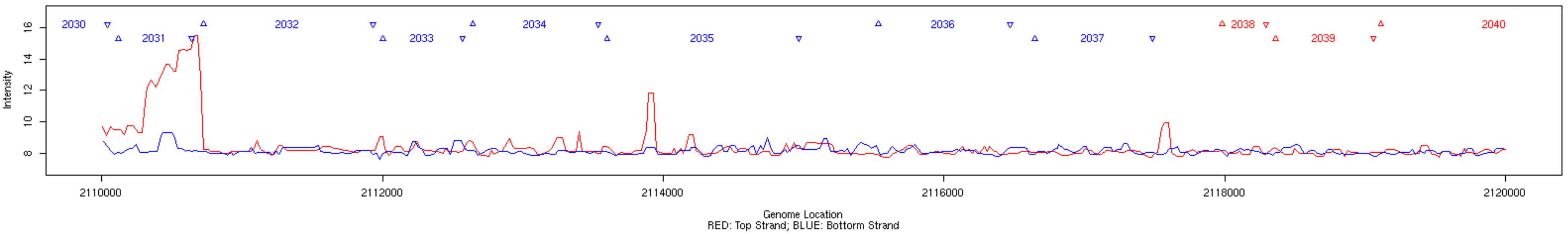

Intensity
16
14
12
10
8
2030
2031
2032
2033
2034
2035
2036
2037
2038
2039
2040
2110000
2112000
2114000
2116000
2118000
2120000
Genome Location
RED: Top Strand; BLUE: Bottorm Strand

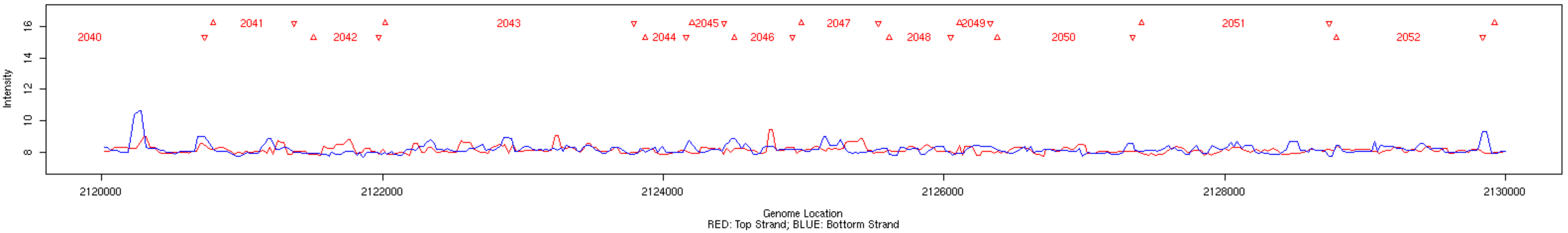
2040
2041
2042
2043
2044
2045
2046
2047
2048
2049
2050
2051
2052
Intensity
8
10
12
14
16
2120000
2122000
2124000
2126000
2128000
2130000
Genome Location
RED: Top Strand; BLUE: Bottorm Strand

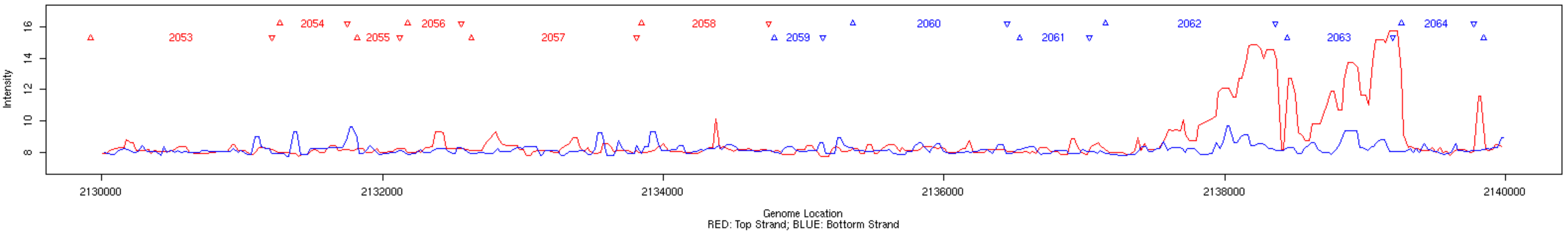
2053
2054
2055
2056
2057
2058
2059
2060
2061
2062
2063
2064
Intensity
8
10
12
14
16
2130000
2132000
2134000
2136000
2138000
2140000
Genome Location
RED: Top Strand; BLUE: Bottorm Strand

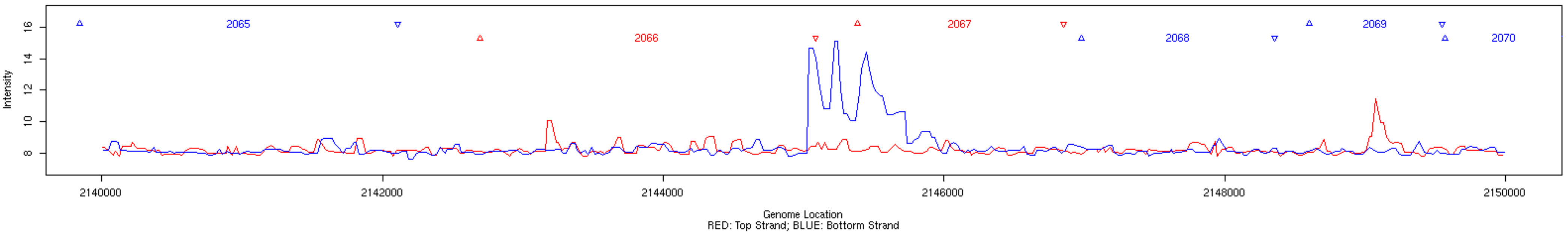
2065
2066
2067
2068
2069
2070
Intensity
8
10
12
14
16
2140000
2142000
2144000
2146000
2148000
2150000
Genome Location
RED: Top Strand; BLUE: Bottorm Strand

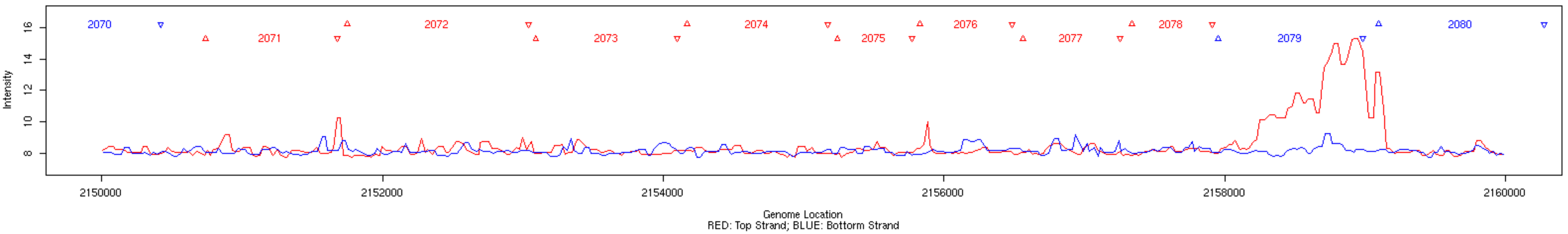
2070
2071
2072
2073
2074
2075
2076
2077
2078
2079
2080
Intensity
8
10
12
14
16
2150000
2152000
2154000
2156000
2158000
2160000
Genome Location
RED: Top Strand; BLUE: Bottorm Strand

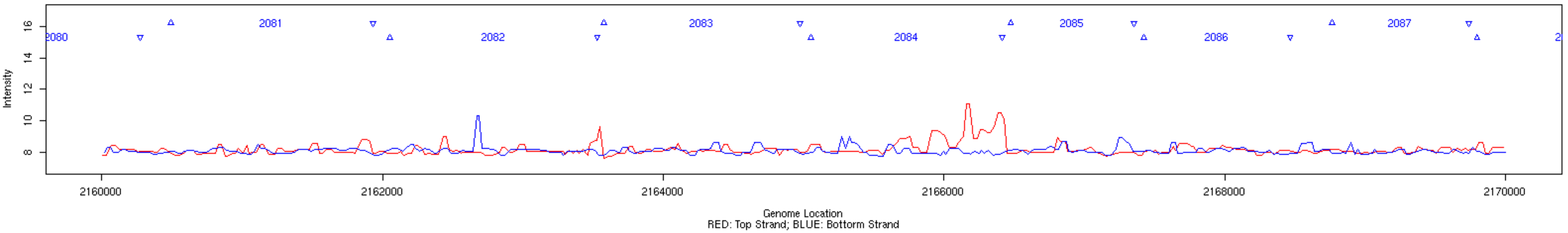
Intensity
2080
2081
2082
2083
2084
2085
2086
2087
2160000
2162000
2164000
2166000
2168000
2170000
Genome Location
RED: Top Strand; BLUE: Bottorm Strand

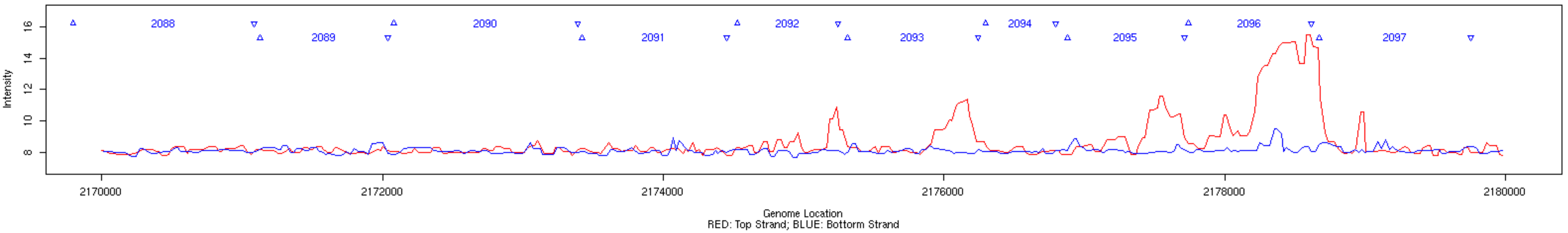
Intensity
2088
2089
2090
2091
2092
2093
2094
2095
2096
2097
2170000
2172000
2174000
2176000
2178000
2180000
Genome Location
RED: Top Strand; BLUE: Bottorm Strand

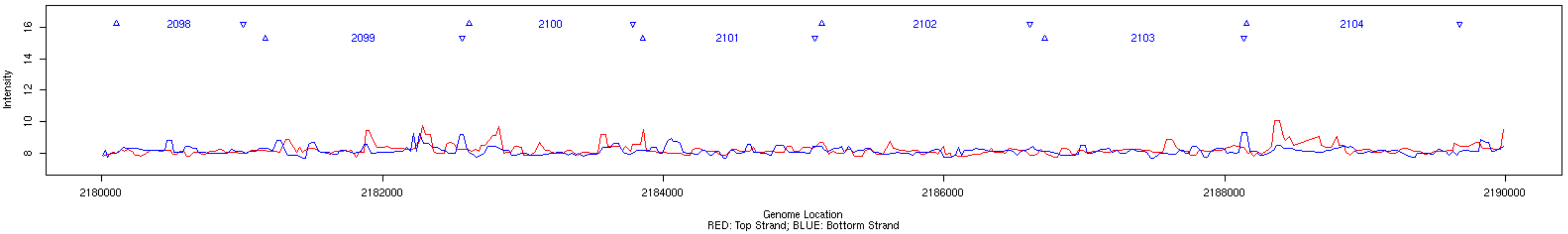
Intensity
2098
2099
2100
2101
2102
2103
2104
2180000
2182000
2184000
2186000
2188000
2190000
Genome Location
RED: Top Strand; BLUE: Bottorm Strand

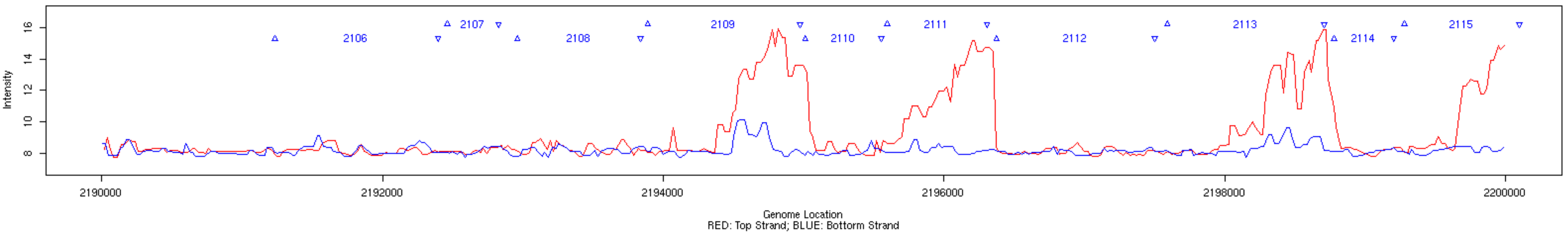
Intensity
2106
2107
2108
2109
2110
2111
2112
2113
2114
2115
2190000
2192000
2194000
2196000
2198000
2200000
Genome Location
RED: Top Strand; BLUE: Bottorm Strand

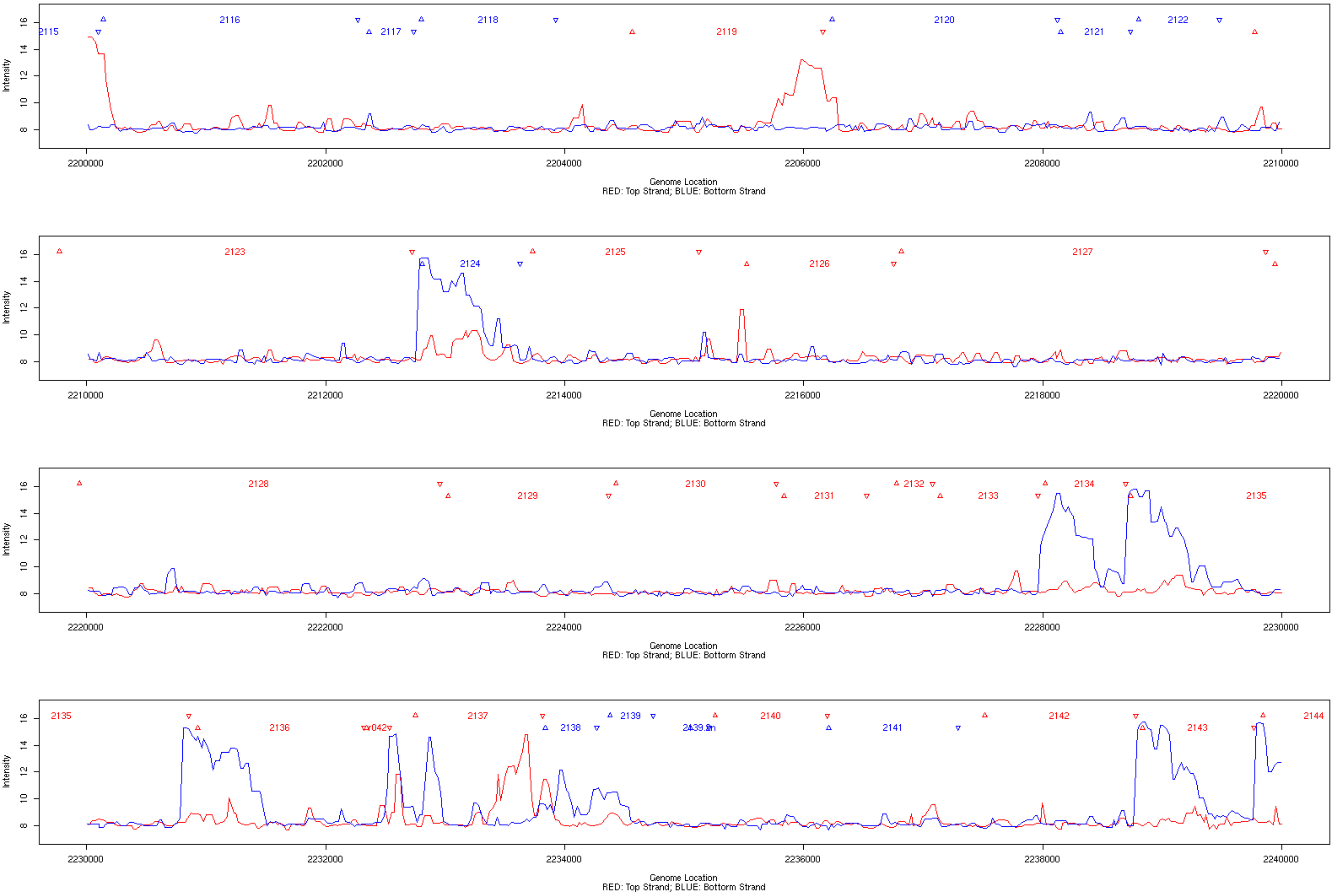
Intensity
Genome Location
RED: Top Strand; BLUE: Bottorm Strand
2115
2116
2117
2118
2119
2120
2121
2122
2123
2124
2125
2126
2127
2128
2129
2130
2131
2132
2133
2134
2135
2136
2137
2138
2139
2140
2141
2142
2143
2144
2200000
2202000
2204000
2206000
2208000
2210000
2212000
2214000
2216000
2218000
2220000
2222000
2224000
2226000
2228000
2230000
2232000
2234000
2236000
2238000
2240000

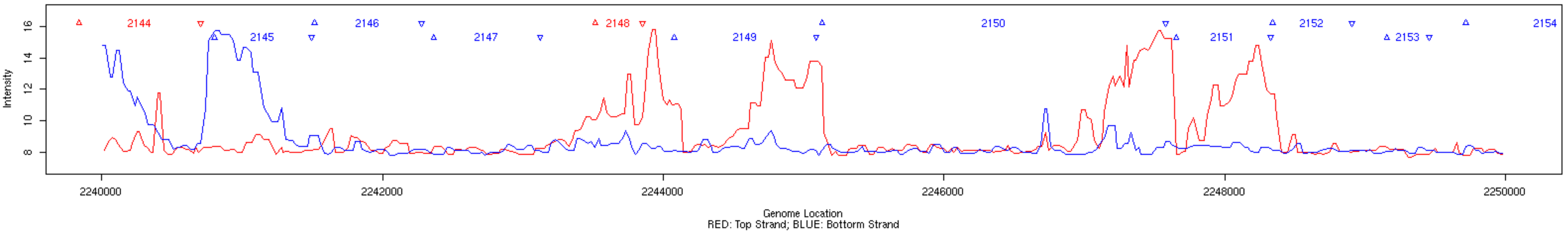
Intensity
2144
2145
2146
2147
2148
2149
2150
2151
2152
2153
2154
2240000
2242000
2244000
2246000
2248000
2250000
Genome Location
RED: Top Strand; BLUE: Bottorm Strand

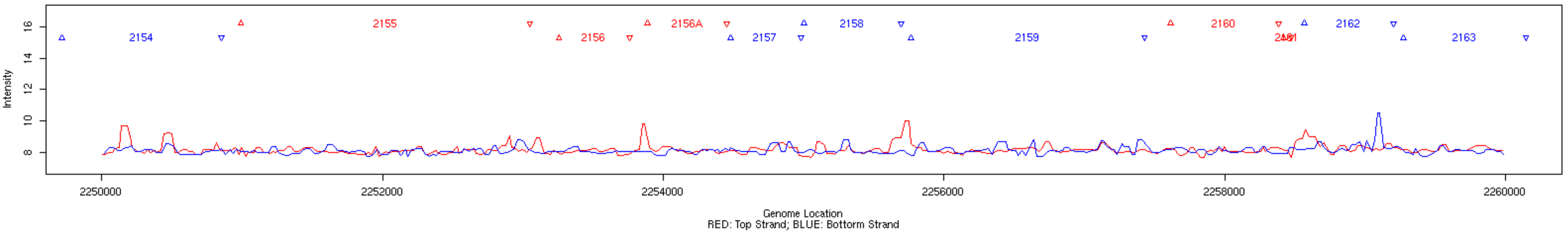
Intensity
2154
2155
2156
2156A
2157
2158
2159
2160
2162
2163
2250000
2252000
2254000
2256000
2258000
2260000
Genome Location
RED: Top Strand; BLUE: Bottorm Strand

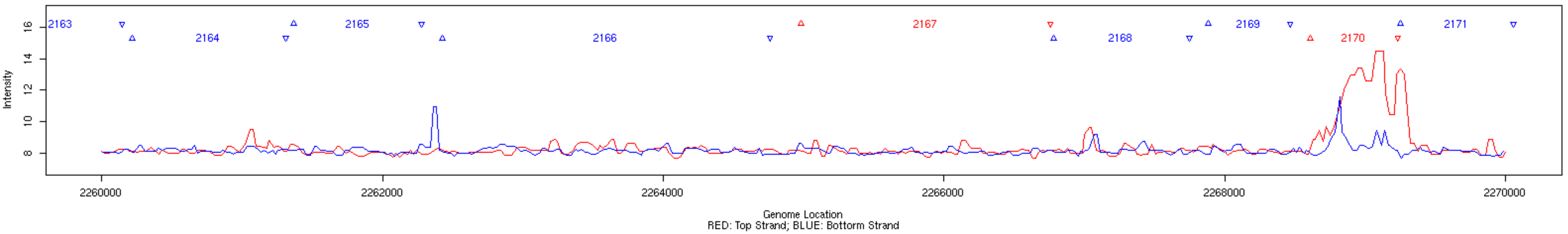
Intensity
2163
2164
2165
2166
2167
2168
2169
2170
2171
2260000
2262000
2264000
2266000
2268000
2270000
Genome Location
RED: Top Strand; BLUE: Bottorm Strand

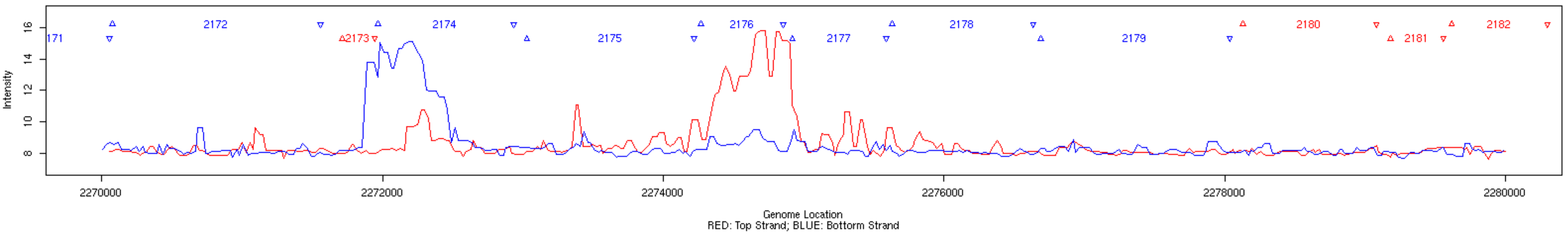
Intensity
171
2172
2173
2174
2175
2176
2177
2178
2179
2180
2181
2182
2270000
2272000
2274000
2276000
2278000
2280000
Genome Location
RED: Top Strand; BLUE: Bottorm Strand

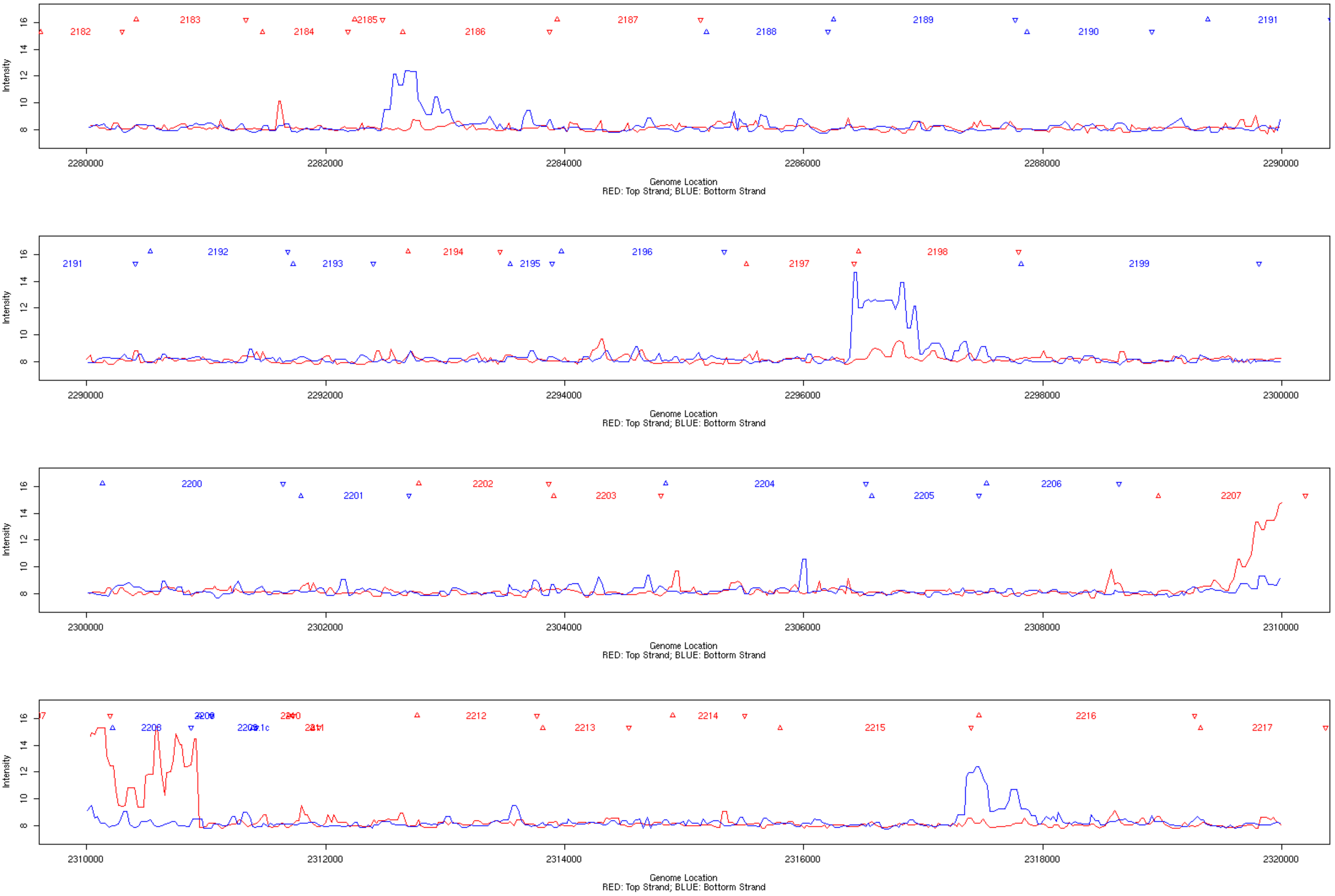
Intensity
Genome Location
RED: Top Strand; BLUE: Bottorm Strand
2182
2183
2184
2185
2186
2187
2188
2189
2190
2191
2192
2193
2194
2195
2196
2197
2198
2199
2200
2201
2202
2203
2204
2205
2206
2207
2208
2212
2213
2214
2215
2216
2217

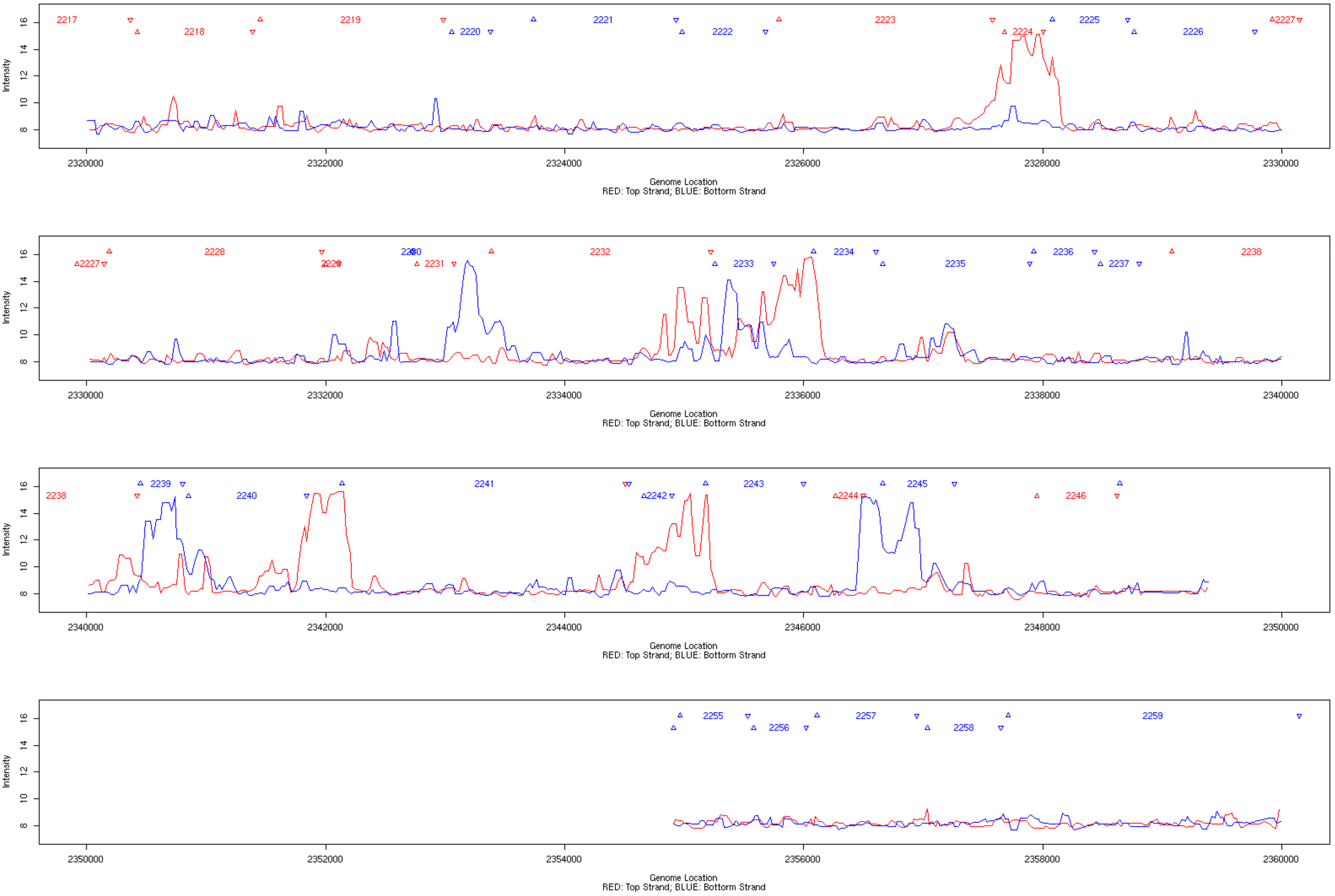
Intensity
Genome Location
RED: Top Strand; BLUE: Bottorm Strand
2217
2218
2219
2220
2221
2222
2223
2224
2225
2226
2227
2228
2231
2232
2233
2234
2235
2236
2237
2238
2239
2240
2241
2242
2243
2244
2245
2246
2255
2256
2257
2258
2259

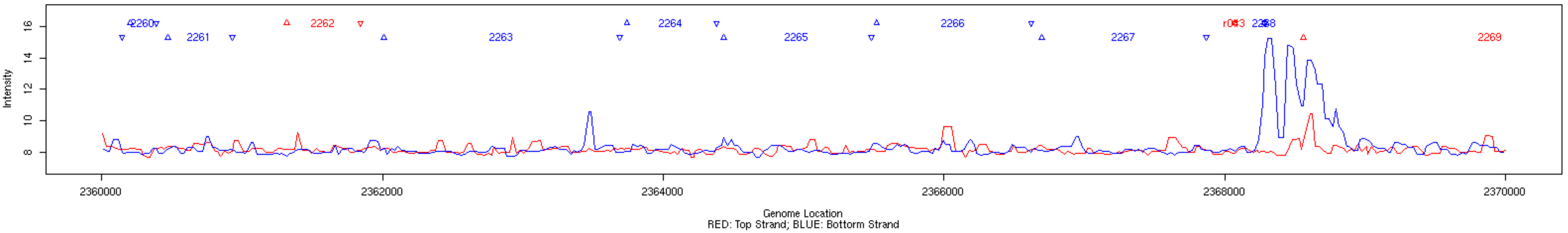

Intensity
2360000
2362000
2364000
2366000
2368000
2370000
2261
2262
2263
2264
2265
2266
2267
2269
Genome Location
RED: Top Strand; BLUE: Bottorm Strand

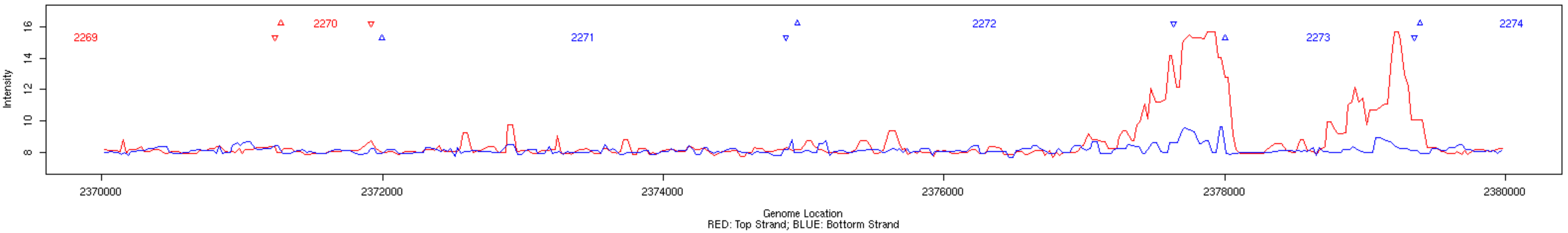

Intensity
2370000
2372000
2374000
2376000
2378000
2380000
2269
2270
2271
2272
2273
2274
Genome Location
RED: Top Strand; BLUE: Bottorm Strand

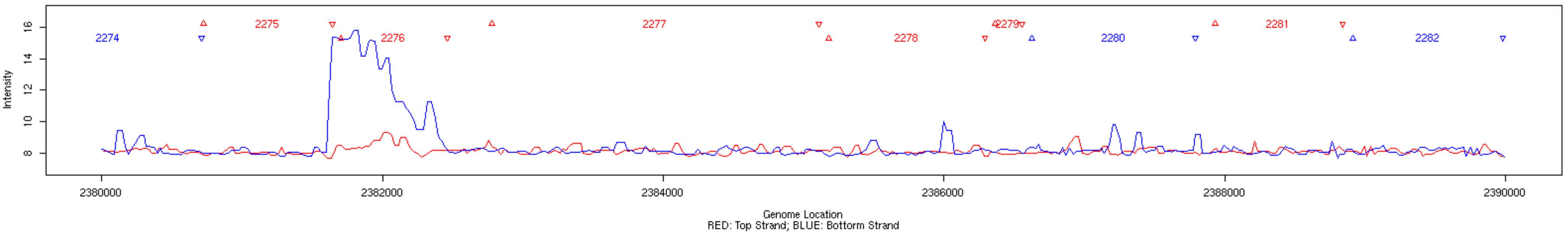

Intensity
2380000
2382000
2384000
2386000
2388000
2390000
2274
2275
2276
2277
2278
2280
2281
2282
Genome Location
RED: Top Strand; BLUE: Bottorm Strand

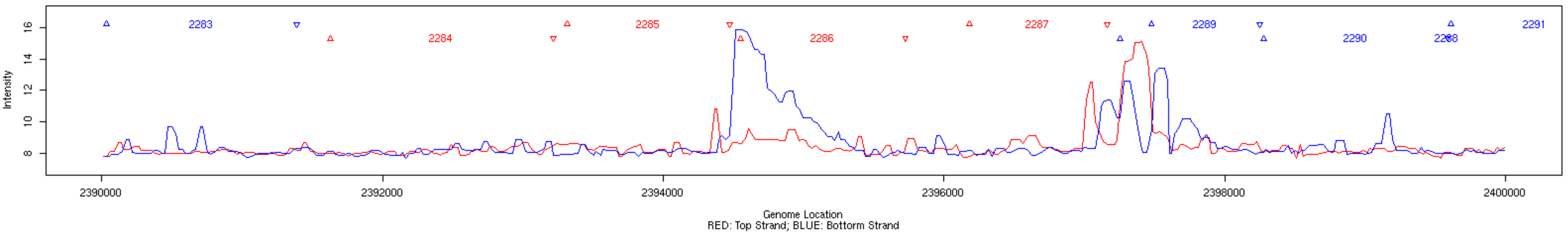

Intensity
2390000
2392000
2394000
2396000
2398000
2400000
2283
2284
2285
2286
2287
2289
2290
2291
Genome Location
RED: Top Strand; BLUE: Bottorm Strand

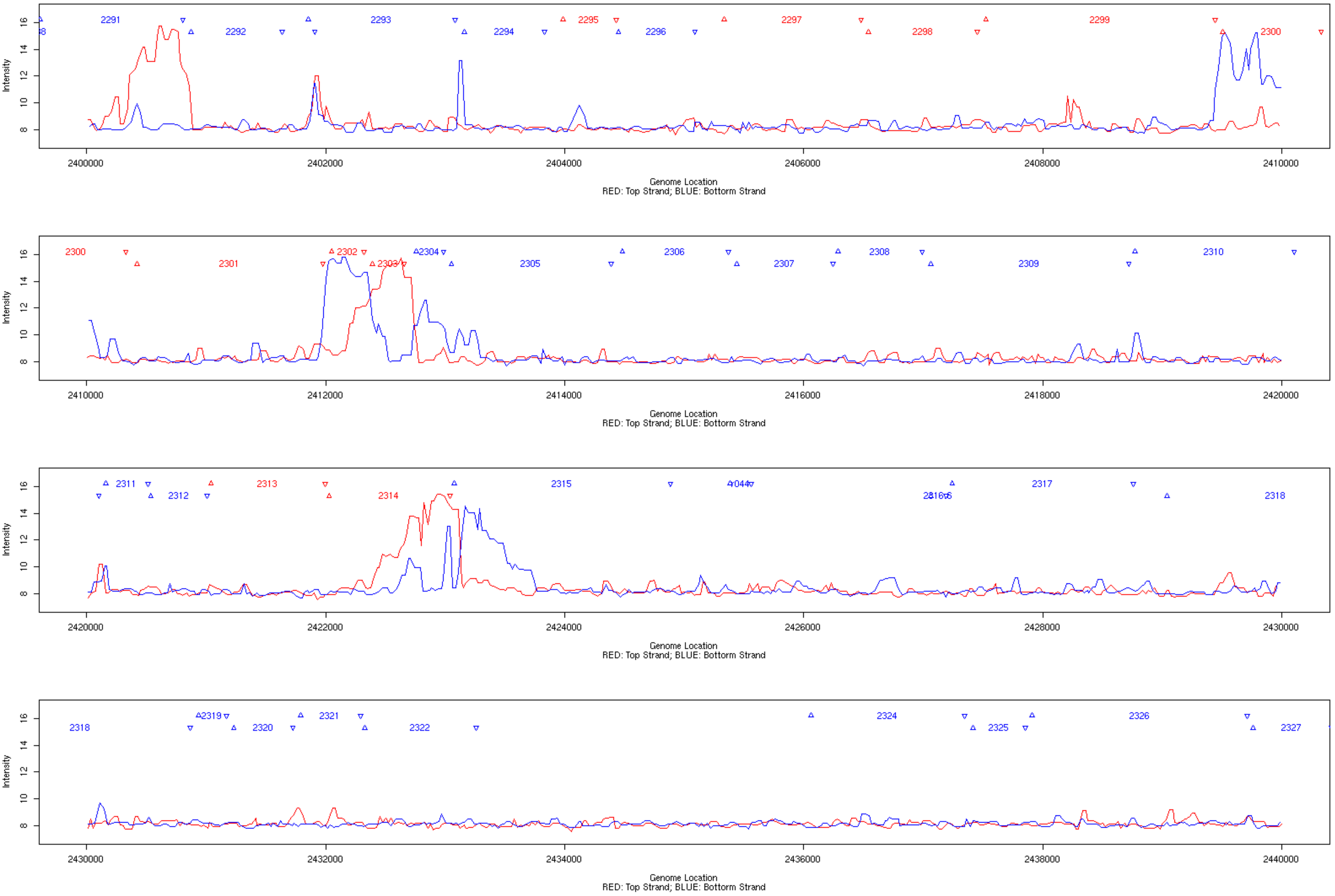
Intensity
Genome Location
RED: Top Strand; BLUE: Bottom Strand
2291
2292
2293
2294
2295
2296
2297
2298
2299
2300
2301
2302
2303
2304
2305
2306
2307
2308
2309
2310
2311
2312
2313
2314
2315
2317
2318
2319
2320
2321
2322
2324
2325
2326
2327

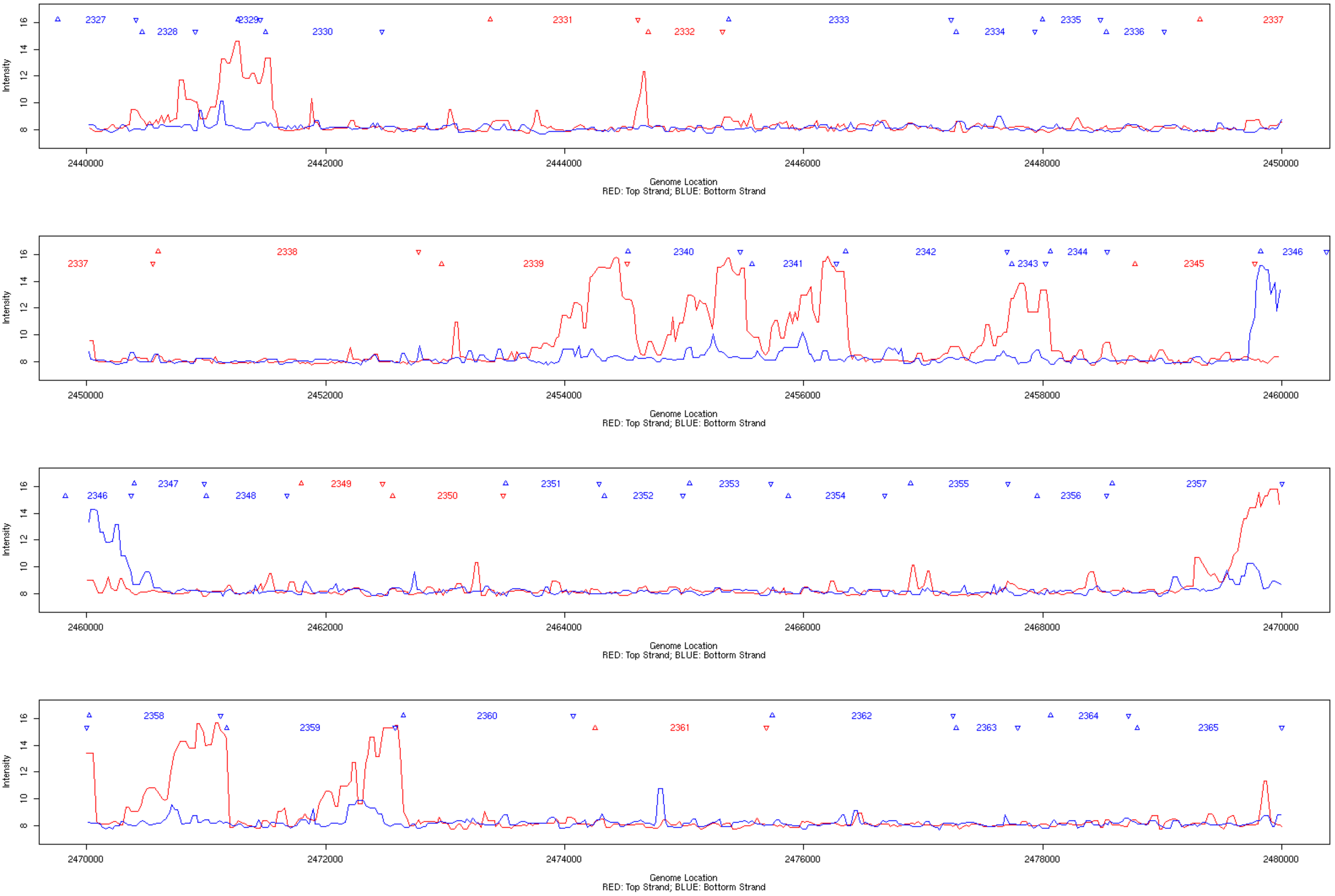
Intensity
2327
2328
2329
2330
2331
2332
2333
2334
2335
2336
2337
2440000
2442000
2444000
2446000
2448000
2450000
Genome Location
RED: Top Strand; BLUE: Bottorm Strand
2337
2338
2339
2340
2341
2342
2343
2344
2345
2346
2450000
2452000
2454000
2456000
2458000
2460000
Genome Location
RED: Top Strand; BLUE: Bottorm Strand
2346
2347
2348
2349
2350
2351
2352
2353
2354
2355
2356
2357
2460000
2462000
2464000
2466000
2468000
2470000
Genome Location
RED: Top Strand; BLUE: Bottorm Strand
2358
2359
2360
2361
2362
2363
2364
2365
2470000
2472000
2474000
2476000
2478000
2480000
Genome Location
RED: Top Strand; BLUE: Bottorm Strand

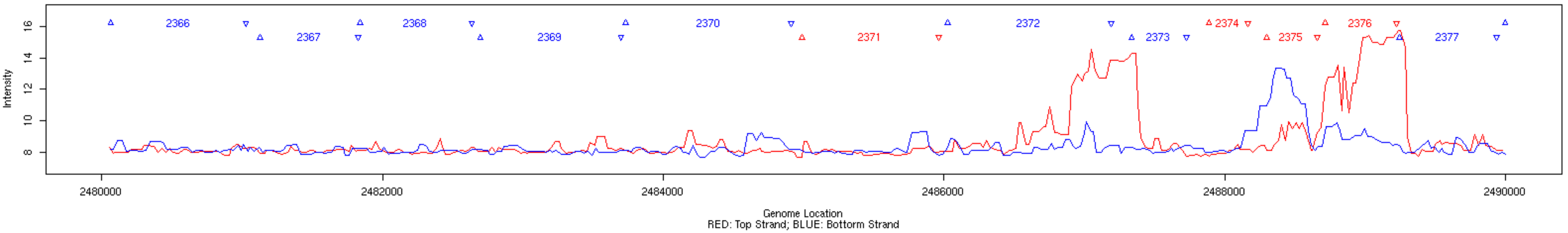
Intensity
Genome Location
RED: Top Strand; BLUE: Bottorm Strand

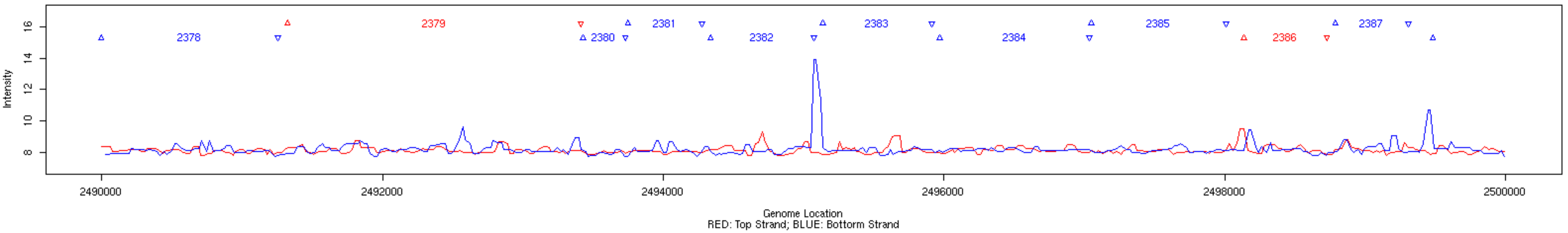
Intensity
Genome Location
RED: Top Strand; BLUE: Bottorm Strand

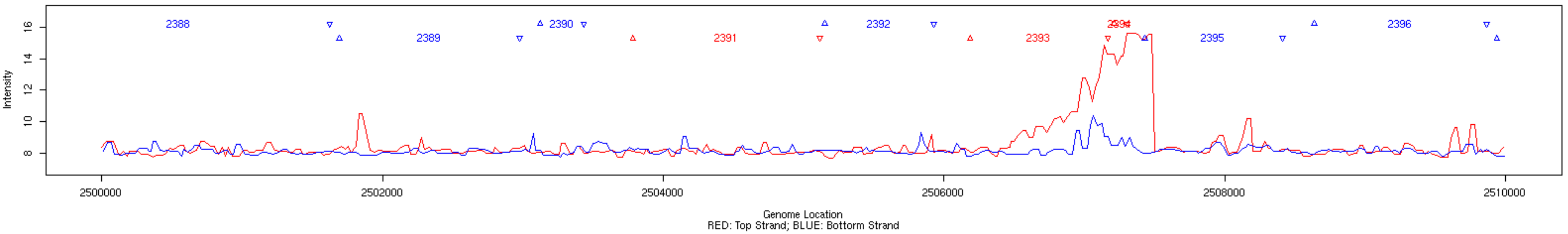
Intensity
Genome Location
RED: Top Strand; BLUE: Bottorm Strand

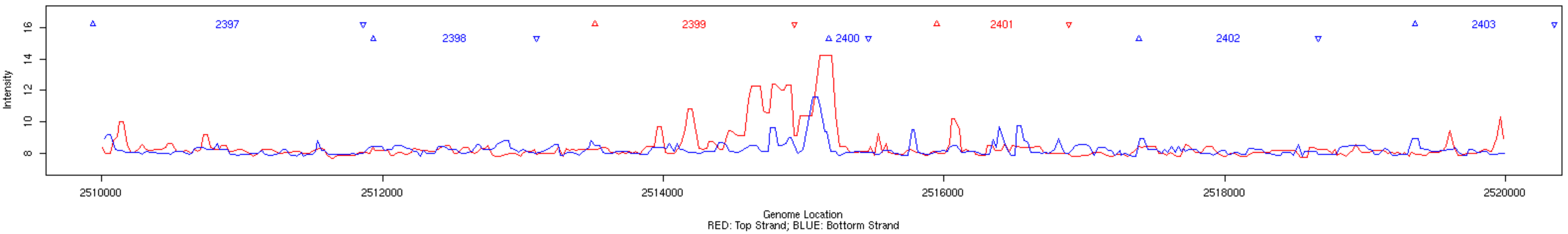
Intensity
Genome Location
RED: Top Strand; BLUE: Bottorm Strand

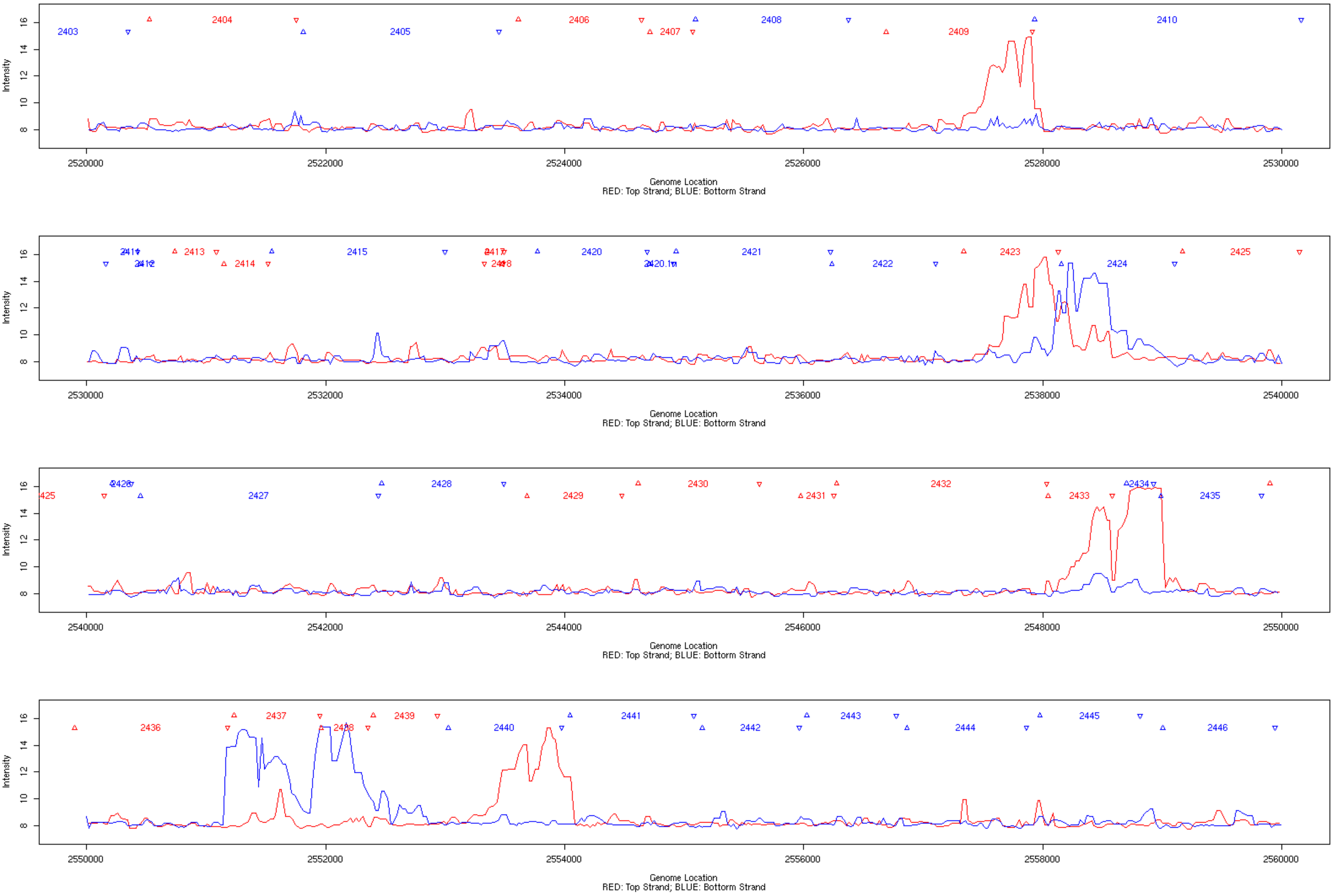
Intensity
Genome Location
RED: Top Strand; BLUE: Bottorm Strand
2403
2404
2405
2406
2407
2408
2409
2410
2413
2414
2415
2420
2421
2422
2423
2424
2425
2427
2428
2429
2430
2431
2432
2433
2435
2436
2437
2439
2440
2441
2442
2443
2444
2445
2446
2520000
2522000
2524000
2526000
2528000
2530000
2532000
2534000
2536000
2538000
2540000
2542000
2544000
2546000
2548000
2550000
2552000
2554000
2556000
2558000
2560000

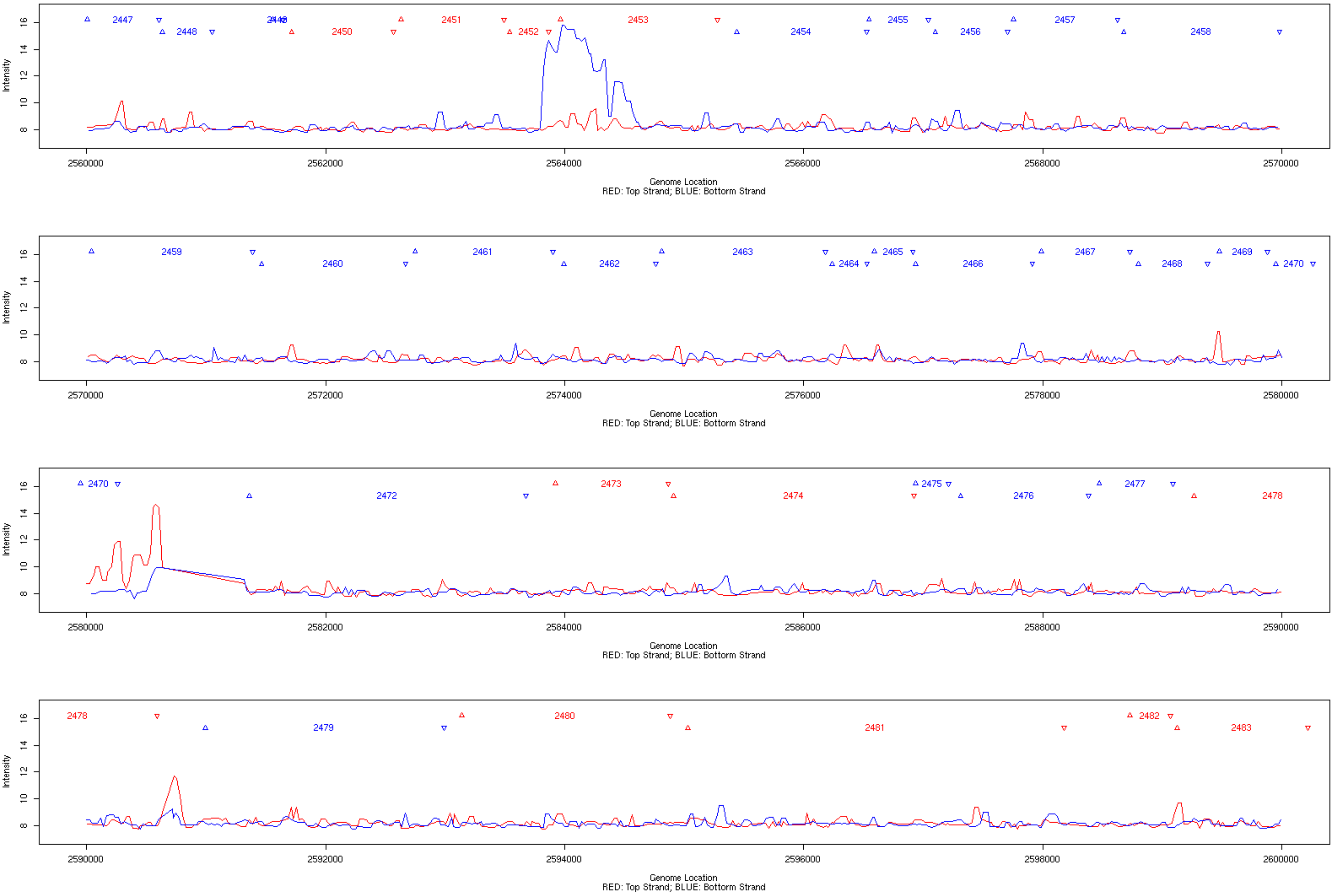
2447
2448
2450
2451
2452
2453
2454
2455
2456
2457
2458
Intensity
Genome Location
RED: Top Strand; BLUE: Bottorm Strand
2459
2460
2461
2462
2463
2464
2465
2466
2467
2468
2469
2470
Intensity
Genome Location
RED: Top Strand; BLUE: Bottorm Strand
2470
2472
2473
2474
2475
2476
2477
2478
Intensity
Genome Location
RED: Top Strand; BLUE: Bottorm Strand
2478
2479
2480
2481
2482
2483
Intensity
Genome Location
RED: Top Strand; BLUE: Bottorm Strand

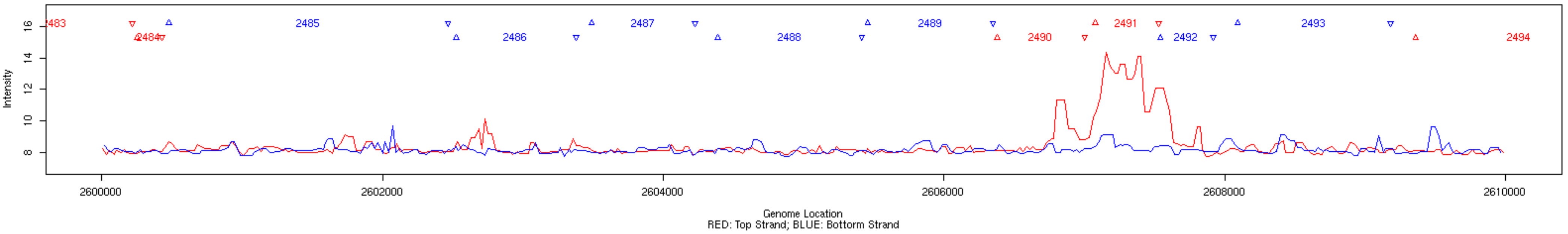
Intensity
2483
2484
2485
2486
2487
2488
2489
2490
2491
2492
2493
2494
2600000
2602000
2604000
2606000
2608000
2610000
Genome Location
RED: Top Strand; BLUE: Bottorm Strand
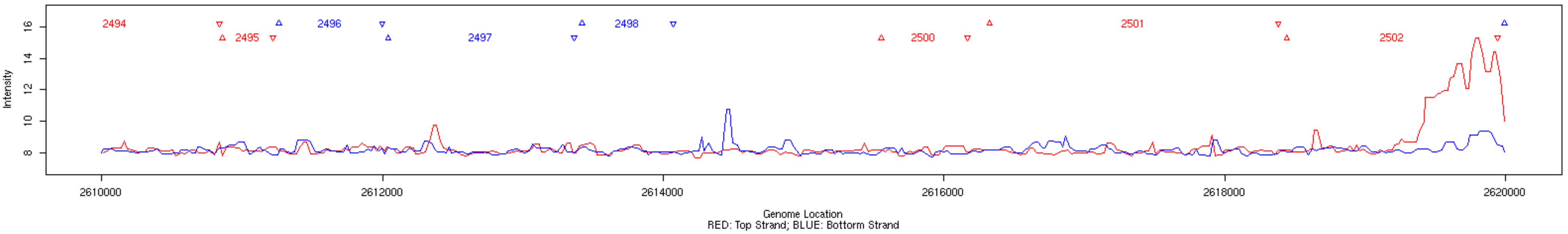
Intensity
2494
2495
2496
2497
2498
2500
2501
2502
2610000
2612000
2614000
2616000
2618000
2620000
Genome Location
RED: Top Strand; BLUE: Bottorm Strand
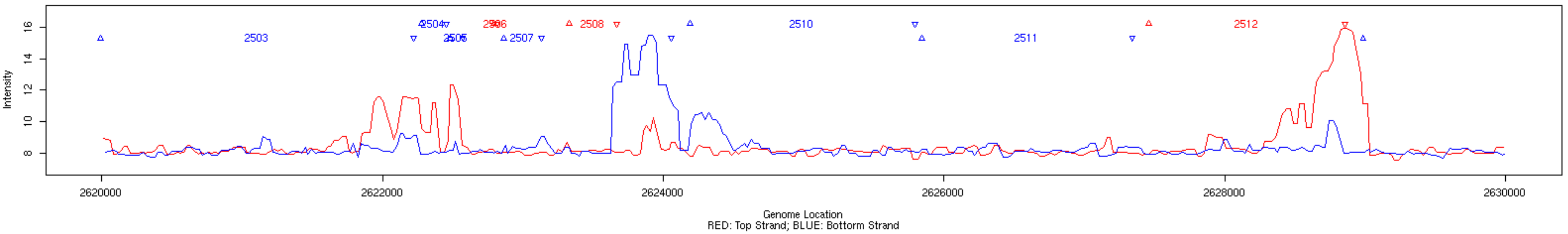
Intensity
2503
2504
2507
2508
2510
2511
2512
2620000
2622000
2624000
2626000
2628000
2630000
Genome Location
RED: Top Strand; BLUE: Bottorm Strand
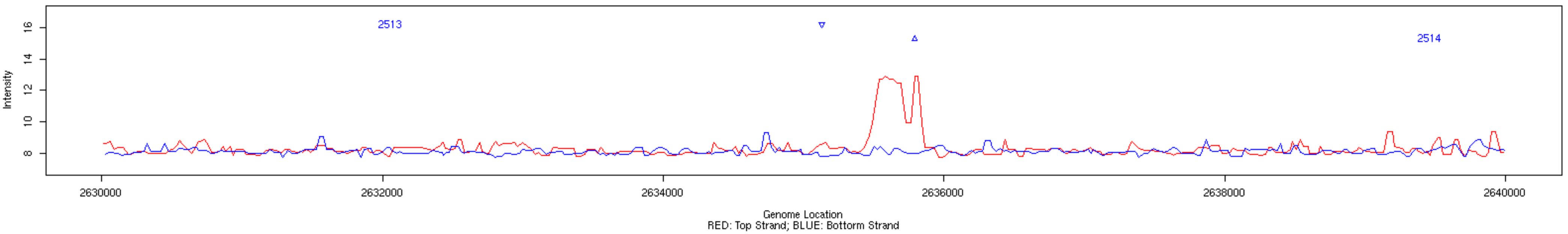
Intensity
2513
2514
2630000
2632000
2634000
2636000
2638000
2640000
Genome Location
RED: Top Strand; BLUE: Bottorm Strand

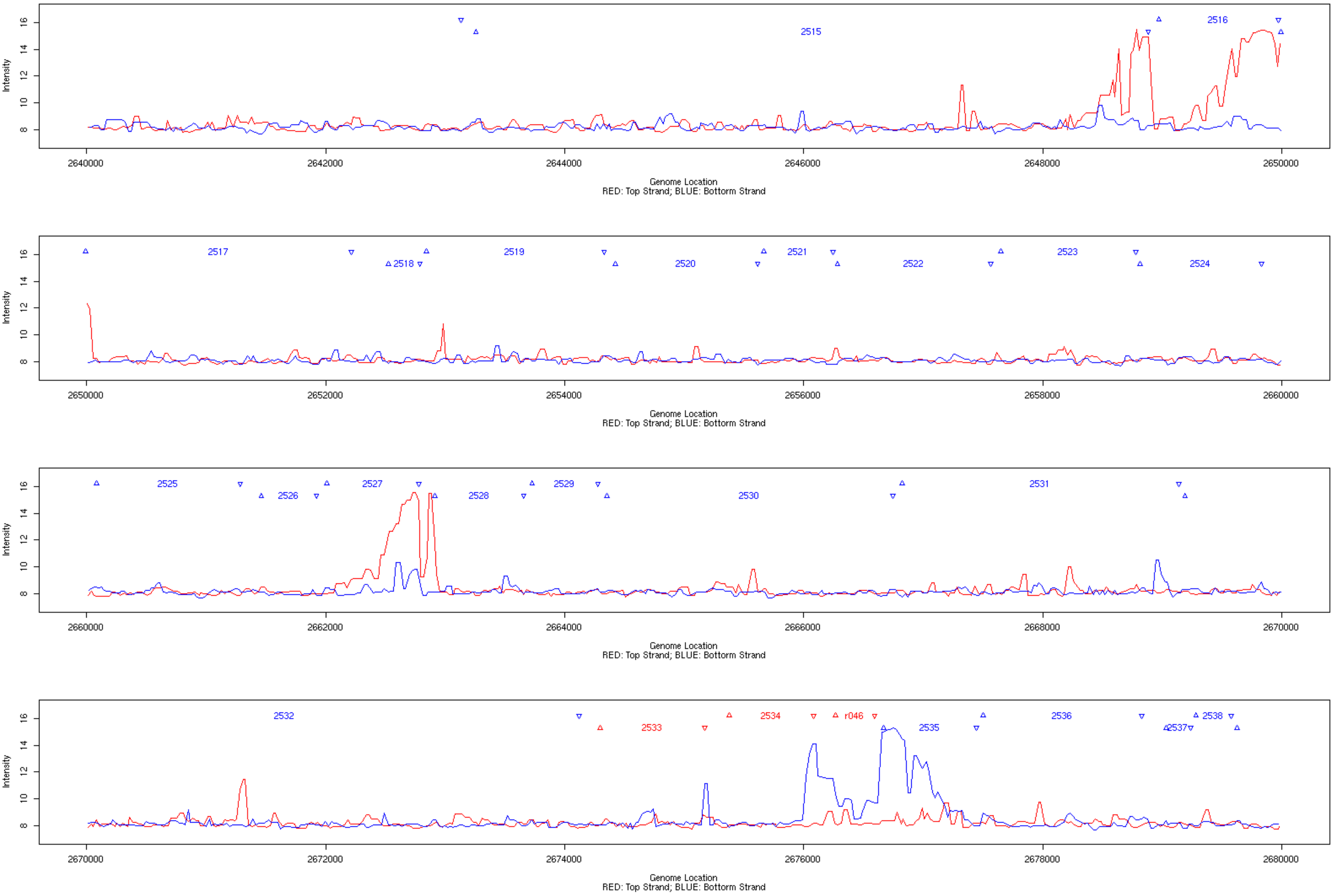

Intensity
2515
2516
2640000
2642000
2644000
2646000
2648000
2650000
Genome Location
RED: Top Strand; BLUE: Bottorm Strand
2517
2518
2519
2520
2521
2522
2523
2524
2652000
2654000
2656000
2658000
2660000
2525
2526
2527
2528
2529
2530
2531
2662000
2664000
2666000
2668000
2670000
2532
2533
2534
r046
2535
2536
2537
2538
2672000
2674000
2676000
2678000
2680000

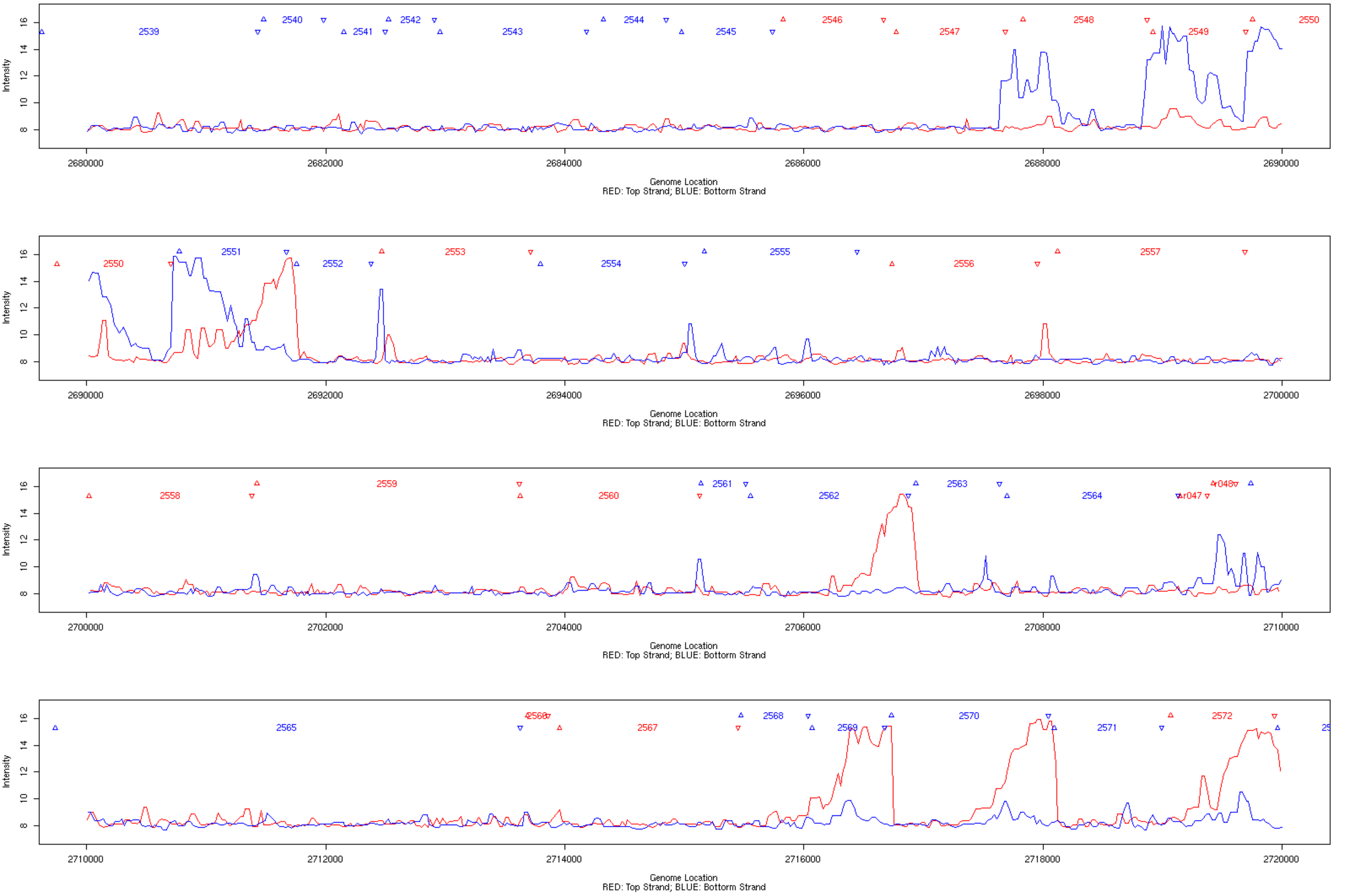
2539
2540
2541
2542
2543
2544
2545
2546
2547
2548
2549
2550
Intensity
2680000
2682000
2684000
2686000
2688000
2690000
Genome Location
RED: Top Strand; BLUE: Bottorm Strand
2550
2551
2552
2553
2554
2555
2556
2557
Intensity
2690000
2692000
2694000
2696000
2698000
2700000
Genome Location
RED: Top Strand; BLUE: Bottorm Strand
2558
2559
2560
2561
2562
2563
2564
r047
r048
Intensity
2700000
2702000
2704000
2706000
2708000
2710000
Genome Location
RED: Top Strand; BLUE: Bottorm Strand
2565
2566
2567
2568
2569
2570
2571
2572
Intensity
2710000
2712000
2714000
2716000
2718000
2720000
Genome Location
RED: Top Strand; BLUE: Bottorm Strand

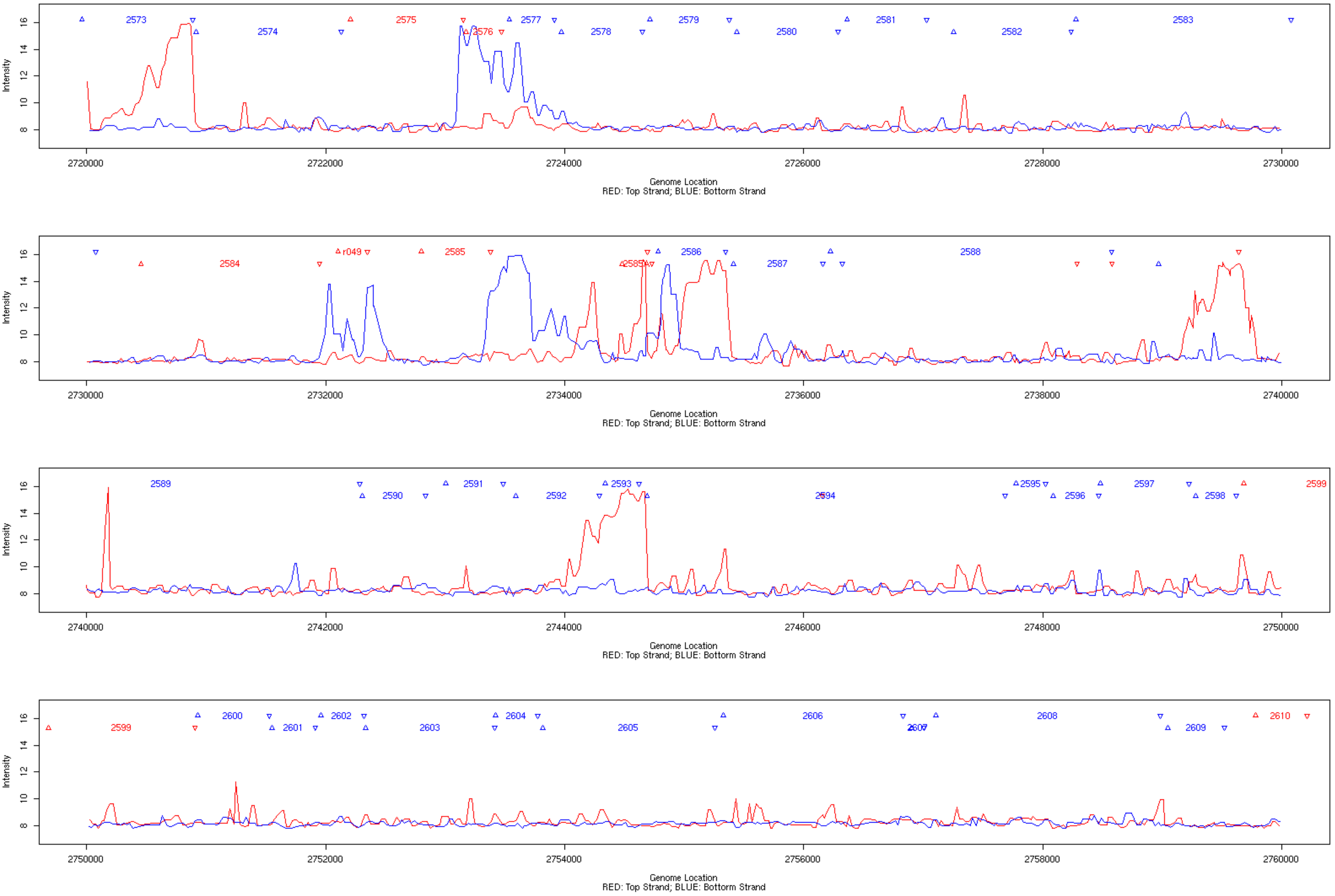
Intensity
Genome Location
RED: Top Strand; BLUE: Bottorm Strand
2573
2574
2575
2576
2577
2578
2579
2580
2581
2582
2583
2584
r049
2585
2586
2587
2588
2589
2590
2591
2592
2593
2595
2596
2597
2598
2599
2600
2601
2602
2603
2604
2605
2606
2608
2609
2610

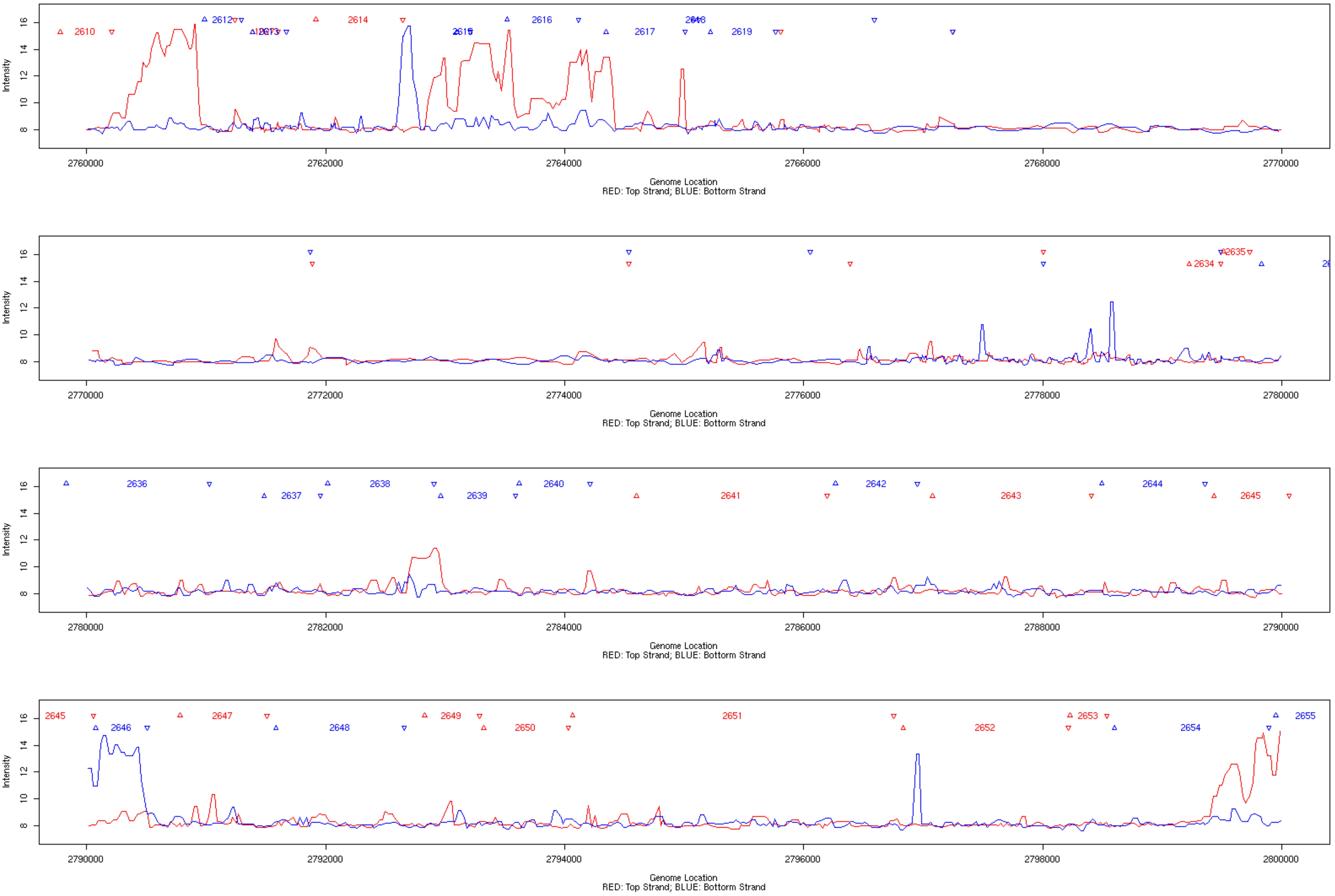
Intensity
Genome Location
RED: Top Strand; BLUE: Bottom Strand
2610
2612
2614
2616
2617
2619
2636
2637
2638
2639
2640
2641
2642
2643
2644
2645
2646
2647
2648
2649
2650
2651
2652
2653
2654
2655
2634
2635
2760000
2762000
2764000
2766000
2768000
2770000
2772000
2774000
2776000
2778000
2780000
2782000
2784000
2786000
2788000
2790000
2792000
2794000
2796000
2798000
2800000

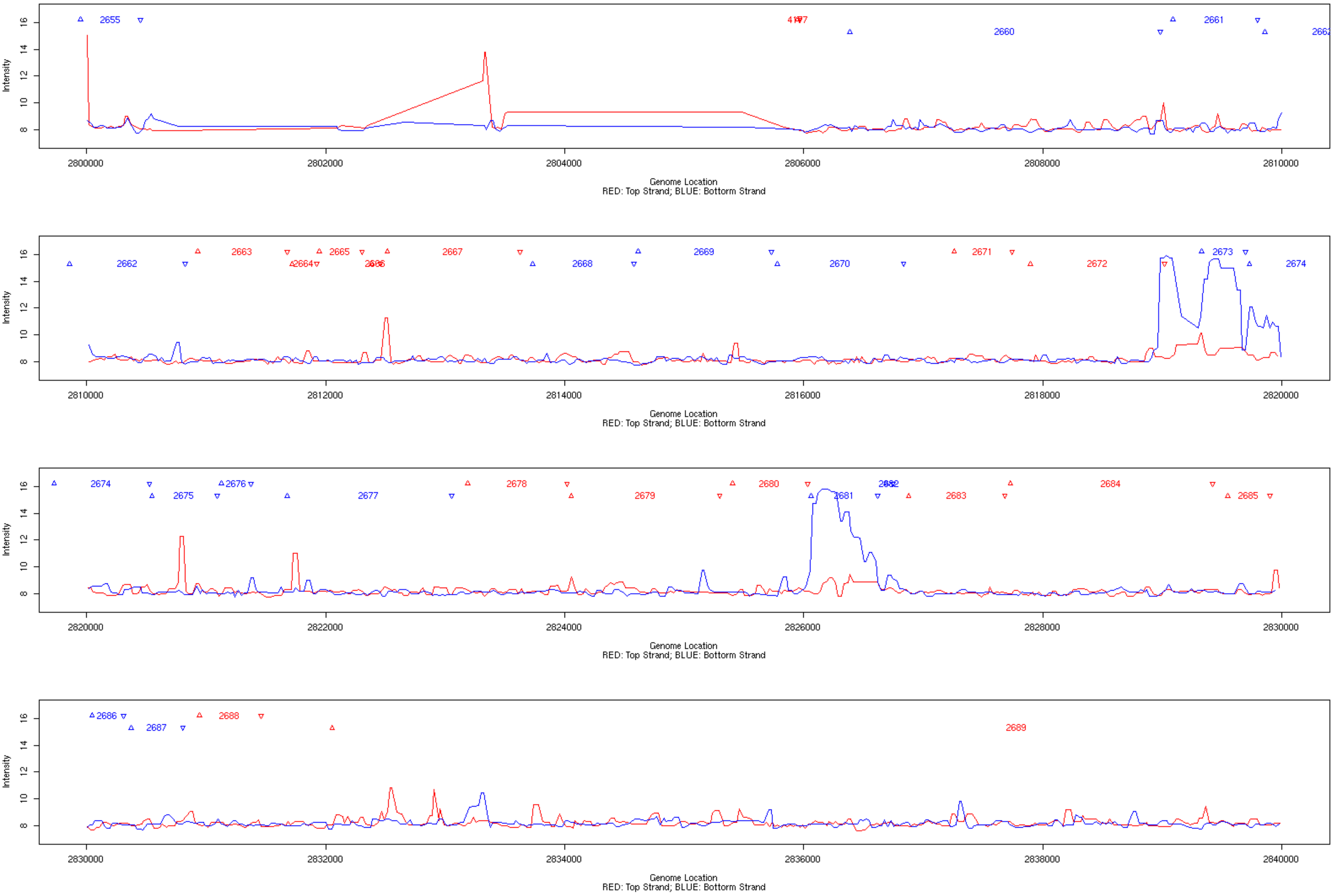

Intensity
Genome Location
RED: Top Strand; BLUE: Bottom Strand
2655
2660
2661
2662
2663
2664
2665
2667
2668
2669
2670
2671
2672
2673
2674
2675
2676
2677
2678
2679
2680
2681
2683
2684
2685
2686
2687
2688
2689
2800000
2802000
2804000
2806000
2808000
2810000
2812000
2814000
2816000
2818000
2820000
2822000
2824000
2826000
2828000
2830000
2832000
2834000
2836000
2838000
2840000

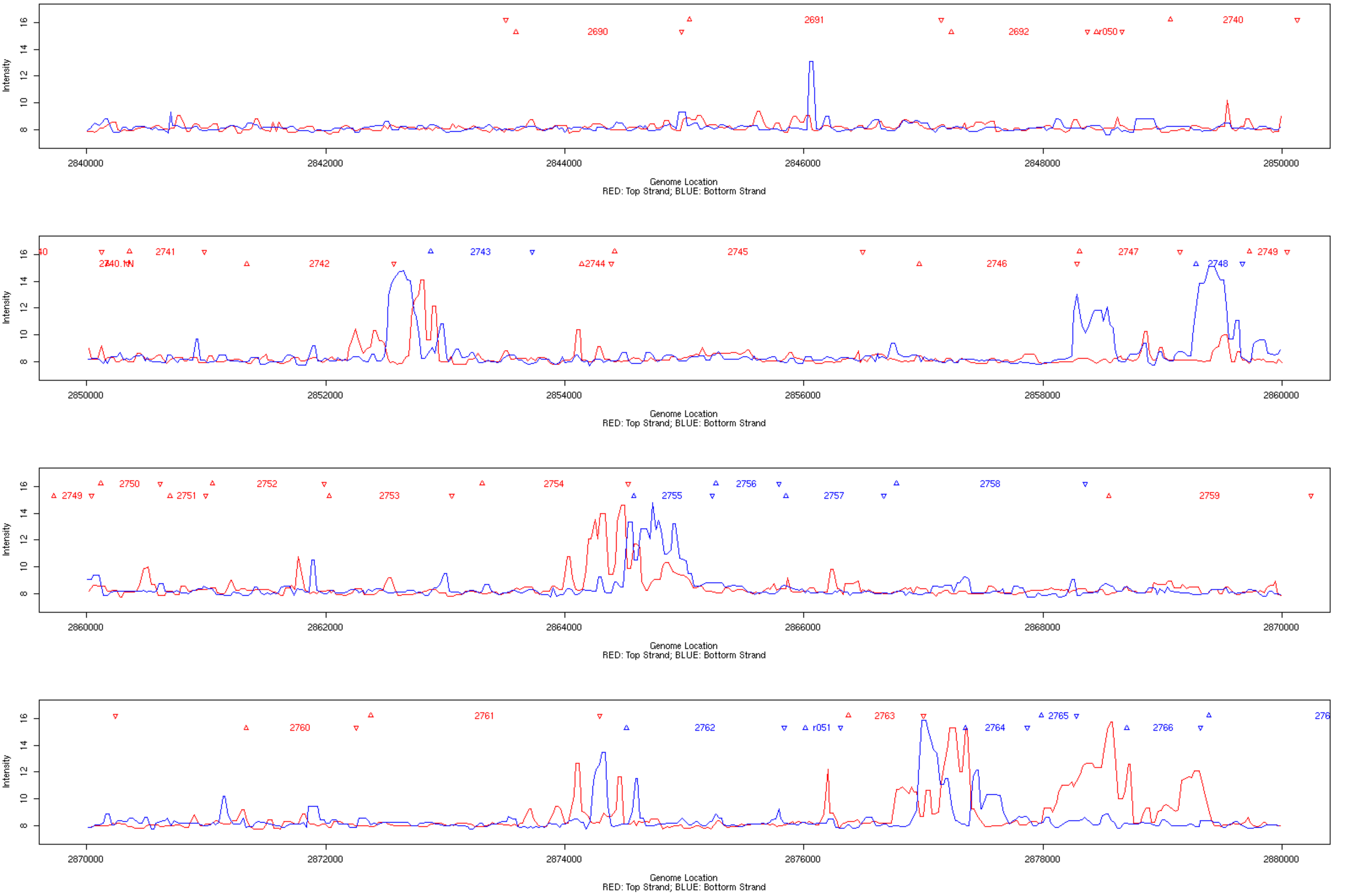

Intensity
2690
2691
2692
r050
2740
2840000
2842000
2844000
2846000
2848000
2850000
Genome Location
RED: Top Strand; BLUE: Bottom Strand
2741
2742
2743
2744
2745
2746
2747
2748
2749
2850000
2852000
2854000
2856000
2858000
2860000
Genome Location
RED: Top Strand; BLUE: Bottom Strand
2749
2750
2751
2752
2753
2754
2755
2756
2757
2758
2759
2860000
2862000
2864000
2866000
2868000
2870000
Genome Location
RED: Top Strand; BLUE: Bottom Strand
2760
2761
2762
r051
2763
2764
2765
2766
2870000
2872000
2874000
2876000
2878000
2880000
Genome Location
RED: Top Strand; BLUE: Bottom Strand

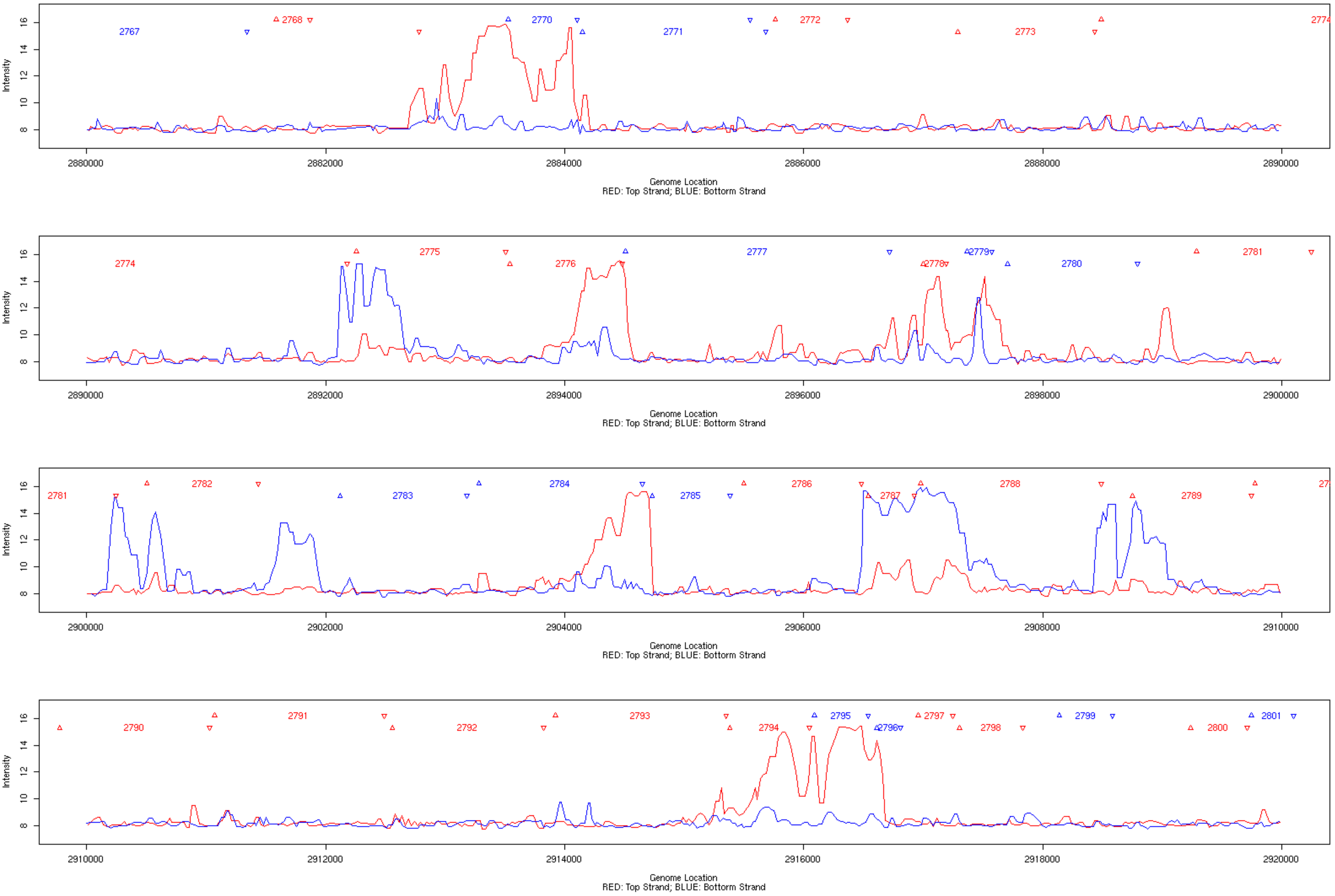
Intensity
Genome Location
RED: Top Strand; BLUE: Bottorm Strand
2767
2768
2770
2771
2772
2773
2774
2775
2776
2777
2778
2779
2780
2781
2782
2783
2784
2785
2786
2787
2788
2789
2790
2791
2792
2793
2794
2795
2796
2797
2798
2799
2800
2801

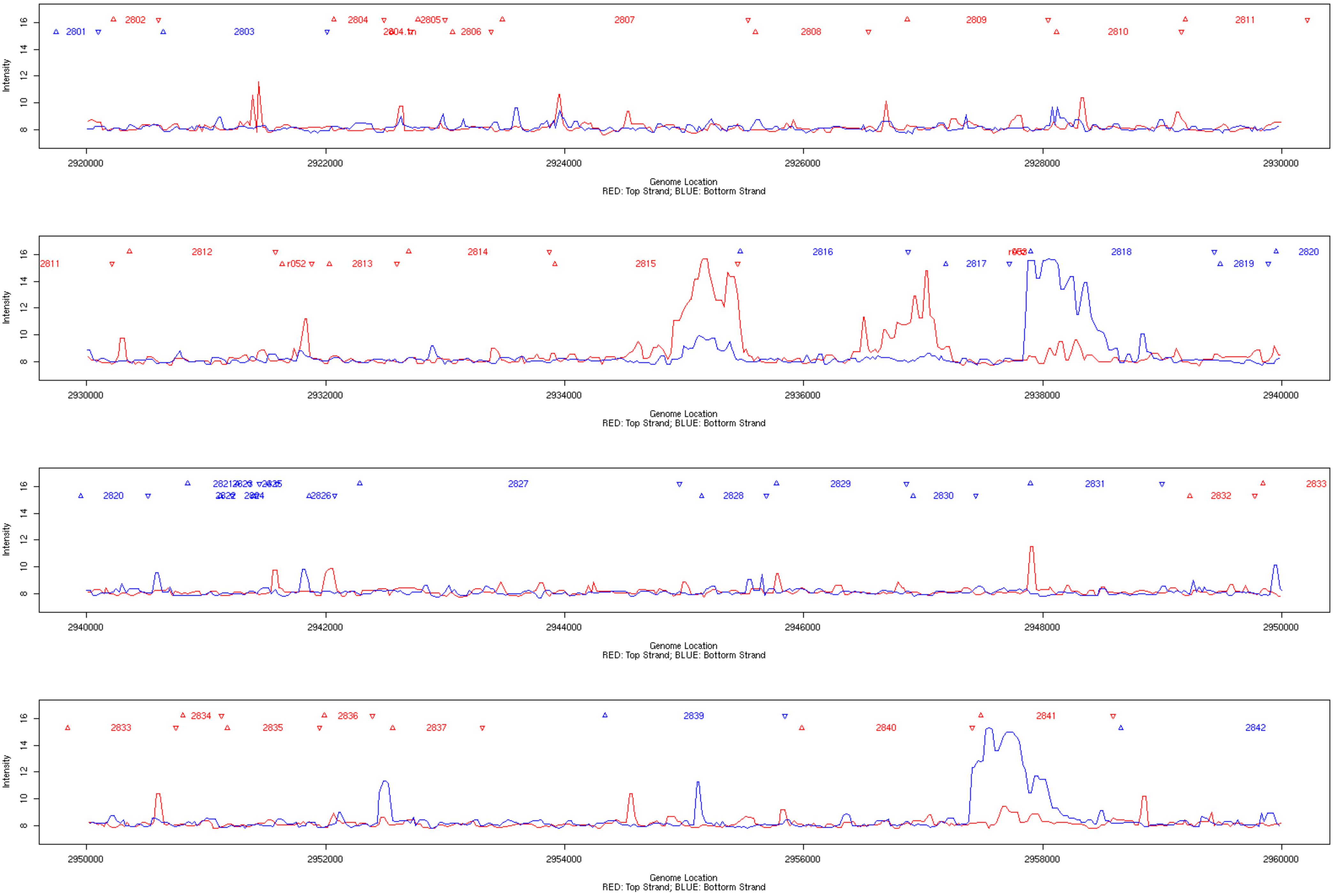
Intensity
Genome Location
RED: Top Strand; BLUE: Bottorm Strand
2801
2802
2803
2804
2805
2806
2807
2808
2809
2810
2811
2812
r052
2813
2814
2815
2816
2817
2818
2819
2820
2826
2827
2828
2829
2830
2831
2832
2833
2834
2835
2836
2837
2839
2840
2841
2842

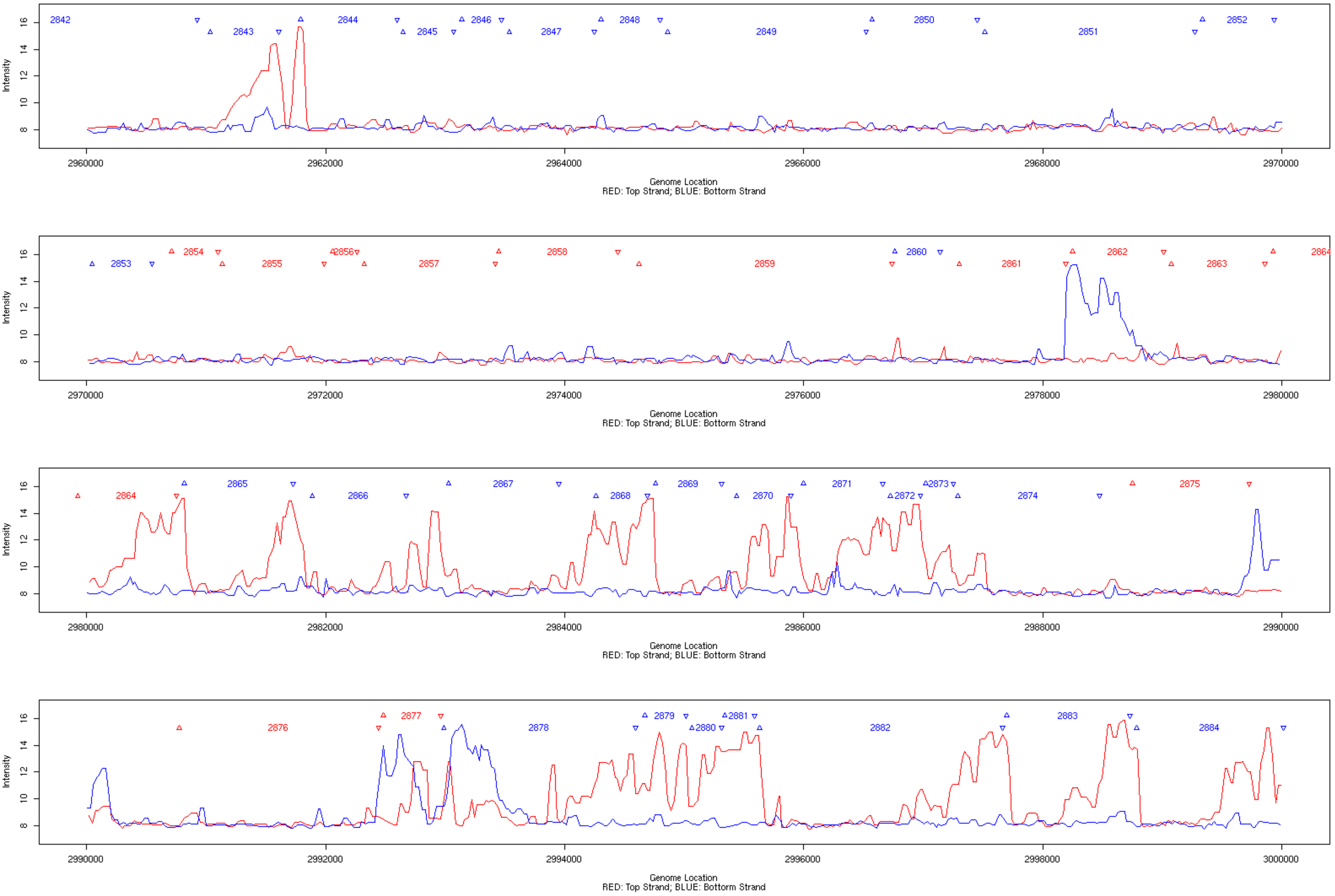

Intensity
16
14
12
10
8
2842
2843
2844
2845
2846
2847
2848
2849
2850
2851
2852
2960000
2962000
2964000
2966000
2968000
2970000
Genome Location
RED: Top Strand; BLUE: Bottorm Strand
2853
2854
2855
2856
2857
2858
2859
2860
2861
2862
2863
2864
2972000
2974000
2976000
2978000
2980000
2865
2866
2867
2868
2869
2870
2871
2872
2873
2874
2875
2982000
2984000
2986000
2988000
2990000
2876
2877
2878
2879
2880
2881
2882
2883
2884
2992000
2994000
2996000
2998000
3000000

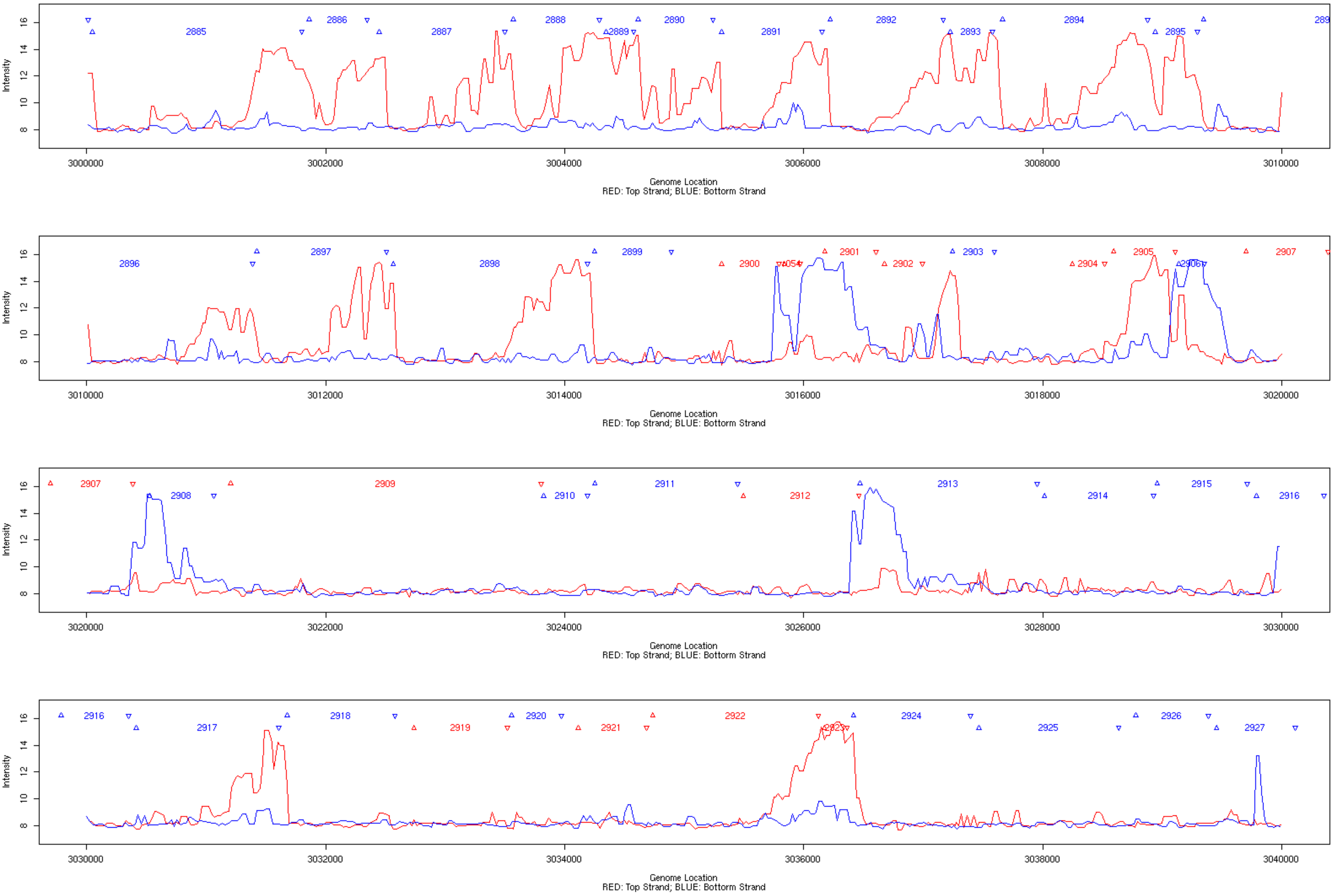
Intensity
Genome Location
RED: Top Strand; BLUE: Bottorm Strand
3000000 3002000 3004000 3006000 3008000 3010000
2885 2886 2887 2888 2889 2890 2891 2892 2893 2894 2895
3010000 3012000 3014000 3016000 3018000 3020000
2896 2897 2898 2899 2900 2901 2902 2903 2904 2905 2907
3020000 3022000 3024000 3026000 3028000 3030000
2907 2908 2909 2910 2911 2912 2913 2914 2915 2916
3030000 3032000 3034000 3036000 3038000 3040000
2916 2917 2918 2919 2920 2921 2922 2924 2925 2926 2927

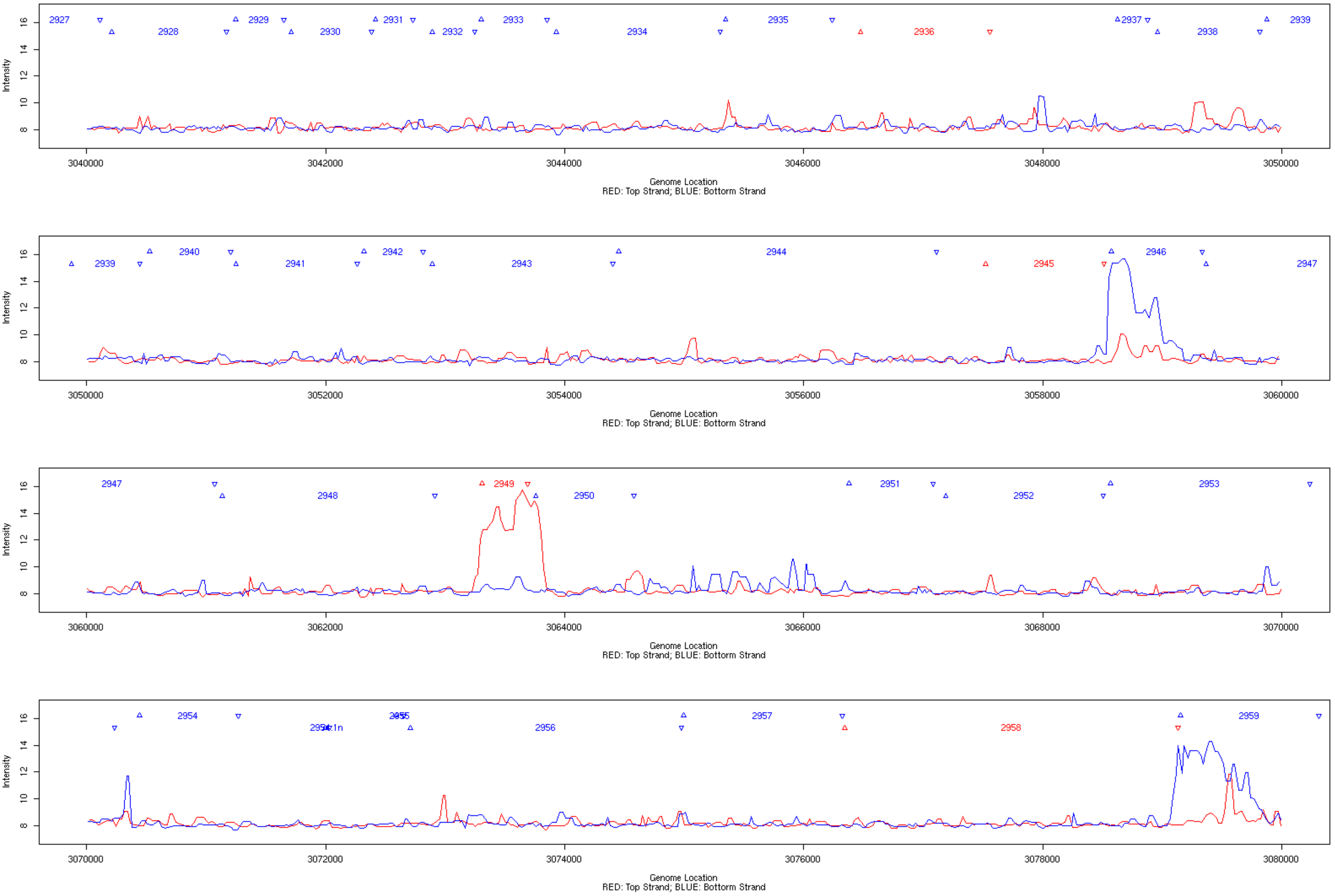
Intensity
2927
2928
2929
2930
2931
2932
2933
2934
2935
2936
2937
2938
2939
3040000
3042000
3044000
3046000
3048000
3050000
Genome Location
RED: Top Strand; BLUE: Bottorm Strand
2940
2941
2942
2943
2944
2945
2946
2947
3052000
3054000
3056000
3058000
3060000
2948
2949
2950
2951
2952
2953
3062000
3064000
3066000
3068000
3070000
2954
2956
2957
2958
2959
3072000
3074000
3076000
3078000
3080000

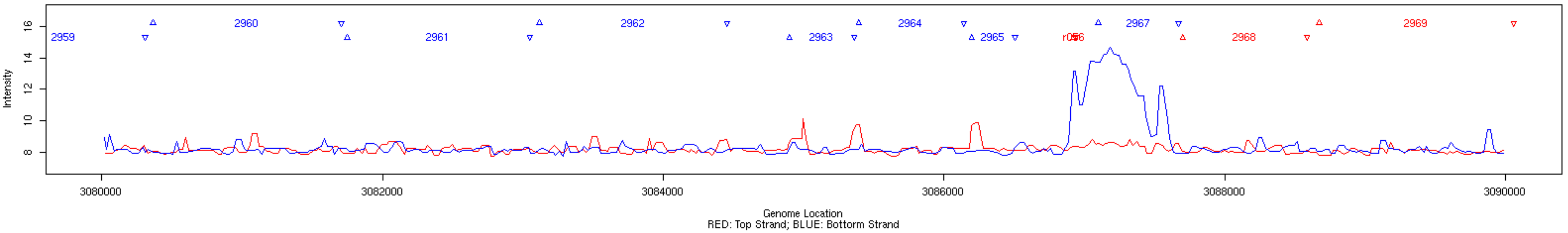
Intensity
2959
2960
2961
2962
2963
2964
2965
2967
r056
2968
2969
3080000
3082000
3084000
3086000
3088000
3090000
Genome Location
RED: Top Strand; BLUE: Bottom Strand

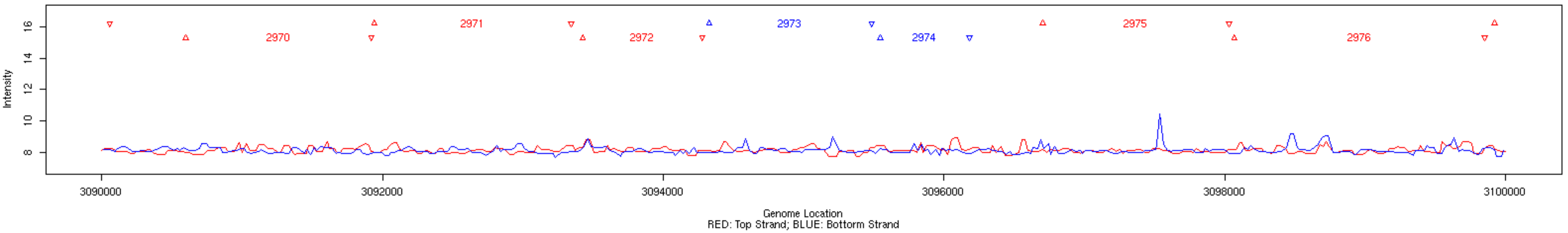
Intensity
2970
2971
2972
2973
2974
2975
2976
3090000
3092000
3094000
3096000
3098000
3100000
Genome Location
RED: Top Strand; BLUE: Bottom Strand

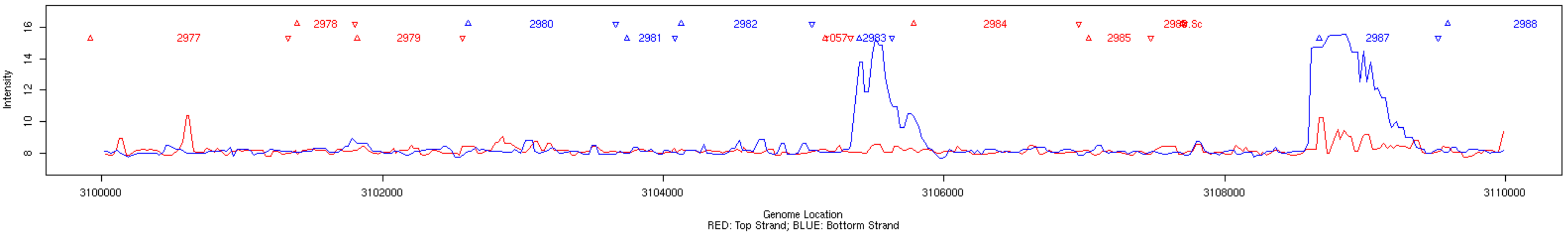
Intensity
2977
2978
2979
2980
2981
2982
r057
2983
2984
2985
2986.Sc
2987
2988
3100000
3102000
3104000
3106000
3108000
3110000
Genome Location
RED: Top Strand; BLUE: Bottom Strand

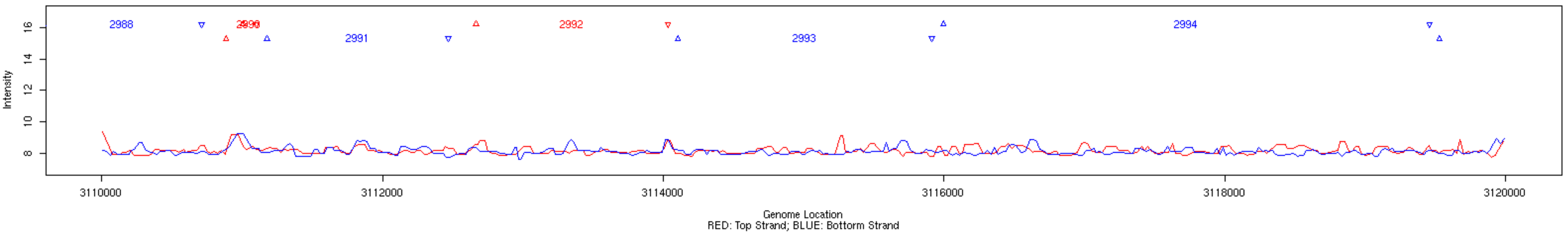
Intensity
2988
2990
2991
2992
2993
2994
3110000
3112000
3114000
3116000
3118000
3120000
Genome Location
RED: Top Strand; BLUE: Bottom Strand

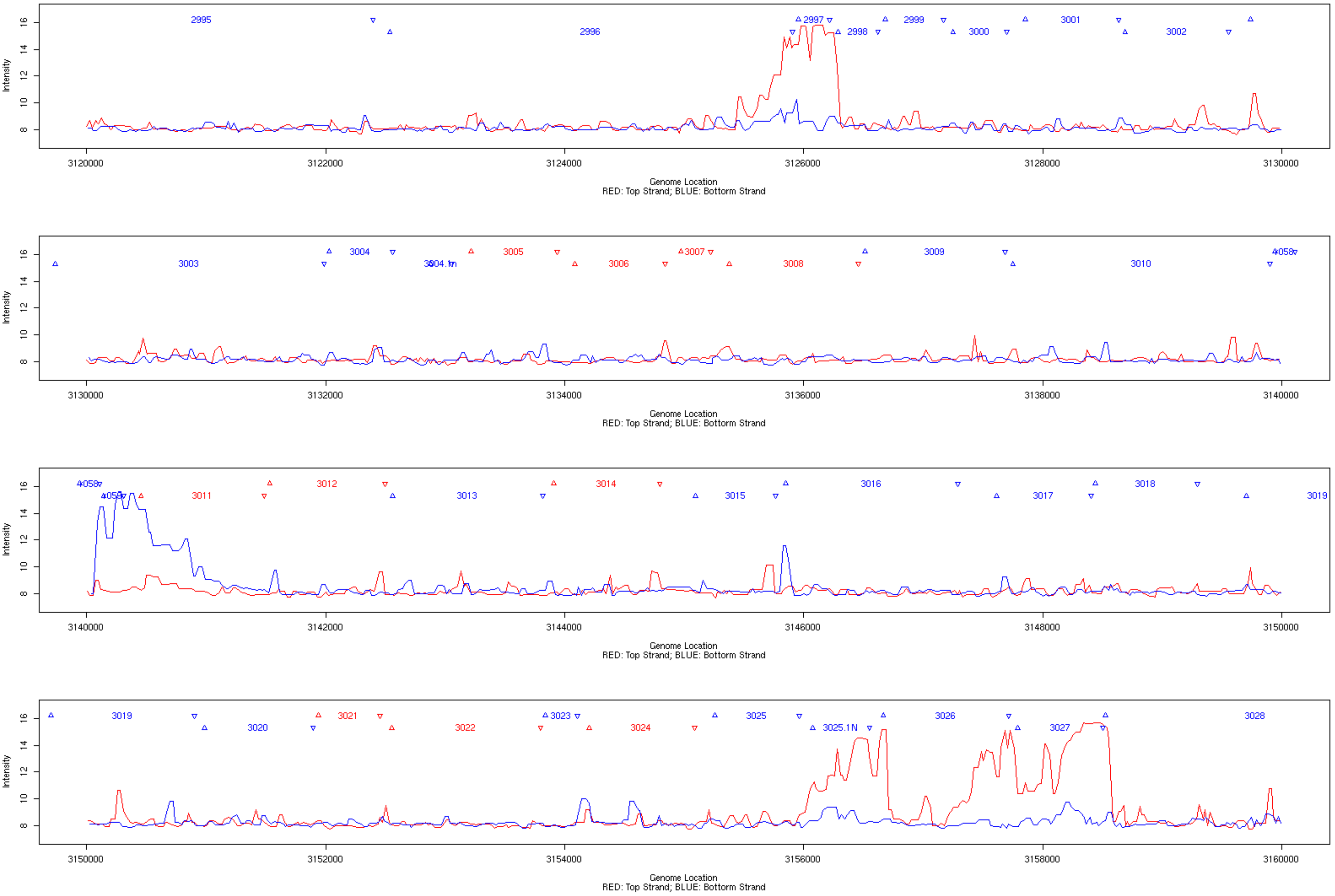
Intensity
2995
2996
2997
2998
2999
3000
3001
3002
Genome Location
RED: Top Strand; BLUE: Bottorm Strand
3003
3004
3005
3006
3007
3008
3009
3010
Genome Location
RED: Top Strand; BLUE: Bottorm Strand
3011
3012
3013
3014
3015
3016
3017
3018
3019
Genome Location
RED: Top Strand; BLUE: Bottorm Strand
3019
3020
3021
3022
3023
3024
3025
3025.1N
3026
3027
3028
Genome Location
RED: Top Strand; BLUE: Bottorm Strand

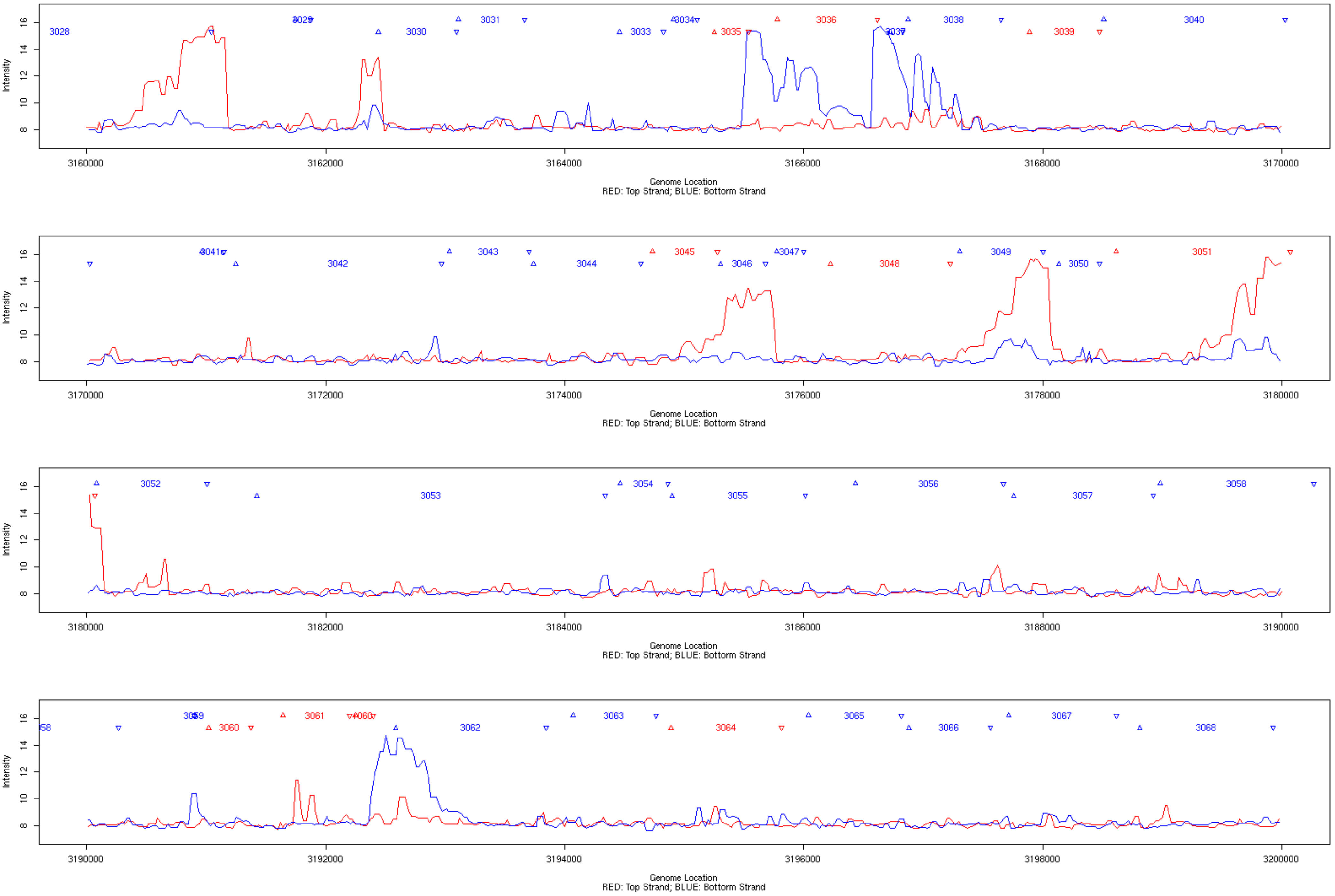
3028
3029
3030
3031
3033
3034
3035
3036
3037
3038
3039
3040
Intensity
Genome Location
RED: Top Strand; BLUE: Bottorm Strand
3041
3042
3043
3044
3045
3046
3047
3048
3049
3050
3051
Intensity
Genome Location
RED: Top Strand; BLUE: Bottorm Strand
3052
3053
3054
3055
3056
3057
3058
Intensity
Genome Location
RED: Top Strand; BLUE: Bottorm Strand
3059
3060
3061
3062
3063
3064
3065
3066
3067
3068
Intensity
Genome Location
RED: Top Strand; BLUE: Bottorm Strand

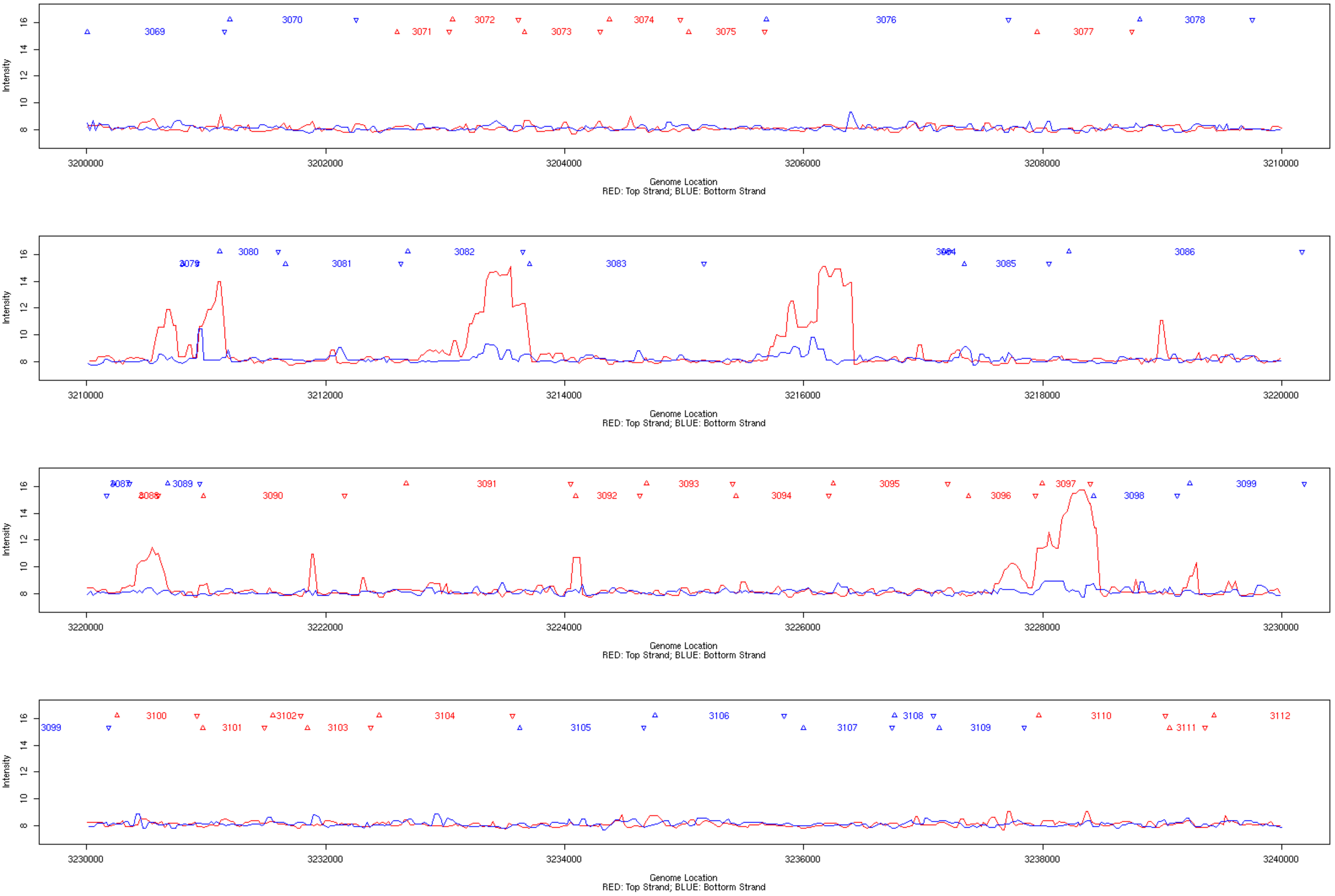
Intensity
Genome Location
RED: Top Strand; BLUE: Bottorm Strand
3069 3070 3071 3072 3073 3074 3075 3076 3077 3078
3080 3081 3082 3083 3085 3086
3090 3091 3092 3093 3094 3095 3096 3097 3098 3099
3099 3100 3101 3102 3103 3104 3105 3106 3107 3108 3109 3110 3111 3112

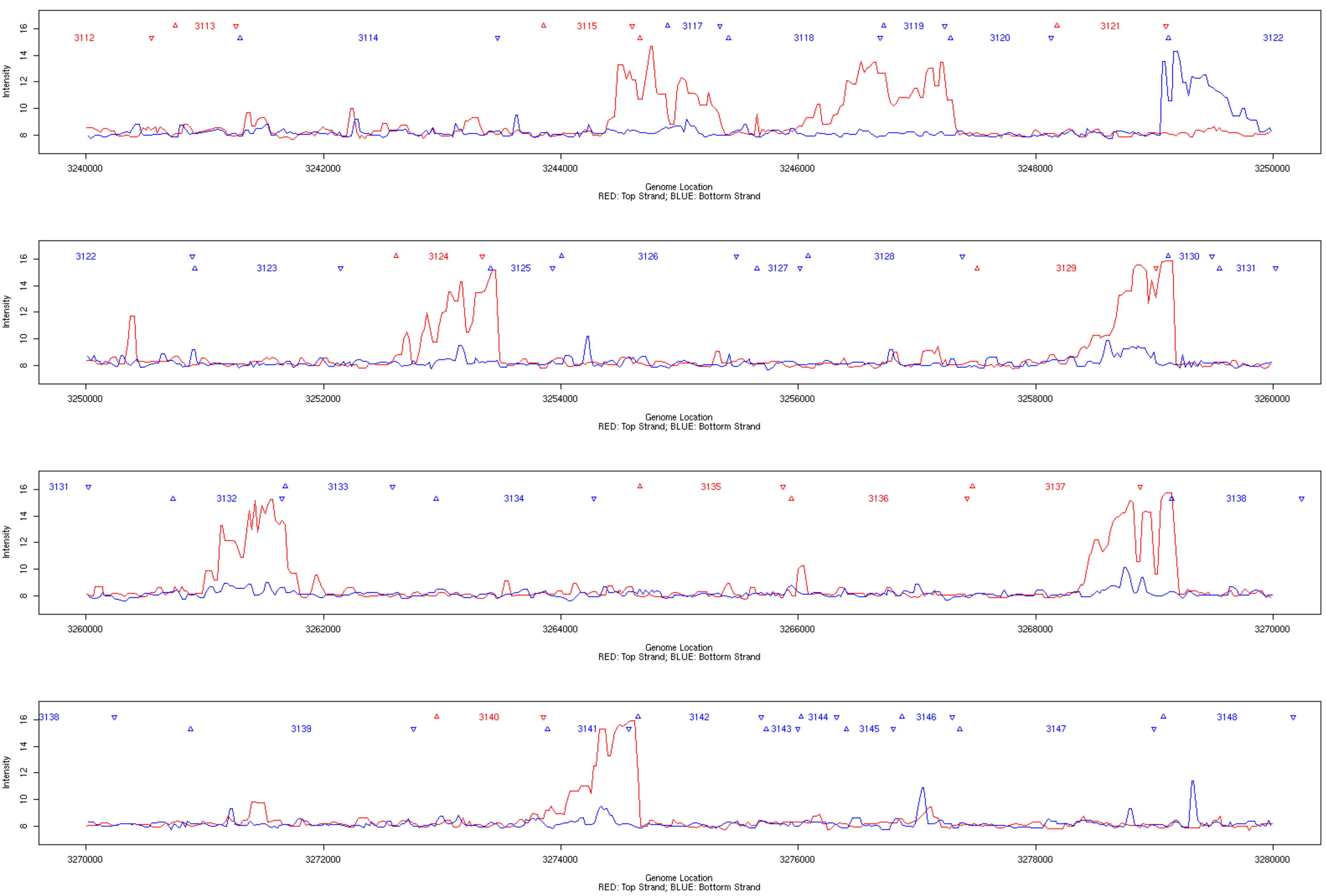
Intensity
3112
3113
3114
3115
3117
3118
3119
3120
3121
3122
Genome Location
RED: Top Strand; BLUE: Bottorm Strand
3123
3124
3125
3126
3127
3128
3129
3130
3131
3132
3133
3134
3135
3136
3137
3138
3139
3140
3141
3142
3143
3144
3145
3146
3147
3148

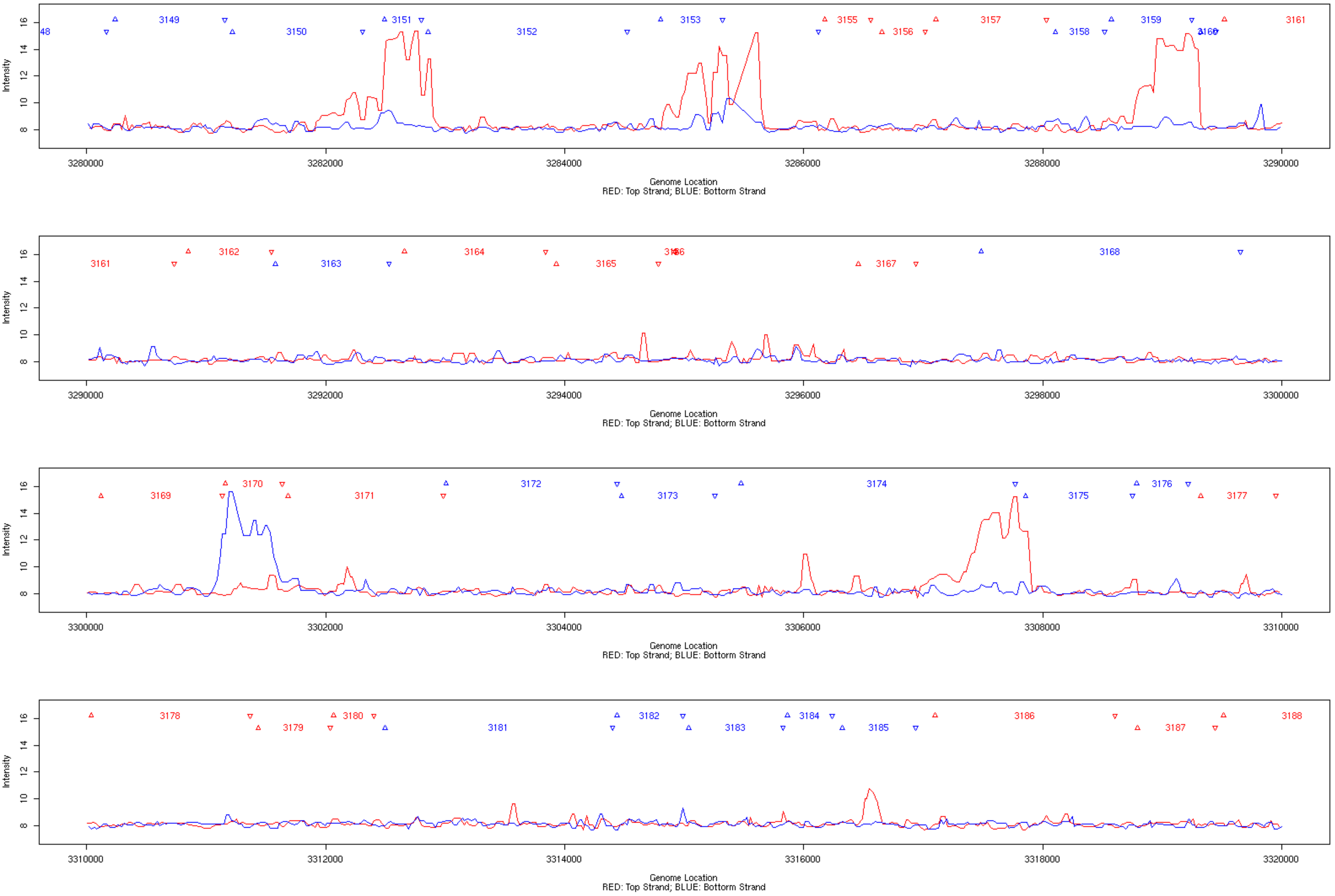
Intensity
Genome Location
RED: Top Strand; BLUE: Bottorm Strand
3280000
3282000
3284000
3286000
3288000
3290000
3292000
3294000
3296000
3298000
3300000
3302000
3304000
3306000
3308000
3310000
3312000
3314000
3316000
3318000
3320000

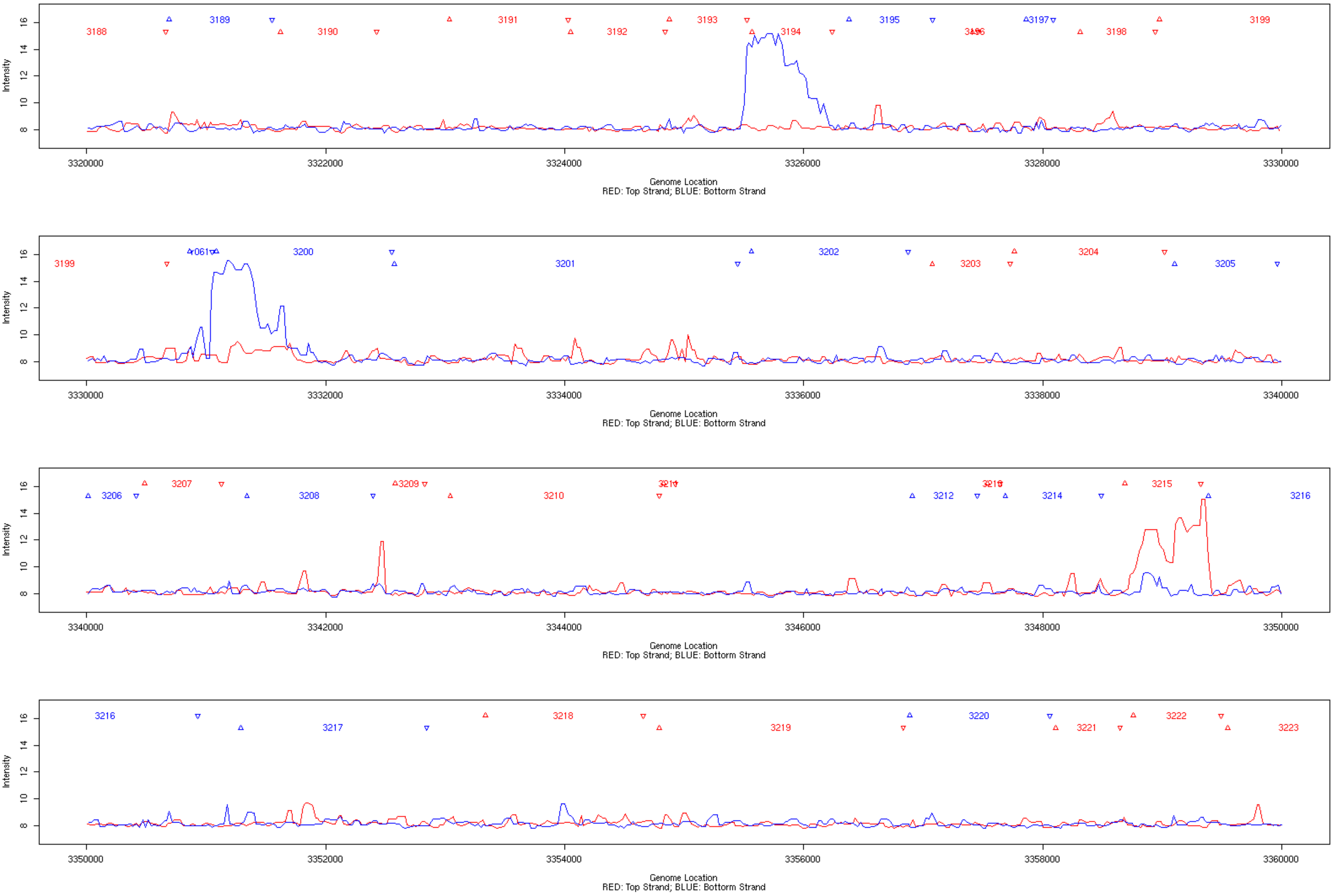
Intensity
Genome Location
RED: Top Strand; BLUE: Bottom Strand
3188
3189
3190
3191
3192
3193
3194
3195
3197
3198
3199
3200
3201
3202
3203
3204
3205
3206
3207
3208
3209
3210
3212
3214
3215
3216
3217
3218
3219
3220
3221
3222
3223
3320000
3322000
3324000
3326000
3328000
3330000
3332000
3334000
3336000
3338000
3340000
3342000
3344000
3346000
3348000
3350000
3352000
3354000
3356000
3358000
3360000

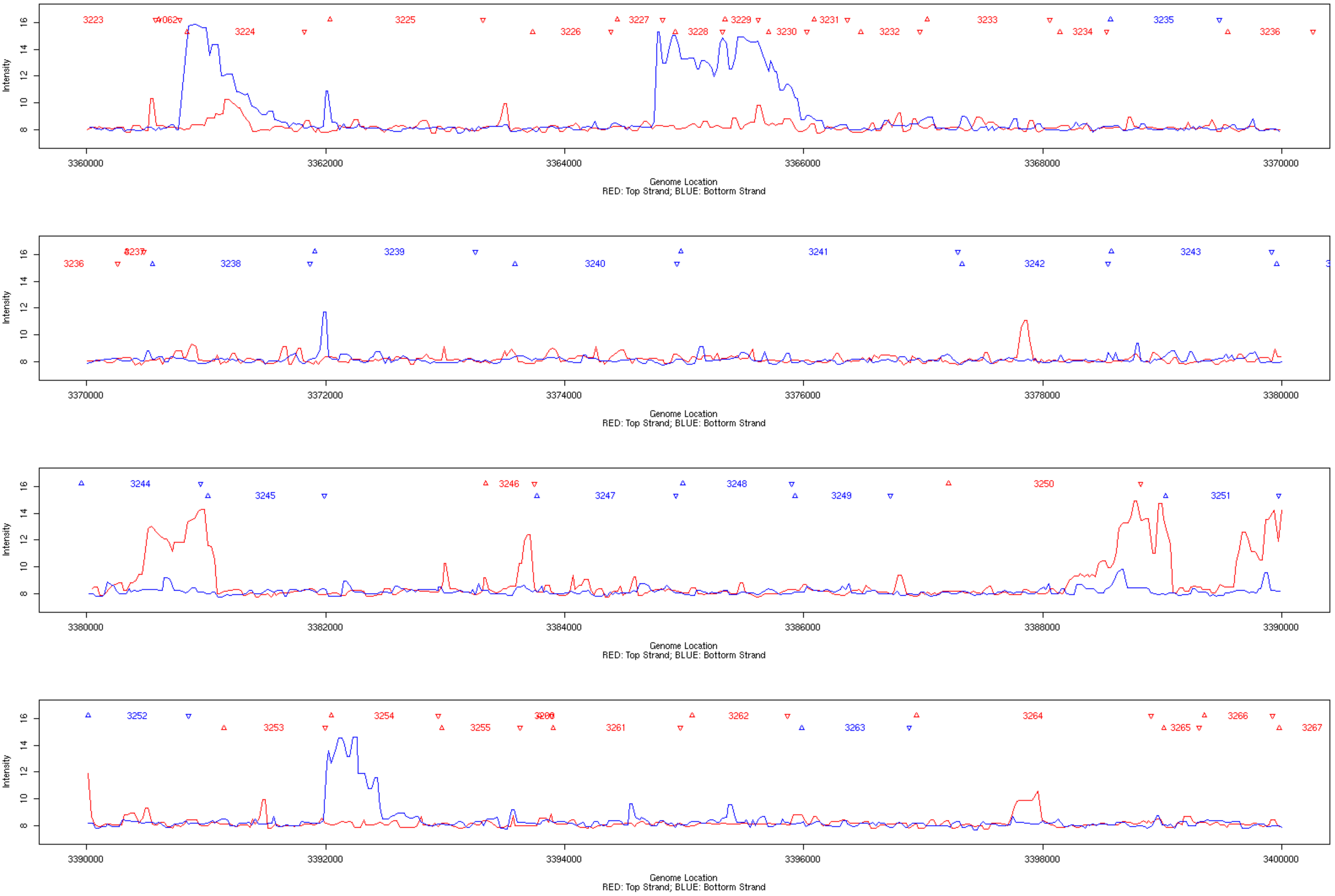

Intensity
Genome Location
RED: Top Strand; BLUE: Bottorm Strand
3223
3224
3225
3226
3227
3228
3229
3230
3231
3232
3233
3234
3235
3236
3238
3239
3240
3241
3242
3243
3244
3245
3246
3247
3248
3249
3250
3251
3252
3253
3254
3255
3261
3262
3263
3264
3265
3266
3267
3360000
3362000
3364000
3366000
3368000
3370000
3372000
3374000
3376000
3378000
3380000
3382000
3384000
3386000
3388000
3390000
3392000
3394000
3396000
3398000
3400000

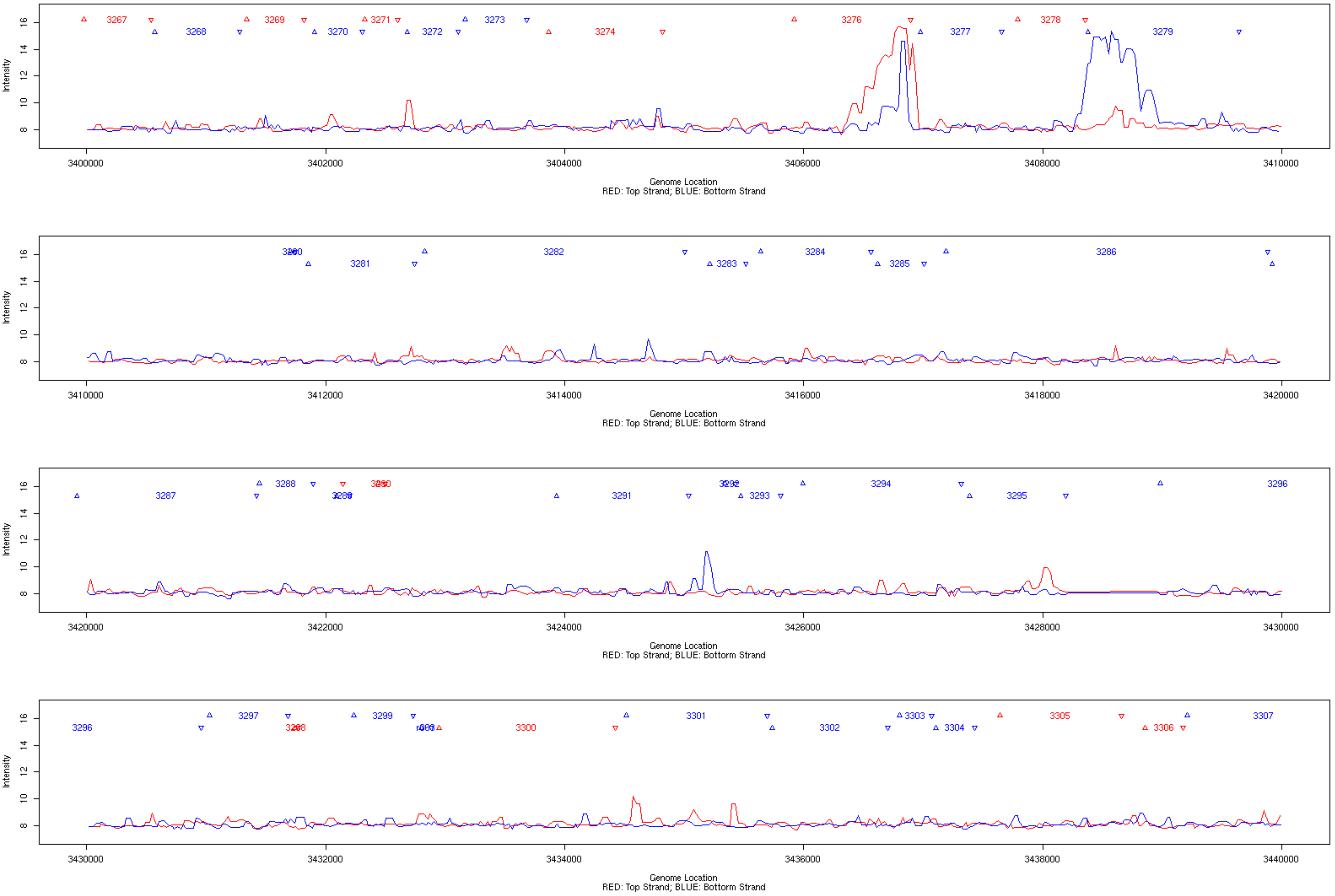

Intensity
Genome Location
RED: Top Strand; BLUE: Bottorm Strand
3267
3268
3269
3270
3271
3272
3273
3274
3276
3277
3278
3279
3281
3282
3283
3284
3285
3286
3287
3288
3291
3293
3294
3295
3296
3297
3299
3300
3301
3302
3303
3304
3305
3306
3307

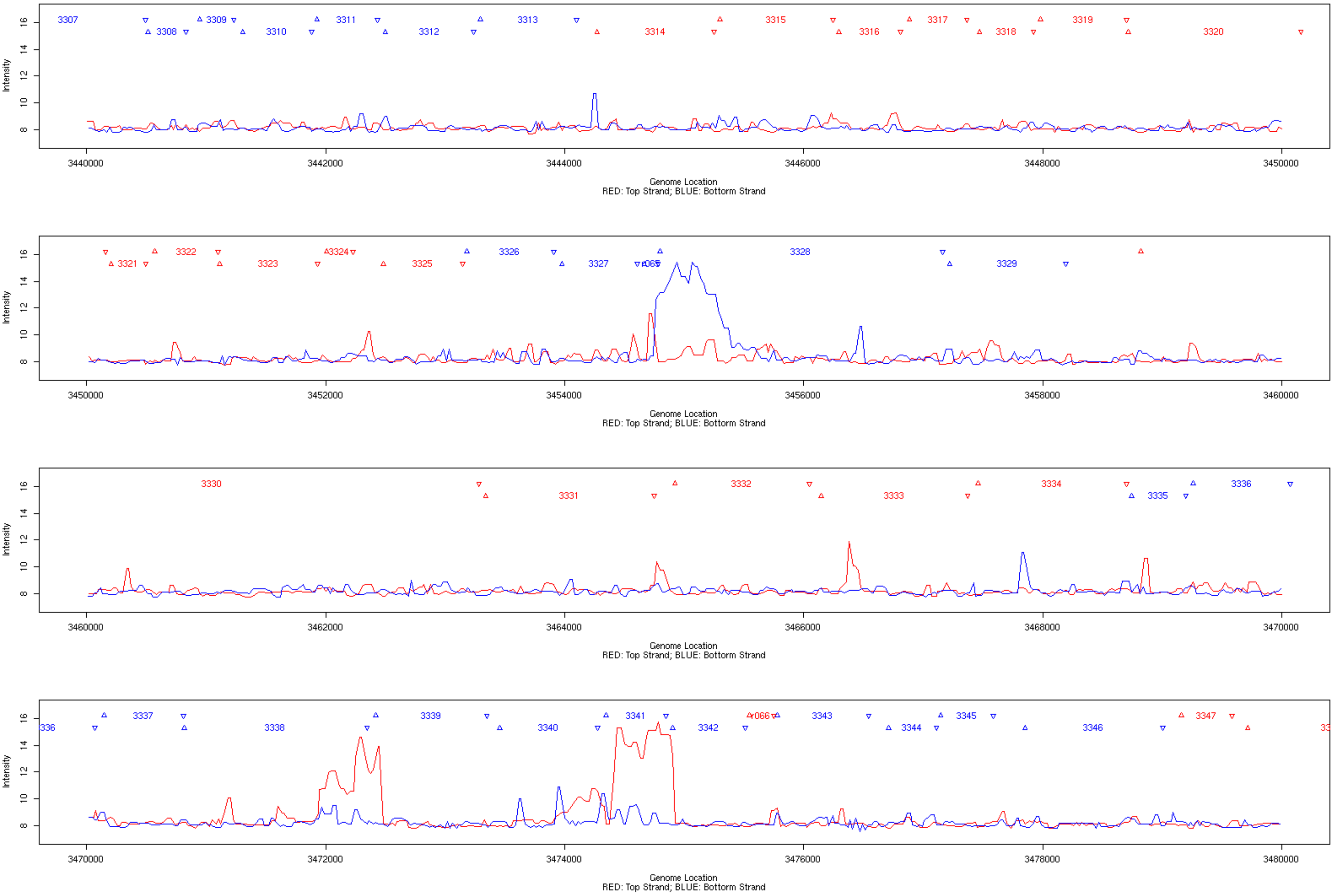
Intensity
Genome Location
RED: Top Strand; BLUE: Bottorm Strand
3307 3308 3309 3310 3311 3312 3313 3314 3315 3316 3317 3318 3319 3320
3321 3322 3323 3324 3325 3326 3327 3328 3329
3330 3331 3332 3333 3334 3335 3336
3337 3338 3339 3340 3341 3342 3343 3344 3345 3346 3347
3440000 3442000 3444000 3446000 3448000 3450000
3450000 3452000 3454000 3456000 3458000 3460000
3460000 3462000 3464000 3466000 3468000 3470000
3470000 3472000 3474000 3476000 3478000 3480000

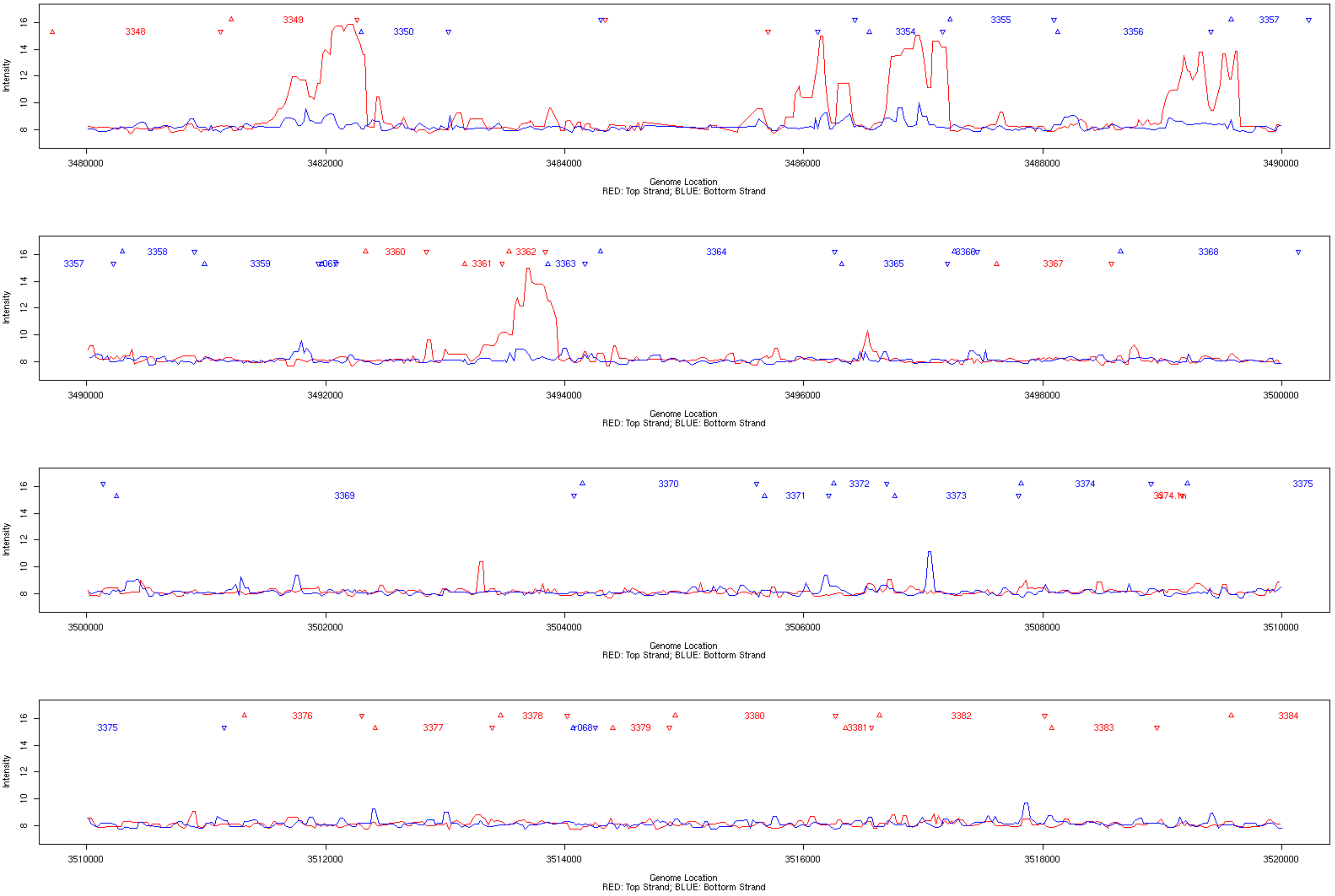
Intensity
3348
3349
3350
3354
3355
3356
3357
Genome Location
RED: Top Strand; BLUE: Bottorm Strand
3358
3359
3360
3361
3362
3363
3364
3365
3367
3368
3369
3370
3371
3372
3373
3374
3375
3376
3377
3378
3379
3380
3381
3382
3383
3384

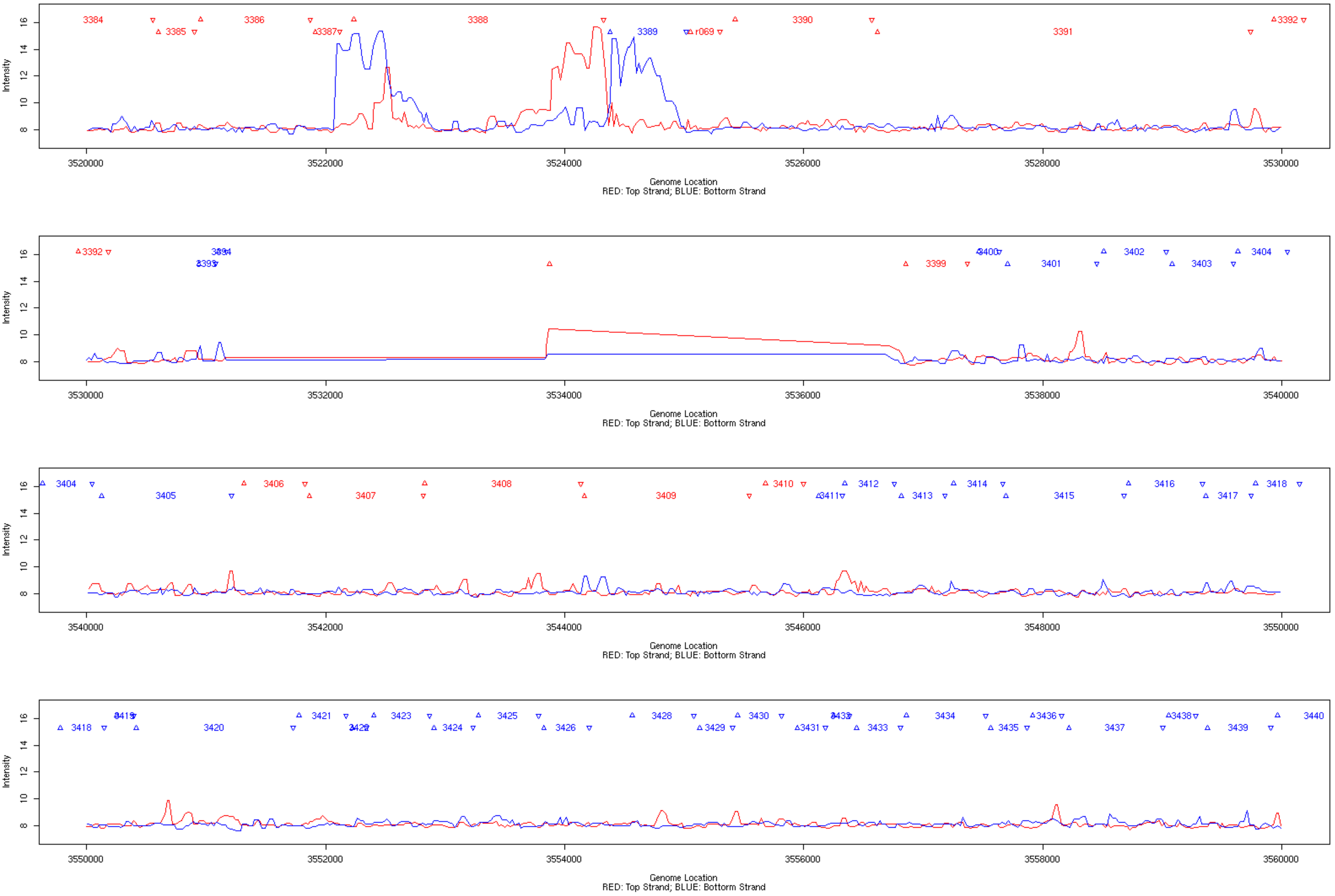
Intensity
Genome Location
RED: Top Strand; BLUE: Bottom Strand
3384
3385
3386
3387
3388
3389
r069
3390
3391
3392
3399
3400
3401
3402
3403
3404
3405
3406
3407
3408
3409
3410
3411
3412
3413
3414
3415
3416
3417
3418
3420
3421
3423
3424
3425
3426
3428
3429
3430
3431
3433
3434
3435
3436
3437
3438
3439
3440

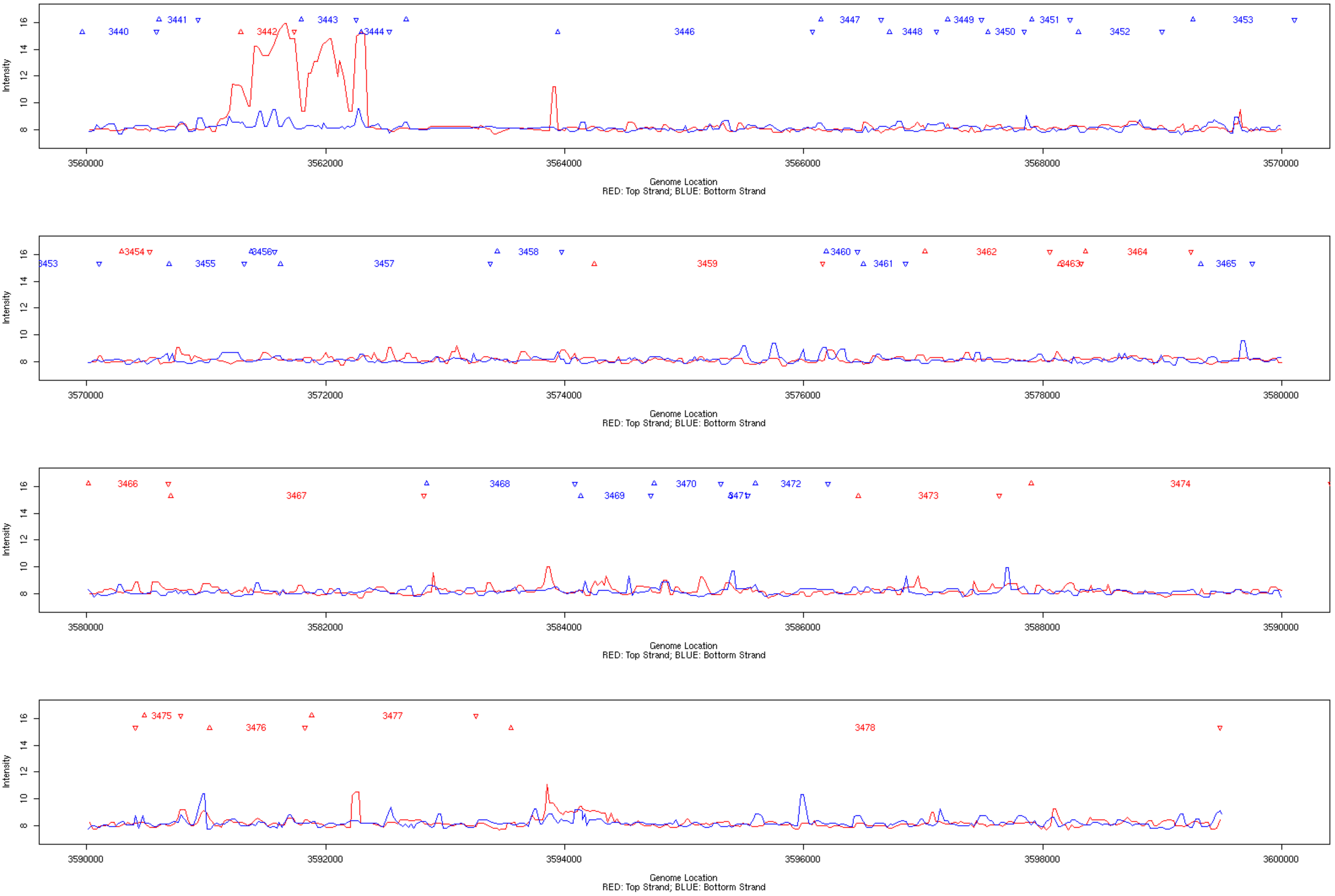

Intensity
3440
3441
3442
3443
3444
3446
3447
3448
3449
3450
3451
3452
3453
Genome Location
RED: Top Strand; BLUE: Bottorm Strand
3454
3455
3456
3457
3458
3459
3460
3461
3462
3463
3464
3465
3466
3467
3468
3469
3470
3472
3473
3474
3475
3476
3477
3478

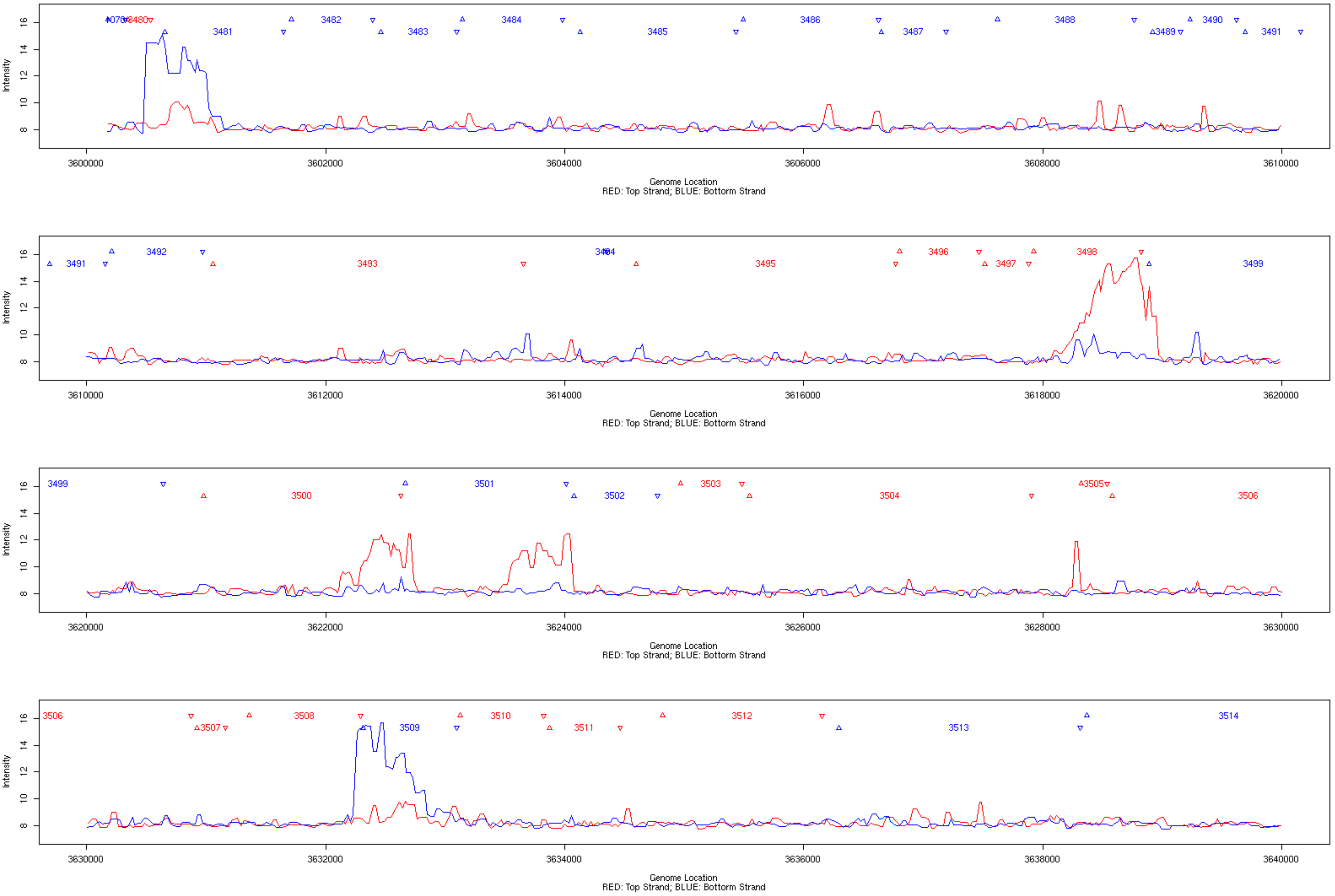
Intensity
Genome Location
RED: Top Strand; BLUE: Bottorm Strand
3481
3482
3483
3484
3485
3486
3487
3488
3489
3490
3491
3492
3493
3495
3496
3497
3498
3499
3500
3501
3502
3503
3504
3505
3506
3507
3508
3509
3510
3511
3512
3513
3514

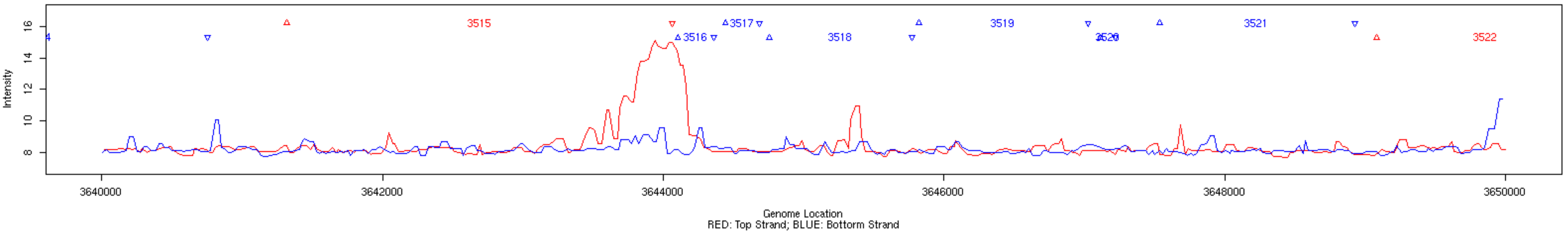
Intensity
16
14
12
10
8
3515
3516
3517
3518
3519
3521
3522
3640000
3642000
3644000
3646000
3648000
3650000
Genome Location
RED: Top Strand; BLUE: Bottorm Strand

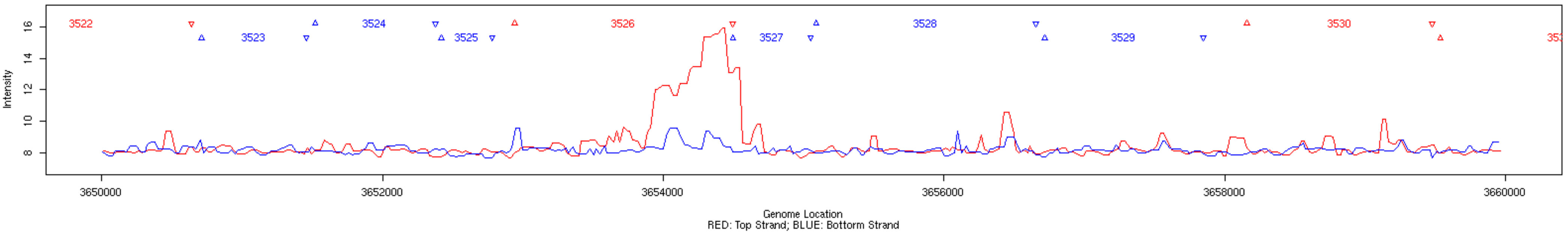
Intensity
16
14
12
10
8
3522
3523
3524
3525
3526
3527
3528
3529
3530
3650000
3652000
3654000
3656000
3658000
3660000
Genome Location
RED: Top Strand; BLUE: Bottorm Strand

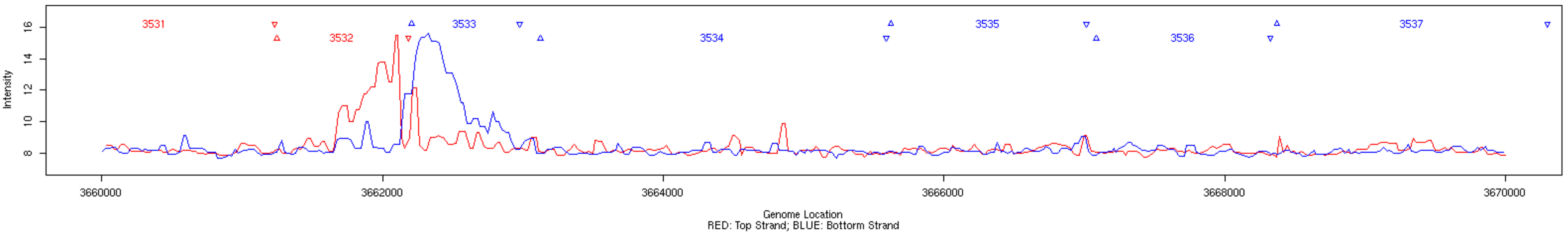
Intensity
16
14
12
10
8
3531
3532
3533
3534
3535
3536
3537
3660000
3662000
3664000
3666000
3668000
3670000
Genome Location
RED: Top Strand; BLUE: Bottorm Strand

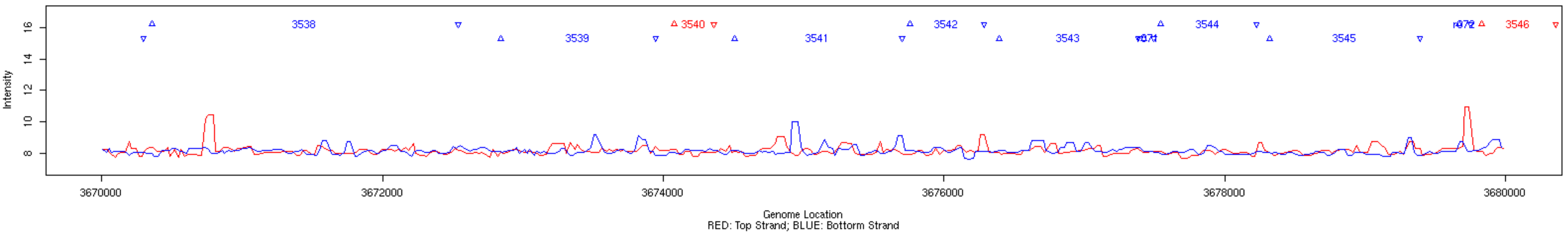
Intensity
16
14
12
10
8
3538
3539
3540
3541
3542
3543
3544
3545
3546
3670000
3672000
3674000
3676000
3678000
3680000
Genome Location
RED: Top Strand; BLUE: Bottorm Strand

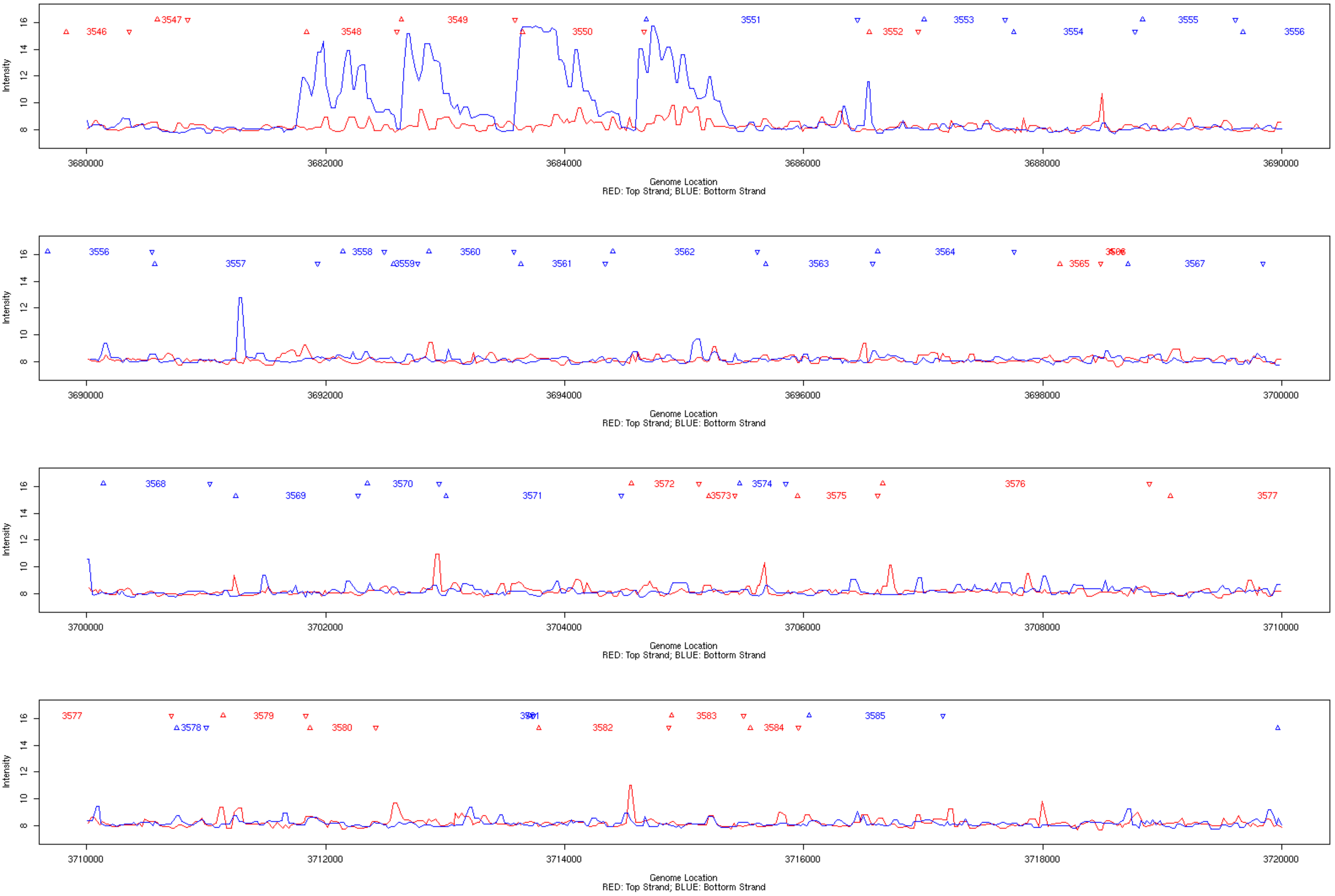
Intensity
Genome Location
RED: Top Strand; BLUE: Bottorm Strand
3680000
3682000
3684000
3686000
3688000
3690000
3692000
3694000
3696000
3698000
3700000
3702000
3704000
3706000
3708000
3710000
3712000
3714000
3716000
3718000
3720000
3546
3547
3548
3549
3550
3551
3552
3553
3554
3555
3556
3557
3558
3559
3560
3561
3562
3563
3564
3565
3567
3568
3569
3570
3571
3572
3573
3574
3575
3576
3577
3578
3579
3580
3582
3583
3584
3585

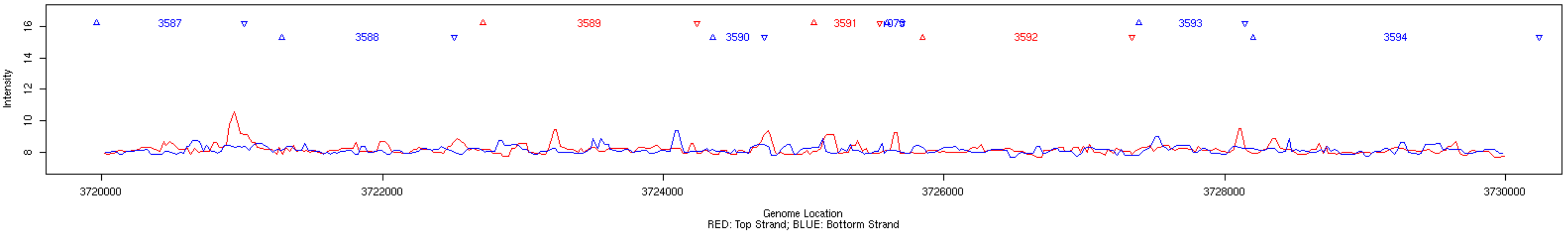
3587
3588
3589
3590
3591
3592
3593
3594
Intensity
Genome Location
RED: Top Strand; BLUE: Bottorm Strand

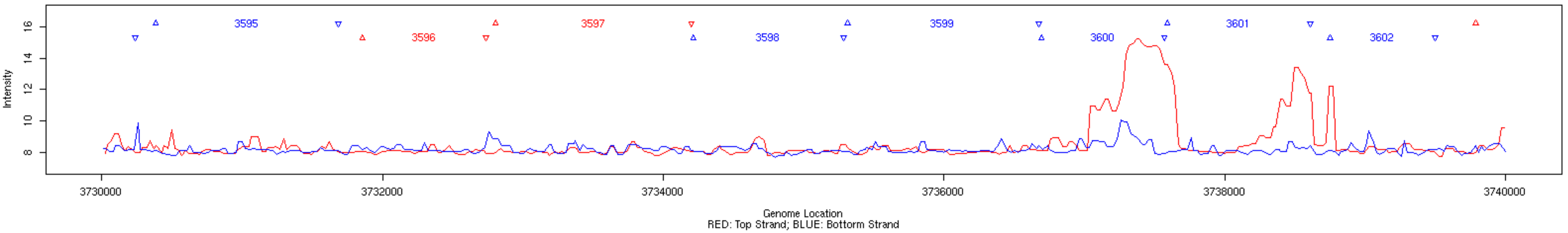
3595
3596
3597
3598
3599
3600
3601
3602
Intensity
Genome Location
RED: Top Strand; BLUE: Bottorm Strand

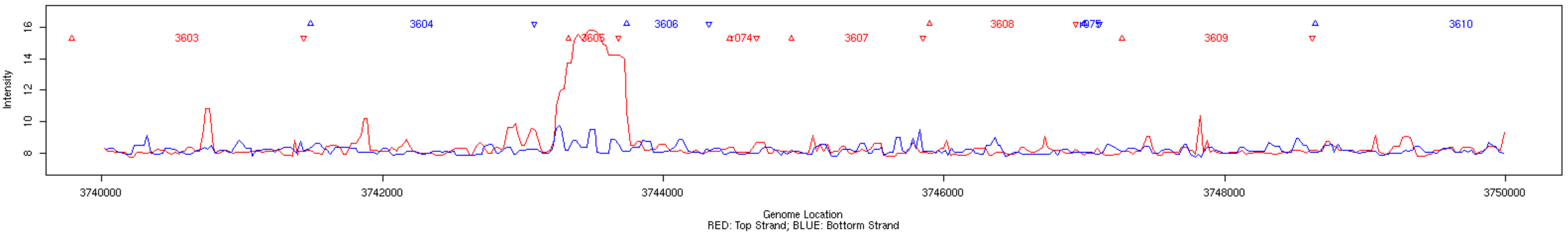
3603
3604
3605
3606
3607
3608
3609
3610
Intensity
Genome Location
RED: Top Strand; BLUE: Bottorm Strand

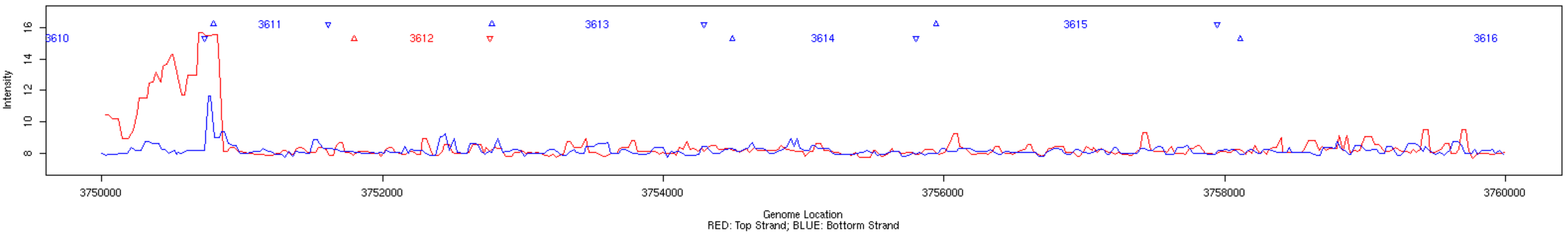
3610
3611
3612
3613
3614
3615
3616
Intensity
Genome Location
RED: Top Strand; BLUE: Bottorm Strand

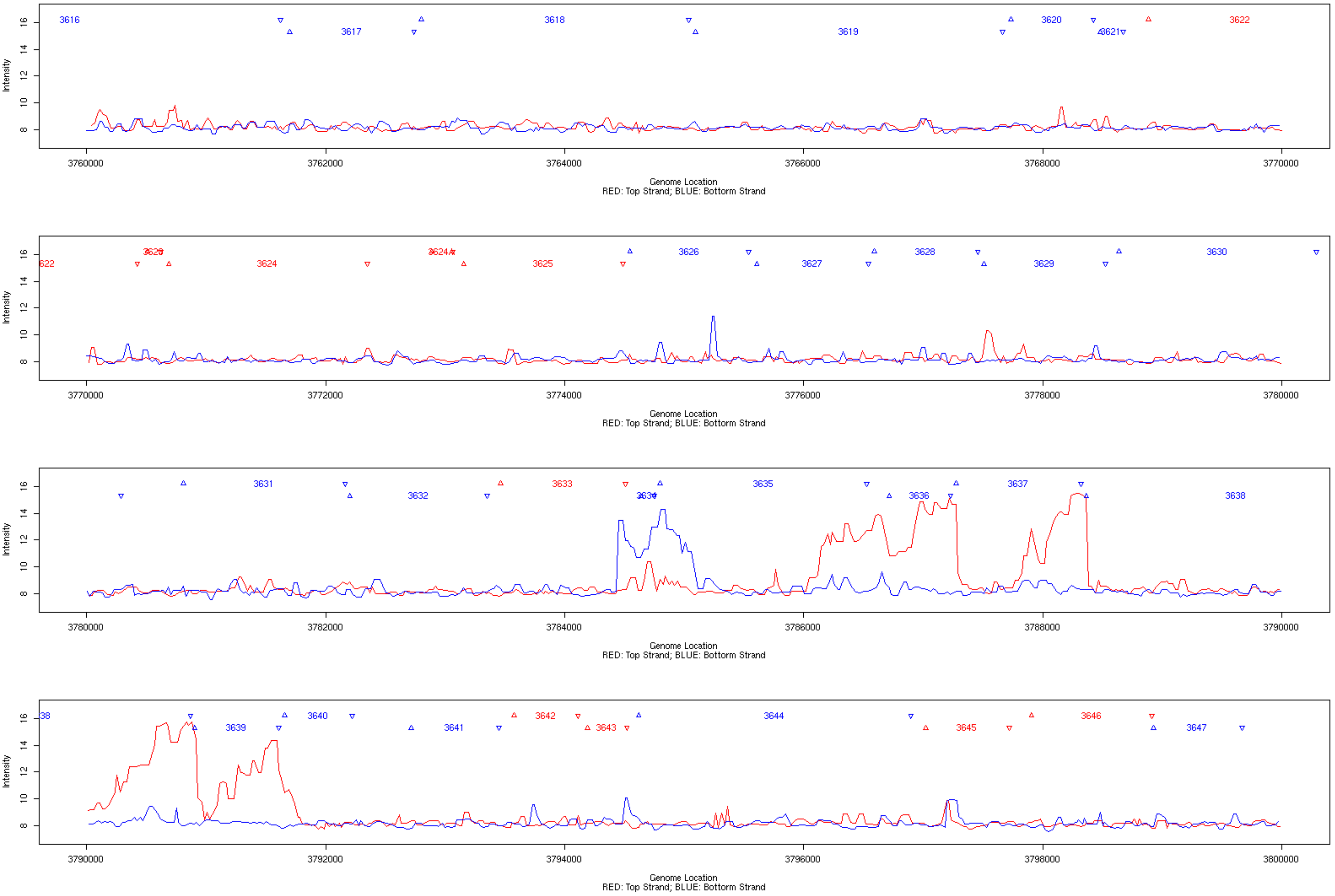
3616
3617
3618
3619
3620
3621
3622
3623
3624
3625
3626
3627
3628
3629
3630
3631
3632
3633
3634
3635
3636
3637
3638
3639
3640
3641
3642
3643
3644
3645
3646
3647
Intensity
Genome Location
RED: Top Strand; BLUE: Bottom Strand

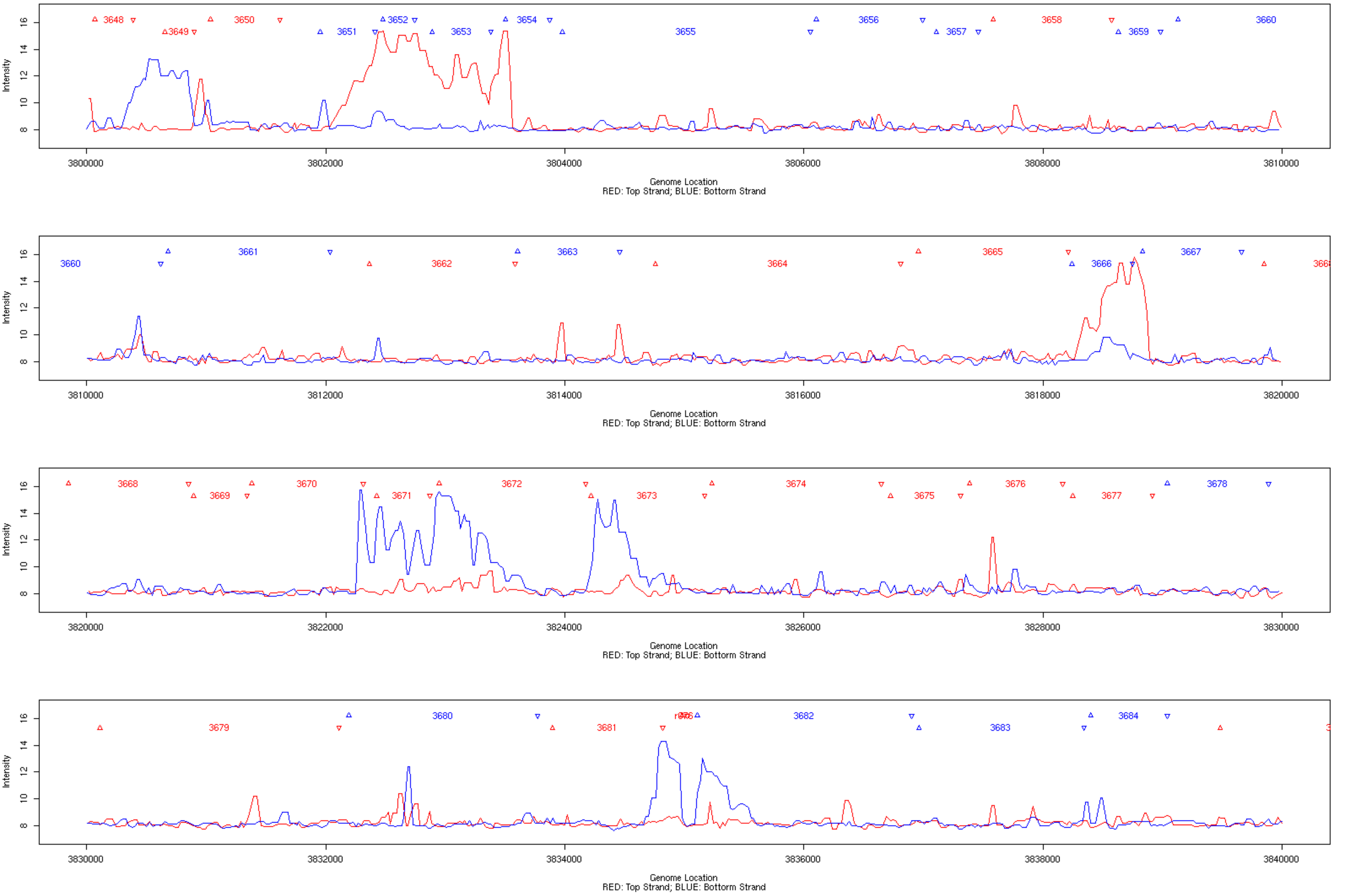
Intensity
Genome Location
RED: Top Strand; BLUE: Bottom Strand
3800000
3802000
3804000
3806000
3808000
3810000
3812000
3814000
3816000
3818000
3820000
3822000
3824000
3826000
3828000
3830000
3832000
3834000
3836000
3838000
3840000

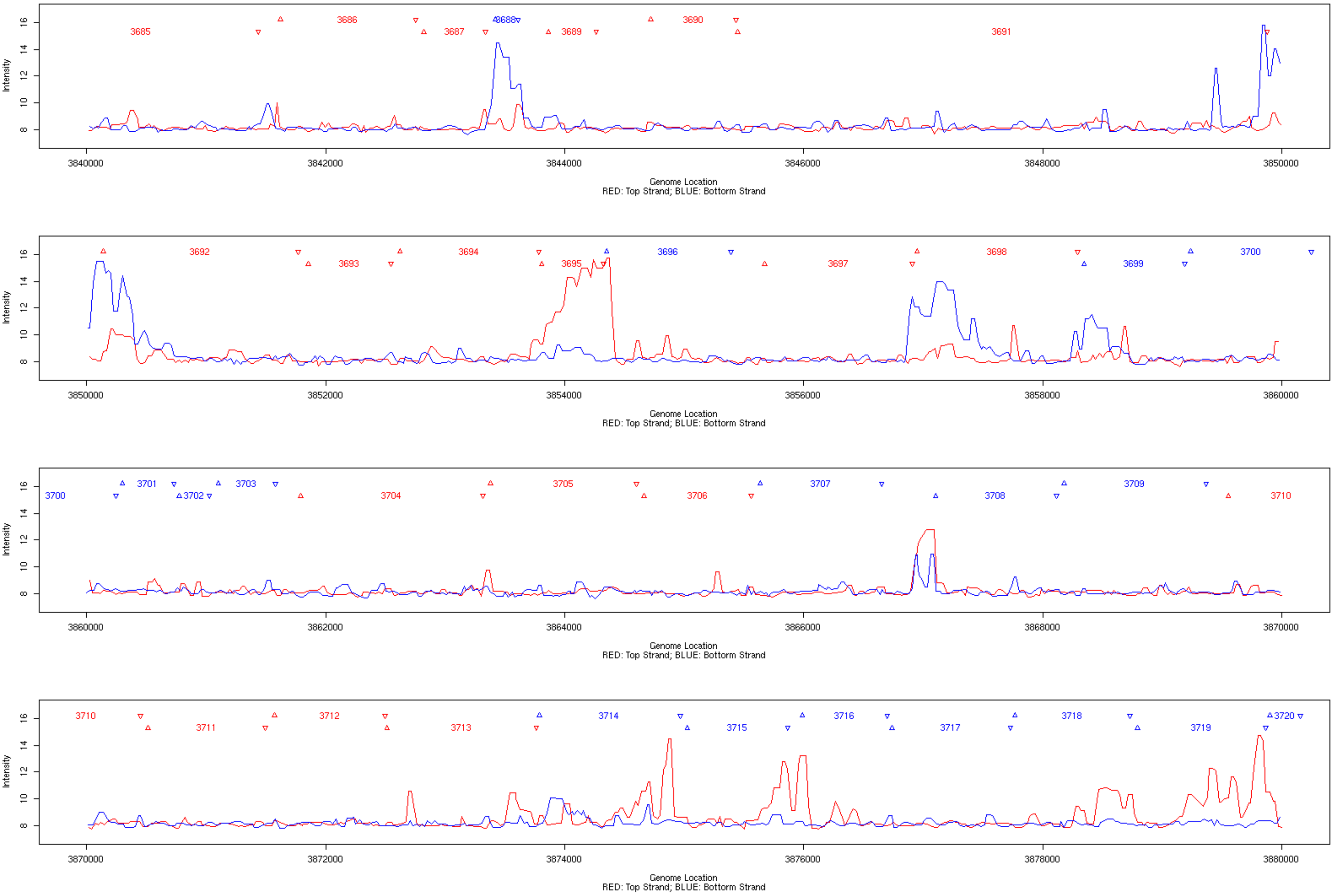

Intensity
3685
3686
3687
3688
3689
3690
3691
3840000
3842000
3844000
3846000
3848000
3850000
Genome Location
RED: Top Strand; BLUE: Bottorm Strand
3692
3693
3694
3695
3696
3697
3698
3699
3700
3850000
3852000
3854000
3856000
3858000
3860000
Genome Location
RED: Top Strand; BLUE: Bottorm Strand
3700
3701
3702
3703
3704
3705
3706
3707
3708
3709
3710
3860000
3862000
3864000
3866000
3868000
3870000
Genome Location
RED: Top Strand; BLUE: Bottorm Strand
3710
3711
3712
3713
3714
3715
3716
3717
3718
3719
3720
3870000
3872000
3874000
3876000
3878000
3880000
Genome Location
RED: Top Strand; BLUE: Bottorm Strand

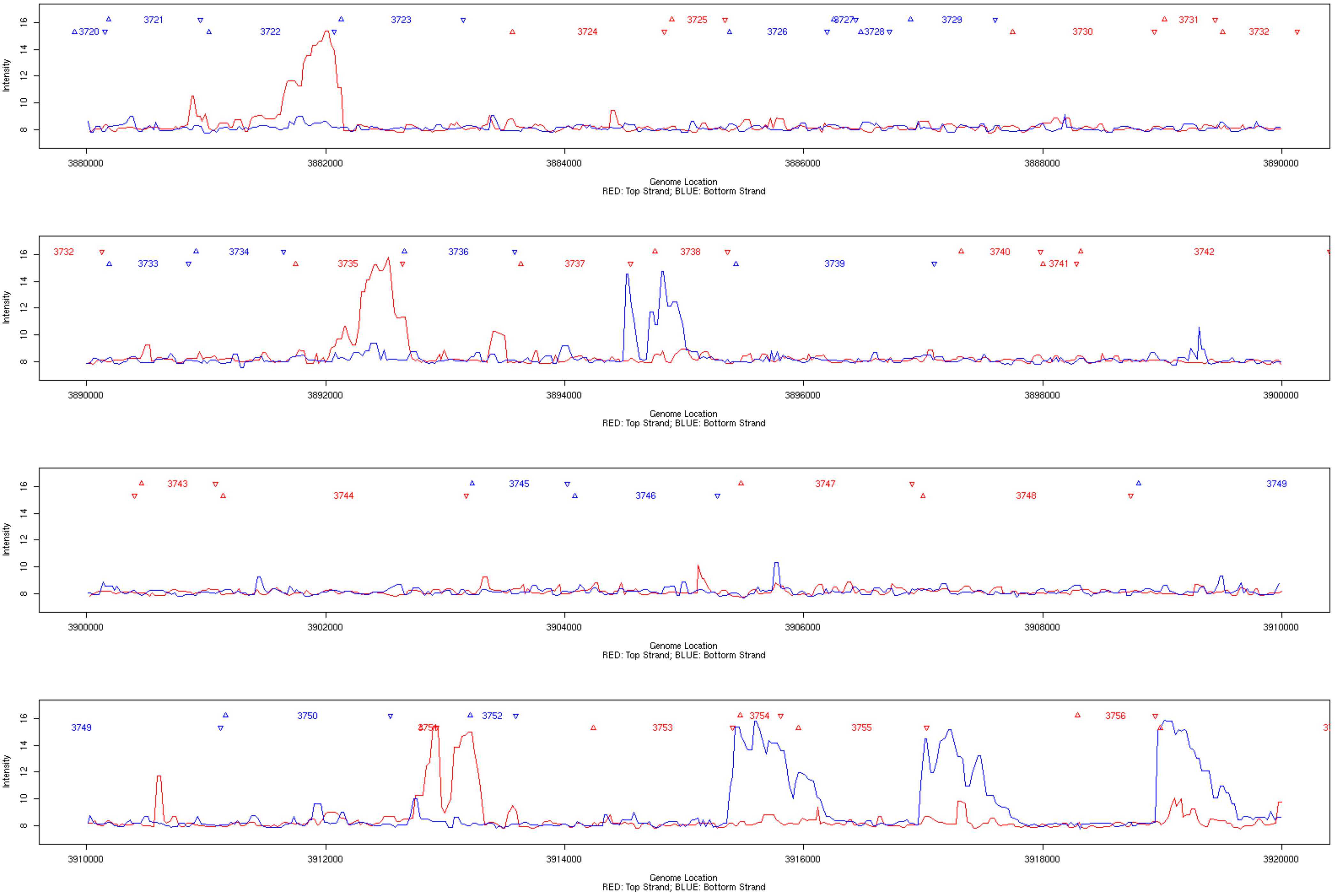
Intensity
3720 3721 3722 3723 3724 3725 3726 3727 3728 3729 3730 3731 3732
3880000 3882000 3884000 3886000 3888000 3890000
Genome Location
RED: Top Strand; BLUE: Bottorm Strand
Intensity
3732 3733 3734 3735 3736 3737 3738 3739 3740 3741 3742
3890000 3892000 3894000 3896000 3898000 3900000
Genome Location
RED: Top Strand; BLUE: Bottorm Strand
Intensity
3743 3744 3745 3746 3747 3748 3749
3900000 3902000 3904000 3906000 3908000 3910000
Genome Location
RED: Top Strand; BLUE: Bottorm Strand
Intensity
3749 3750 3752 3753 3754 3755 3756
3910000 3912000 3914000 3916000 3918000 3920000
Genome Location
RED: Top Strand; BLUE: Bottorm Strand

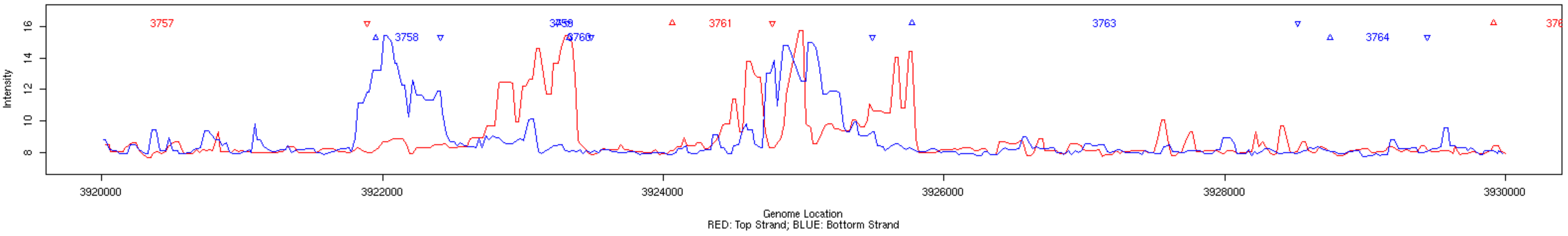
Intensity
3757
3758
3761
3763
3764
3920000
3922000
3924000
3926000
3928000
3930000
Genome Location
RED: Top Strand; BLUE: Bottorm Strand

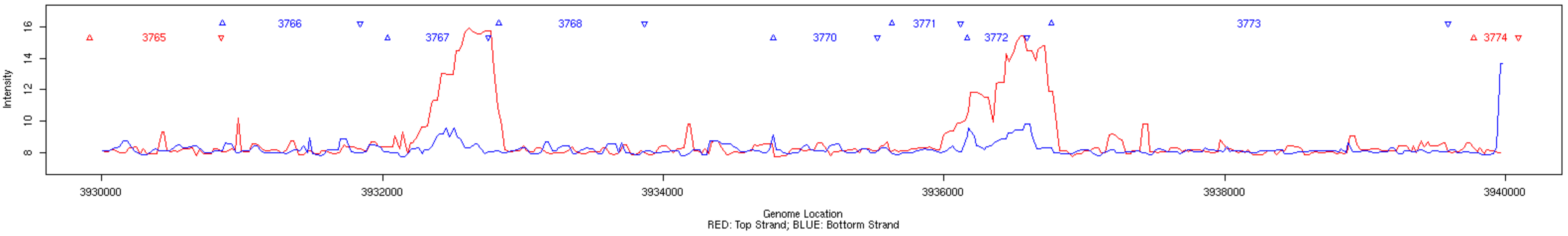
Intensity
3765
3766
3767
3768
3770
3771
3772
3773
3774
3930000
3932000
3934000
3936000
3938000
3940000
Genome Location
RED: Top Strand; BLUE: Bottorm Strand

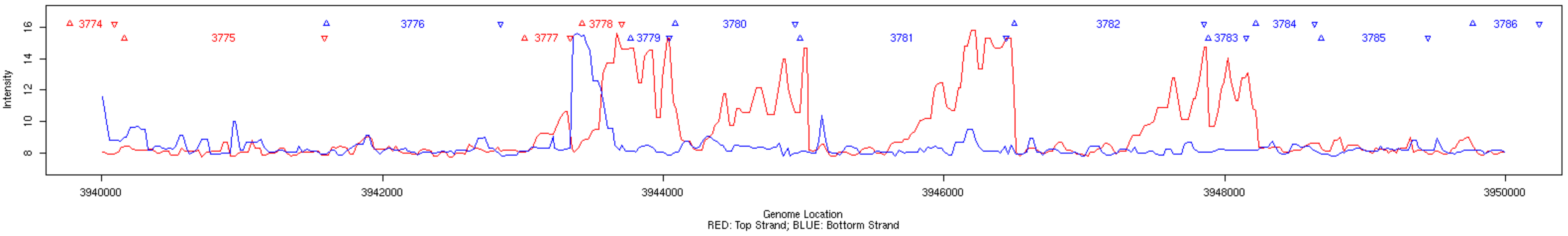
Intensity
3774
3775
3776
3777
3778
3779
3780
3781
3782
3783
3784
3785
3786
3940000
3942000
3944000
3946000
3948000
3950000
Genome Location
RED: Top Strand; BLUE: Bottorm Strand

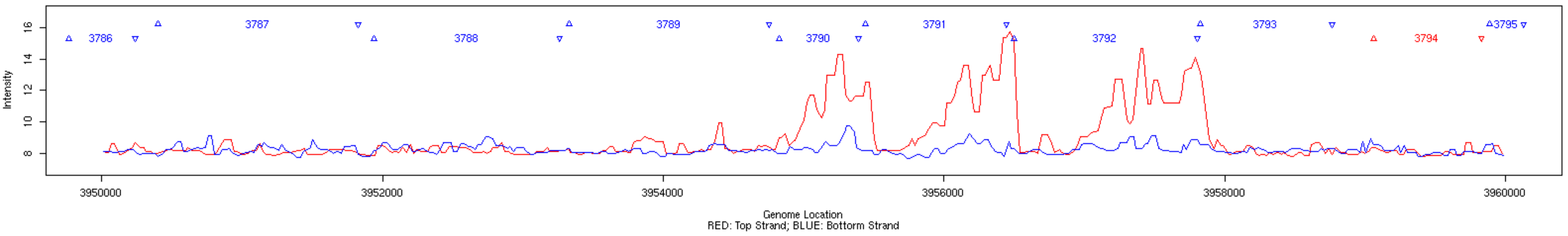
Intensity
3786
3787
3788
3789
3790
3791
3792
3793
3794
3795
3950000
3952000
3954000
3956000
3958000
3960000
Genome Location
RED: Top Strand; BLUE: Bottorm Strand

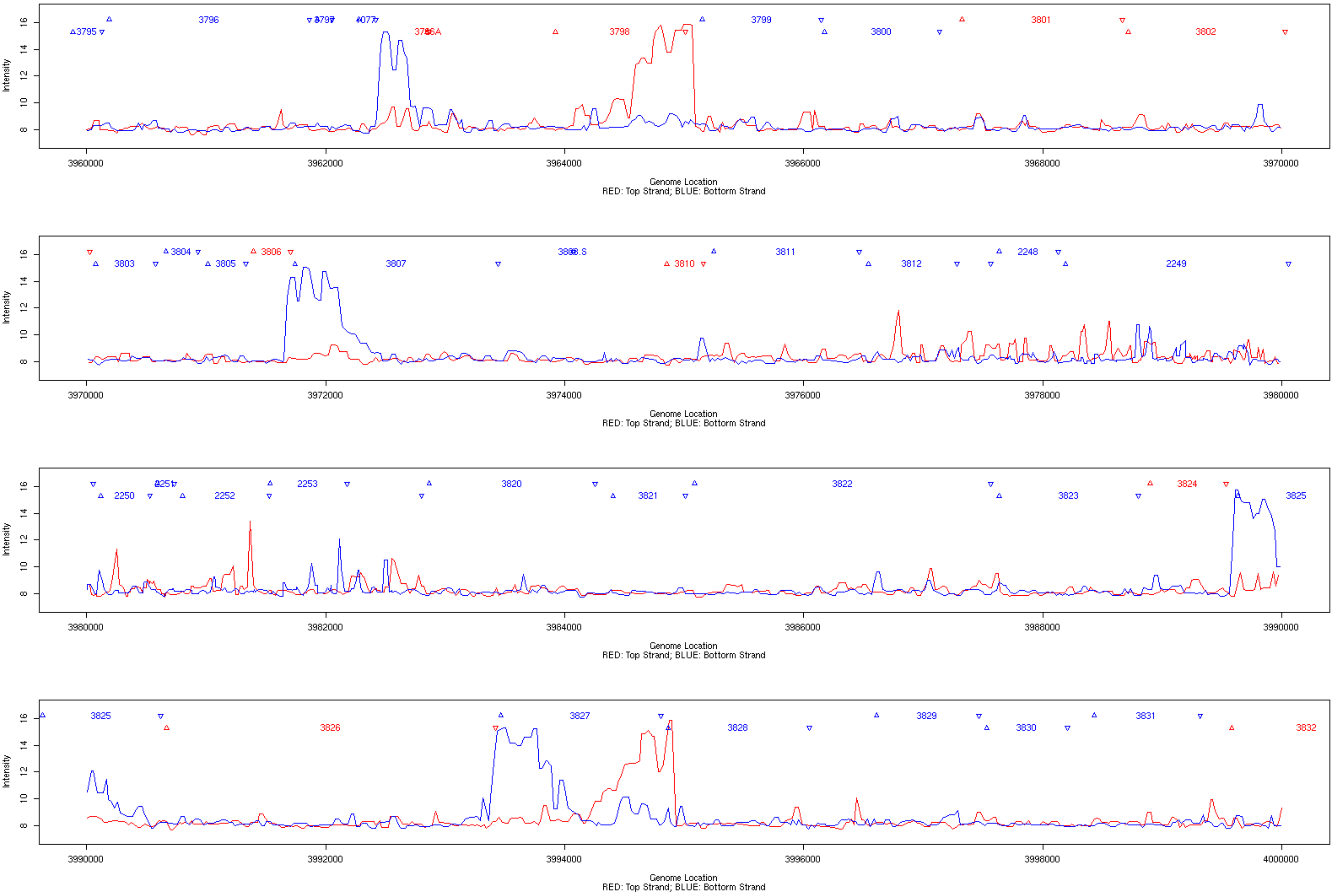
Intensity
Genome Location
RED: Top Strand; BLUE: Bottorm Strand
3795
3796
3799
3800
3801
3802
3798
3803
3804
3805
3806
3807
3810
3811
3812
2248
2249
2250
2252
2253
3820
3821
3822
3823
3824
3825
3826
3827
3828
3829
3830
3831
3832

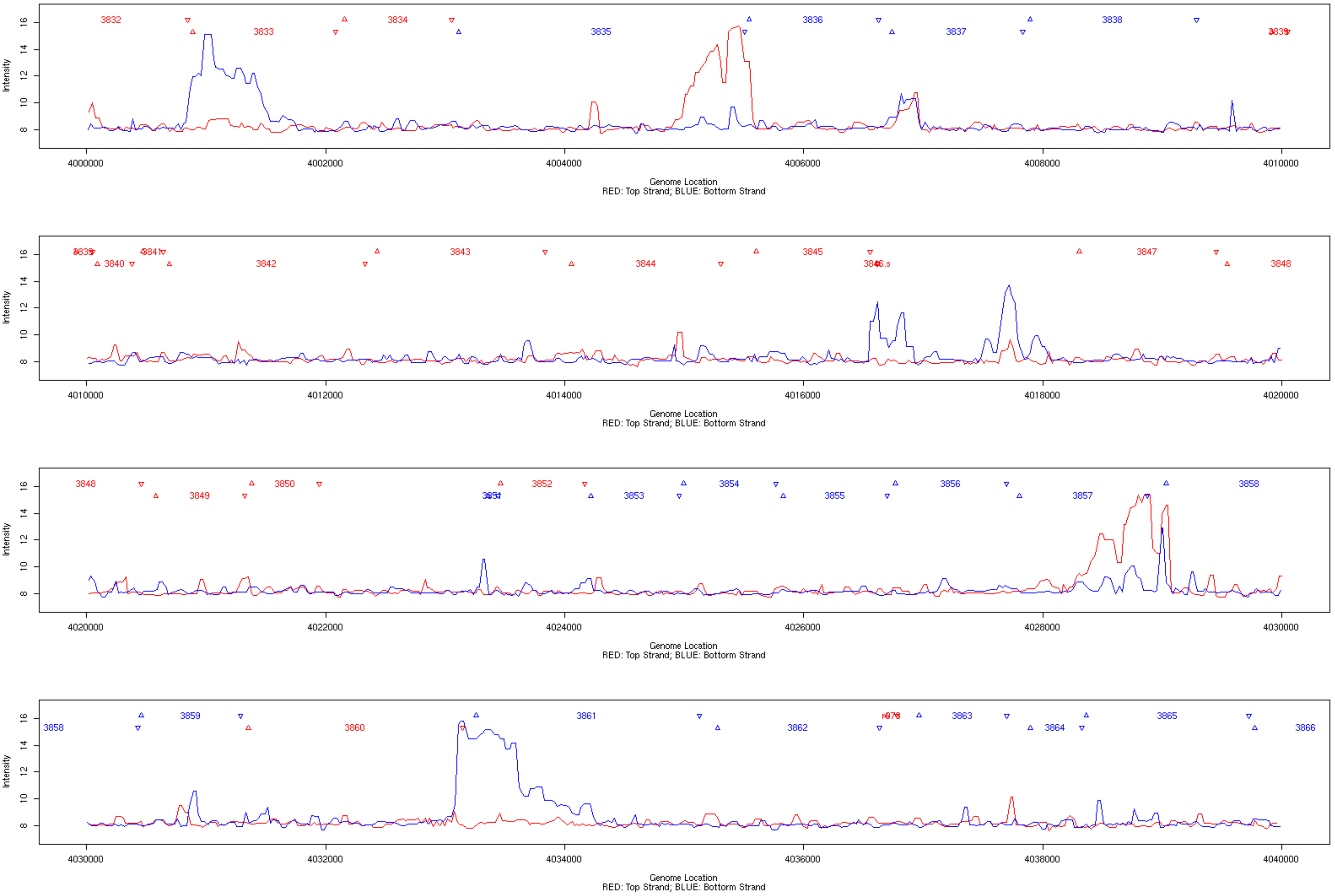

Intensity
Genome Location
RED: Top Strand; BLUE: Bottom Strand
4000000
4002000
4004000
4006000
4008000
4010000
4012000
4014000
4016000
4018000
4020000
4022000
4024000
4026000
4028000
4030000
4032000
4034000
4036000
4038000
4040000
3832
3833
3834
3835
3836
3837
3838
3840
3842
3843
3844
3845
3847
3848
3849
3850
3852
3853
3854
3855
3856
3857
3858
3859
3860
3861
3862
3863
3864
3865
3866

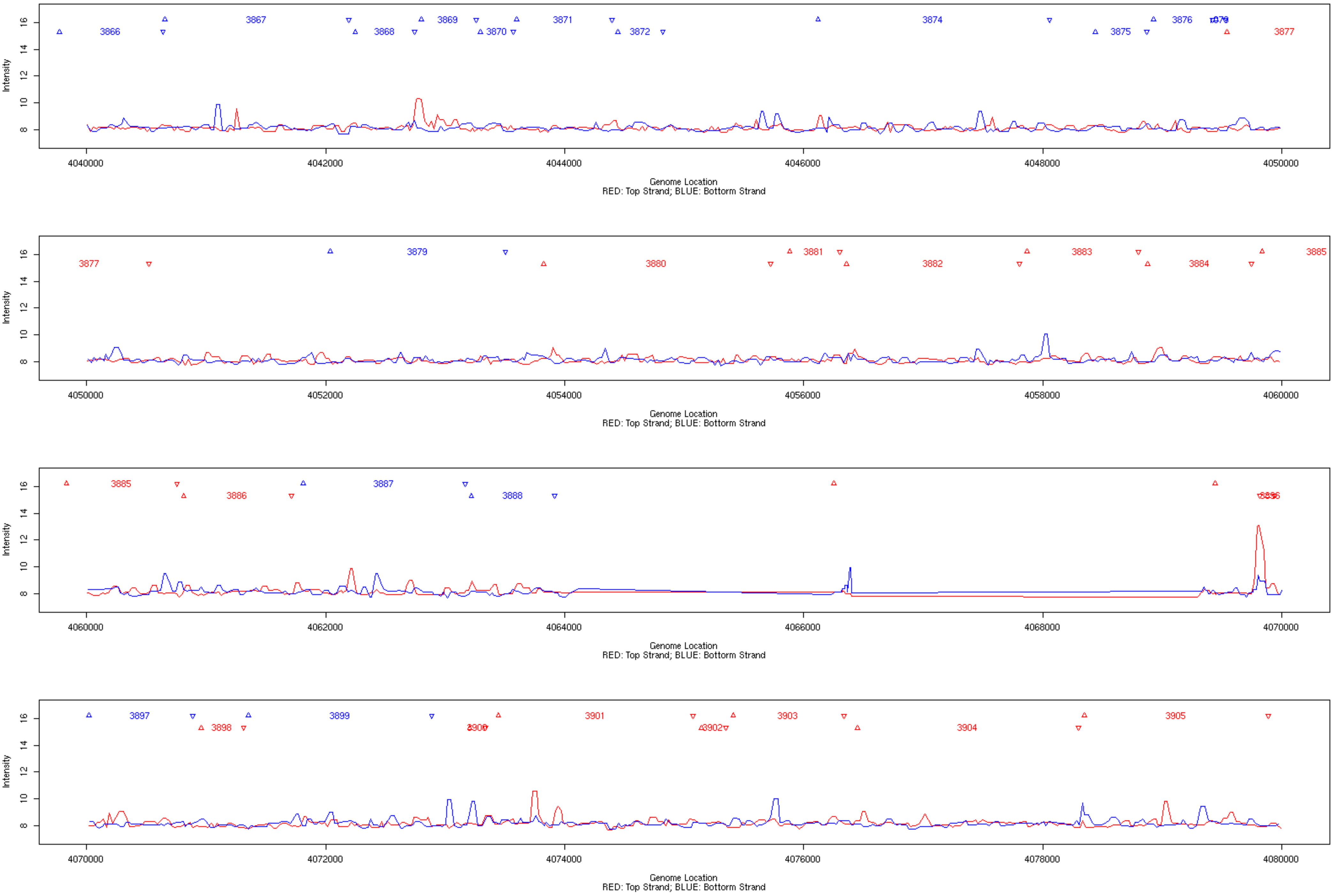

Intensity
3866
3867
3868
3869
3870
3871
3872
3874
3875
3876
3877
4040000
4042000
4044000
4046000
4048000
4050000
Genome Location
RED: Top Strand; BLUE: Bottorm Strand
3877
3879
3880
3881
3882
3883
3884
3885
4050000
4052000
4054000
4056000
4058000
4060000
Genome Location
RED: Top Strand; BLUE: Bottorm Strand
3885
3886
3887
3888
4060000
4062000
4064000
4066000
4068000
4070000
Genome Location
RED: Top Strand; BLUE: Bottorm Strand
3897
3898
3899
3901
3902
3903
3904
3905
4070000
4072000
4074000
4076000
4078000
4080000
Genome Location
RED: Top Strand; BLUE: Bottorm Strand

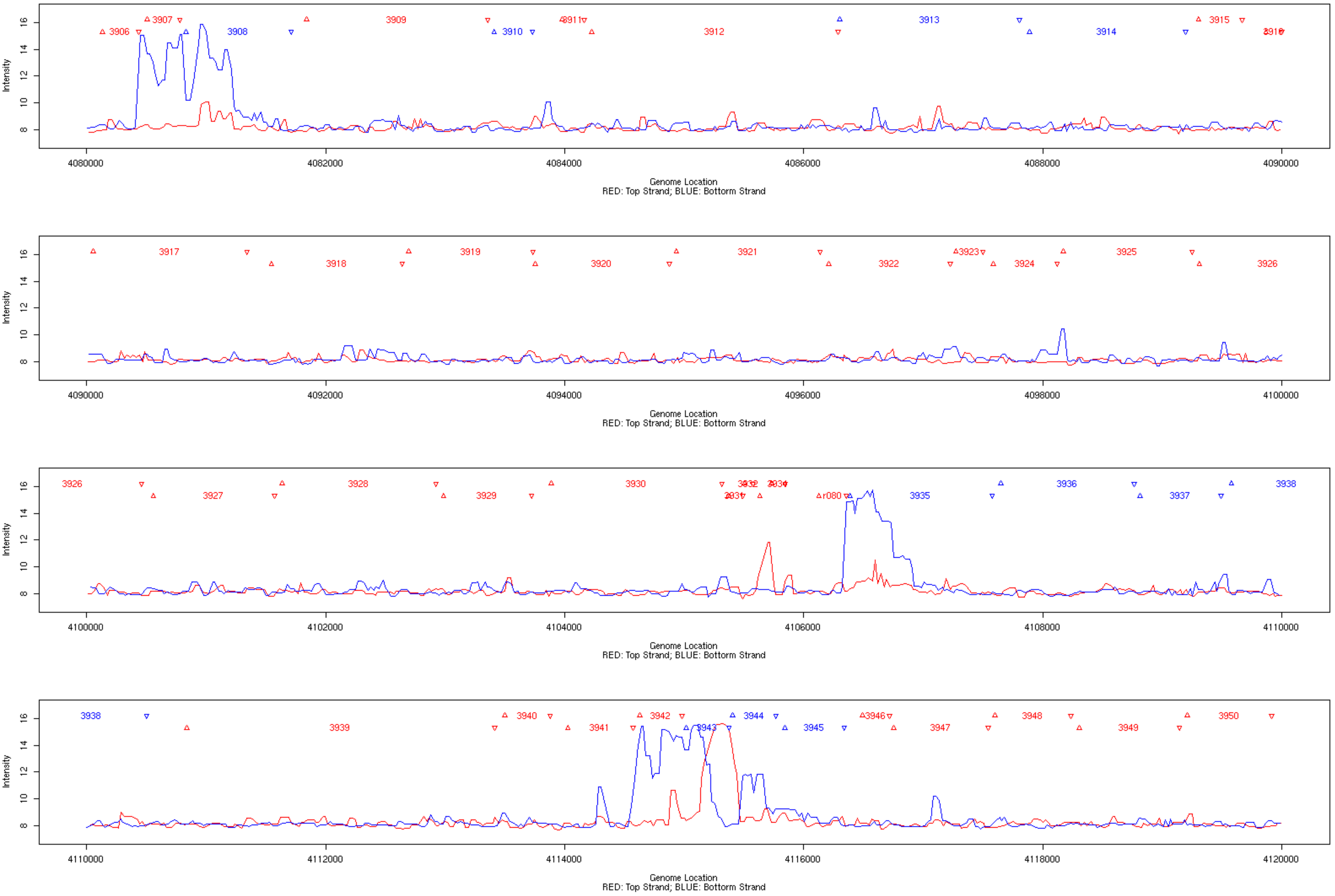
Intensity
Genome Location
RED: Top Strand; BLUE: Bottorm Strand
4080000
4082000
4084000
4086000
4088000
4090000
3906
3907
3908
3909
3910
3912
3913
3914
3915
4092000
4094000
4096000
4098000
4100000
3917
3918
3919
3920
3921
3922
3923
3924
3925
3926
4102000
4104000
4106000
4108000
4110000
3927
3928
3929
3930
3935
3936
3937
3938
4112000
4114000
4116000
4118000
4120000
3939
3940
3941
3942
3943
3944
3945
3946
3947
3948
3949
3950

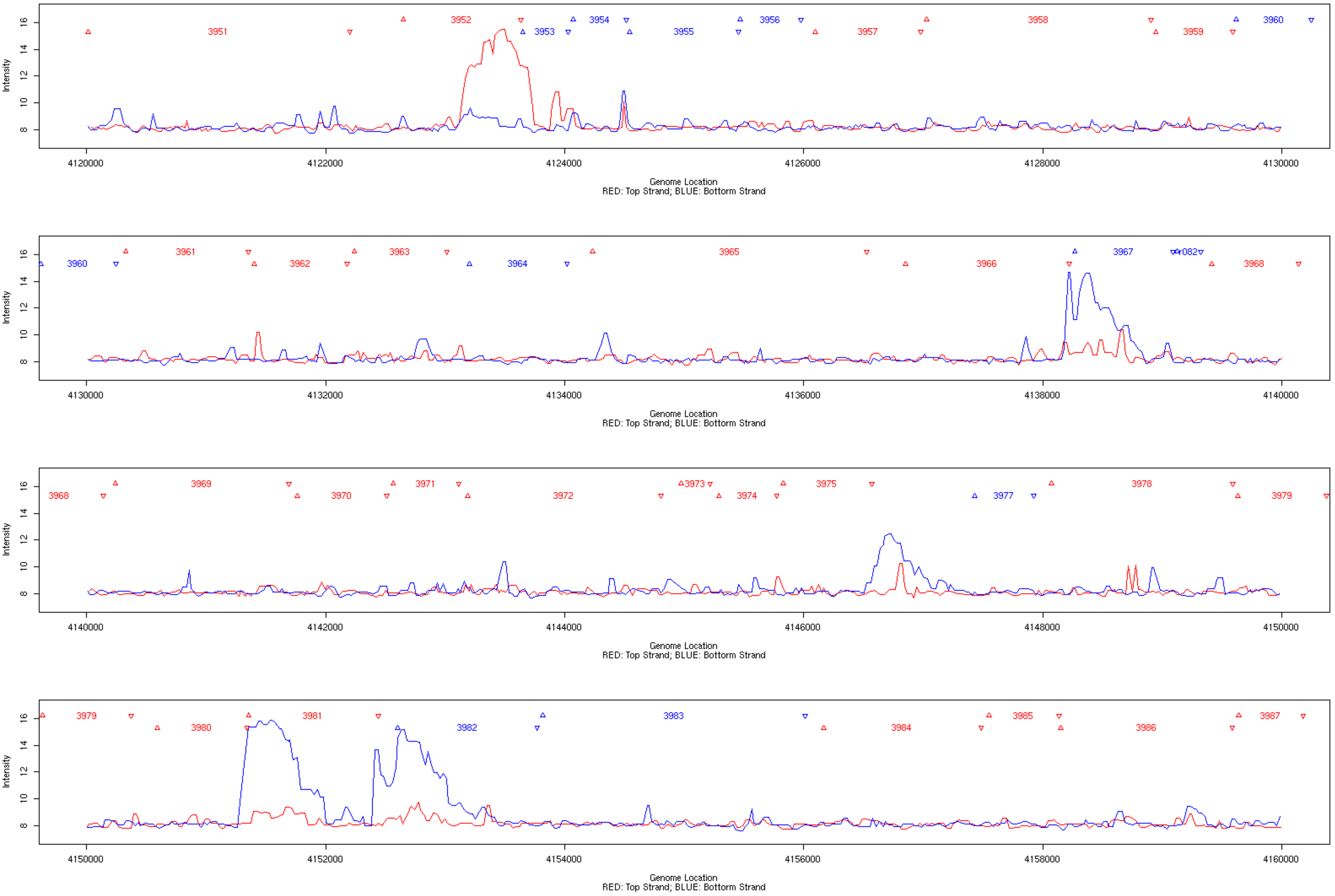

Intensity
3951
3952
3953
3954
3955
3956
3957
3958
3959
3960
4120000
4122000
4124000
4126000
4128000
4130000
Genome Location
RED: Top Strand; BLUE: Bottorm Strand
3960
3961
3962
3963
3964
3965
3966
3967
3968
4130000
4132000
4134000
4136000
4138000
4140000
3968
3969
3970
3971
3972
3973
3974
3975
3977
3978
3979
4140000
4142000
4144000
4146000
4148000
4150000
3979
3980
3981
3982
3983
3984
3985
3986
3987
4150000
4152000
4154000
4156000
4158000
4160000

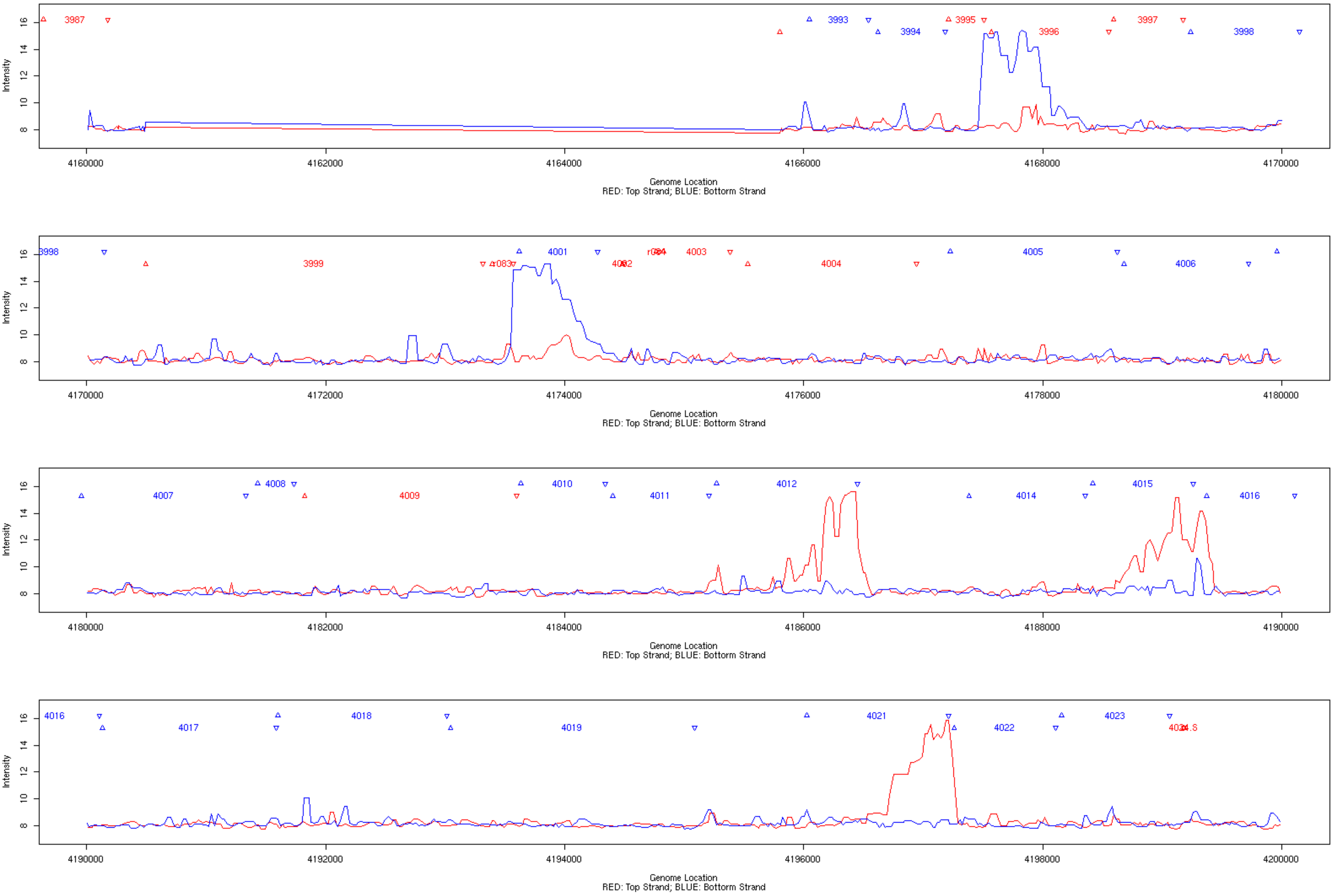
Intensity
3987
3993
3994
3995
3996
3997
3998
4160000
4162000
4164000
4166000
4168000
4170000
Genome Location
RED: Top Strand; BLUE: Bottorm Strand
3999
4001
4003
4004
4005
4006
4172000
4174000
4176000
4178000
4180000
4007
4008
4009
4010
4011
4012
4014
4015
4016
4182000
4184000
4186000
4188000
4190000
4017
4018
4019
4021
4022
4023
4192000
4194000
4196000
4198000
4200000

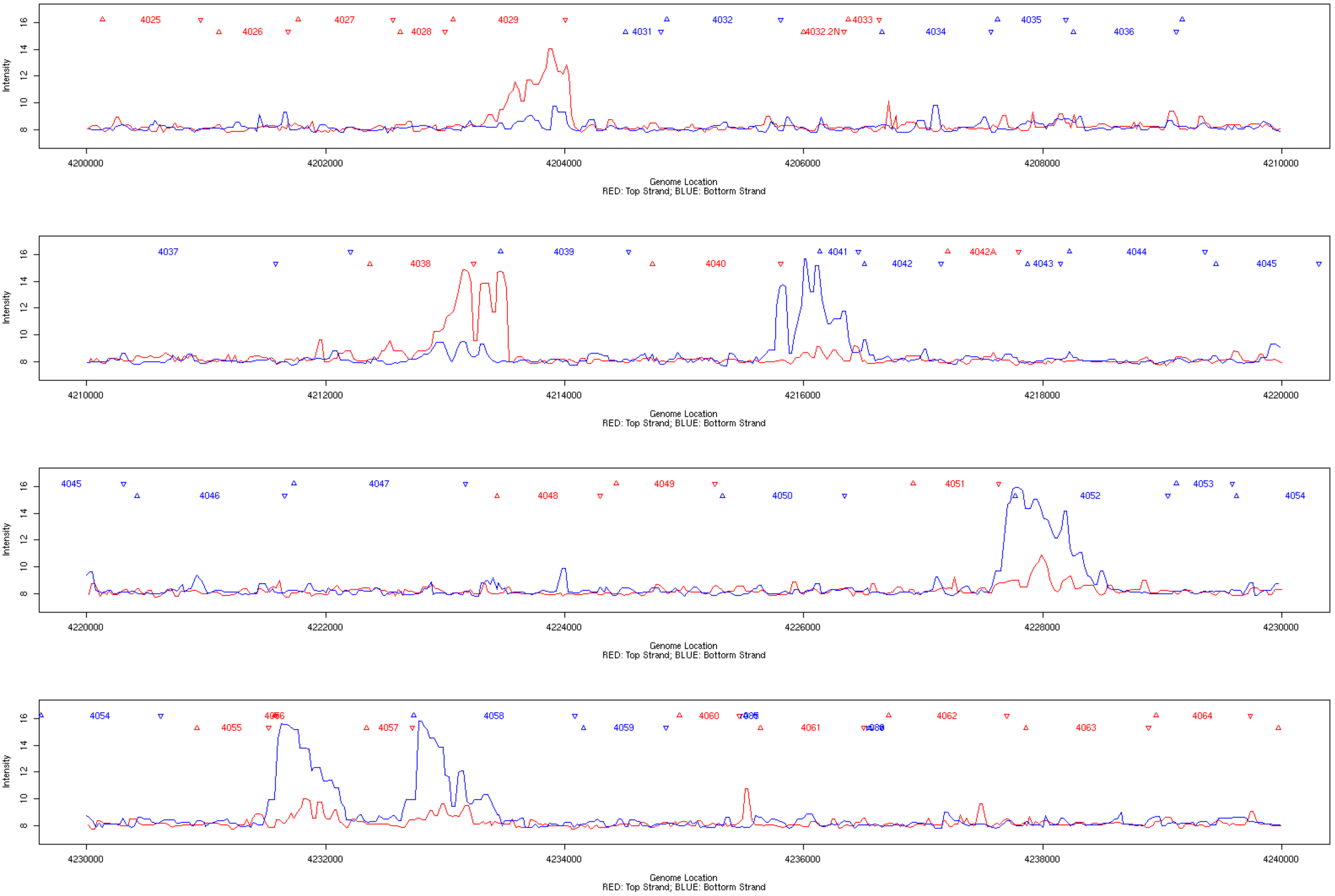
Intensity
4025
4026
4027
4028
4029
4031
4032
4032.2N
4033
4034
4035
4036
4200000
4202000
4204000
4206000
4208000
4210000
Genome Location
RED: Top Strand; BLUE: Bottorm Strand
4037
4038
4039
4040
4041
4042
4042A
4043
4044
4045
4210000
4212000
4214000
4216000
4218000
4220000
Genome Location
RED: Top Strand; BLUE: Bottorm Strand
4045
4046
4047
4048
4049
4050
4051
4052
4053
4054
4220000
4222000
4224000
4226000
4228000
4230000
Genome Location
RED: Top Strand; BLUE: Bottorm Strand
4054
4055
4057
4058
4059
4060
4061
4062
4063
4064
4230000
4232000
4234000
4236000
4238000
4240000
Genome Location
RED: Top Strand; BLUE: Bottorm Strand

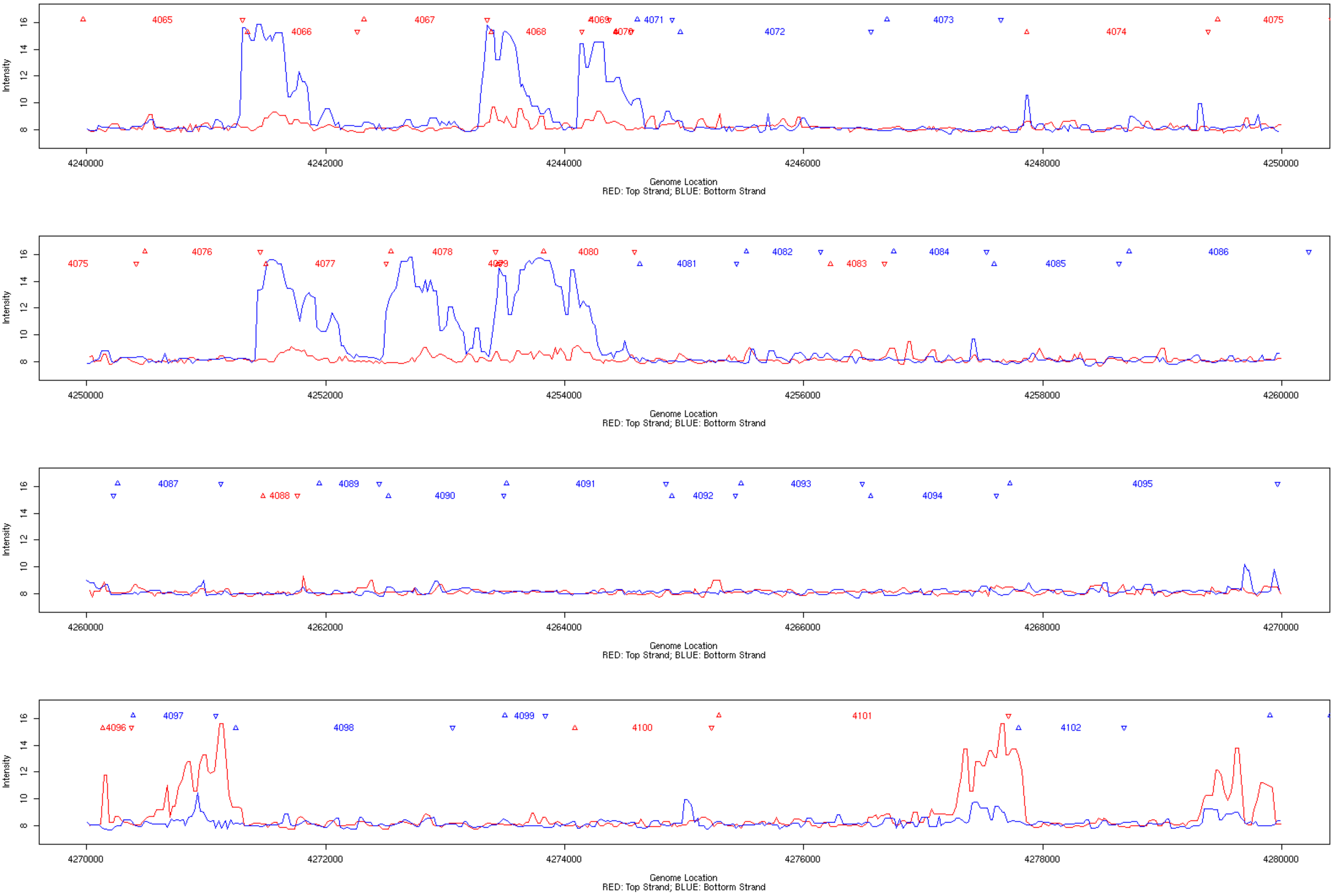

Intensity
4065
4066
4067
4068
4069
4070
4071
4072
4073
4074
4075
Genome Location
RED: Top Strand; BLUE: Bottorm Strand
4076
4077
4078
4079
4080
4081
4082
4083
4084
4085
4086
4087
4088
4089
4090
4091
4092
4093
4094
4095
4096
4097
4098
4099
4100
4101
4102

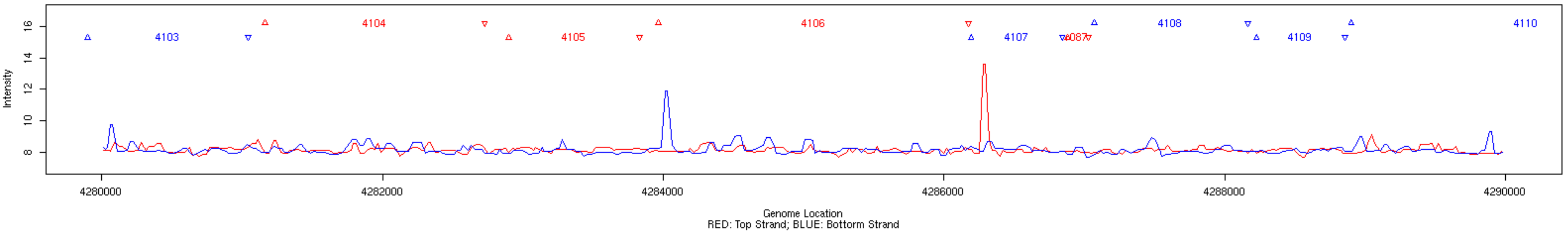
Intensity
4103
4104
4105
4106
4107
4108
4109
4110
Genome Location
RED: Top Strand; BLUE: Bottorm Strand

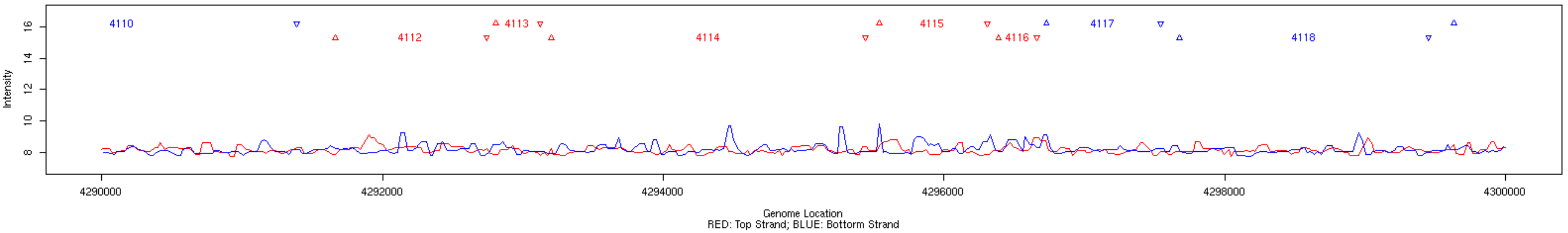
Intensity
4110
4112
4113
4114
4115
4116
4117
4118
Genome Location
RED: Top Strand; BLUE: Bottorm Strand

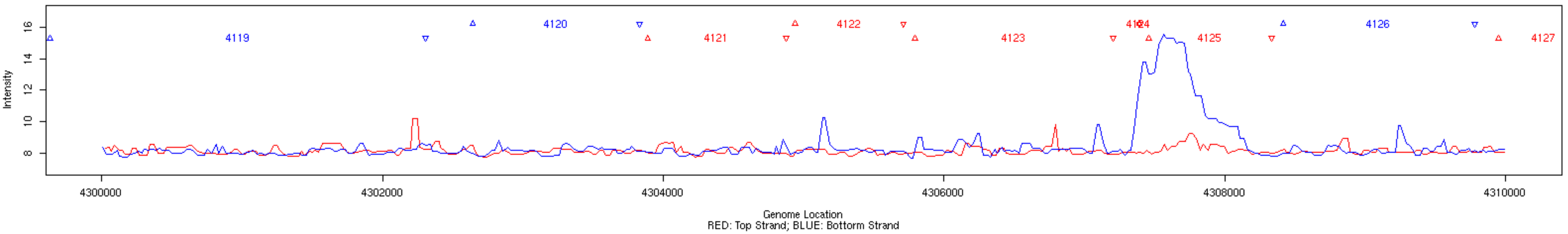
Intensity
4119
4120
4121
4122
4123
4125
4126
4127
Genome Location
RED: Top Strand; BLUE: Bottorm Strand

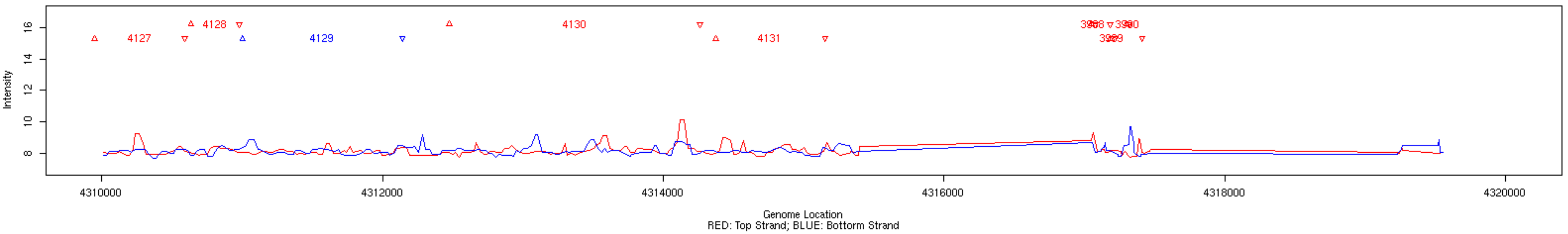
Intensity
4127
4128
4129
4130
4131
Genome Location
RED: Top Strand; BLUE: Bottorm Strand

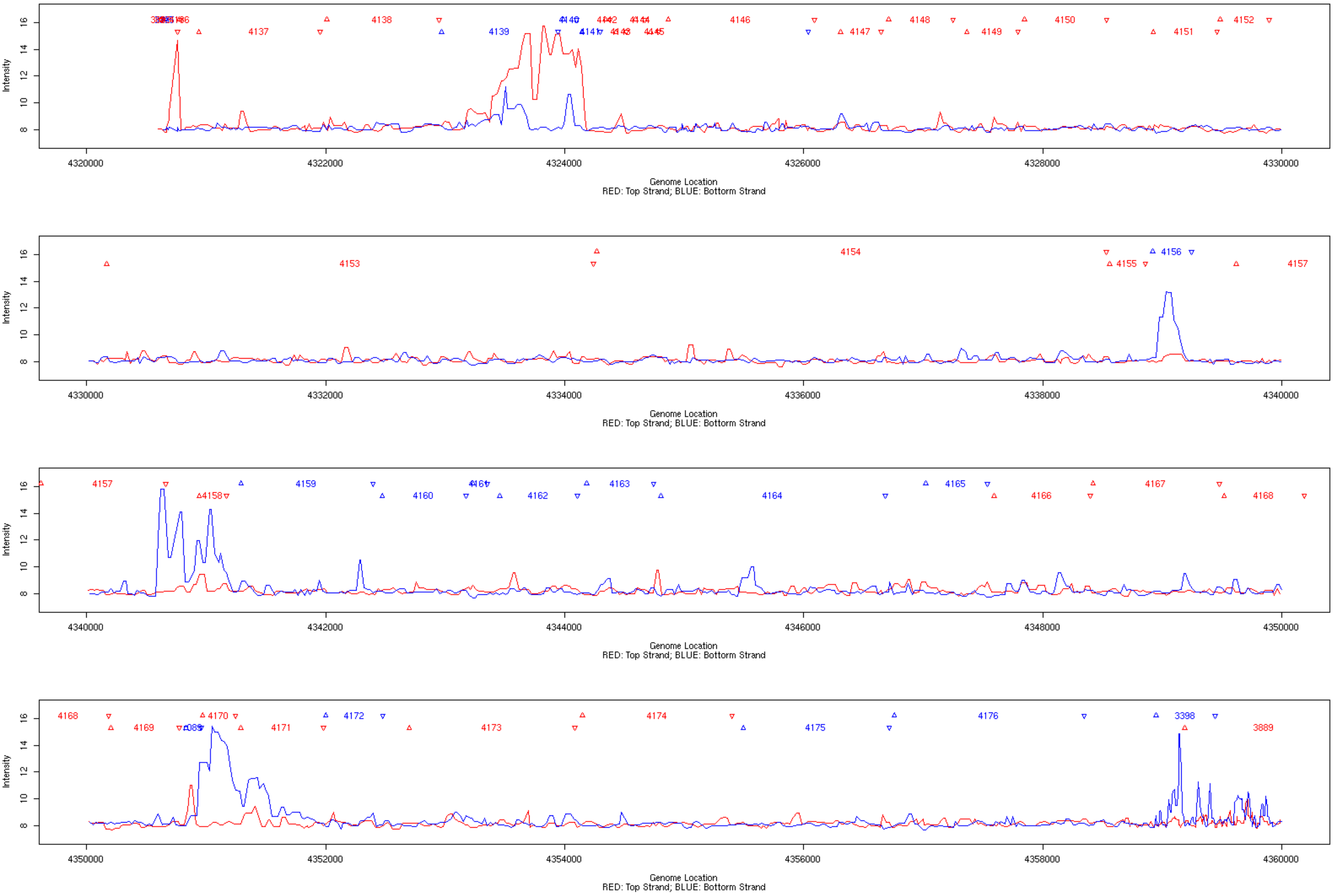
Intensity
Genome Location
RED: Top Strand; BLUE: Bottom Strand
4320000
4322000
4324000
4326000
4328000
4330000
4137
4138
4139
4146
4147
4148
4149
4150
4151
4152
4153
4154
4155
4156
4157
4332000
4334000
4336000
4338000
4340000
4158
4159
4160
4162
4163
4164
4165
4166
4167
4168
4342000
4344000
4346000
4348000
4350000
4169
4170
4171
4172
4173
4174
4175
4176
3398
3889
4352000
4354000
4356000
4358000
4360000

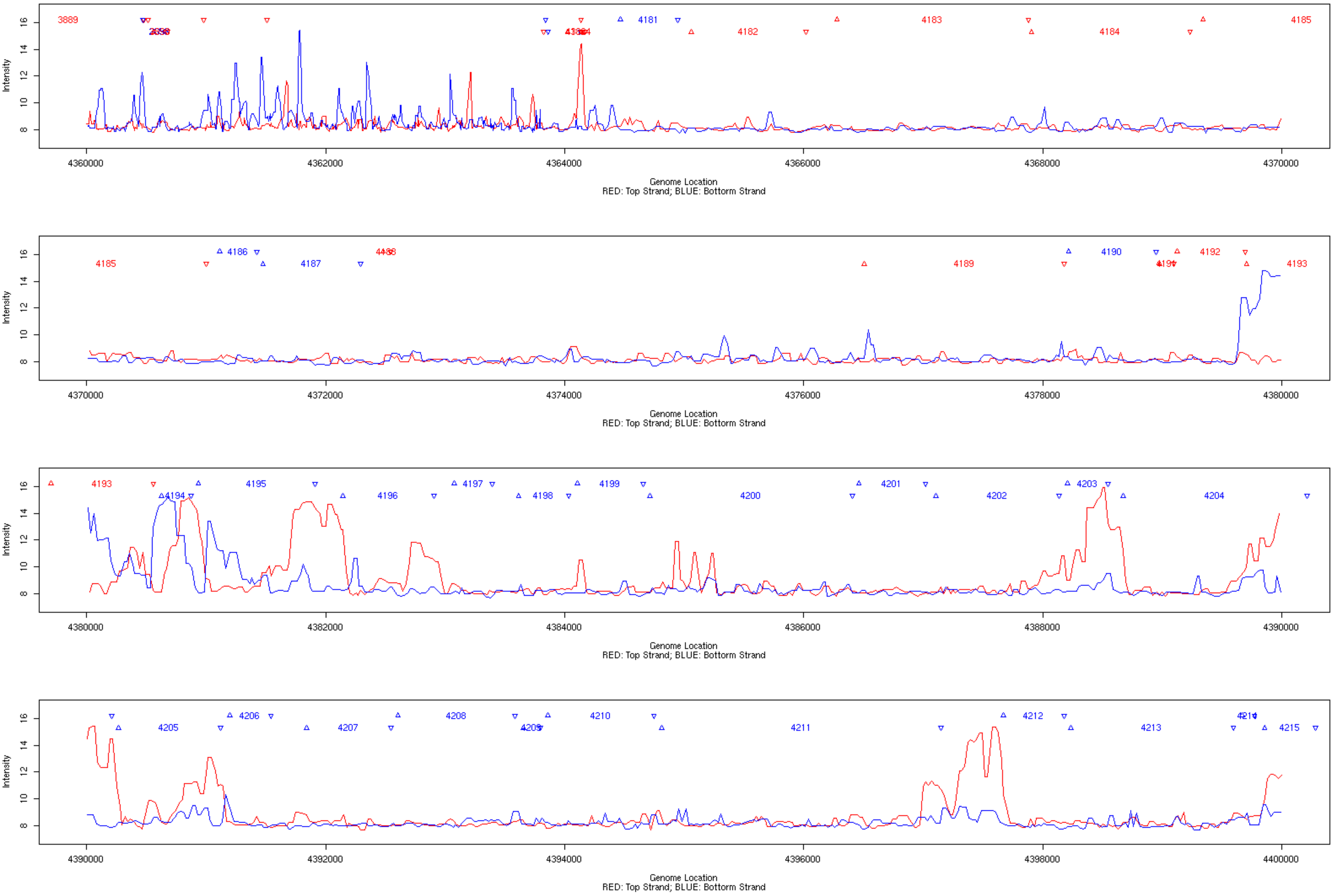

Intensity
3889
4181
4182
4183
4184
4185
4360000
4362000
4364000
4366000
4368000
4370000
Genome Location
RED: Top Strand; BLUE: Bottorm Strand
4185
4186
4187
4189
4190
4192
4193
4370000
4372000
4374000
4376000
4378000
4380000
Genome Location
RED: Top Strand; BLUE: Bottorm Strand
4193
4194
4195
4196
4197
4198
4199
4200
4201
4202
4203
4204
4380000
4382000
4384000
4386000
4388000
4390000
Genome Location
RED: Top Strand; BLUE: Bottorm Strand
4205
4206
4207
4208
4210
4211
4212
4213
4215
4390000
4392000
4394000
4396000
4398000
4400000
Genome Location
RED: Top Strand; BLUE: Bottorm Strand

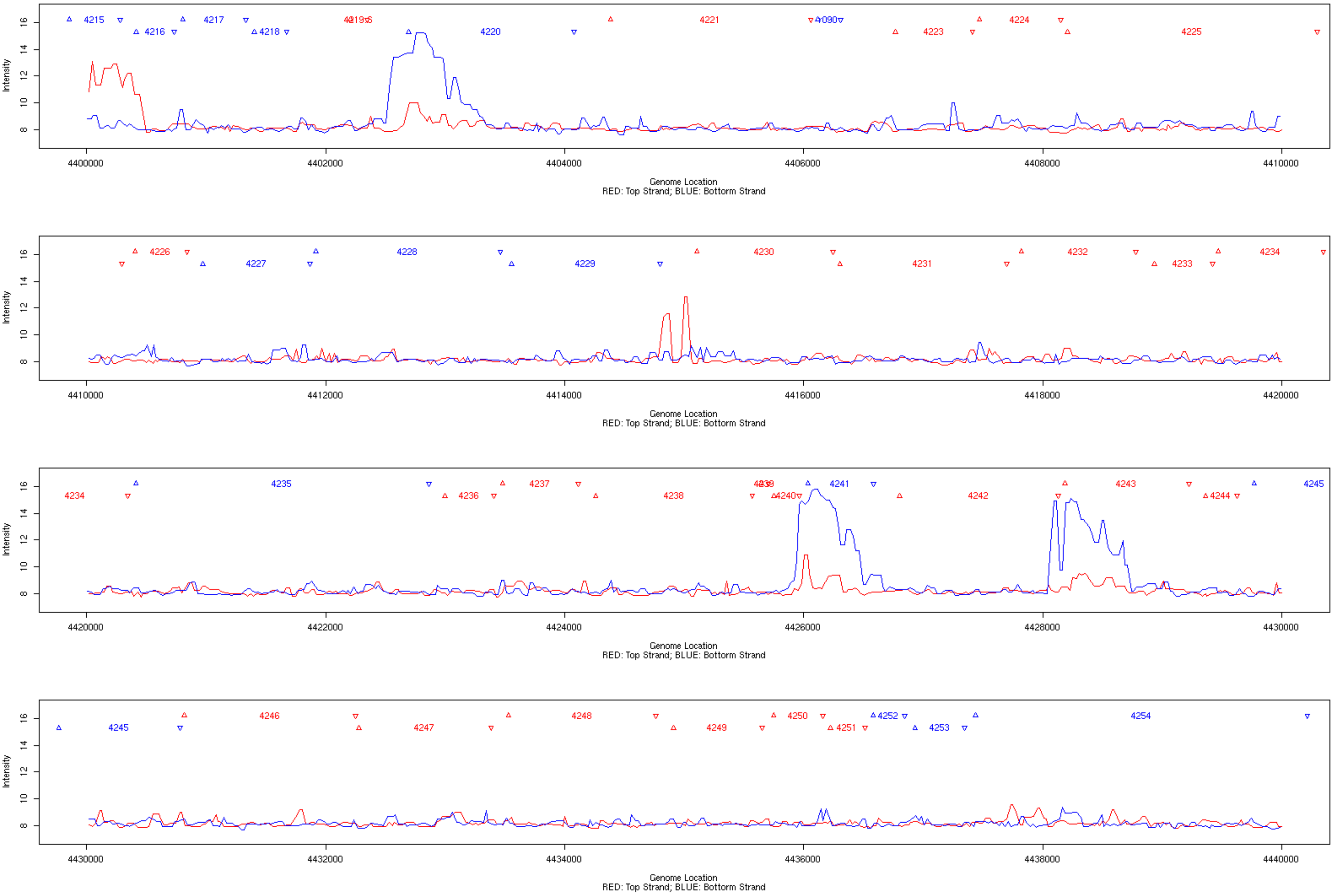
Intensity
Genome Location
RED: Top Strand; BLUE: Bottorm Strand

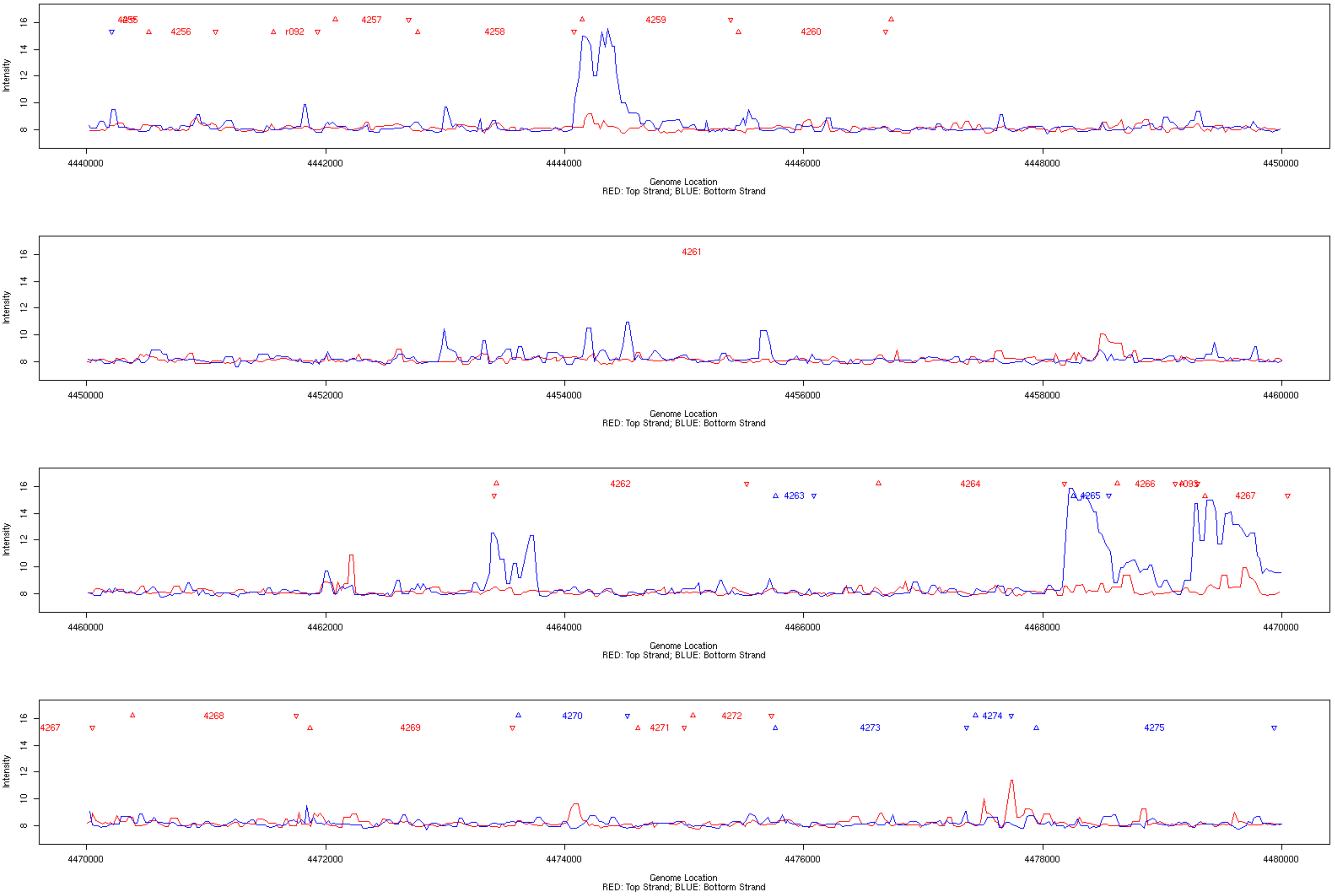

Intensity
4255
4256
r092
4257
4258
4259
4260
4440000
4442000
4444000
4446000
4448000
4450000
Genome Location
RED: Top Strand; BLUE: Bottorm Strand
4261
4452000
4454000
4456000
4458000
4460000
4262
4263
4264
4265
4266
r093
4267
4462000
4464000
4466000
4468000
4470000
4268
4269
4270
4271
4272
4273
4274
4275
4472000
4474000
4476000
4478000
4480000

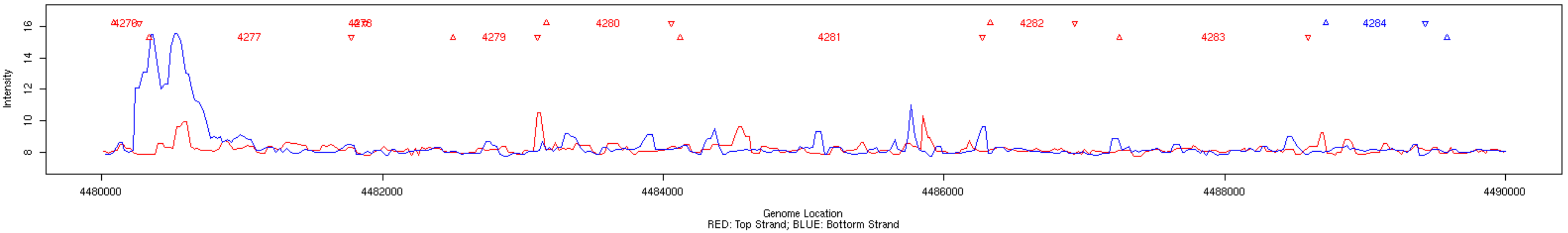
Intensity
Genome Location
RED: Top Strand; BLUE: Bottom Strand
4480000
4482000
4484000
4486000
4488000
4490000
4277
4279
4280
4281
4282
4283
4284

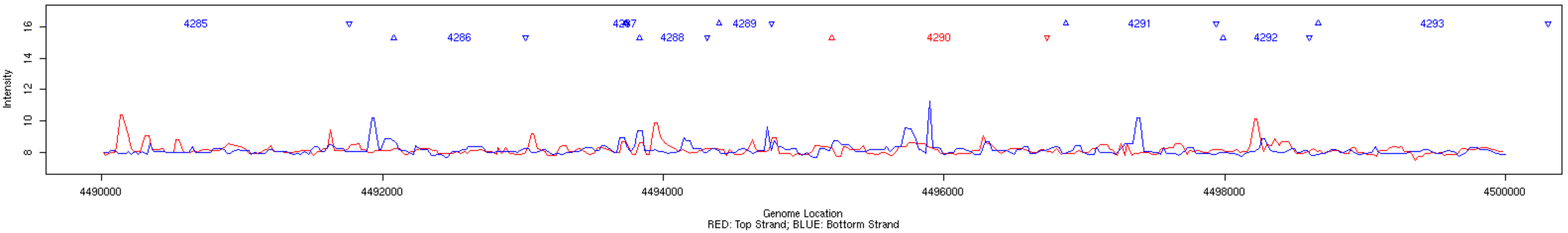
Intensity
Genome Location
RED: Top Strand; BLUE: Bottom Strand
4490000
4492000
4494000
4496000
4498000
4500000
4285
4286
4288
4289
4290
4291
4292
4293

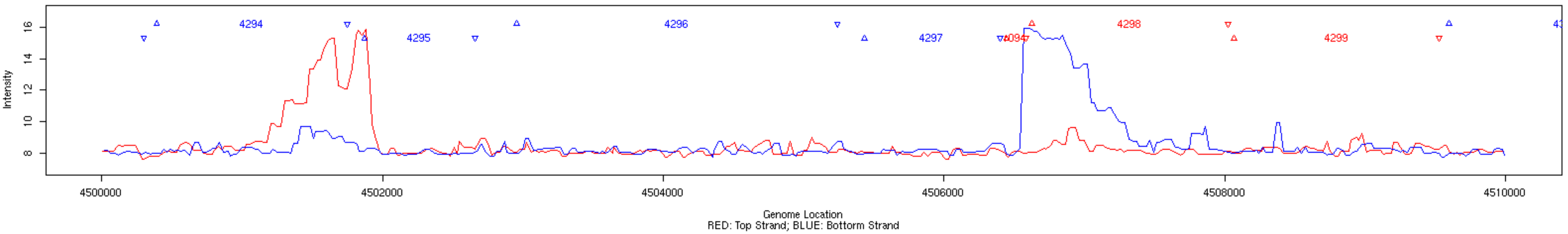
Intensity
Genome Location
RED: Top Strand; BLUE: Bottom Strand
4500000
4502000
4504000
4506000
4508000
4510000
4294
4295
4296
4297
4298
4299

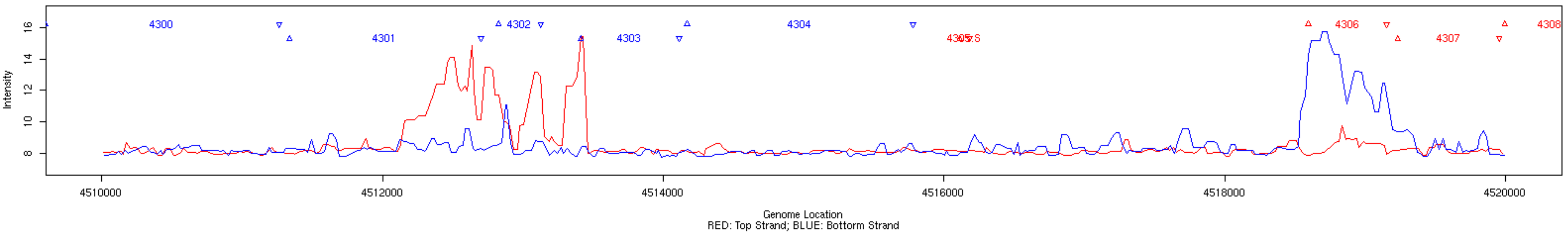
Intensity
Genome Location
RED: Top Strand; BLUE: Bottom Strand
4510000
4512000
4514000
4516000
4518000
4520000
4300
4301
4302
4303
4304
4306
4307
4308

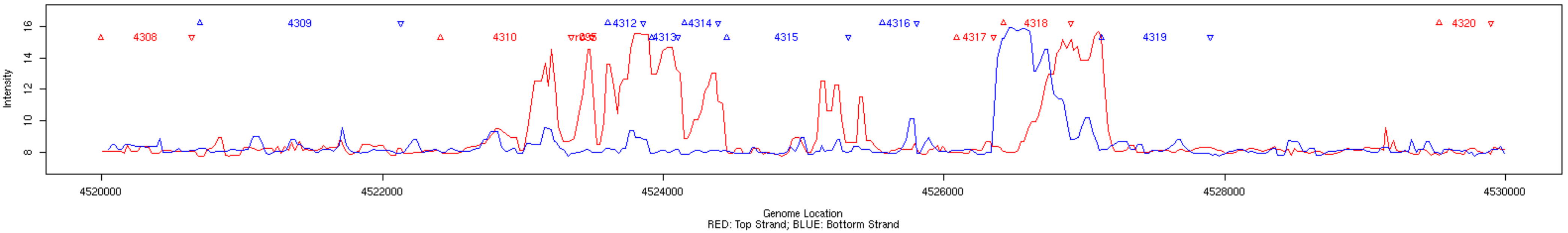
Intensity
4308
4309
4310
4312
4313
4314
4315
4316
4317
4318
4319
4320
4520000
4522000
4524000
4526000
4528000
4530000
Genome Location
RED: Top Strand; BLUE: Bottorm Strand

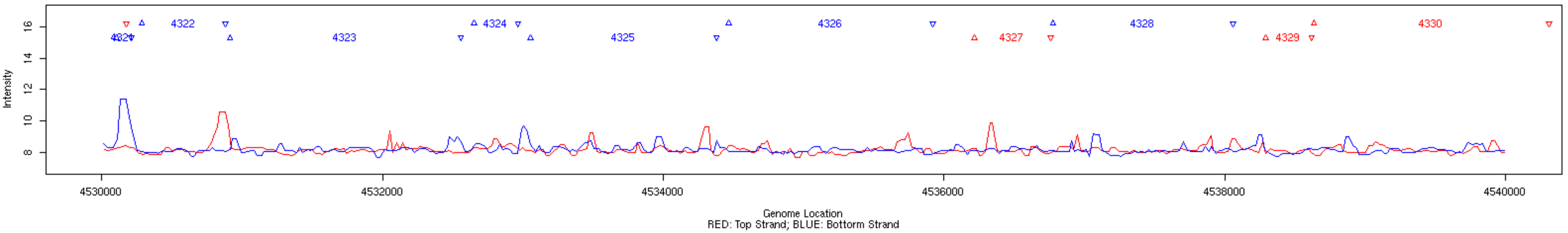
Intensity
4322
4323
4324
4325
4326
4327
4328
4329
4330
4530000
4532000
4534000
4536000
4538000
4540000
Genome Location
RED: Top Strand; BLUE: Bottorm Strand

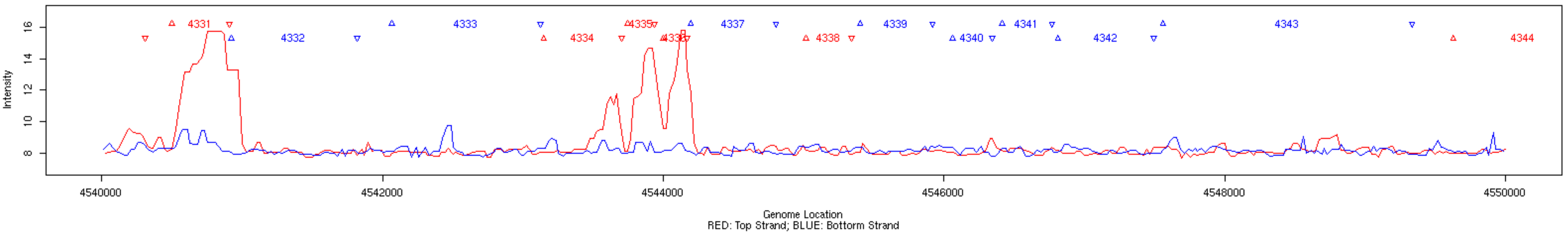
Intensity
4331
4332
4333
4334
4337
4338
4339
4340
4341
4342
4343
4344
4540000
4542000
4544000
4546000
4548000
4550000
Genome Location
RED: Top Strand; BLUE: Bottorm Strand

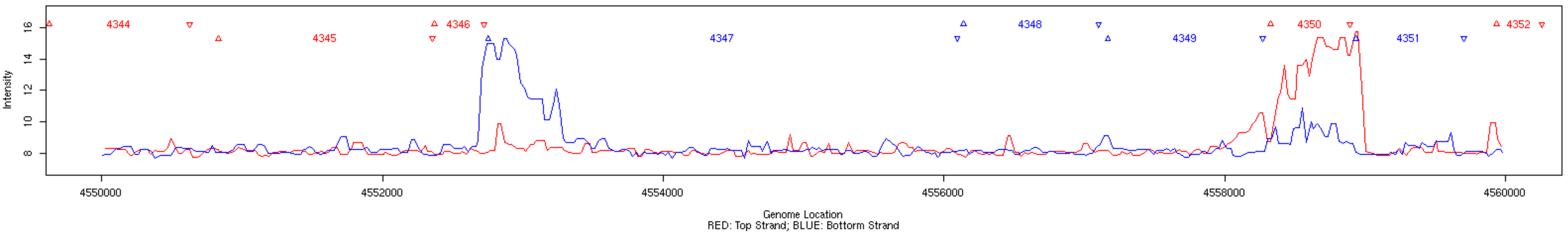
Intensity
4344
4345
4346
4347
4348
4349
4350
4351
4352
4550000
4552000
4554000
4556000
4558000
4560000
Genome Location
RED: Top Strand; BLUE: Bottorm Strand

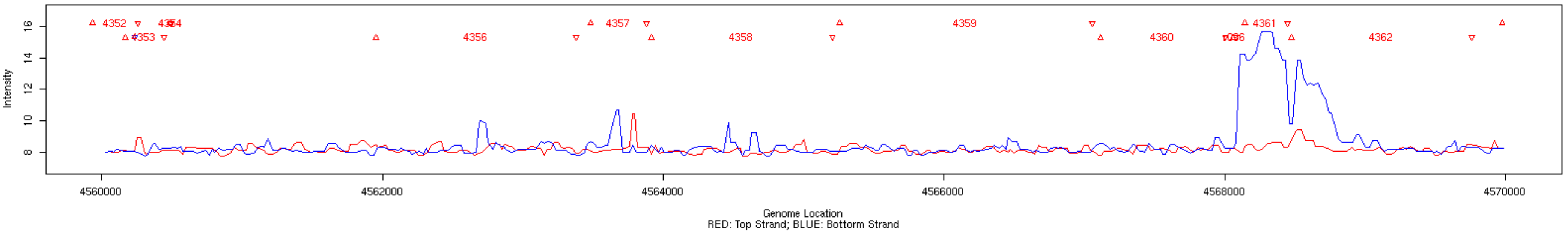

Intensity
4352
4353
4356
4357
4358
4359
4360
4361
4362
4560000
4562000
4564000
4566000
4568000
4570000
Genome Location
RED: Top Strand; BLUE: Bottorm Strand

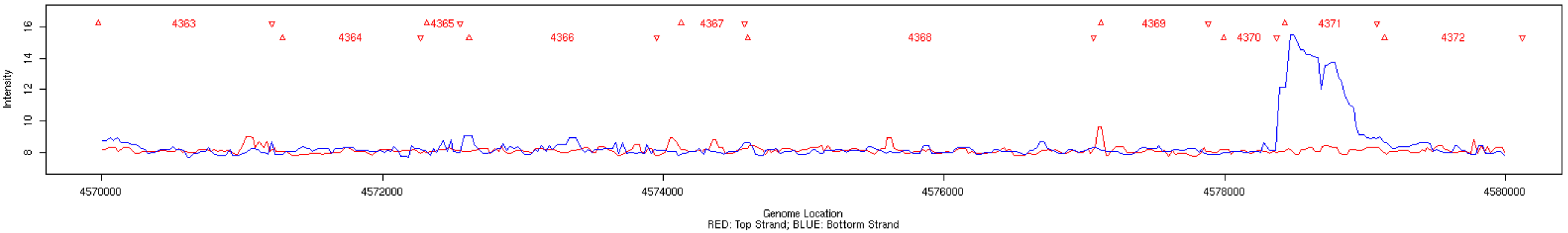

Intensity
4363
4364
4365
4366
4367
4368
4369
4370
4371
4372
4570000
4572000
4574000
4576000
4578000
4580000
Genome Location
RED: Top Strand; BLUE: Bottorm Strand

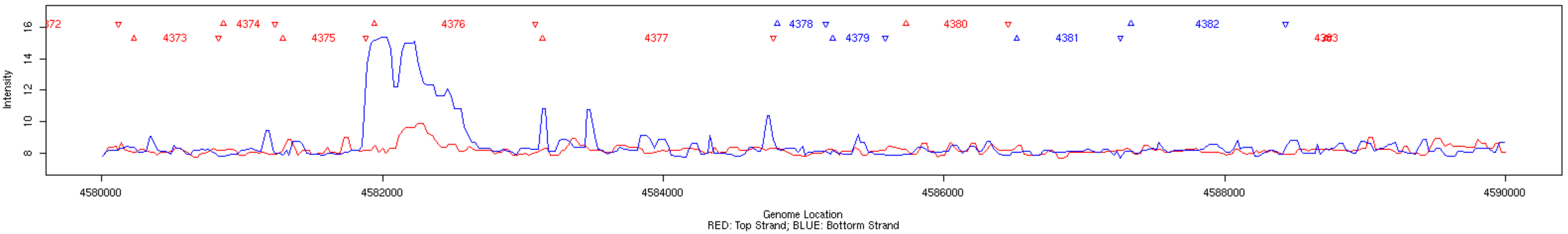

Intensity
4373
4374
4375
4376
4377
4378
4379
4380
4381
4382
4580000
4582000
4584000
4586000
4588000
4590000
Genome Location
RED: Top Strand; BLUE: Bottorm Strand

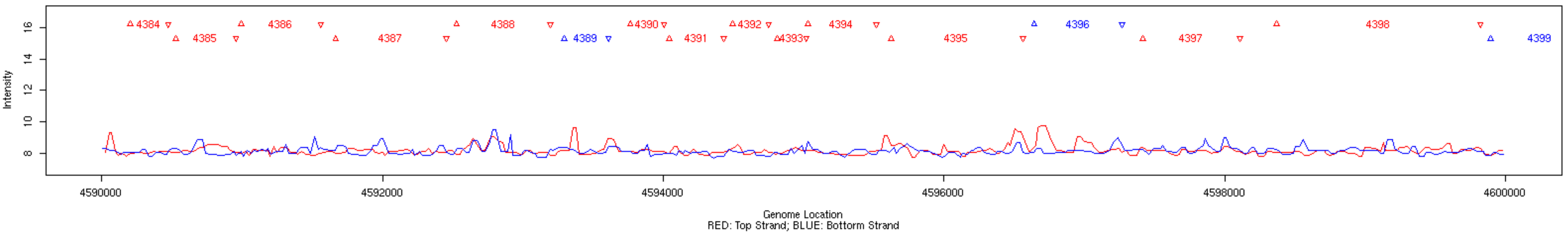

Intensity
4384
4385
4386
4387
4388
4389
4390
4391
4392
4393
4394
4395
4396
4397
4398
4399
4590000
4592000
4594000
4596000
4598000
4600000
Genome Location
RED: Top Strand; BLUE: Bottorm Strand

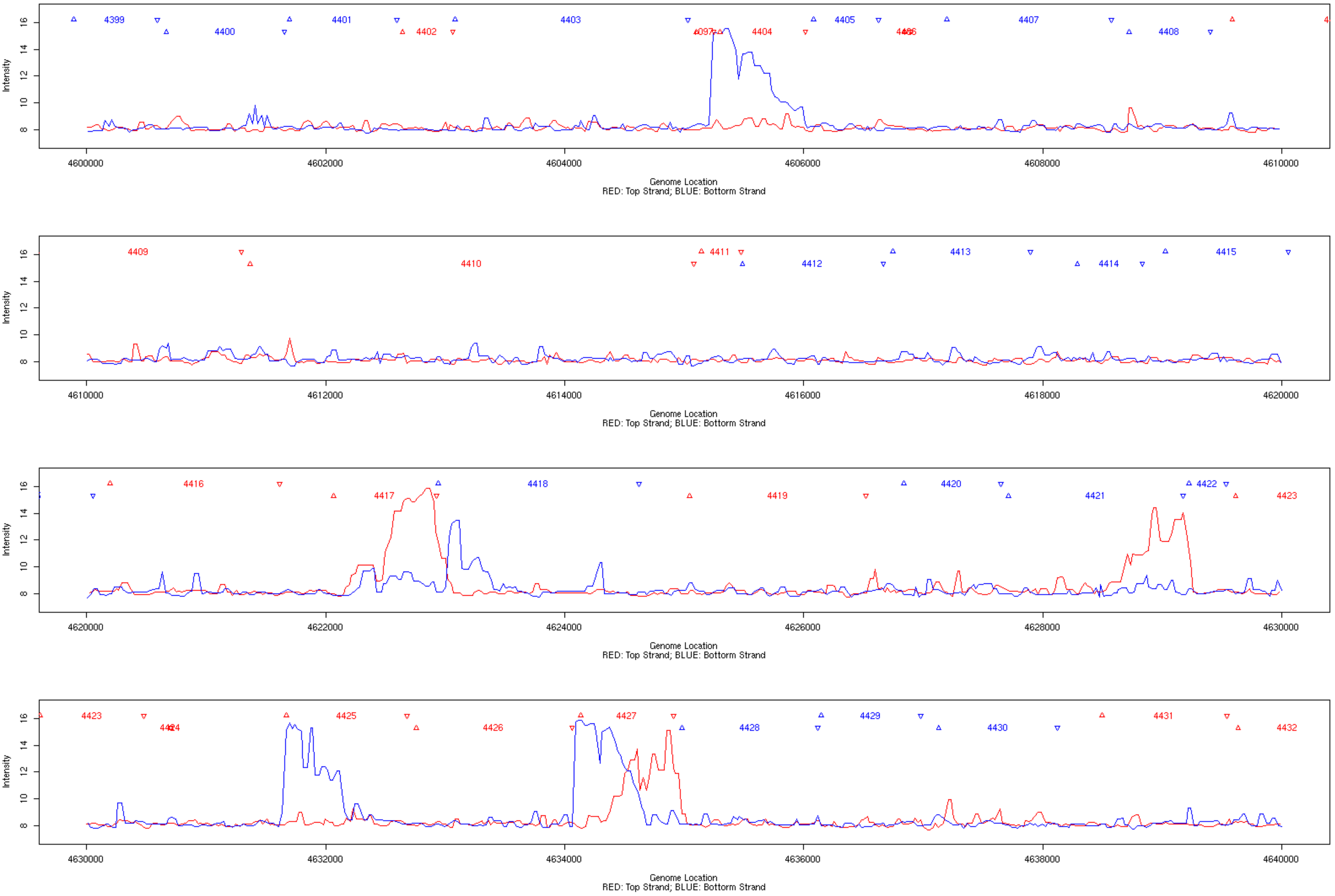
Intensity
Genome Location
RED: Top Strand; BLUE: Bottorm Strand
4399
4400
4401
4402
4403
4404
4405
4407
4408
4409
4410
4411
4412
4413
4414
4415
4416
4417
4418
4419
4420
4421
4422
4423
4425
4426
4427
4428
4429
4430
4431
4432

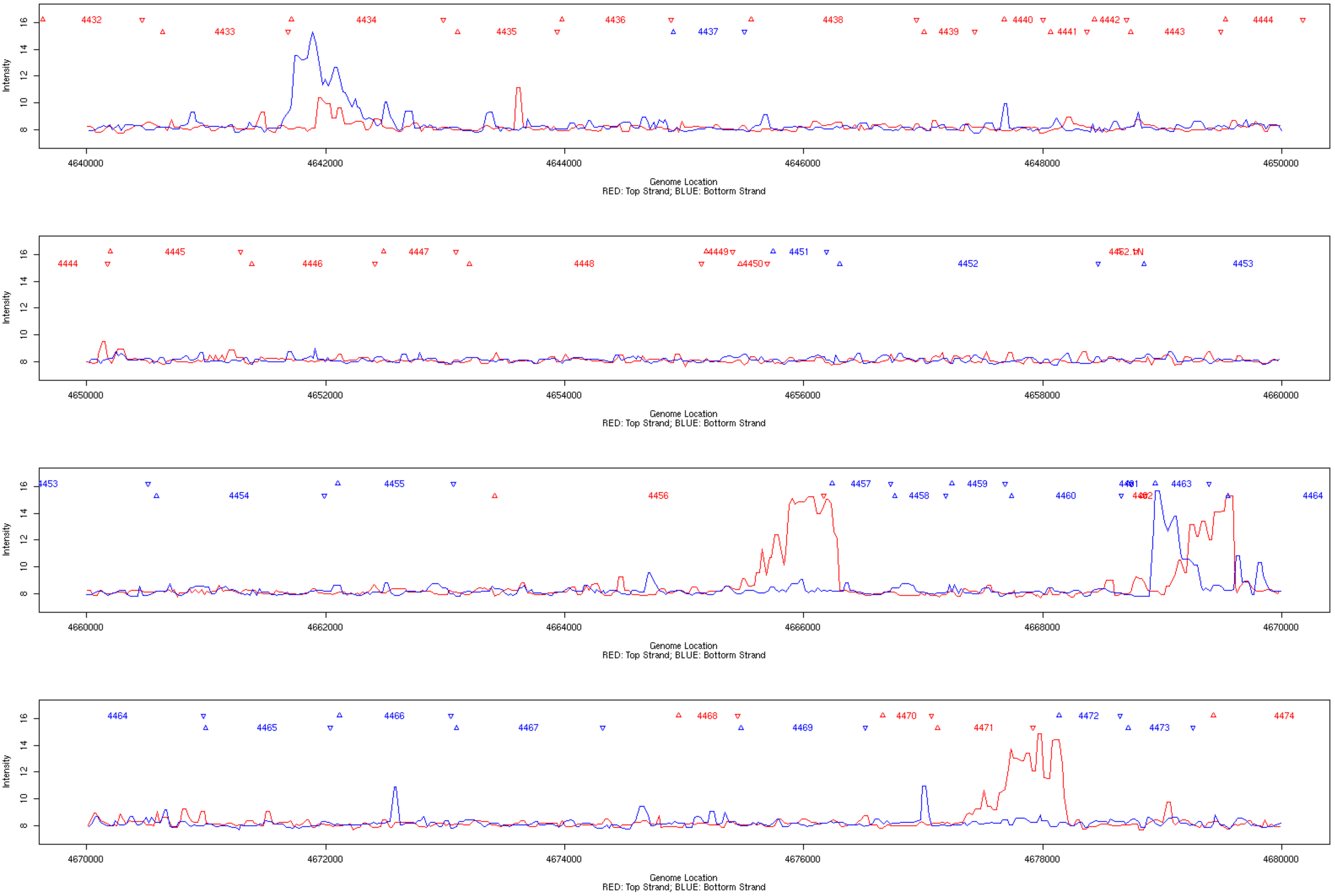
Intensity
Genome Location
RED: Top Strand; BLUE: Bottorm Strand
4432
4433
4434
4435
4436
4437
4438
4439
4440
4441
4442
4443
4444
4445
4446
4447
4448
4449
4450
4451
4452
4453
4454
4455
4456
4457
4458
4459
4460
4463
4464
4465
4466
4467
4468
4469
4470
4471
4472
4473
4474

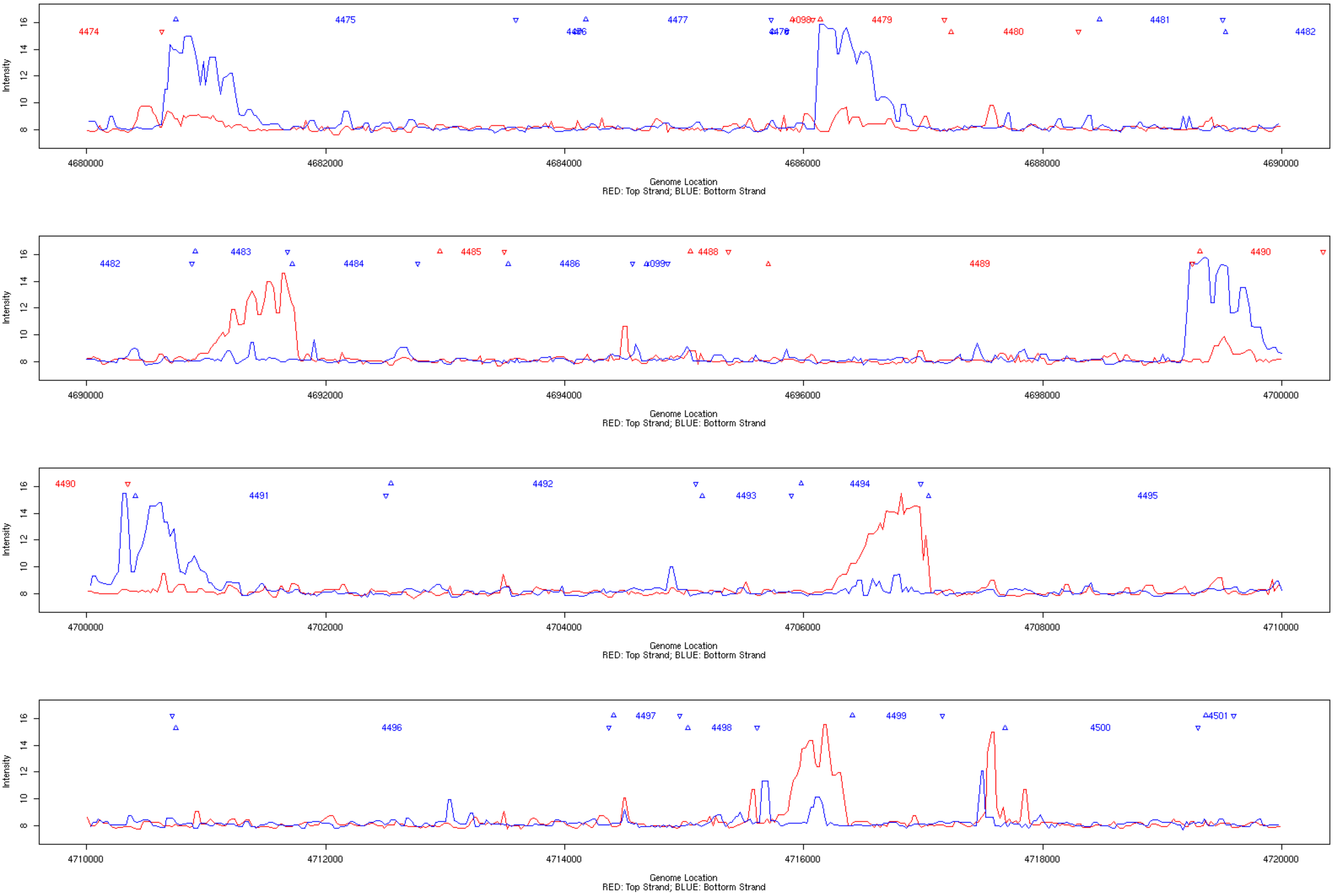
Intensity
Genome Location
RED: Top Strand; BLUE: Bottorm Strand
4474
4475
4477
4479
4480
4481
4482
4483
4484
4485
4486
4488
4489
4490
4491
4492
4493
4494
4495
4496
4497
4498
4499
4500
4501
4680000
4682000
4684000
4686000
4688000
4690000
4692000
4694000
4696000
4698000
4700000
4702000
4704000
4706000
4708000
4710000
4712000
4714000
4716000
4718000
4720000

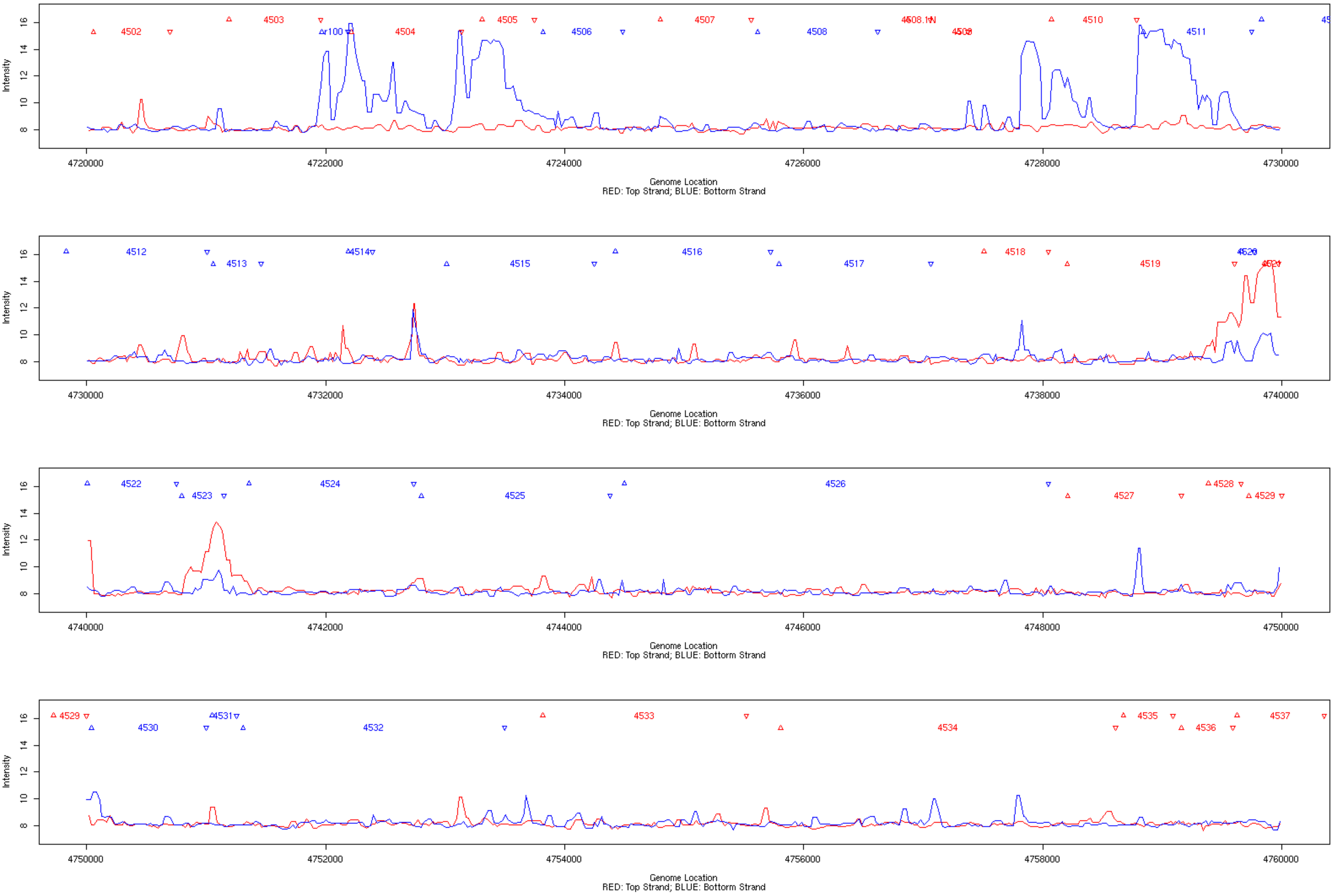
Intensity
Genome Location
RED: Top Strand; BLUE: Bottorm Strand
4502
4503
4504
4505
4506
4507
4508
4510
4511
4512
4513
4515
4516
4517
4518
4519
4522
4523
4524
4525
4526
4527
4528
4529
4530
4532
4533
4534
4535
4536
4537
4720000
4722000
4724000
4726000
4728000
4730000
4732000
4734000
4736000
4738000
4740000
4742000
4744000
4746000
4748000
4750000
4752000
4754000
4756000
4758000
4760000

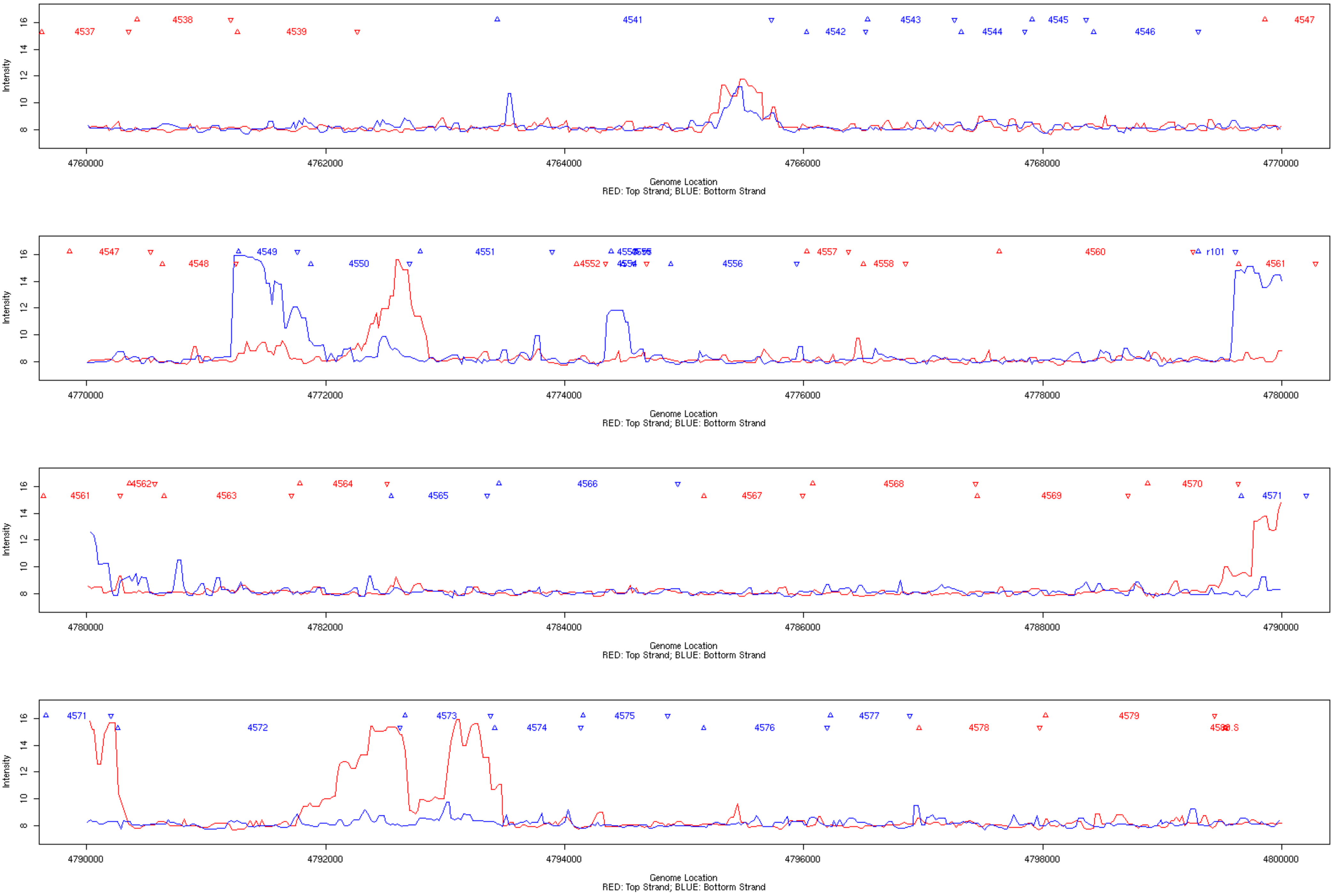

Intensity
4537
4538
4539
4541
4542
4543
4544
4545
4546
4547
4760000
4762000
4764000
4766000
4768000
4770000
Genome Location
RED: Top Strand; BLUE: Bottom Strand
Intensity
4547
4548
4549
4550
4551
4552
4556
4557
4558
4560
r101
4561
4770000
4772000
4774000
4776000
4778000
4780000
Genome Location
RED: Top Strand; BLUE: Bottom Strand
Intensity
4561
4562
4563
4564
4565
4566
4567
4568
4569
4570
4571
4780000
4782000
4784000
4786000
4788000
4790000
Genome Location
RED: Top Strand; BLUE: Bottom Strand
Intensity
4571
4572
4573
4574
4575
4576
4577
4578
4579
4790000
4792000
4794000
4796000
4798000
4800000
Genome Location
RED: Top Strand; BLUE: Bottom Strand

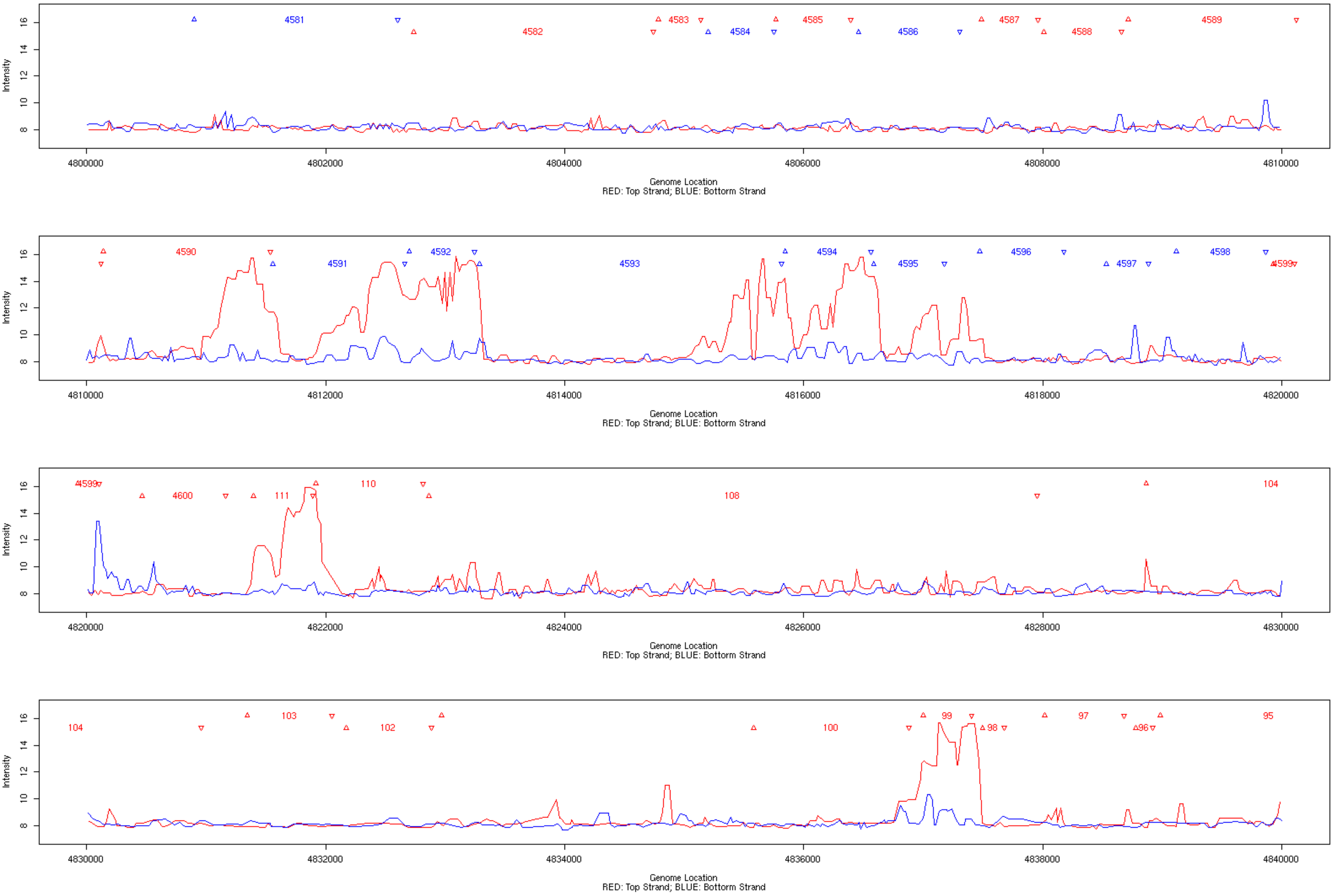

Intensity
Genome Location
RED: Top Strand; BLUE: Bottom Strand
4581
4582
4583
4584
4585
4586
4587
4588
4589
4590
4591
4592
4593
4594
4595
4596
4597
4598
4599
4600
111
110
108
104
103
102
100
99
98
97
96
95

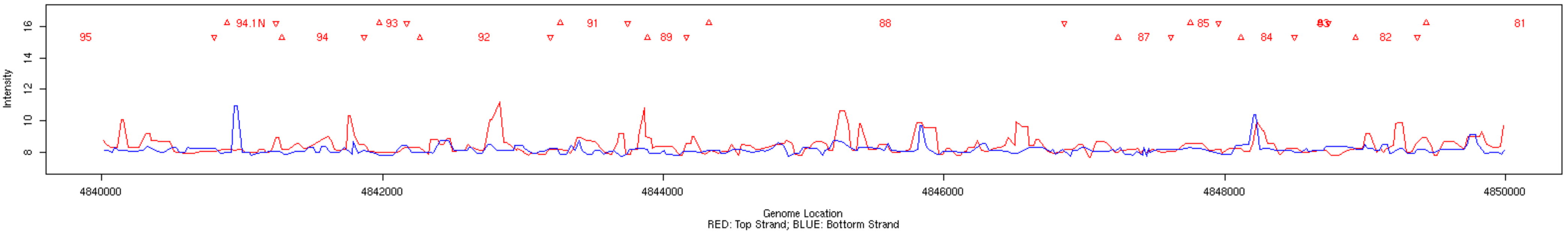
Intensity
95
94.1N
94
93
92
91
89
88
87
85
84
82
81
4840000
4842000
4844000
4846000
4848000
4850000
Genome Location
RED: Top Strand; BLUE: Bottorm Strand

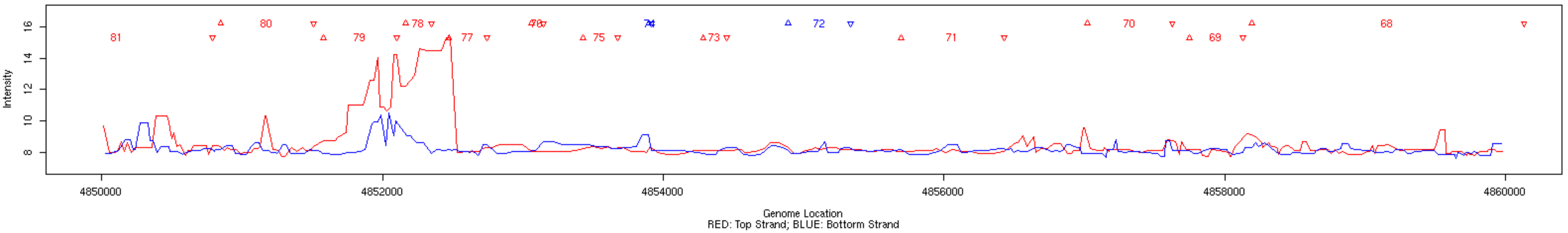
Intensity
81
80
79
78
77
75
73
72
71
70
69
68
4850000
4852000
4854000
4856000
4858000
4860000
Genome Location
RED: Top Strand; BLUE: Bottorm Strand

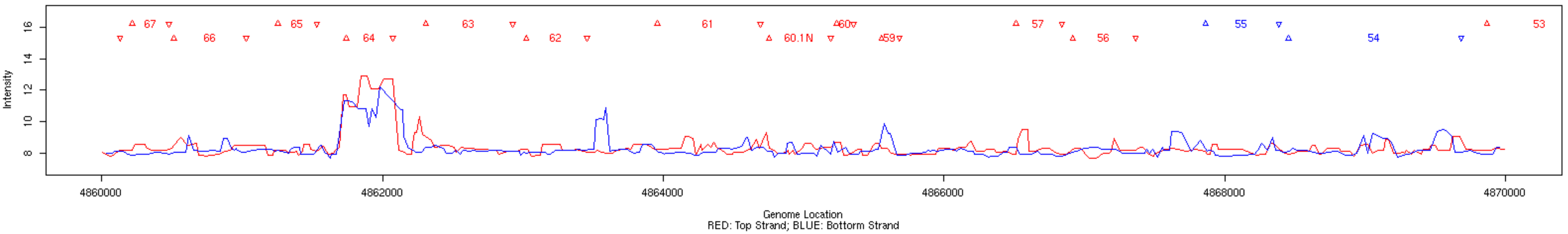
Intensity
67
66
65
64
63
62
61
60.1N
57
56
55
54
53
4860000
4862000
4864000
4866000
4868000
4870000
Genome Location
RED: Top Strand; BLUE: Bottorm Strand

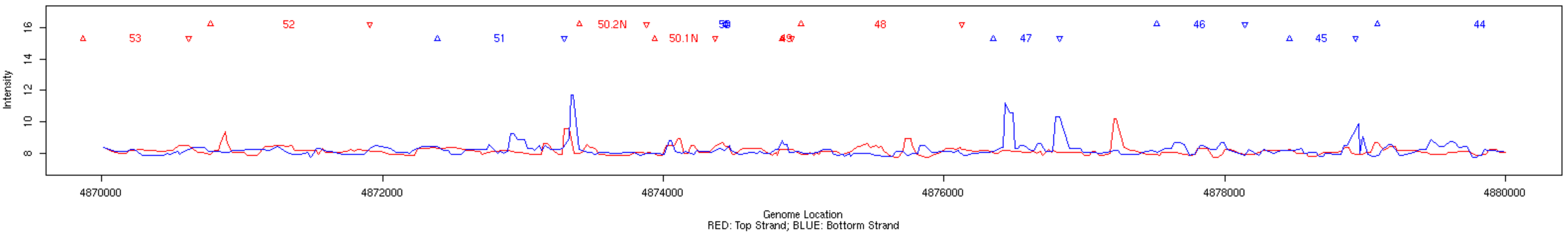
Intensity
53
52
51
50.2N
50.1N
48
47
46
45
44
4870000
4872000
4874000
4876000
4878000
4880000
Genome Location
RED: Top Strand; BLUE: Bottorm Strand

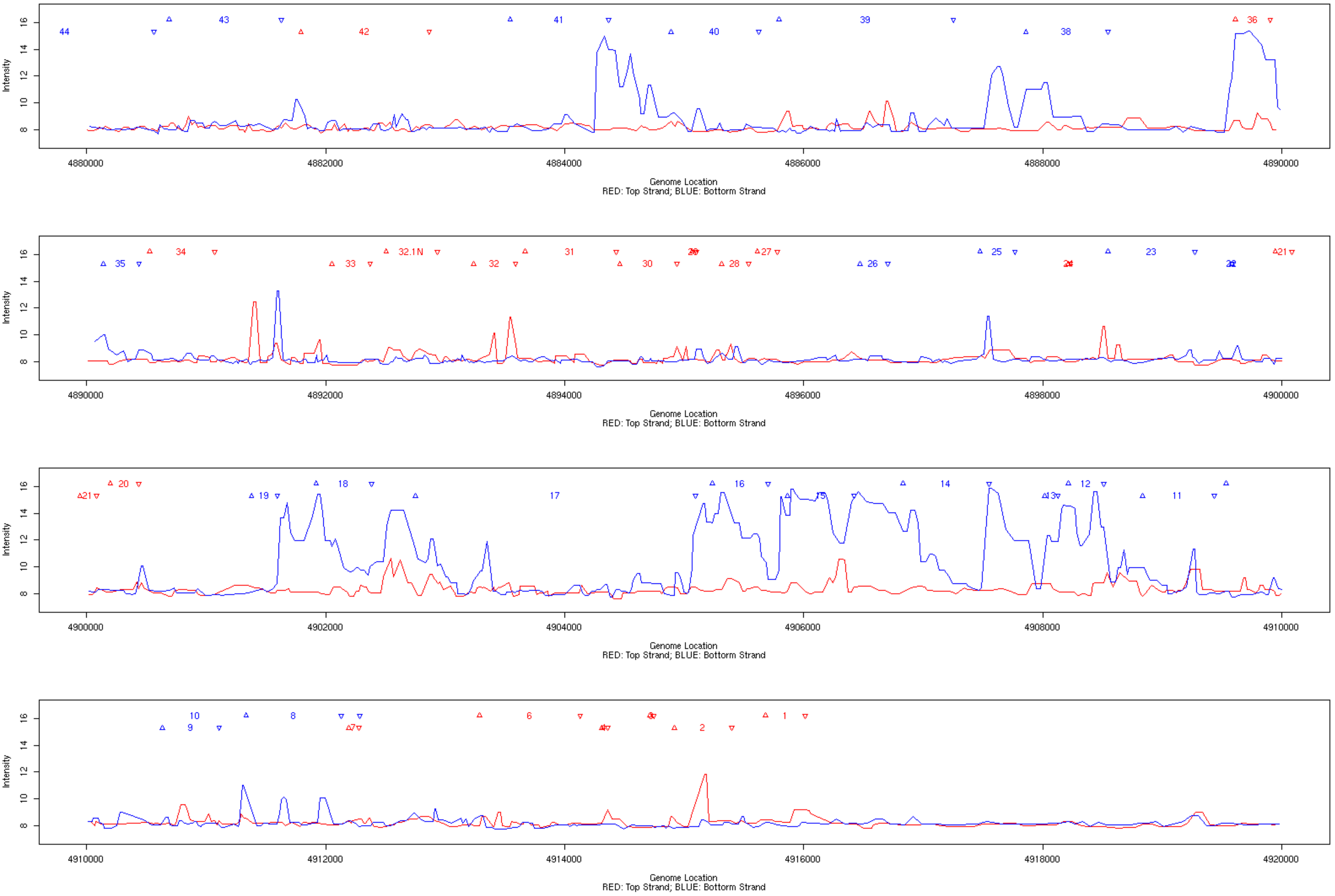
Intensity
Genome Location
RED: Top Strand; BLUE: Bottorm Strand
Intensity
Genome Location
RED: Top Strand; BLUE: Bottorm Strand
Intensity
Genome Location
RED: Top Strand; BLUE: Bottorm Strand
Intensity
Genome Location
RED: Top Strand; BLUE: Bottorm Strand

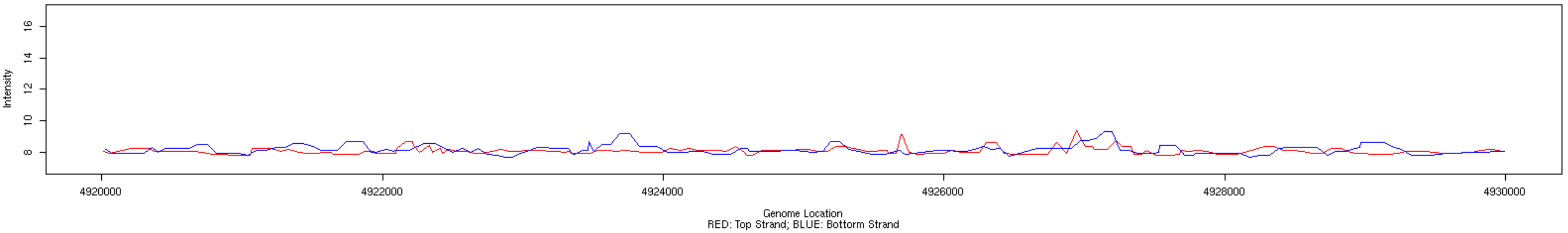
Intensity
8
10
12
14
16
4920000
4922000
4924000
4926000
4928000
4930000
Genome Location
RED: Top Strand; BLUE: Bottorm Strand

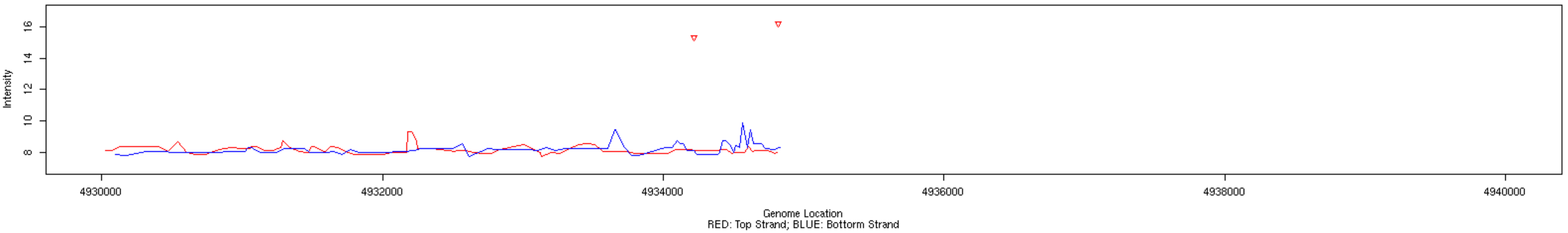
Intensity
8
10
12
14
16
4930000
4932000
4934000
4936000
4938000
4940000
Genome Location
RED: Top Strand; BLUE: Bottorm Strand
