# Supplementary material for: Analysis of Pools of Targeted Salmonella Deletion Mutants Identifies Novel Genes Affecting Fitness during Competitive Infection in Mice
Source: PLoS Pathog. 2009 Jul 3;5(7):e1000477. doi: 10.1371/journal.ppat.1000477 (PMC2698986; doi:10.1371/journal.ppat.1000477)

Figure S2. Addition of the 27mer competitor oligo results in high specificity of array detection.

Each RNA transcript from the T7 promoter contains a 27 base sequence encoded in the cassette used for mutagenesis and therefore present in the transcript from all mutants. The complement of the last 20 bases of this sequence is also present in every 3' oligo used on the array. To block the interaction between the complementary labeled RNA and array oligonucleotide sequences, a 27-mer DNA that is complementary to the common region in all the T7 RNAs was added to the hybridization.

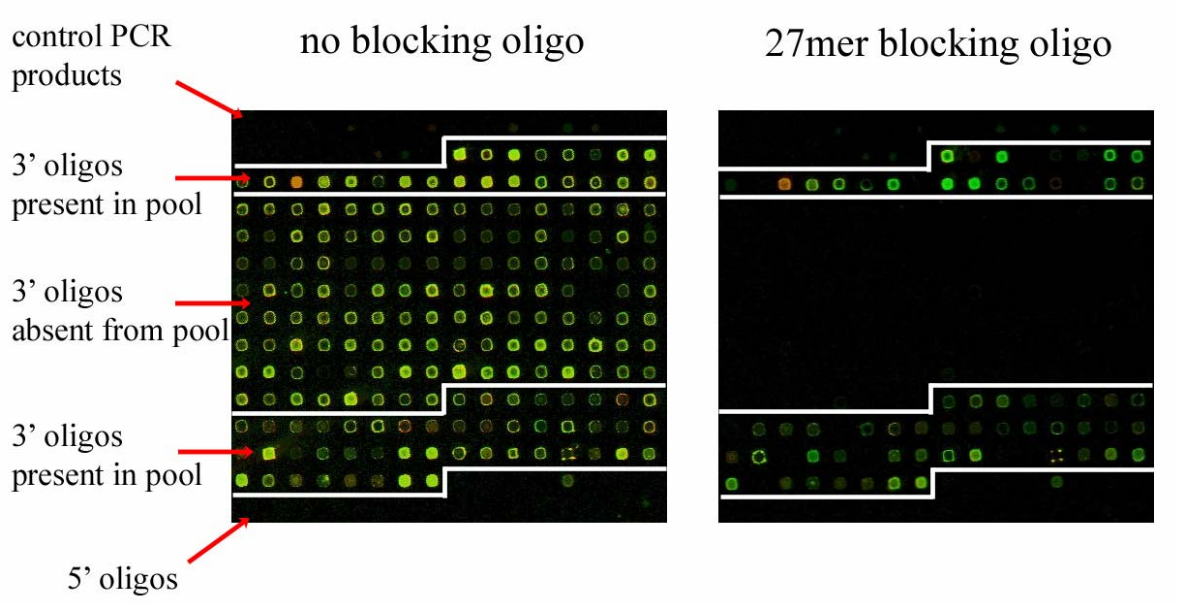

Supplement: Figure S2 — Addition of the 27mer competitor oligo results in high specificity of array detection. (0.10 MB PDF) [file ppat.1000477.s002.pdf]
