# Supplementary material for: Analysis of Pools of Targeted Salmonella Deletion Mutants Identifies Novel Genes Affecting Fitness during Competitive Infection in Mice
Source: PLoS Pathog. 2009 Jul 3;5(7):e1000477. doi: 10.1371/journal.ppat.1000477 (PMC2698986; doi:10.1371/journal.ppat.1000477)
Supplement: Table S2 — Specificity and sensitivity of array-based detection of mutants in a pool. (0.01 MB PDF) [file ppat.1000477.s004.pdf]

Table S2. Specificity and sensitivity of array-based detection of mutants in a pool. Data shown are median values from six hybridizations of input samples, including three different preparations, and dye swaps. Inputs were generated from a pool of 1,031 Kan^R gene deletion mutants grown to stationary phase in LB Kan. Hybridizations included a 27mer competitor oligo (see Materials & Methods, and **Supplementary Figure 2**)

	**Number of probes detected (%)***
3' gene oligos for mutants in pool	905 / 933 (97.0)
3' gene oligos for mutants not in pool (negative controls)	8 / 308 (2.6)
5' gene oligos used to make deletions (negative controls)	7 / 955 (0.7)

* normalized signal three-fold above median of negative controls.
