# Supplementary material for: Analysis of Pools of Targeted Salmonella Deletion Mutants Identifies Novel Genes Affecting Fitness during Competitive Infection in Mice
Source: PLoS Pathog. 2009 Jul 3;5(7):e1000477. doi: 10.1371/journal.ppat.1000477 (PMC2698986; doi:10.1371/journal.ppat.1000477)
Supplement: Table S3 — Details of the Competitive Index data. (0.03 MB PDF) [file ppat.1000477.s005.pdf]

Table S3: Details of the Competitive Index data.

Gene	Mutant	Locus	ABACUS (pool of 933 mutants)		Competition with wt (single mutant)		
			ratio spleen / input	p-value	CI	standard error	p-value
STM0731	Δ*STM0731*::*kan*	Spleen	**0.18**	**2.E-4**			
STM0732	[1]Δ*STM0732*::*kan*	Spleen*			0.68	0.06	**5.E-4**
		Liver*			0.55	0.07	7.E-2
		Cecum*			0.74	0.10	**7.E-3**
STM0857	Δ*STM0857*::*kan*	Spleen	**0.47**	**8.E-3**	1.28	0.03	**2.E-3**
		Liver			1.37	0.04	**1.E-2**
		Cecum			1.08	0.02	**7.E-3**
STM1131	[2]Δ*STM1131*::*kan*	Spleen*	**0.44**	**1.E-4**	0.0008	0.78	**2.E-2**
		Liver*			0.002	0.25	**5.E-4**
		Cecum*			0.007	0.78	6.E-2
	Δ*STM1131*::FRT	Spleen			**0.37**	0.06	**2.E-4**
		Liver			0.58	0.07	**3.E-3**
		Cecum			**0.32**	0.40	2.E-1
STM1760	Δ*STM1760*::*kan*	Spleen*	**0.19**	**1.E-6**	**0.35**	0.17	**4.E-2**
		Liver*			0.59	0.22	3.E-1
		Cecum*			**0.46**	0.24	2.E-1
STM2120	Δ*STM2120*::*kan*	Spleen	**0.22**	**9.E-12**	**0.09**	0.06	**1.E-4**
		Liver			**0.07**	0.08	**2.E-4**
		Cecum			**0.04**	0.25	**6.E-3**
STM2215	Δ*STM2215*::*kan*	Spleen*	**0.12**	**2.E-8**	**0.01**	0.14	**1.E-4**
		Liver*			**0.01**	0.14	**1.E-4**
		Cecum*			**0.02**	0.22	**1.E-3**
STM2303	Δ*STM2303*::*kan*	Spleen	**0.08**	**6.E-12**	**0.12**	0.08	**3.E-4**
		Liver			**0.27**	0.14	**2.E-2**
		Cecum			**0.20**	0.16	**2.E-2**
	Δ*STM2303*::FRT	Spleen			**0.22**	0.09	**1.E-3**
		Liver			**0.32**	0.24	9.E-2
		Cecum			1.42	0.22	5.E-1
	Δ*STM2303*::FRT pWSK29	Spleen			**0.23**	0.04	**1.E-4**
		Liver			**0.40**	0.09	**1.E-2**
		Cecum			0.52	0.08	**2.E-2**
	Δ*STM2303*::FRT pWSK29::*STM2303*	Spleen			1.51	0.04	**3.E-2**
		Liver			2.18	0.04	**2.E-3**
		Cecum			4.76	0.14	**8.E-3**
STM2639	Δ*STM2639*::*kan*	Spleen*	**0.15**	**5.E-8**	1.22	0.04	5.E-1
		Liver*			1.31	0.05	3.E-1
		Cecum*			1.20	0.25	9.E-1
STM3120	Δ*STM3120*::*kan*	Spleen*	**0.19**	**1.E-6**	**0.13**	0.15	**1.E-2**
		Liver*			**0.28**	0.04	**9.E-4**
		Cecum*			**0.35**	0.09	**2.E-2**
	Δ*STM3120*::FRT	Spleen			**0.22**	0.04	**<1.E-7**
		Liver			**0.17**	0.10	**7.E-4**

		Cecum			**0.06**	0.25	**6.E-3**
STM3121	Δ*STM3121*::*kan*	Spleen	**0.38**	**1.E-5**	**0.31**	0.11	**4.E-3**
		Liver			**0.28**	0.06	**2.E-4**
		Cecum			**0.27**	0.26	9.E-2
	Δ*STM3121*::FRT	Spleen			**0.07**	0.07	**8.E-6**
		Liver			**0.08**	0.05	**2.E-6**
		Cecum			**0.03**	0.10	**2.E-5**
	Δ*STM3121*::FRT pWSK29	Spleen			**0.14**	0.08	**1.E-4**
		Liver			**0.22**	0.19	**1.E-2**
		Cecum			**0.25**	0.32	9.E-2
	Δ*STM3121*::FRT pWSK29::*STM3121*	Spleen			3.75	0.09	**1.E-2**
		Liver			4.83	0.15	**3.E-2**
		Cecum			5.32	0.33	2.E-1
leuX	Δ*leuX*::*kan*	Spleen	**0.05**	**6.E-12**	**0.0001**	0.14	**<1.E-7**
		Liver			**0.0002**	0.14	**<1.E-7**
		Cecum					
	Δ*leuX*::FRT	Spleen			**0.01**	0.12	**<1.E-7**
		Liver			**0.01**	0.16	**<1.E-7**
		Cecum			**0.05**	0.29	**6.E-3**
istR	Δ*istR*::*kan*	Spleen	**0.41**	**5.E-6**	**0.34**	0.12	**3.E-2**
		Liver			**0.24**	0.22	7.E-2
		Cecum					
	Δ*istR*::FRT	Spleen			**0.42**	0.09	**1.E-2**
		Liver			**0.41**	0.09	**1.E-2**
		Cecum			**0.03**	0.40	**2.E-2**
sroA	Δ*sroA*::*kan*	Spleen	**0.13**	**2.E-10**	**0.81**	0.04	**5.E-3**
		Liver			1.34	0.05	6.E-1
		Cecum					
	Δ*sroA*::FRT	Spleen			**0.24**	0.04	**<1.E-7**
		Liver			**0.21**	0.05	**4.E-3**
		Cecum			**0.09**	0.25	**4.E-3**
oxyS	Δ*oxyS*::*kan*	Spleen	**2.30**	**6.E-6**	1.67	0.04	**3.E-3**
		Liver			1.23	0.17	7.E-1
		Cecum					
	Δ*oxyS*::FRT	Spleen			1.74	0.06	**3.E-3**
		Liver			1.34	0.07	**2.E-2**
		Cecum			0.68	0.29	8.E-1
rybB	Δ*rybB*::*kan*	Spleen	**0.50**	**6.E-4**	1.79	0.09	2.E-2
		Liver			1.24	0.14	4.E-1
		Cecum					
sraA	Δ*sraA*::*kan*	Spleen	**0.35**	**1.E-7**	1.03	0.02	**1.E-2**
		Liver			0.63	0.06	**4.E-3**
		Cecum					

Red indicates either P<0.05 or a ratio of greater than two-fold for mutants with reduced fitness in systemic infection. Blue indicates the same thresholds for mutants with increased fitness after selection.
,*Indicates mice were euthanized later than 24 but prior to 48 hours in CI experiments.
[1] overlapping mutant with STM0731

[2]Δ*STM1131*::*kan,* the P22 transduced version of this mutant used in CI experiments, was discovered to have rough LPS. Δ*STM1131*::*FRT* was generated by re-transduction of our original red-swap mutant, and does not have rough LPS (data not shown).
